# Intergenerational effects of early life-stage temperature modulation on gene expression and DNA methylation in Atlantic cod (*Gadus morhua*)

**DOI:** 10.1080/15592294.2023.2237759

**Published:** 2023-07-27

**Authors:** Velmurugu Puvanendran, Erik Burgerhout, Øivind Andersen, Matthew Kent, Øyvind Hansen, Torstein Tengs

**Affiliations:** a Nofima AS, Tromsø, Norway; b Department of Animal and Aquacultural Sciences, Centre for Integrative Genetics (CIGENE), Faculty of Biosciences, Norwegian University of Life Sciences, Ås, Norway

**Keywords:** Atlantic cod, epigenetics, temperature, aquaculture, intergenerational

## Abstract

After suffering several collapses, the cod farming industry is now in the process of trying to re-establish itself. We have used material from Norway’s National Cod Breeding Program to study how different early life-stage temperature regimes affect DNA methylation and gene expression. Long-term effects were detected by sampling fish several weeks after the end of differential treatments, and offspring from the different exposure groups was also sampled. Many overlapping genes were found between the different exposure groups and generations, coupled with genes associated with differential CpG methylation levels. Genes involved in muscle fibre development, general metabolic processes and formation of deformities were significantly affected, and genes relevant for intergenerational transfer of epigenetic marks were also detected. We believe the use of environmental cues can be a useful strategy for improving the production of Atlantic cod.

## Introduction

Atlantic cod is an abundant species in the North Atlantic Ocean and has contributed to the prosperity of several nations in this region. The decline in cod stocks in the late 1970s and the 1990s, particularly in Norway [1], has spurred interest in cod farming [2]. The first cod farming initiatives collapsed in less than 10 years due to cod stock recovery, global economic crisis, and unsolved biological issues such as early maturation, poor growth, deformities, and diseases – mainly furunculosis [3]. However, Norway’s National Cod Breeding Program (NCBP) has continued to produce juveniles for research activities. In 2022, NCBP produced the sixth generation of cod, and most of the main biological problems have been addressed through selective breeding for growth and disease resistance, improvements in larval and juvenile rearing protocols, feed nutrition, and husbandry practices, though a relatively high frequency of larval deformities is still reported [2]. Potential improvements exist within the environment Atlantic cod is exposed to, especially during early life stages. The biotic and abiotic environments during embryogenesis as well as nutrition during early larval stages have been shown to influence the later life stages in various organisms, including teleost fish [4]. These changes are largely driven by modifications in the epigenome resulting from its interaction with the environment [5].

Epigenetic regulation of gene expression has been investigated in great detail, in both model- and non-model organisms [6]. The most important mechanisms seem to be methylation of cytosine residues in CpG islands, histone modifications, and non-coding RNAs, such as microRNAs (miRNAs) [7]. Some of the epigenetic mechanisms are highly dynamic [8], whereas others are believed to survive even meiotic cell division [9]. In teleosts, most of the epigenetics research has been on zebrafish (Danio rerio) [10], though recent progress has been made on species relevant for aquaculture [11]. Manipulation of the epigenome through environmental cues, such as temperature, oxygen, and handling stress, can have long-term benefits that the aquaculture industry might be able to harness [12]. Embryonic temperature has been shown to influence muscle development and growth through epigenetic regulation of myogenic-regulating factors in several teleost species, including Atlantic salmon and Senegalese sole [13,14]. Atlantic salmon exposed to chronic low dissolved oxygen levels (~60% hypoxia) during early showed stimulation of several immune genes, as well as a reduction in their down-regulation commonly observed during smoltification [15]. In juvenile rainbow trout, glucose metabolism and growth performance were found to be affected by embryonic hypoxia exposure alone, or combined with a high carbohydrate diet during early life [16,17]. Moreover, epigenetic marks have been associated with disease resistance [18] and adaptation to climate changes [19]. In Atlantic cod, several studies have also linked epigenetic regulation with phenotypic features that could be of interest for the cod farming industry, including photoperiod regimes, believed to be tightly linked with growth [20,21].

Intergenerational transmission of DNA methylation as response to environmental cues has previously been observed in Atlantic salmon and other non-model fish species [22,23], and quantitative studies have suggested that DNA methylation is linked with both long-term changes in temperature and acute thermal stress in Atlantic cod embryos [24]. The aim of this study was to explore the long-term effects of different early life-stage temperature regimes on farmed Atlantic cod. Rearing conditions previously shown to be relevant for juvenile cod production such as temperature and feeding protocols were used [25,26] and persistent changes in the transcriptome and the methylome, as well as deformities, growth rates, and mortality rates, were recorded. In addition, the exposed animal’s offspring were kept under identical conditions and studied in order to investigate any intergenerational effects. RNA-Seq in combination with a complete annotation of the Atlantic cod transcriptome was used to get a comprehensive view of the affected pathways, and high coverage and quantitative DNA methylation data from CpG islands were obtained using reduced representation bisulphite sequencing (RRBS).

## Material and methods

### Broodstock

Experiments were carried out in 2013 (F0) and 2017 (F1) using second- and third-generation broodstock from the National Cod Breeding Program (NCBP). The broodstock fish were transferred to NCBP sea cages in Røsnes in Troms county (69°48’12.7“N 18°38’21.5“E) as 10-month-old juveniles and kept in the sea for 2 years. One month prior to spawning in 2013 and in 2017, mature adults were transferred to 25 m^3^ tanks at the Center for Marine Aquaculture in Kraknes (69°45’46.6“N 19°2’47.6“E). The tanks were supplied with ambient seawater at 4°C during spawning.

### Egg incubation and treatments

**Figure 1.**
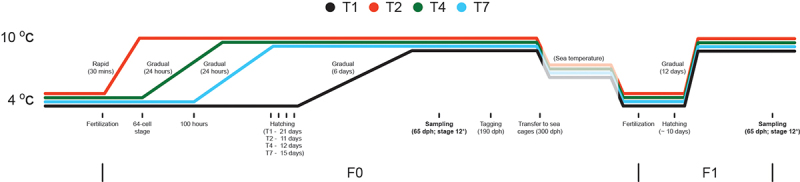
Sampling scheme. Males and females from mixed families were stripped and fertilized egg produced. For the T1 treatment the temperature was kept at 4°C until hatching, whereas the other groups went through gradual temperature increases until 10°C was reached. After tagging, fish were transferred to a common pen and offspring (F1) were produced by internally crossing the different treatment groups (no differential treatment prior to sampling). * - see vonHerbing et al. [28] or main text for details.

Hand-stripped eggs and sperm were obtained from eight females and four males (mixed families) in both generations. Eggs were externally fertilized using standard protocols [27]. The incubators were connected to a flowthrough system under the following conditions: continuous light at ~600 lux (measured at the top of the incubator), 34 ppt salinity, and UV-treated water. There were four temperature treatment groups in F0 (T1, T2, T4, and T7; Figure 1). For the T1 treatment, the water temperature was kept constant at 4°C until hatching. Newly hatched larvae were transferred to rearing tanks at 4°C, and 5–10 dph (days post hatching), the temperature was gradually increased to 10°C. For T2, the temperature was gradually increased over 30 min to 10°C directly after fertilization. For the T4 and T7 treatments, the temperature was gradually increased from 4°C to 10°C in 24 h after blastopore closure (64-cell stage) and late somitogenesis (100 h), respectively. After the temperature increase, T2, T4, and T7 embryos were kept at 10°C until hatching. For the F1 generation, eggs from all four treatment groups were treated similarly to the F0 T1 cohort and were not exposed to 10°C prior to hatching (Figure 1).

### Larval and juvenile rearing

At 2 dph, larvae were transferred to circular fibre glass rearing tanks (190 L) at a density of 150 larvae per litre. Water flow was gradually increased from 1.1 L min^−1^ at 2 dph to 3.3 L min^−1^ at 56 dph. The larvae were fed following the standard feeding protocol [25]. Briefly, microalgae (*Nannochloropsis* sp., Reed Mariculture, Campbell, USA) were added to the tank twice a day during the first 10 days. The larvae were fed with nutrient-enriched rotifers (2–29 dph) and Artemia (25–55 dph) and later weaned on to dry feed (44–56 dph). The above feeding regime changes were delayed by 2–3 weeks for the T1 group because of slower development due to a prolonged period of lower temperatures during early ontogeny. The number of fish kept in each tank was reduced to a maximum of 300 fish at 85–87 dph to minimize density-related effects on growth [29]. At 190 dph (~20 g), F0 fish were tagged intraperitoneally using Passive Integrated Transponder (PIT, Sokymat, Switzerland) tags. Survival rates were recorded, and the fish were visually inspected for the following skeletal/vertebral abnormalities: head deformity (stargazer; an axial deviation, V-shaped curvature in the neck), lower jaw deformity (shorter and/or ventrally bent lower jaw leaving an opened mouth), upper jaw deformity (mouth terminates in an upward direction), short tail fusion (fusion of vertebra at or near the tail), opercular deformity (short opercula that is not fully covering the gills), scoliosis (axial deviation, S shaped vertebral curvature from a dorsal view, sideways bending of the spine), kyphosis (axial, inverted V-shaped deviation seen from a lateral view, a dorso-ventral bending of the spine), and lordosis (axial V-shaped deviation seen from a lateral view, a dorso-ventral bending of the spine) [30–33]. Fish were transferred to sea cages (300 dph). For the F1 generation, offspring larvae of all parental treatment groups were treated similarly to the F0 T1 cohort.

### Isolation of DNA and RNA

DNA and RNA were isolated from the tail muscle tissue at larval stage 12 (~65 dph; end metamorphosis; approximately 15 mm body length [28]) for both the F0 and the F1 fish. Six biological replicates were collected, and nucleic acids were purified using the AllPrep DNA/RNA/miRNA Universal Kit (QIAGEN Nordic, Oslo, Norway) according to the manufacturer’s protocol. RNA/DNA quantity and integrity were analysed using a Nanodrop 2000 (Thermo Fisher Scientific, Waltham, MA, USA) and a Bioanalyzer 2100 (Agilent, Santa Clara, CA, USA).

### RNA-Seq

RNA-Seq library preparation and single-end sequencing (100 bp) were performed at The Norwegian Sequencing Centre using the TruSeq Stranded mRNA kit (Illumina, San Diego, CA, USA). Raw reads were trimmed and quality checked using Trimmomatic (version 0.39) [34] with recommended settings for single-end Illumina data. Reads were mapped to the *G. morhua* genome (assembly gadMor3.0) [35] using the STAR aligner (version 2.7.10b) [36] with default parameters. Differentially expressed genes (DEGs) were identified using the R wrapper SARTOOLS (version 1.8.1) [37] and DESeq2 (version 3.38.4) [38] with default settings (FDR cut-off 0.05).

### Reduced representation bisulphite sequencing (RRBS)

RRBS libraries were prepared using the Ovation RRBS Methyl-Seq kit (Tecan, Männedorf, Switzerland) following the manufacturer’s protocol. 100 bp single-end sequencing was done using Illumina (Illumina) chemistry at the Norwegian Sequencing Centre. Raw RRBS reads were adaptor clipped and quality trimmed using Trimmomatic [34] before they were mapped to the *G. morhua* genome (gadMor3.0) using the Bismark Bisulfite Mapper [39]. Individual cytosine positions were analysed using the Bismark module coverage2cytosine, and the report files were used as input for the R-package DMRcaller (version 1.28.0) [40]. The following parameters were used to identify differentially methylated regions (DMRs): method = noise_filter, window size = 100, kernel function = triangular, test = score, *p*-value threshold = 0.01, minimum cytosine count = 4, minimum proportion difference = 10%, minimum gap = 0, minimal size = 50 and minimal reads per cytosine = 4. For all the regions reported by DMRcaller as being differentially methylated, a region covering this locus and 2000 bp flanking sequence (upstream and downstream) was extracted from the *G. morhua* genome and used in Megablast searches [41] against the gadMor3.0 cod transcriptome (word size 28). The (highest scoring) transcript/gene with a Megablast hit was recorded as being differentially methylated.

### Cod transcriptome annotation and pathway analyses

The entire cod transcriptome (assembly gadMor3.0) [35] was annotated using InterProScan (version 5.51–85.0) [42] and the -goterms option. Gene Ontology (GO) terms [43,44] were extracted from the InterProScan output, and hypergeometric testing for enriched GO terms in sets of genes was done using GOStats (version 3.16) [45].

Smaller sets of genes were annotated using the software KofamKOALA [46] with default settings. KEGG Orthologs (KOs) were extracted from the KofamKOALA output and used in KEGG Mapper Reconstruct analyses [47] and the KEGG Pathway Database (Release 105.0).

## Results

### Fish phenotypes

**Figure 2.**
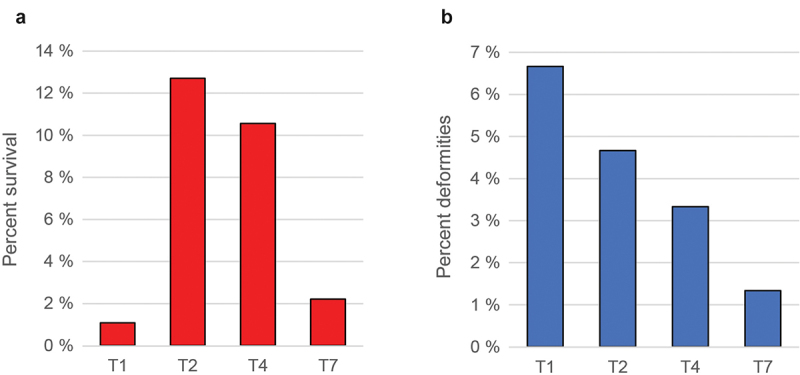
Survival rates (a) and skeletal deformities (b) (~190 dph).

**Figure 3.**
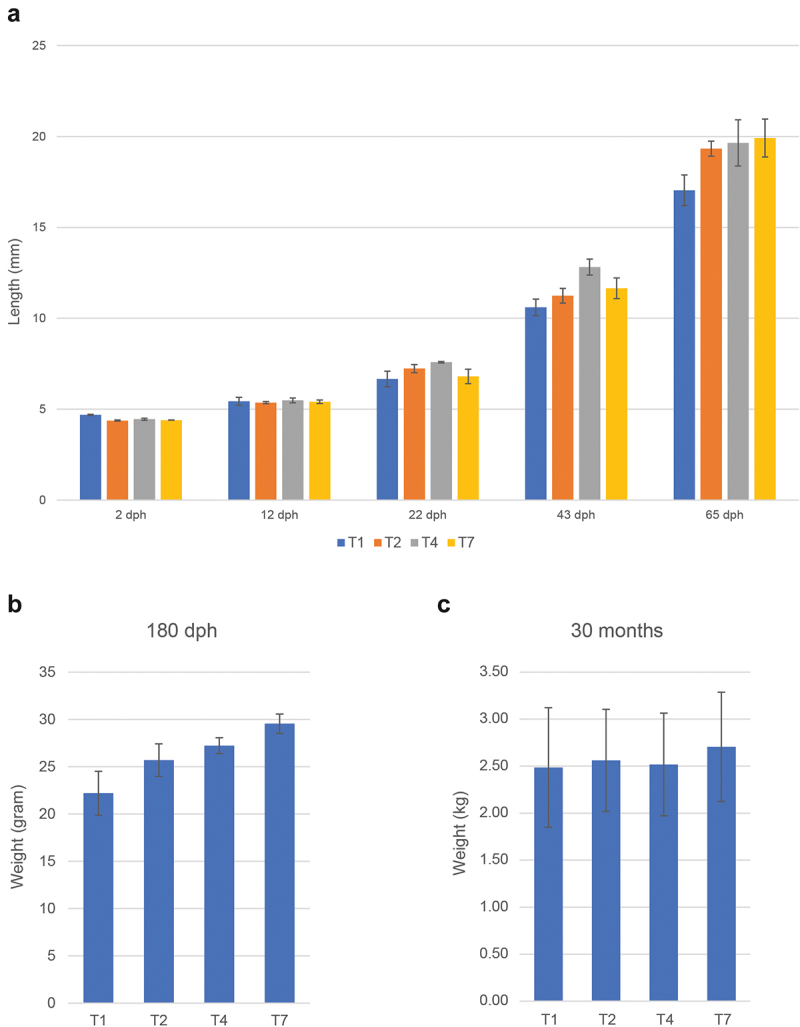
Size of fish at various timepoints for the different treatments. (a) Length (mm), average values for 30 individuals with standard deviation. (b) and (c) similar data (weight gram/kg) 180 dph and 30 months post hatch (slaughter).

The different temperature regimes affected the length of time between fertilization and hatching, with the T2 group hatching first (11 days after fertilization) and the T1 group hatching last (21 days after fertilization) (Figure 1), consistent with what has been reported previously for Atlantic cod [48]. The survival rate of the four temperature groups 80 dph varied more than 10-fold (Figure 2a), with the T1 and T7 groups showing a very low survival rate (1.1% and 2.2%, respectively), whereas survival rates for the T2 and T3 cohorts were much higher (Figure 2a). Differences in body size could be observed at 180 dph, but the different treatment groups were nearly identical at slaughter weight (Figure 3).

The temperature treatment T1 seemed to be associated with a relatively high degree of deformities, followed by T2, T4, and T7 (Figure 2b), and most of the common deformities overall were shorter and/or ventrally bent lower jaw (Supplementary table S1).

### RNA-Seq and RRBS

The average number of reads that could be mapped per sample for the RNA-Seq data was 34 393 945 (median: 34 298 536), and a total of 26 317 genes had at least one mapped read. The treatment associated with the highest degree of survival (T2) was defined as a control group in the DEGs analysis.

**Figure 4.**
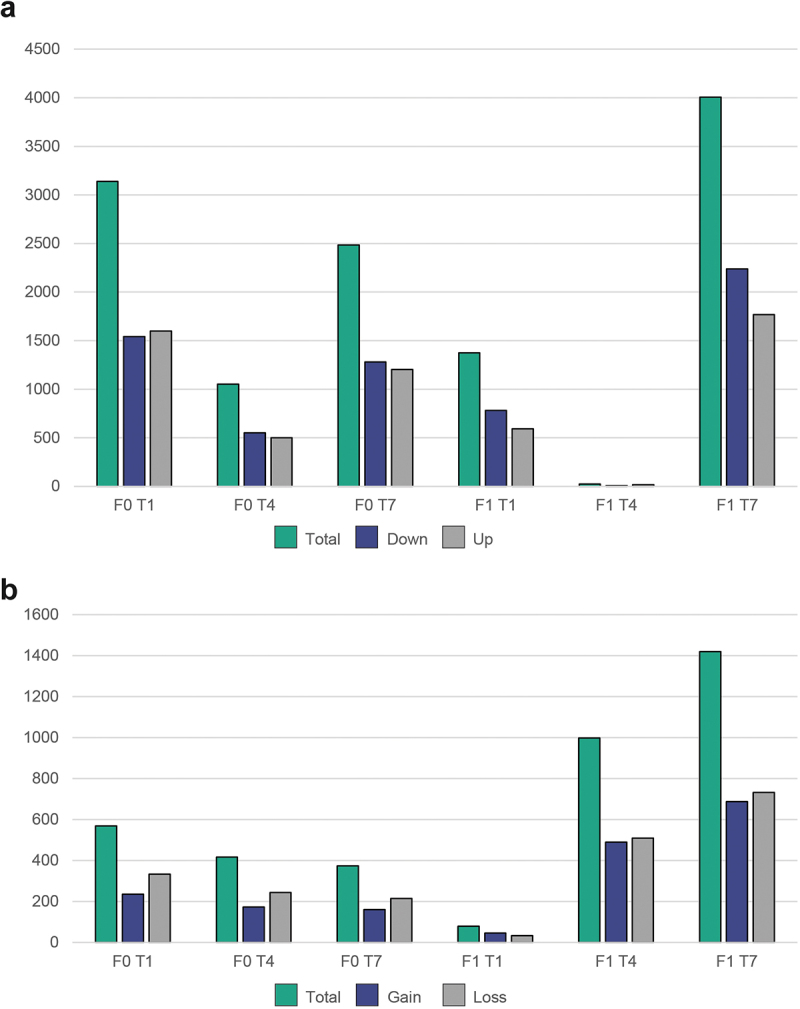
Number of differentially expressed genes and differentially methylated loci for different treatment groups using (T2 treatment as reference). (a) Number of DEGs as scored by DESeq2 (FDR cut-off 0.05) and (b) total number of differentially methylated loci (DMRcaller, adjusted p-value <0,01).

The number of DEGs seen for the different treatments ranged from 1053 (T4) to 3193 (T1) for the F0 fish and from 24 (T4) to 4006 (T7) for the F1 fish (Figure 4a; Supplementary table S2). The raw RNA-Seq data were submitted to NCBI’s Short Read Archive (SRA) database as part of BioProject accession PRJNA934710.

In the RRBS data (F0 and F1), an average of 2 081 373 (median: 2 216 211) CpG positions had >10X coverage per sample. These positions were used in the DMRcaller analyses after excluding two samples with low coverage. The T2 samples were again used as a reference, and a total of 3854 DMRs were detected by DMRcaller. 3037 of these had a matching gene <2000 bp upstream/downstream (Supplementary table S2). The raw RRBS data were submitted to the SRA database as part of BioProject accession PRJNA934710.

A large number of DMRs could be observed for all four treatments (Figure 4b; Supplementary table S2). The high number of DMRs seen in the T4 and T7 fish in the F1 generation indicates that the temperature modulation performed in the larval stage of the F0 fish had a substantial impact on the epigenome of the offspring for these treatment groups, whereas the T1 treatment seems to have less effect (Figure 4b). Looking at the DMR-associated genes with the lowest adjusted *p*-values, some genes appeared to be reoccurring targets for robust differential methylation (Table 1).

**Table 1.** Top three loci with lowest-adjusted *p*-values found by DMRcaller for different generations/treatments.

Generation/treatment	Contig (gadMor3.0)	Start	End	Length	# of cytosines	Adjusted *p*-value	Gain/Loss	Gene	Annotation (KEGG)
F0 T1	NC_044060.1	15903446	15903751	306	30	0	Gain	*pde1b*	Calcium/calmodulin-dependent 3‘,5’-cyclic nucleotide phosphodiesterase 1B
F0 T1	NC_044063.1	33396967	33397250	284	25	0	Loss	LOC115539306	lncRNA
F0 T1	NW_021963984.1	7077	7436	360	31	8.49E–275	Loss		
F0 T4	NC_044051.1	18847155	18847523	369	36	0	Gain	LOC115541396	Neuropeptide receptor-related G-protein coupled receptor
F0 T4	NC_044060.1	15903446	15903751	306	30	0	Gain	*pde1b*	Calcium/calmodulin-dependent 3‘,5’-cyclic nucleotide phosphodiesterase 1B
F0 T4	NC_044055.1	10803222	10803421	200	27	5.26E–227	Gain	LOC115548542	Glycoprotein-N-acetylgalactosamine 3-beta-galactosyltransferase 1
F0 T7	NC_044051.1	537465	537685	221	32	0	Loss		
F0 T7	NC_044051.1	19703878	19704200	323	45	0	Loss	*dipk1b*	Protein-kinase domain of FAM69
F0 T7	NC_044069.1	18818387	18818713	327	92	0	Gain	*col28a1*	Collagen, type XXVII, alpha 1B-related
F1 T1	NC_044059.1	912136	912290	155	6	1.41E–154	Gain	LOC115555977	Small GTPase superfamily, Ras-type
F1 T1	NW_021964013.1	49440	49615	176	9	3.20E–139	Gain		
F1 T1	NC_044063.1	29127493	29127687	195	13	1.19E–134	Gain	LOC115533202	DNA/RNA polymerase superfamily
F1 T4	NC_044048.1	19978664	19978972	309	31	0	Loss	*tti1*	Tel2 interacting protein 1 tti1 family member
F1 T4	NC_044051.1	537465	537650	186	17	0	Gain		
F1 T4	NC_044052.1	17623621	17623967	347	27	0	Loss		
F1 T7	NC_044048.1	890808	891313	506	40	0	Loss	LOC115538267	Sulfate permease family
F1 T7	NC_044048.1	7808540	7808886	347	33	0	Gain	LOC115552102	Glycoprotein-N-acetylgalactosamine 3-beta-galactosyltransferase 1-A
F1 T7	NC_044048.1	19978673	19978953	281	28	0	Loss	*tti1*	Tel2 interacting protein 1 tti1 family member

**Figure 5.**
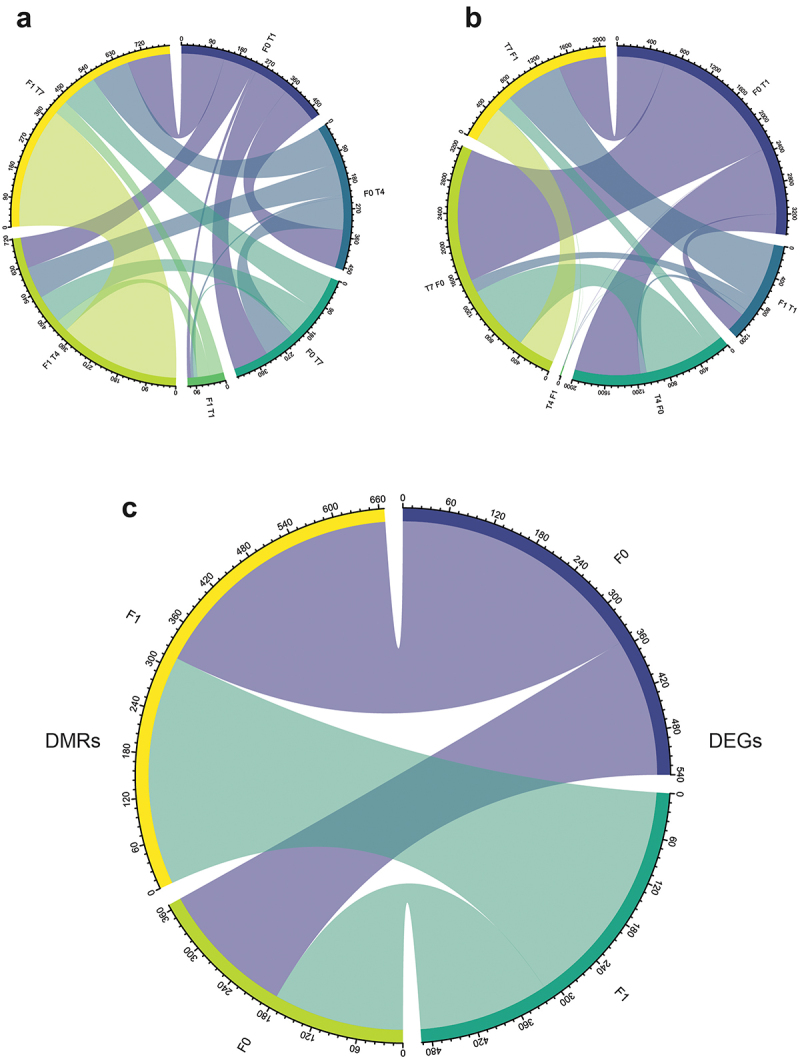
Chord diagram showing overlapping genes. (a) Overlapping DEGs between different treatments/generations. (b) Overlapping DMR-associated genes. (c) General overlap between F0 and F1 DEGs and DMR-associated genes.

Overall, all cohorts of fish showed overlap for both the DMR-associated genes and the DEGs (Figure 5a/b). In general, the F0 and F1 fish also displayed large overlaps between the genes reported as DMRs and DEGs (Figure 5c), indicating that similar mechanisms are evoked in several of the temperature treatment regimes. Within the F0 and F1 generations, some of the treatments resulted in genes being both differentially expressed and associated with a DMR region, but no annotated genes possessed this dual status in both F0 and F1 fish (Supplementary table S3). The four pathways with the highest number of mapped genes from the annotation and KEGG mapping of the DEG/DMR genes were ‘Pathways in cancer’, ‘MicroRNAs in cancer’, ‘Signaling pathways regulating pluripotency of stem cells’ and ‘Axon guidance’ (Supplementary table S3).

**Figure 6.**
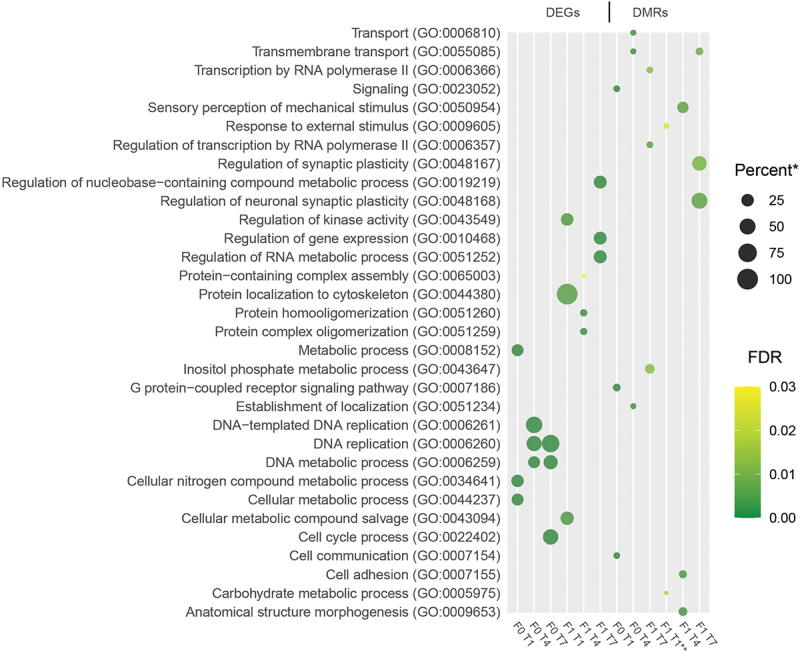
Enriched GO-terms. Dot plot showing the three GO-terms with the lowest adjusted *p*-values (filtered to include only terms with at least two representative genes in list). * - Percent of genes in GO-term bin detected (all genes with at least one mapped read in all samples were used as a reference transcriptome). ** - Only two significant terms found (adjusted p-value <0.05).

The hypergeometric testing for overrepresentation of ‘Biological Process’ GO terms showed small overlaps in terms enriched for the different sets of DEGs and DMR-associated genes (Figure 6). Most of the pathways identified were higher-order processes, and only a small number of terms were found in more than one gene set when looking at the enriched GO terms with the lowest adjusted *p*-values.

## Discussion

The majority of the GO-terms that showed significant enrichment for the different gene-sets appeared to be higher-order terms associated with general biological processes (Figure 6). There was no overlap in enriched terms for DEGs and DMRs, but several terms appeared in multiple treatment groups (transmembrane transport and DNA replication/metabolic processes; Figure 6). It is quite possible that the effects of the treatments are generic in nature and that primarily quantitative changes in metabolic processes can be recorded when analysing DEGs and DMRs using an unbiased approach, though individual genes might point towards more specific pathways being affected. Looking at the loci with the lowest *p*-values in the DNA-methylation analysis, two genes appear twice: *pde1b* and *tti1* (Table 1). Members of the Pde1 family have previously been linked with cysts and deformities in zebrafish [49], and Tti1 is an integral part of the mTOR pathway, a major regulator of autophagy, translation, and cell growth [50]. Multiple of our cohorts also showed differential expression and methylation of DNA (cytosine-5)-methyltransferases, such as *dnmt1* and *dnmt3a* (Supplementary table S2), consistent with previous studies on epigenetics in Atlantic cod [20].

The E2F transcription factor 3 (*e2f3*) was the only gene found to be differentially expressed as well as differentially methylated in the F0 T1, T4, and T7 groups (Supplementary table S3). *e2f3*-expression was downregulated and showed an overall loss of methylation for the F0 cohorts (Supplementary table S2). Additionally, *e2f1*, *e2f4*, *e2f7* and *e2f8* were upregulated, although no differences in DNA methylation were observed (Supplementary table S2). The E2f family has been found to be expressed in most cell types and play a vital role in processes controlling cell cycle, proliferation, and differentiation, as well as apoptosis [51,52]. *In vitro*, E2f3 has been shown to promote the proliferation and differentiation of myocytes [53], and the downregulation of E2f3 results in a suppression of the myogenic transcription factor myogenin and myosin heavy chain 1, which are involved in the differentiation of muscle cells [54]. Our data show that increasing the incubation temperature directly after fertilization resulted in persistent changes in E2f3 expression and methylation pattern, still present in muscle tissue of Atlantic cod after metamorphosis (developmental stage 12). This suggests that muscle differentiation in the T2 group is affected, which may influence muscle development. Although the muscle cellularity of the fish was not analysed in the present study, elevated embryonic temperature of 10°C in Atlantic cod has previously been found to increase the number of smaller myofibers in larvae compared with those reared at 4°C or 7°C [55]. Furthermore, this underscores the relevance of temperature change timing during embryonic development and that changes in the environment during the early stages appear to have more pronounced effects.

Our samples were collected for an extended period of time after exposure to temperature manipulations, and offspring from the different treatment groups were sampled after having been held under identical conditions for several weeks. In order to reduce differences in DNA methylation and RNA expression caused by factors other than the temperature treatments, both F0 and F1 fish were sampled at the same developmental stage [28], it is, however, possible that the delayed feeding regime used in the F0 T1 May also have affected our results. Also, it cannot be excluded that the data from our F1 fish are the result of germline cells in F0 fish being epigenetically altered by the original temperature cues, as our experimental design falls within the field of ‘intergenerational’ studies [56], as opposed to true ‘transgenerational’ studies. In zebrafish, the level of DNA methylation is reduced during the first cell division after fertilization, and reaches a minimum at the 64-cell stage [57]. If this mechanism is conserved within the teleosts, replicative transmission of epigenetic imprinting might not be sufficient to explain the different DNA methylation patterns seen in our F1 fish. Looking for KEGG orthologs enriched in the set of genes that show both differential expression and are in the genomic vicinity of a differentially methylated locus, the best hits included pathways involved in stem cell maintenance and miRNA processing (Suplementary Table S3). We believe these findings might offer important clues as to how epigenetic marks may survive both mitosis and meiosis in Atlantic cod. miRNAs are known to be carriers of epigenetic information, and the epigenetic (re)programming of stem cells seems in many regards to be homologous to the different stages of embryonic development [58]. Long-term changes in miRNAs expression have previously been linked with temperature shifts in Atlantic cod embryos/larvae [59], supporting the idea that such manipulations have epigenetic consequences.

The different treatment groups showed different trends when comparing the number of genomic regions that are differentially methylated in F0 *versus* F1 fish. Treatment T1 seemed to lead to a decrease in the number of F1 DMRs, whereas the two other treatments showed an increase (Figure 4B). It is common for epigenetic phenomenon to get ‘diluted’ in later generations [60], and even in the best documented cases of transgenerational inheritance in teleosts, the effect is lost after a few generations [61]. The increase seen in the F1 T4 and T7 cohorts could be the result of germ cells in the F0 fish being exposed to different temperature regimes. Since the whole fish were exposed in the F0 generation, it is likely that the cells representing different tissue types responded differently. In sampling caudal muscle tissue in F0 fish, only the effects manifested in this tissue after multiple rounds of mitotic division would be observed. If the germ cells for the F1 fish were already established when the F0 fish were exposed, the epigenetic changes in this cell population could lead to a broader effect, where the whole F1 animal would carry the marks from a genetic imprinting of the F0 germ cells. This would effectively lead to a higher number of DMRs in F1 fish being detected, as a consistent pattern of differential methylation could be present throughout all tissue types. The level of germ cell differentiation in our F0 fish was not investigated, but a model where the DMRs observed in F0 fish are the results of somatic cells being exposed to temperature modulations and the DMRs observed in F1 fish are the result of F0 germline cells being epigenetically altered, could also explain the limited overlap in specific genes when comparing F0 and F1 DMR-associated genes within the different treatment groups.

There did not seem to be a strong functional connection between DEGs and DMRs, though some overlap was observed (Figure 5, Supplementary table S3). An (inverse) correlation between expression level and methylation level was not significant (data now shown) and the number of DMRs and DEGs for the different generations/treatments was inconsistent (Figure 4). Assuming that many of the affected genes behave according to the epigenetic priming model [62–65], long-term epigenetic marks may have been laid down as a response to environmental cues during early life stages, but the manifestation on a gene-expression level would have been dependent on tissue types sampled and perhaps also the presence of environmental triggers in connection with the sampling time point. We also note that many genes involved in histone modifications are listed as DEGs and/or DMRs, including multiple histone acetyltransferases, deacetylases, and arginine demethylases (Supplementary table S2), opening up the possibility that such mechanisms might also be relevant for the epigenetic control of gene expression in our experimental setup.

The protocol for rearing cod larvae is under constant development, and different temperature treatments (as well as other parameters) are being tested to ensure efficient production and fish welfare. The effect of temperature changes on egg viability and larval quality has been investigated previously [48], and our findings on deformities are generally consistent with what has been published previously. High variability in mortality rates is a field of active research, but we do not believe this phenomenon introduces a selection bias relevant for our study, as most of these events show very high mortality rates and are limited to specific tanks and/or pens. Increases, or changes, in the bacterial flora have been suggested as a possible aetiology, but the exact mechanisms remain to be elucidated.

In summary, we believe our data support the idea that Atlantic cod shows long-term changes in transcriptional activity after temperature modulation at the larval stage. Some of these changes might be linked with changes in CpG island cytosine methylation and involve genes relevant for growth/general metabolic processes, formation of deformities, and muscle fibre composition. Atlantic cod also appears to be capable of intergenerational transfer of changes in transcriptional activity/DNA methylation. Taken together, these data indicate that environmental priming of juvenile Atlantic cod can have phenotypic effects that could complement traditional breeding programmes when trying to establish a sustainable cod farming industry.

## Supplementary Material

Supplemental Material

## Data Availability

All molecular data relevant for this publication have been submitted to the National Center for Biotechnology Information’s Short Read Archive as BioProject PRJNA934710.
