# Supplementary material for: Intergenerational effects of early life-stage temperature modulation on gene expression and DNA methylation in Atlantic cod (Gadus morhua)
Source: Epigenetics. 2023 Jul 27;18(1):2237759. doi: 10.1080/15592294.2023.2237759 (PMC10376914; doi:10.1080/15592294.2023.2237759)
Supplement: Supplemental Material [file KEPI_A_2237759_SM3549.zip › Supplementary files/Supplementary_table_1.docx]

Supplementary table 1. Deformities detected in F0 fish (190 dph).

| Treatment | Head deformity | Lordosis | Scoliosis | Lower jaw | Upper jaw | Short tail fusion | Operculum deformity | Kyphosis |
| --- | --- | --- | --- | --- | --- | --- | --- | --- |
| **T7** | 0 | 0 | 0 | 2 | 0 | 0 | 0 | 0 |
| **T1** | 1 | 0 | 0 | 9 | 0 | 0 | 0 | 0 |
| **T2** | 2 | 0 | 0 | 4 | 1 | 0 | 0 | 0 |
| **T4** | 3 | 0 | 0 | 1 | 1 | 0 | 0 | 0 |
