# Supplementary material for: Intergenerational effects of early life-stage temperature modulation on gene expression and DNA methylation in Atlantic cod (Gadus morhua)
Source: Epigenetics. 2023 Jul 27;18(1):2237759. doi: 10.1080/15592294.2023.2237759 (PMC10376914; doi:10.1080/15592294.2023.2237759)
Supplement: Supplemental Material [file KEPI_A_2237759_SM3549.zip › Supplementary files/Supplementary_table_2.pdf]

Supplementary table 2. Differentially expressed and differentially methylated genes.

	Differentially expressed genes (DESeq2; FDR < 0.05)						Differentially methylated gene loci (DMRCaller; FDR < 0.01)							
**Gene (gadMor3.0)**	F0 T1	F0 T4	F0 T7	F1 T1	F1 T4	F1 T7	F0 T1	F0 T4	F0 T7	F1 T1	F1 T4	F1 T7	**Kegg-term**	**Annotation**
abat	0.41													4-aminobutyrate aminotransferase / (S)-3-amino-2-methylpropionate transaminase
abca3	-0.56	-0.36		0.57										ATP-binding cassette, subfamily A (ABC1), member 3
abcb10	0.54			-0.46		-0.46							K05657	ATP-binding cassette, subfamily B (MDR/TAP), member 10
abcb6						-0.50						Gain	K05661	ATP-binding cassette, subfamily B (MDR/TAP), member 6
abcb9	0.60					0.54							K05656	ATP-binding cassette, subfamily B (MDR/TAP), member 9
abcc3						-0.39								ATP-binding cassette, subfamily C (CFTR/MRP), member 1 , ATP-binding cassette, subfamily C (CFTR/MRP), member 3
abcc5	-0.44											Gain	K05668	ATP-binding cassette, subfamily C (CFTR/MRP), member 5
abcd3	0.30					0.29							K05677	ATP-binding cassette, subfamily D (ALD), member 3
abcf1	0.36												K06158, K06184	ATP-binding cassette, subfamily F, member 1, ATP-binding cassette, subfamily F, member 3
abcf3						0.33								ATP-binding cassette, subfamily F, member 3
abcg4								Gain						ATP-binding cassette, subfamily G (WHITE), member 1, ATP-binding cassette, subfamily G (WHITE), member 4
abhd10						0.52							K13702	Abhydrolase domain-containing protein 10
abhd11	0.61											Loss	K13703	Abhydrolase domain-containing protein 11
abhd14a						-0.43							K13706	Abhydrolase domain-containing protein 14
abhd14b	-0.43												K13706	Abhydrolase domain-containing protein 14
abhd16a						0.61								Abhydrolase domain-containing protein 16A
abhd17b				-0.44		-0.83							K01076	Abhydrolase domain-containing protein 17
abhd6				-0.39										Abhydrolase domain-containing protein 6
abi1						-0.50								Abl interactor 1
abitram	0.31			-0.33										
abraxas1				0.41		0.42							K20774	BRCA1-A complex subunit Abraxas
abraxas2				0.26									K20799	BRISC complex subunit Abro1
abtb1	-2.08		-1.29											Ankyrin repeat and BTB/POZ domain-containing protein 1
acacb			-0.66										K01946, K11262	Acetyl-coa carboxylase / biotin carboxylase 1 , acetyl-coa carboxylase / biotin carboxylase 2
acad8						-0.59							K00249, K11538	Acyl-coa dehydrogenase , isobutyryl-coa dehydrogenase
acadm						-0.41								Acyl-coa dehydrogenase

acadsb						-0.53							K00249, K09478	Acyl-coa dehydrogenase , short-chain 2-methylacyl-coa dehydrogenase
acadvl				-0.29		-0.38								Very long chain acyl-coa dehydrogenase
acan												Gain		Aggrecan 1
acbd3	0.32												K23935	Golgi resident protein GCP60
acbd5	-0.63		-0.66			0.61								
accs		-0.32												
acer2				-0.45		-0.96							K01441	Alkaline ceramidase
acer3			-0.47	-0.47		-0.83								Dihydroceramidase
ackr4	-0.49													
acly						-0.49							K01648, K02907	ATP citrate (pro-S)-lyase , large subunit ribosomal protein L30
aco1			0.33										K01681	Aconitate hydratase
acod1				-0.43		-0.85							K17724	Aconitate decarboxylase
acot11						-0.59								Acetyl-coa hydrolase , acyl-coa thioesteraes 11
acp2	-0.52													Lysosomal acid phosphatase
acp5							Gain							Tartrate-resistant acid phosphatase type 5
acp7	-0.39		-0.46										K22390	Acid phosphatase type 7
acsm3						-1.24								Medium-chain acyl-coa synthetase
acss3						0.70							K01908	Propionyl-coa synthetase
actg1			0.31											Actin beta/gamma 1, actin, other eukaryote
actl6a	0.67		0.52										K11340, K11652	Actin-like protein 6A, actin-like protein 6B
actn4			0.41										K05699	Actinin alpha 1/4
actr10		0.33											K16576	Actin-related protein 10
actr2						0.46								Actin-related protein 2
actr6	0.39			-0.53		-0.47							K11662	Actin-related protein 6
acvr1				0.32										Activin receptor type-1
acvr1c											Gain	Gain	K13568	Activin receptor type-1C
ada2	-0.73	-0.39	-0.67											Adenosine deaminase CECR1
adam10											Loss		K06704	Disintegrin and metalloproteinase domain-containing protein 10
adam12								Loss					K06835	Disintegrin and metalloproteinase domain-containing protein 12
adam15	-0.46										Loss	Loss		
adam9						-0.33								Disintegrin and metalloproteinase domain-containing protein 9

adamts1						-0.59								A disintegrin and metalloproteinase with thrombospondin motifs 1
adamts13						-0.53							K08627	A disintegrin and metalloproteinase with thrombospondin motifs 13
adamts18	0.73													A disintegrin and metalloproteinase with thrombospondin motifs 18
adamts5				-0.37		-0.31								A disintegrin and metalloproteinase with thrombospondin motifs 5
adamts6						0.71								A disintegrin and metalloproteinase with thrombospondin motifs 6
adamts8						-0.81							K08623	A disintegrin and metalloproteinase with thrombospondin motifs 8
adamts9						-0.48							K08624	A disintegrin and metalloproteinase with thrombospondin motifs 9
adamtsl1			-0.48								Gain	Gain	K24429	ADAMTS-like protein 1/3
adamtsl2				-0.44		-1.08							K24430	ADAMTS-like protein 2
adar	0.54		0.41										K12968	Double-stranded RNA-specific adenosine deaminase
adck2	0.95		0.56											Aarf domain-containing kinase
add1						-0.49							K18622	Adducin
add3	-0.83		-0.65										K18622	Adducin
adgrb3											Gain	Gain		Adhesion G protein-coupled receptor B3
adgrg6						-1.16								Adhesion G-protein coupled receptor G6
adipor1						-0.53							K07297	Adiponectin receptor
adm						-1.01								Adrenomedullin
ado				-0.58		-1.23							K10712	Cysteamine dioxygenase
adora2a							Gain						K04266	Adenosine receptor a2a
adpgk						-0.41							K08074	ADP-dependent glucokinase
adsl												Loss		Adenylosuccinate lyase
adss2				-0.43		-0.54							K01939	Adenylosuccinate synthase
afap1												Gain	K18616	Actin filament associated protein 1
afap1l2						0.42								Actin filament associated protein 1-like
aff2											Gain + Loss	Gain + Loss		AF4/FMR2 family member 2
aff3						-0.87								
aff4						-0.52							K15185	AF4/FMR2 family member 4
afmid						-0.44								
agap1			0.39										K12491	Arf-GAP with gtpase, ANK repeat and PH domain-containing protein 1/3/4/5/6/9/11

agfg1	0.33		0.41										K15044	Arf-GAP domain and FG repeats-containing protein 1
aggf1	0.76		0.57			0.34								
agpat5	0.42	0.42												Lysophosphatidiate acyltransferase
agr2						0.81							K20356	Anterior gradient protein 2
agtpbp1	-0.47		-0.39			-0.52							K23435	Cytosolic carboxypeptidase protein 1
agtrap												Loss		
ahcyl2												Gain	K01251	Adenosylhomocysteinase
aida				-0.38		-0.83								
ak2						0.57								Adenylate kinase
ak5									Gain				K00939	Adenylate kinase
akap10						-0.81						Gain	K16526	A-kinase anchor protein 10
akap11	-0.32		-0.41											A-kinase anchor protein 11
akap12						0.71								A-kinase anchor protein 12
akip1				0.43										
akr1a1	0.48	0.35	0.68										K00002	Alcohol dehydrogenase (NADP+)
akr1d1	-0.59													3-oxo-5-beta-steroid 4-dehydrogenase
akt1												Loss		RAC serine/threonine-protein kinase
akt1s1	-0.82	-0.43	-0.83											Proline-rich AKT1 substrate 1
aktip						-0.47								
alad	0.41		0.30										K01698	Porphobilinogen synthase
aldh16a1						-0.57								
aldh18a1	0.56													Delta-1-pyrroline-5-carboxylate synthetase
aldh1a3						-0.81								Aldehyde dehydrogenase (NAD+) , retinal dehydrogenase
aldh1l2						0.58							K00289	Formyltetrahydrofolate dehydrogenase
aldh6a1	-0.63		-0.45										K00140	Malonate-semialdehyde dehydrogenase (acetylating) / methylmalonate-semialdehyde dehydrogenase
aldh9a1	-0.57													Aldehyde dehydrogenase family 9 member A1
aldoc						-0.59					Gain		K01623	Fructose-bisphosphate aldolase, class I
alg14	-0.83		-0.59			0.54							K07441	Beta-1,4-N-acetylglucosaminyltransferase
alg2	0.62	0.55	0.68			0.48								Alpha-1,3/alpha-1,6-mannosyltransferase
alg3						-0.46								Alpha-1,3-mannosyltransferase
alg5	0.42		0.38										K00729	Dolichyl-phosphate beta-glucosyltransferase
alg6	0.47			0.47		0.52								Alpha-1,3-glucosyltransferase

alg9	0.49		0.48											Alpha-1,2-mannosyltransferase
alkbh5							Loss						K10767	Mrna N6-methyladenine demethylase
alpk1				0.39								Loss		Alpha-kinase
alpk2	-0.58		-0.65									Loss		Alpha-kinase
alpk3	-0.75		-0.71	-0.42		-0.86							K08868	Alpha-kinase
alpl						0.81								Alkaline phosphatase
alx1	-1.71												K09334	ALX homeobox protein 1
ambra1			0.32											Activating molecule in BECN1-regulated autophagy protein 1
amd1	0.45	0.70	0.54											S-adenosylmethionine decarboxylase
amdhd2						1.03							K01443	N-acetylglucosamine-6-phosphate deacetylase
ampd1	-2.52												K01490	AMP deaminase
ampd3						0.52							K01490	AMP deaminase
amt						-0.63							K00605	Aminomethyltransferase
anapc1			0.56										K03348	Anaphase-promoting complex subunit 1
anapc16	-0.69	-0.32	-0.75										K25229	Anaphase-promoting complex subunit 16
anapc2	0.72		0.53											Anaphase-promoting complex subunit 2
anapc4			0.33										K03351	Anaphase-promoting complex subunit 4
anapc5			0.44			0.63								Anaphase-promoting complex subunit 5
anapc7						-0.39							K03354	Anaphase-promoting complex subunit 7
angel2			-0.31										K18729	Protein angel
angptl2				-0.62		-1.13								Angiopoietin-related protein 2
angptl4				-0.45		-1.07							K08767	Angiopoietin-like 4
angptl7						-0.83								Angiopoietin-related protein 7
ankfn1	0.36												K24478	Ankyrin repeat and fibronectin type-III domain-containing protein 1
ankfy1				-0.32		-0.37								Rabankyrin-5
ankib1	-0.30	-0.28	-0.47	0.31		0.35								Ankyrin repeat and IBR domain-containing protein 1
ankle1	0.81													Ankyrin repeat and LEM domain-containing protein 1
ankle2	-0.67	-0.43	-0.37	0.37										Ankyrin repeat and LEM domain-containing protein 2
ankmy2						0.46							K24633	Ankyrin repeat and MYND domain-containing protein 2
ankra2				-0.45										Ankyrin repeat family A protein 2, uncharacterized protein
ankrd1				-0.41										
ankrd10						-0.56								

ankrd11						0.40							K21436	Ankyrin repeat domain-containing protein 11/12
ankrd13a	0.30												K21437	Ankyrin repeat domain-containing protein 13
ankrd13b						0.78								Ankyrin repeat domain-containing protein 13
ankrd29			-0.45											
ankrd46						-0.70								
ankrd49	-0.56													Ankyrin repeat domain-containing protein 49
ankrd53						-0.69								Ankyrin repeat domain-containing protein 53
ankrd54						-0.43								Ankyrin repeat domain-containing protein 54
ankrd9	-2.26	-0.58	-2.54										K25208	Ankyrin repeat domain-containing protein 9
anks1a						-0.34								Ankyrin repeat and SAM domain-containing protein 1
ankzf1	0.55					0.34								Ankyrin repeat and zinc finger domain-containing protein 1
anln	0.46		0.76											Actin-binding protein anillin
ano4								Loss						Anoctamin-4
ano5						-0.42							K19480	Anoctamin-5
anp32b	0.54	0.34	0.49										K18647	Acidic leucine-rich nuclear phosphoprotein 32 family member B
antxr2						0.70								Anthrax toxin receptor
anxa1	-0.47												K17091	Annexin A1
ap1ar			0.34			-0.30								
ap1m1	0.51							Gain					K12393	AP-1 complex subunit mu
ap1s1			0.64										K12394	AP-1 complex subunit sigma 1/2
ap2s1			0.31											AP-2 complex subunit sigma-1
ap3s1											Gain			AP-3 complex subunit sigma
ap4m1	-0.66		-0.43											AP-4 complex subunit mu-1
apbb1						0.70								Amyloid beta A4 precursor protein-binding family B member 1
apbb3							Gain		Gain		Gain	Gain		
api5	0.42	0.28	0.28											
aplf	-1.11	-0.72	-0.92											Aprataxin and PNK-like factor
apmap						0.79							K21407	Adipocyte plasma membrane-associated protein
apoe	-1.09													Apolipoprotein E
apom						1.27							K25354	Apolipoprotein M
app						-0.31								Amyloid beta A4 protein
appl1	-0.55	-0.32	-0.41										K08733	DCC-interacting protein 13 alpha
aprt				-0.41		0.39								Adenine phosphoribosyltransferase

aptx						-0.53							K10863	Aprataxin
aqr			0.46											Intron-binding protein aquarius
araf	0.35		0.35										K08845	A-Raf proto-oncogene serine/threonine-protein kinase
arap1				0.52									K18439	Arf-GAP with Rho-GAP domain, ANK repeat and PH domain-containing protein 1
arcn1	0.36	0.37	0.47										K20471	Coatomer subunit delta
arel1				0.43										Apoptosis-resistant E3 ubiquitin protein ligase 1
arfgap1	0.40		0.28											ADP-ribosylation factor gtpase-activating protein 1
arfgef2			0.38									Loss		Brefeldin A-inhibited guanine nucleotide-exchange protein
arfgef3						0.71								Brefeldin A-inhibited guanine nucleotide-exchange protein 3
arfip1						-0.58							K20314	Arfaptin
arfrp1	-0.35												K07952	ADP-ribosylation factor related protein 1
arg2	-2.56	-0.95	-1.72			-0.46								Arginase
arglu1				0.32		0.28								Arginine and glutamate-rich protein 1
arhgap10						-0.40								Rho gtpase-activating protein 10
arhgap18	-0.52		-0.48			-0.49							K20639	Rho gtpase-activating protein 18/28/40
arhgap22	0.44												K20642	Rho gtpase-activating protein 22/24/25
arhgap24	-0.58		-0.53										K20642	Rho gtpase-activating protein 22/24/25
arhgap31						-0.63	Loss	Loss						Rho gtpase-activating protein 31
arhgap32						0.64								Rho gtpase-activating protein 32
arhgap39			-0.47			0.70								Rho gtpase-activating protein 39
arhgap9				0.55									K20634	Rho gtpase-activating protein 9
arhgef1				0.42									K12330	Rho guanine nucleotide exchange factor 1
arhgef10				0.37									K16727	Rho guanine nucleotide exchange factor 10
arhgef10l									Gain				K16727	Rho guanine nucleotide exchange factor 10
arhgef11	-0.29												K12331	Rho guanine nucleotide exchange factor 11
arhgef12						0.48							K07532	Rho guanine nucleotide exchange factor 12
arhgef19											Loss			Rho guanine nucleotide exchange factor 19
arhgef2						0.66							K12791	Rho guanine nucleotide exchange factor 2
arhgef26						0.57								Rho guanine nucleotide exchange factor 26
arhgef28				0.60		0.52								A-kinase anchor protein 28
arhgef37	-1.01	-0.63	-1.01										K20710	Rho guanine nucleotide exchange factor 37
arid4a						0.49	Loss						K19194	AT-rich interactive domain-containing protein 4A

arid4b	-0.31		-0.39											AT-rich interactive domain-containing protein 4B
arid5b	-0.48					0.56								AT-rich interactive domain-containing protein 5
arih1						0.89								Ariadne-1
arih2						-0.38							K11969	Ariadne-2
arl14		-0.56	-0.69											ADP-ribosylation factor-like protein 14
arl16	-0.55		-0.48											
arl2bp	-0.49													ADP-ribosylation factor-like protein 2-binding protein
arl3				-0.36										ADP-ribosylation factor-like protein 3
arl4c	0.49					-0.46								ADP-ribosylation factor-like protein 4
arl6ip1	0.40													ADP-ribosylation factor-like protein 6-interacting protein 1
arl6ip4				-0.36		-0.44								
arl6ip5						0.56	Gain							PRA1 family protein 3
arl9						-0.67							K07957	ADP-ribosylation factor-like protein 9
armc5						-0.72							K22499	Armadillo repeat-containing protein 5
armc6	0.42		0.29											Armadillo repeat-containing protein 6
armc7												Gain	K24825	Armadillo repeat-containing protein 7
armc8						0.52								Armadillo repeat-containing protein 8
armc9						-0.54							K22864	Lish domain-containing protein ARMC9
armh4						-0.95								
arpc2			0.44											Actin related protein 2/3 complex, subunit 2
arpp19	0.52													
arpp21	-0.38		-0.39											
arrdc2	0.54													
arrdc3	0.51		-0.65										K20910	Thioredoxin-interacting protein
arsg	-0.48												K12381	Arylsulfatase G
arvcf	-0.57		-0.50			0.61							K05690	Catenin (cadherin-associated protein), delta 1
arx						0.77							K09452	Homeobox protein aristaless-related
asah1	-0.60		-0.37											Acid ceramidase
asah2	-1.55	-1.86	-2.35											Neutral ceramidase
asap2							Gain						K12488	Arf-GAP with SH3 domain, ANK repeat and PH domain-containing protein
asb10	-1.29		-0.79								Loss	Loss	K10332	Ankyrin repeat and SOCS box protein 10
asb14						-0.63								Ankyrin repeat and SOCS box protein 14

asb2	0.48					-0.47							K10324	Ankyrin repeat and SOCS box protein 2
asb5	-0.98													
asb6	-0.77	-0.40	-0.42										K10328	Ankyrin repeat and SOCS box protein 6
asb8	-0.75		-0.95			-1.67							K10330	Ankyrin repeat and SOCS box protein 8
ascc2								Loss	Loss					Activating signal cointegrator complex subunit 2
ash2l			0.38										K14964	Set1/Ash2 histone methyltransferase complex subunit ASH2
aspn						0.83								Asporin
asxl2						0.78							K11471	Additional sex combs-like protein
atad2	0.62	0.58	0.65										K22531	Atpase family AAA domain-containing protein 2
atad2b	0.40	0.33	0.39			-0.43						Gain	K22531	Atpase family AAA domain-containing protein 2
atat1						0.48								Alpha-tubulin N-acetyltransferase 1
ate1						-0.39								Arginyl-trna---protein transferase
atf6						-0.55							K09054	Cyclic AMP-dependent transcription factor ATF-6 alpha
atg10						0.60							K17888	Ubiquitin-like-conjugating enzyme ATG10
atg101	-0.92	-0.40	-0.94										K19730	Autophagy-related protein 101
atg13	-0.46		-0.36											Autophagy-related protein 13
atg14	-0.93	-0.60	-0.69											Beclin 1-associated autophagy-related key regulator
atg16l1						0.30							K17890	Autophagy-related protein 16-1
atg2a	-1.04	-0.55	-0.68										K17906	Autophagy-related protein 2
atg2b	-1.47	-1.05	-1.55	0.46									K17906	Autophagy-related protein 2
atg4b	-0.67		-0.45	0.52		0.64								Cysteine protease ATG4
atg4c	-0.40													Cysteine protease ATG4
atg9a	-0.64		-0.89											Autophagy-related protein 9
atic	0.48													Phosphoribosylaminoimidazolecarboxamide formyltransferase / IMP cyclohydrolase
atm	0.79	0.94	0.75										K04728	Serine-protein kinase ATM
atmin						0.61								
atn1						-0.88								
atox1						0.49								
atp10b				0.56										Phospholipid-translocating atpase
atp10d	-0.32							Gain						Phospholipid-translocating atpase
atp13a1	0.45		0.59										K14950	Manganese-transporting P-type atpase
atp1b4						-0.48								Sodium/potassium-transporting atpase subunit beta

atp23						0.39								Mitochondrial inner membrane protease ATP23
atp2c1	0.42					0.34								P-type Ca2+ transporter type 2C
atp5f1c												Gain	K02136	F-type H+-transporting atpase subunit gamma
atp5if1	-0.94													Atpase inhibitor, mitochondrial
atp5mf	0.38												K02130	F-type H+-transporting atpase subunit f
atp6ap2				-0.45		-0.72							K19514	Renin receptor
atp6v0a2						-0.57								V-type H+-transporting atpase subunit a
atp6v1d	-0.39		-0.41										K02149	V-type H+-transporting atpase subunit d
atp8a1			-0.38										K01530, K14802	Phospholipid-translocating atpase , phospholipid-transporting atpase
atp9a				-0.39		-0.54							K01530	Phospholipid-translocating atpase
atpaf2	0.52												K07556	ATP synthase mitochondrial F1 complex assembly factor 2
atr	0.66		0.85	0.71										Serine/threonine-protein kinase ATR
atraid	-1.40	-0.80	-1.24											
atrx	0.46	0.66	0.48			0.62								Transcriptional regulator ATRX
atxn10	0.36					0.43							K19323	Ataxin-10
atxn1l				-0.35		-0.70								Ataxin 1/1L
atxn2l						0.47						Gain		Ataxin 2/2L
auh				-0.37										Methylglutaconyl-coa hydratase
aup1	-0.43													Ancient ubiquitous protein 1
aurkaip1						0.44								Aurora kinase A-interacting protein
auts2						-0.88								Autism susceptibility gene 2 protein
aven						0.40								
avl9				0.42										
avpi1			-0.39			-0.64								
axdnd1	-0.58													
axl						0.59								AXL receptor tyrosine kinase
azi2	-0.47	-0.32	-0.41											5-azacytidine-induced protein 2
azin1	-0.34													Ornithine decarboxylase
b3galt4						0.61							K00715	Ganglioside galactosyltransferase
b3galt6				-0.45		-0.48							K00734	Galactosylxylosylprotein 3-beta-galactosyltransferase
b3gat1											Loss	Gain + Loss	K00735	Galactosylgalactosylxylosylprotein 3-beta-glucuronosyltransferase 1

b3gat3	0.62		0.35			0.33								Galactosylgalactosylxylosylprotein 3-beta-glucuronosyltransferase 3
b3gnt3												Loss	K07970	Beta-1,3-N-acetylglucosaminyltransferase 3
b3gnt9						0.54							K18705	Beta-1,3-N-acetylglucosaminyltransferase 9
b3gntl1	0.45													
b4galnt1	0.44												K00725	(N-Acetylneuraminyl)-galactosylglucosylceramide N-acetylgalactosaminyltransferase
b4galnt4						0.64							K09657	Beta-1,4-N-acetylgalactosaminyltransferase 4
b4galt7	0.84		0.51										K00733	Xylosylprotein 4-beta-galactosyltransferase
b4gat1												Gain		Beta-1,4-glucuronyltransferase 1
b9d1				-0.49		-0.59								B9 domain-containing protein 1
b9d2				-0.43										B9 domain-containing protein 2
bad	-0.52			0.48									K02158	Bcl-2-antagonist of cell death
bag1	0.50		0.34											BCL2-associated athanogene 1
bag2				-0.44										BCL2-associated athanogene 2
bag3						-0.79								BCL2-associated athanogene 3
bag4			0.36											BCL2-associated athanogene 4
bag5						-0.47								BCL2-associated athanogene 5
bag6						-0.97								Large proline-rich protein BAG6
bahcc1						-0.49								
baiap2l2								Gain					K23645	BAI1-associated protein 2-like protein 2
banf1	0.45												K21870	Barrier-to-autointegration factor
banp						0.40								
bard1	0.61		0.55											BRCA1-associated RING domain protein 1
batf											Loss			ATF-like basic leucine zipper transcriptional factor
bax						0.56								Apoptosis regulator BAX
baz1b	0.45		0.40											Bromodomain adjacent to zinc finger domain protein 1B
baz2a						-0.38								Bromodomain adjacent to zinc finger domain protein 2A
bbox1	0.58					-1.36							K00471	Gamma-butyrobetaine dioxygenase
bbs1						-0.92							K16746	Bardet-Biedl syndrome 1 protein
bbs4						-0.41							K16531	Bardet-Biedl syndrome 4 protein
bbs9						0.68							K19398	Bardet-Biedl syndrome 9 protein
bcam				-0.44		-0.66								Lutheran blood group glycoprotein
bcap29						0.63							K14009	B-cell receptor-associated protein 31

bcas2	0.38		0.32											Pre-mrna-splicing factor SPF27
bcas3	-0.33													Breast carcinoma-amplified sequence 3
bccip				-0.44										Protein BCP1
bckdha						-0.42							K00166	2-oxoisovalerate dehydrogenase E1 component alpha subunit
bckdk			-0.30										K00905	
bcl2l13	0.61		0.42			-0.45							K15485	Bcl-2-like protein 13
bcl7a						-0.38							K25605	B-cell CLL/lymphoma 7 protein
bcl9			0.42											B-cell CLL/lymphoma 9 protein
bcl9l						-0.32								B-cell CLL/lymphoma 9 protein
bco2						-0.65								Beta,beta-carotene 9',10'-dioxygenase
bcor						-0.82							K23215	Bcl-6 corepressor
bcorl1						-0.65							K26199	BCL-6 corepressor-like protein 1
becn1	-0.58	-0.32	-0.50											Beclin
bet1						-0.54							K08504	Blocked early in transport 1
bfar						-0.36							K15684	Bifunctional apoptosis regulator
bhlha15											Loss	Loss		Class B basic helix-loop-helix protein 8
bicc1							Loss		Loss				K18756	Protein bicaudal C
bicd1											Loss	Loss	K18739	Protein bicaudal D
bicra	0.63		0.55											BRD4-interacting chromatin-remodeling complex-associated protein
bicral	-0.30		-0.26	0.33		0.35								BRD4-interacting chromatin-remodeling complex-associated protein-like
bin3						-0.63							K20120	Bridging integrator 3
birc6						-0.36							K10586	Baculoviral IAP repeat-containing protein 6 (apollon)
blcap				-0.36										
bloc1s1	-0.37		-0.49										K20185	Biogenesis of lysosome-related organelles complex 1 subunit 1
bloc1s5			-0.37											Biogenesis of lysosome-related organelles complex 1 subunit 5
bloc1s6	-0.79	-0.60	-0.65											Biogenesis of lysosome-related organelles complex 1 subunit 6
blzf1				-0.38		-0.71								
bmp2k						0.44								BMP2 inducible kinase
bmp4				-0.41		-0.73								Bone morphogenetic protein 2, bone morphogenetic protein 4
bmp5						-0.94								Bone morphogenetic protein 5
bmp6						-0.55							K16620, K16621	Bone morphogenetic protein 6, bone morphogenetic protein 7

bmpr1a						-0.85								Bone morphogenetic protein receptor type-1A
bmpr1b						-0.45					Loss			Bone morphogenetic protein receptor type-1B
bmpr2						0.59						Gain	K04671	Bone morphogenetic protein receptor type-2
bms1						0.50							K14569	Ribosome biogenesis protein BMS1
bnip3	-2.50	-1.20	-2.40											
bod1l1	0.58	0.55	0.68											
bop1						0.48							K14824	Ribosome biogenesis protein ERB1
borcs6						0.69							K20820	BLOC-1 related complex subunit 6
bpnt1						0.68							K01082	3'(2'), 5'-bisphosphate nucleotidase
braf						0.63							K04365	B-Raf proto-oncogene serine/threonine-protein kinase
brap	-0.41		-0.34	0.29									K10632	BRCA1-associated protein
brat1	0.93	0.70	0.87											BRCA1-associated ATM activator 1
brca1	0.41		0.54											Breast cancer type 1 susceptibility protein
brca2				0.57		0.76								Breast cancer 2 susceptibility protein
brcc3						0.44								BRCA1/BRCA2-containing complex subunit 3
brd3os	0.77													
bri3	-0.61	-0.34	-0.64											
brip1	0.40	0.40	0.45										K15362	Fanconi anemia group J protein
brix1	0.75												K14820	Ribosome biogenesis protein BRX1
brms1l	-0.54	-0.36	-0.60										K19196	Breast cancer metastasis-suppressor 1 and related proteins
brox	-0.59	-0.31	-0.42											
bsdc1	-0.49		-0.35											
bsg				-0.35		-0.36								Basigin
btaf1	0.77	0.40	0.63											TATA-binding protein-associated factor
btbd7	-0.25													BTB/POZ domain-containing protein 7
btbd9	-0.28		-0.55										K10481	BTB/POZ domain-containing protein 9
btg1			-0.50			-0.60								Protein Tob/BTG
btk				0.44		0.69								Bruton agammaglobulinemia tyrosine kinase
btla											Loss		K06707	B- and T-lymphocyte attenuator
bub3	0.66		0.67										K02180	Cell cycle arrest protein BUB3
bves			-0.42	-0.50		-0.92							K21108	Blood vessel epicardial substance
c10h4orf47	-0.63					-0.62								
c11h8orf37			-0.34											Protein c8orf37

c12h1orf52				-0.33										
c13h1orf50						0.96								
c13h20orf85	-0.56													
c13h3orf18	0.65													
c13h6orf89						0.52								
c14h16orf87				-0.43										
c16h19orf47	0.79	0.41	0.55											
c16h19orf54				-0.66		-0.44								
c17h20orf27	-0.66	-0.64	-0.71											
c18h10orf88						-0.59								
c18h16orf91	0.51													
c18h7orf50	0.51		0.43			0.40								
c19h18orf25	0.27													
c19h9orf16						-0.96								
c1d	0.87												K12592	Exosome complex protein LRP1
c1galt1			0.46	0.56									K00731	Glycoprotein-N-acetylgalactosamine 3-beta-galactosyltransferase
c1galt1c1											Gain		K09653	C1GALT1-specific chaperone 1
c1h1orf174						0.40	Gain					Gain		
c1h1orf74				-0.40										
c1qbp	0.64			-0.45										Complement component 1 Q subcomponent-binding protein, mitochondrial
c1qtnf5	0.62												K24212	Complement C1q and tumor necrosis factor-related protein 5
c20h21orf62	-0.41													
c20h2orf49	0.43					-0.70								Ashwin
c20h2orf76				-0.35										
c21h12orf57						0.68								
c21h1orf131	0.79											Gain		
c21h1orf198				-0.31		-0.32								
c22h18orf21	-2.06	-1.19	-2.72											
c22h6orf62						0.30								
c2cd2	0.32													
c2cd2l						-0.47								
c2cd5						-0.30								

c2h17orf53												Loss		
c2h17orf75				0.35		0.30								
c4h11orf98				-0.48		-0.51								
c4h12orf45				-0.39									K26142	NOP protein chaperone 1
c4h12orf66			-0.47										K23297	KICSTOR complex protein c12orf66
c4h2orf42	0.61	0.40	0.94									Gain		
c5			0.50											Complement component 5
c5h14orf180				0.35		0.48								
c5h15orf41	-0.76	-0.41	-0.92										K24865	CDAN1-interacting nuclease 1
c5h2orf50						-1.05								
c6h12orf49			-0.33											
c6h12orf65			-0.44											Peptide chain release factor
c6h2orf68	0.38													
c6h5orf51				-0.40		-0.55								
c6h9orf116			-0.56											
c6h9orf85				-0.36		0.37								
c7h17orf49	0.33		0.49										K23406	Chromatin complexes subunit BAP18
c7h5orf24				-0.45		-0.83								
c8h14orf119			-0.36											
c8h5orf22				-0.39										
c9h11orf58	0.33					0.57								
c9h11orf96		-0.49	-0.57											
c9h12orf73						0.37								
c9h16orf70	-0.66	-0.58	-0.59											
c9orf72				-0.37		-0.38							K23609	Guanine nucleotide exchange protein c9orf72
cab39						0.41							K08272	Calcium binding protein 39
cacfd1			-0.48											
cacnb1				-0.43		-0.86							K04862	Voltage-dependent calcium channel beta-1
cacng1						0.63								Voltage-dependent calcium channel gamma-1
cactin						0.37					Gain	Gain		Cactin
cad	0.57	0.39	0.35											Aspartate carbamoyltransferase catalytic subunit , carbamoyl-phosphate synthase / aspartate carbamoyltransferase / dihydroorotase , dihydroorotase
cadps2				0.53										Calcium-dependent secretion activator

calcoco1	-0.76		-0.96										K25828	Calcium-binding and coiled-coil domain-containing protein 1
calcoco2	-2.28	-1.24	-2.14										K21348	Calcium-binding and coiled-coil domain-containing protein 2
calm2				-0.33										Calcium-binding protein CML, calmodulin
calm3						0.42								Calcium-binding protein CML, calmodulin
camkmt	0.34	0.32				0.62							K18826	Calmodulin-lysine N-methyltransferase
camlg	0.40		0.32	-0.35										Calcium signal-modulating cyclophilin ligand
camsap2						-0.53								Calmodulin-regulated spectrin-associated protein
camsap3				0.47						Gain		Gain		Calmodulin-regulated spectrin-associated protein
camta2			-0.33										K21596	Calmodulin-binding transcription activator
cand1						-0.56								Cullin-associated NEDD8-dissociated protein 1
cap1			0.42											Adenylyl cyclase-associated protein
capn10	0.72	0.31	0.55											Calpain-10
capn7	-0.97	-0.76	-1.11											Calpain-7
caprin1						0.42							K18743	Caprin-1
capsl			-0.60											Calcyphosin
capzb	-0.37		-0.37										K10365	F-actin-capping protein subunit beta
card14				0.54										Caspase recruitment domain-containing protein 14
carm1	0.65	0.32	0.41											Type I protein arginine methyltransferase
carmil1	0.39												K20493	Leucine-rich repeat-containing protein 16
carns1							Gain						K14755	Carnosine synthase
cars1	0.67	0.38											K01883	Cysteinyl-trna synthetase
cars2						0.69							K01883	Cysteinyl-trna synthetase
casc3	0.33													Protein CASC3
caskin2						-0.80								CASK-interacting protein
casp2				0.44										Caspase 2
casp7				0.54									K04397	Caspase 7
casp9	-0.63	-0.39	-0.62											Caspase 9
cass4						-0.88								
cast	-0.70		-0.27										K04281	Calpastatin
castor2	-0.57	-0.34	-0.50											Cytosolic arginine sensor for mtorc1 subunit 2
casz1						-0.66								Zinc finger protein castor
cav3	-0.50													Caveolin 3
cbarp											Gain	Gain		

cbfa2t2			-0.41										K22751	CBFA2/RUNX1 translocation partner 2
cbfb						0.47							K25826	Core-binding factor subunit beta
cbl			0.39	0.35										E3 ubiquitin-protein ligase CBL
cblb	-0.60		-0.43	0.35									K22517	E3 ubiquitin-protein ligase CBL-B
cbx1	0.24												K11585	Chromobox protein 1
cbx2			-0.50			-0.51								Chromobox protein 2
cbx3	0.36													Chromobox protein 3
cbx5	0.48	0.31	0.46											Chromobox protein 5
cc2d1a				0.44									K18260	Coiled-coil and C2 domain-containing protein 1
cc2d1b	-0.54		-0.42											Coiled-coil and C2 domain-containing protein 1
ccdc102a												Gain	K16759	Coiled-coil domain-containing protein 102
ccdc107	-0.40													
ccdc117				0.53										
ccdc12				-0.39										Coiled-coil domain-containing protein 12
ccdc124						0.34								
ccdc126	0.40													
ccdc137	0.40													
ccdc167				-0.42		-0.41								
ccdc22		0.37	0.45			-0.35								Coiled-coil domain-containing protein 22
ccdc25	0.71	0.39	0.43			0.38								
ccdc28b	-0.44													
ccdc33											Loss	Loss		
ccdc36	-0.59	-0.44	-0.61										K26085	Interactor of HORMAD1 protein 1
ccdc43				-0.42										
ccdc47	0.63	0.45	0.67			0.49							K24962	PAT complex subunit CCDC47
ccdc50				0.35										Coiled-coil domain-containing protein 50
ccdc51	0.89		0.51											
ccdc57	0.71													
ccdc59	0.37													
ccdc61	0.49		0.67			-0.52								Coiled-coil domain-containing protein 61
ccdc69						0.63								
ccdc81											Gain	Gain		
ccdc84						0.46								Centrosomal AT-AC splicing factor

ccdc86				-0.39										Rrna-processing protein CGR1
ccdc96							Loss							
ccdc97	0.46	0.32	0.49											
cckar						-0.58								Cholecystokinin A receptor
ccm2	-0.35	-0.23	-0.30											
ccm2l			-0.48	0.45		0.86								
ccn1						-0.48								Insulin-like growth factor-binding protein 10
ccna2	0.60		0.83										K06627	Cyclin-A
ccnb1	0.48		0.60											G2/mitotic-specific cyclin-B1
ccnb1ip1												Loss		E3 ubiquitin-protein ligase CCNP1IP1
ccnb3	0.39		0.53	-0.47		-0.45							K21771	G2/mitotic-specific cyclin-B3
ccnc						0.48								Cyclin-C
ccnd2			0.30			-0.37								G1/S-specific cyclin-D2
ccne1	0.87	0.51	0.82											G1/S-specific cyclin-E1
ccne2	1.16	0.70	1.08										K06626	G1/S-specific cyclin-E1
ccng2	-1.77	-1.05	-1.57											Cyclin G2
ccnk						0.75								Cyclin K
ccnl2	0.35												K23966	Cyclin L
ccnq	0.59											Gain		Cyclin-Q
ccnt1						-0.55							K15188	Cyclin T
ccnt2	0.37												K15188	Cyclin T
ccny						0.36								
ccnyl1											Loss	Loss		
ccp110						-0.48								Centrosomal protein CEP110
ccpg1	-0.73	-0.49	-0.87											
ccs						-0.77								Copper chaperone for superoxide dismutase, superoxide dismutase, Cu-Zn family
ccser2						0.50								
ccz1				-0.41		-0.76								
cd276									Gain		Gain		K06746	Immune costimulatory protein B7-H3
cd28				0.56		0.64	Loss							
cd2bp2						0.35							K13099	CD2 antigen cytoplasmic tail-binding protein 2
cd63						0.48								CD63 antigen

cd79a	-0.89	-0.37				-0.82							K06506	CD79A antigen
cd81						0.78								CD81 antigen
cd9	-0.48												K06460	CD9 antigen
cda			2.84	0.29									K01489	Cytidine deaminase
cdan1				0.46		0.60							K19531	Codanin-1
cdc123	0.44													
cdc14a				0.55									K06639	Cell division cycle 14
cdc16	0.68	0.34	0.58			0.33							K03353	Anaphase-promoting complex subunit 6
cdc20			0.59											Cell division cycle 20, cofactor of APC complex
cdc23	0.43		0.59			-0.43							K03355	Anaphase-promoting complex subunit 8
cdc25b	0.65		0.83											M-phase inducer phosphatase 2
cdc37	0.33												K09554	Cell division cycle protein 37
cdc37l1						0.69								Cell division cycle protein 37
cdc40				0.41		0.66							K12816	Pre-mrna-processing factor 17
cdc42bpa						-0.45							K16307	Serine/threonine-protein kinase MRCK
cdc42ep4						-0.73								
cdc45	0.72	0.40	0.63											Cell division control protein 45
cdc6	0.92		0.76						Gain				K02213	Cell division control protein 6
cdc7	0.84	0.55	0.52											Cell division control protein 7
cdc73						0.38								Parafibromin
cdca2			0.57											Cell division cycle-associated protein 2
cdca3			0.69											
cdca5	0.52		0.48											Sororin
cdca7l	1.29	0.58	1.04										K23408	Cell division cycle-associated protein 7
cdcp2						0.80							K24792	CUB domain-containing protein 2
cdh22						-0.66								Cadherin 22
cdh7											Loss			
cdhr1							Loss						K16501	Cadherin-related family member 1
cdk1			0.71											Cyclin-dependent kinase 1
cdk2	0.51	0.56	0.61			-0.46								Cyclin-dependent kinase 2
cdk2ap2	0.43		0.43											
cdk6						-0.35								Cyclin-dependent kinase 4 , cyclin-dependent kinase 6
cdk8						0.54								Cyclin-dependent kinase 8/11

cdk9						0.36							K02211	Cyclin-dependent kinase 9
cdkl5											Gain	Gain		Cyclin-dependent kinase-like
cdkn1b	-0.40		-0.40										K06624	Cyclin-dependent kinase inhibitor 1B
cdkn2d				-0.39		-0.54							K06623	Cyclin-dependent kinase inhibitor 2D
cdo1	-0.89		-0.64											Cysteine dioxygenase
cdon						-0.61							K20033	Cell adhesion molecule-related/down-regulated by oncogenes
cdt1	0.67		0.59			-0.48								Chromatin licensing and DNA replication factor 1
cdv3	0.70	0.36	0.50											
cdyl2	-0.67		-0.59											Chromodomain protein Y
cebpz	0.44												K14832	Ribosome biogenesis protein MAK21
celf1				-0.36		-0.40							K13207	CUG-BP- and ETR3-like factor
celsr1						-0.67								Cadherin EGF LAG seven-pass G-type receptor 1
cemip						-0.60								Cell migration-inducing and hyaluronan-binding protein
cemip2						0.78	Loss					Loss	K22144	Cell surface hyaluronidase
cenpf	0.48		0.63										K11499	Centromere protein F
cenpi	0.42		0.64										K11501	Centromere protein I
cenpj			0.43										K11502	Centromere protein J
cenpk			0.54											Centromere protein K
cenpm	0.41		0.52										K11505	Centromere protein M
cenpn						0.43								Centromere protein N
cenpp	-0.42												K11508	Centromere protein P
cenpv			-0.52											
cep104						-0.77								Centrosomal protein CEP104
cep120												Loss	K16459	Centrosomal protein CEP120
cep128						-0.50								Centrosomal protein CEP128
cep135				0.36									K16461	Centrosomal protein CEP135
cep164				0.31		0.47							K16462	Centrosomal protein CEP164
cep192				0.36										Centrosomal protein CEP192
cep350						-0.71							K16768	Centrosomal protein CEP350
cep57l1	0.44		0.42											Centrosomal protein CEP57
cep63											Loss			Centrosomal protein CEP63
cep72	-0.49													Centrosomal protein CEP72
cep76			0.63										K16457	Centrosomal protein CEP76

cep78				0.42		0.97							K16765	Centrosomal protein CEP78
cep85	-1.41	-0.64	-1.81			0.58							K16766	Centrosomal protein CEP85
cept1						-0.53							K00994, K13644	Choline/ethanolamine phosphotransferase , diacylglycerol cholinephosphotransferase
cers5	0.55												K23727	Sphingoid base N-palmitoyltransferase
cers6				0.41		0.68						Loss		Sphingoid base N-palmitoyltransferase
cetn3	0.57		0.43										K16466	Centrin-3
cetp	-0.61												K16835	Cholesteryl ester transfer protein
cfap157						-0.70							K25609	Cilia- and flagella-associated protein 157
cfap298	0.39					0.54							K24229	Cilia- and flagella-associated protein 298
cfap36						-0.43							K25395	Cilia- and flagella-associated protein 36
cfap45				0.55									K25551	Cilia- and flagella-associated protein 45
cfap52												Loss		Cilia- and flagella-associated protein 52
cfap69							Loss							Cilia- and flagella-associated protein 69
cfdp1		0.30	0.38											
cfl1						0.46								Cofilin
cfl2						0.56								Cofilin
cfp	-0.62		-2.01			1.06							K15412	Complement factor properdin
cftr						-0.41						Loss	K05031	Cystic fibrosis transmembrane conductance regulator
cgnl1	-0.40													Cingulin-like protein 1
chac1	0.73													Glutathione-specific gamma-glutamylcyclotransferase
chad				-0.47		-0.68								Chondroadherin
chaf1a	0.68	0.46	0.77										K10750	Chromatin assembly factor 1 subunit A
chaf1b	0.45		0.51											Chromatin assembly factor 1 subunit B
chchd1	0.42			-0.46		-0.63								
chchd2	0.59												K22758	Coiled-coil-helix-coiled-coil-helix domain-containing protein 2
chchd4				-0.41									K17782	Mitochondrial intermembrane space import and assembly protein 40
chchd5	0.54													
chd1						-0.71								Chromodomain-helicase-DNA-binding protein 1
chd1l	0.44	0.40	0.54	0.43									K20092	Chromodomain-helicase-DNA-binding protein 1-like
chd2	-0.35		-0.39										K11367, K20091	Chromodomain-helicase-DNA-binding protein 1 , chromodomain-helicase-DNA-binding protein 2
chd5						0.86							K11643, K14435	Chromodomain-helicase-DNA-binding protein 4 , chromodomain-helicase-DNA-binding protein 5

chd6	0.24													Chromodomain-helicase-DNA-binding protein 6
chd7				-0.37		-0.57								Chromodomain-helicase-DNA-binding protein 7
chd8	0.56	0.35	0.46											Chromodomain helicase DNA binding protein 8
chek1	0.50		0.54											Serine/threonine-protein kinase CHEK1
cherp			0.39			-0.38								Calcium homeostasis endoplasmic reticulum protein
chfr	-0.45			0.47									K10644	E3 ubiquitin-protein ligase CHFR
chmp4b						-0.37							K12194	Charged multivesicular body protein 4A/B
chmp7	-0.64		-0.40											Charged multivesicular body protein 7
chn1						-0.68							K20630	Chimaerin
chrac1				-0.45		-0.43								Chromatin accessibility complex protein 1
chrna1						-0.62								Nicotinic acetylcholine receptor alpha-1
chrna9						0.63							K04810	Nicotinic acetylcholine receptor alpha-9
chrnd	-0.33													Nicotinic acetylcholine receptor delta
chst14												Gain + Loss		Dermatan 4-sulfotransferase 1
chst2												Gain	K04745	Carbohydrate 6-sulfotransferase 2
chst7						0.56							K04743	Chondroitin 6-sulfotransferase 7
chsy1	0.56		0.54											Chondroitin sulfate synthase
chtf18	0.82	0.90	0.62											Chromosome transmission fidelity protein 18
chtf8	0.58		0.80										K11270	Chromosome transmission fidelity protein 8
ciao1						0.77								Cytosolic iron-sulfur protein assembly protein CIAO1
ciao2b						0.49						Gain		
ciapin1						0.54								Anamorsin
cic						-0.33							K20225	Capicua transcriptional repressor
cideb						-0.76								Cell death activator CIDE-B
cilp				-0.48		-0.82							K24436	Cartilage intermediate layer protein
cinp	0.45					-0.52								Cyclin-dependent kinase 2-interacting protein
cish	-1.70	-0.41	-1.84	-0.45										Cytokine inducible SH2-containing protein
ckap4	0.74										Loss	Loss	K13999	Cytoskeleton-associated protein 4
ckap5				0.45		0.71								Cytoskeleton-associated protein 5
clcn1	-0.82		-0.43										K05010	Chloride channel 1, skeletal muscle
clcn2						0.80								Chloride channel 2
clcn3						0.37								Chloride channel 3/4/5

clcn5	-0.32		-0.39											Chloride channel 3/4/5
clcn6			-0.44			0.47								Chloride channel 6, chloride channel 7
cldn34												Gain	K06087	Claudin
clec3a						-0.72							K17519	C-type lectin domain family 3 member A
clic2	-0.55													Chloride intracellular channel protein 2
clic4				-0.46		-0.41							K05024	Chloride intracellular channel protein 4
clip4	-0.96	-0.38	-0.67										K10423	CAP-Gly domain-containing linker protein 3/4
clk2				0.52		0.34							K08823	Dual specificity protein kinase CLK2/3
cln3	-0.33													Battenin
cln5	-0.50		-0.41										K12390	Ceroid-lipofuscinosis neuronal protein 5
cln6	-0.41	-0.34	-0.44	0.48									K12359	Ceroid-lipofuscinosis neuronal protein 6
cln8			-0.46										K12360	Ceroid-lipofuscinosis neuronal protein 8
clock	-0.51		-0.33										K02223	Circadian locomoter output cycles kaput protein
clpb	-0.69	-0.55	-0.52											
clpp	0.35												K01358	ATP-dependent Clp protease, protease subunit
clptm1	0.38			-0.38		-0.55								
clptm1l			0.39											
clspn	1.12	0.45	0.91									Loss	K25773	Claspin
cltc			0.29											Clathrin heavy chain
cluap1						0.48							K19684	Clusterin-associated protein 1
cluh	0.42			-0.44									K03255	Protein TIF31
cmss1						0.50								Protein CMS1
cmtm4						-0.44								
cmtm6						-0.26								
cmtm7						0.43								
cnih1	0.41					0.46		Gain	Gain					Protein cornichon
cnksr2												Gain		Connector enhancer of kinase suppressor of Ras 2
cnmd							Gain			Gain	Gain	Gain + Loss		
cnot11			0.48											
cnot6l	-0.56												K12603	CCR4-NOT transcription complex subunit 6
cnot8			0.47										K12581	CCR4-NOT transcription complex subunit 7/8
cnot9	0.33													CCR4-NOT transcription complex subunit 9

cnppd1	-0.41		-0.43											
cnpy4	0.45			-0.35										Protein canopy 3/4
cntfr				-0.55		-1.04								Ciliary neurotrophic factor receptor
cntln				0.42										Centlein
cntnap2							Gain				Loss	Loss	K07380	Contactin associated protein-like 2
cntnap5												Loss		Contactin associated protein-like 5
cog3				0.49		0.76								Conserved oligomeric Golgi complex subunit 3
cog4				0.41									K20291	Conserved oligomeric Golgi complex subunit 4
cog8			0.42											Conserved oligomeric Golgi complex subunit 8
coil			0.42								Loss	Loss	K13150	Coilin
col12a1						-0.81								Collagen type XII alpha
col1a2			0.54										K06236	Collagen type I alpha
col20a1						-1.00								Collagen type XX alpha
col22a1						0.83								Collagen type XXII alpha
col25a1	-0.39		-0.36											Collagen type XXV alpha
col28a1									Gain		Gain		K23619	Collagen type XXVIII alpha
col4a1						0.82								Collagen type IV alpha
col4a5						-0.74							K06237	Collagen type IV alpha
col4a6						-0.61								Collagen type IV alpha
col5a1				-0.53		-0.86								Collagen type V/XI/XXIV/XXVII, alpha
col5a2						-0.37								
col8a2						-0.43							K23455	Collagen type VIII alpha
col9a2											Loss	Loss		Collagen type IX alpha
commd6						0.54							K22562	COMM domain containing 6
cop1	0.35		0.43										K10143	E3 ubiquitin-protein ligase RFWD2
copa			0.57										K05236	Coatomer subunit alpha
copb1	0.32		0.62										K17301	Coatomer subunit beta
copb2			0.42										K17302	Coatomer subunit beta'
copg2			0.42											Coatomer subunit gamma
cops3						0.28							K12177	COP9 signalosome complex subunit 3
cops6	0.38		0.32										K12179	COP9 signalosome complex subunit 6
cops7a												Loss		COP9 signalosome complex subunit 7
coq10b						-0.63								Coenzyme Q-binding protein COQ10

coq4	-0.66		-0.47											Ubiquinone biosynthesis protein COQ4
coq5						0.59							K06127	2-methoxy-6-polyprenyl-1,4-benzoquinol methylase
cpa6						-1.51								Carboxypeptidase A6
cpamd8				-0.51		-1.25								CD109 antigen
cpd						-0.78								Carboxypeptidase D
cpeb3						-0.60								Cytoplasmic polyadenylation element-binding protein
cpeb4				-0.89		-1.28								Cytoplasmic polyadenylation element-binding protein
cpn1						-0.50								Carboxypeptidase N catalytic subunit
cpne9						0.43								Copine 5/8/9
cpox	0.42												K00228	Coproporphyrinogen III oxidase
cpq				-0.37		-0.63								Carboxypeptidase Q
cpsf1	0.77	0.61	0.79			0.41							K14401	Cleavage and polyadenylation specificity factor subunit 1
cpsf2	0.61	0.46	0.67			0.60							K14402	Cleavage and polyadenylation specificity factor subunit 2
cpsf6			0.30	-0.35		-0.47								Cleavage and polyadenylation specificity factor subunit 6/7
cpt2				-0.48		-1.04								Carnitine O-palmitoyltransferase 2
cpvl	-0.47					-0.64							K09645	Vitellogenic carboxypeptidase-like protein
cpxm2						0.61							K08639	Carboxypeptidase X2
cracr2a				0.48									K17199	Ras and EF-hand domain-containing protein
cracr2b	-0.48												K17199	Ras and EF-hand domain-containing protein
crb3	-0.58												K06090	Crumbs 3
crbn			-0.40			-0.40								Cereblon
crcp				-0.45									K25304	DNA-directed RNA polymerase III subunit RPC9
creb1											Gain	Gain		Cyclic AMP-responsive element-binding protein 1
creb5						0.52							K09047	Cyclic AMP-responsive element-binding protein 5
crebbp	-0.47													E1A/CREB-binding protein
crebl2			-0.71											
crebrf	-1.13		-1.12			0.67							K21554	CREB3 regulatory factor
creg1						-0.93							K25476	Protein CREG
creg2	-1.59	-0.44	-1.55											Protein CREG
creld2	0.88	0.65	0.70			0.51							K24335	Protein disulfide-isomerase
crhbp						-0.61								Corticotropin-releasing factor-binding protein
crhr1	0.48					0.39								Corticotropin releasing hormone receptor 1
cript			-0.38										K24826	Cysteine-rich PDZ-binding protein

crkl											Loss		K04438	C-crk adapter molecule crk
crnkl1			0.56											Crooked neck
crppa				-0.37									K21031	D-ribitol-5-phosphate cytidylyltransferase
crtap	0.92	0.69	0.91	-0.40										Cartilage-associated protein
crtc1						0.67								CREB-regulated transcription coactivator 1
crtc3	-0.47	-0.41									Gain		K16334	CREB-regulated transcription coactivator 3
cryba2						4.24							K23482	Beta-crystallin
cryba4						1.09							K23482	Beta-crystallin
crybb1						1.12						Gain	K23482	Beta-crystallin
crygn						2.92							K23483	Gamma-crystallin
csdc2						-0.55								
cse1l		0.39	0.46											Exportin-2 (importin alpha re-exporter)
csf3r				0.42		0.79								
csgalnact2										Gain		Gain	K00746	Chondroitin sulfate N-acetylgalactosaminyltransferase 1/2
csmd2				-0.49		-0.63							K17495	CUB and sushi domain-containing protein
csnk1g1			0.29	0.30										Casein kinase 1, gamma
csnk1g2	0.43	0.43	0.30											Casein kinase 1, gamma
csnk2a2			0.39										K03097	Casein kinase II subunit alpha
csnk2b	0.44		0.33			0.43							K03115	Casein kinase II subunit beta
cspg5						-0.60								Chondroitin sulfate proteoglycan 5
cstf1			0.57										K14406	Cleavage stimulation factor subunit 1
ctdsp1	-0.40		-0.33										K15731	Carboxy-terminal domain RNA polymerase II polypeptide A small phosphatase
ctdspl	-0.72	-0.70	-0.64											Carboxy-terminal domain RNA polymerase II polypeptide A small phosphatase
ctdspl2				-0.43		-0.89							K17616	CTD small phosphatase-like protein 2
cthrc1	0.45												K25700	Collagen triple helix repeat-containing protein 1
ctif						0.35								
ctnnal1			-0.39											
ctnnb1			0.33											Catenin beta 1
ctnnbip1	0.55	0.28	0.48										K04493	Beta-catenin-interacting protein 1 (inhibitor of beta-catenin and Tcf-4)
ctnnbl1	0.53		0.44											Beta-catenin-like protein 1
ctns	-0.65	-0.45	-0.71										K12386	Cystinosin

ctps1	0.61		0.37											CTP synthase
ctsa	-0.42													Cathepsin A (carboxypeptidase C)
ctsf	-0.58		-0.57											Cathepsin F
ctsl	-0.79		-0.74										K01365	Cathepsin L
ctsz						0.68								Cathepsin X
cttnbp2				0.51		0.80								
ctu1				-0.37		-0.41							K14168	Cytoplasmic trna 2-thiolation protein 1
cuedc1							Loss		Loss					
cuedc2						-0.79								
cul4b						0.38								Cullin 4
cul9				0.33									K11970	Cullin 9
cutc						0.43								Copper homeostasis protein
cux1						-0.51							K09313	Homeobox protein cut-like
cux2						-0.63							K09313	Homeobox protein cut-like
cwf19l1	0.65	0.46	0.56											CWF19-like protein 1
cxcl14						-0.43								C-X-C motif chemokine 14
cxcr4										Loss				C-X-C chemokine receptor type 4
cyhr1	-1.19	-0.91	-1.32											
cyth2	-0.31		-0.32										K18441	Cytohesin
cytip							Gain					Loss	K24053	General receptor for phosphoinositides 1-associated scaffold protein
cytl1							Loss							Cytokine-like protein 1
cyyr1	1.28		0.52											
caap1	0.59		0.49											
d2hgdh	-0.56		-1.30											D-2-hydroxyglutarate dehydrogenase
dab1						-0.46							K20054	Disabled homolog 1
dab2				0.55		0.53							K12475	Disabled homolog 2
dad1			0.61			0.36							K12668	Oligosaccharyltransferase complex subunit epsilon
dag1				-0.39										Dystroglycan 1
daglb			-0.38										K13806	Sn1-specific diacylglycerol lipase
dalrd3							Gain							DALR anticodon-binding domain-containing protein 3
dao				-0.51		-0.64							K00273	D-amino-acid oxidase
dapk1				0.57									K08803	Death-associated protein kinase

dapk3												Loss		Death-associated protein kinase
dapp1						0.58							K12229	Dual adapter for phosphotyrosine and 3-phosphotyrosine and 3-phosphoinositide
dars2						-0.55							K01876	Aspartyl-trna synthetase
dazap1	0.62	0.49	0.39										K14411	RNA-binding protein Musashi
dazap2	-0.39		-0.38			-0.78								
dbf4	0.44		0.46										K06629	Protein DBF4
dbn1						0.36	Gain							Drebrin
dbnl						-0.42								Drebrin-like protein
dbr1	0.36	0.38	0.46			-0.63							K18328	Lariat debranching enzyme
dbt						-0.39							K09699	2-oxoisovalerate dehydrogenase E2 component (dihydrolipoyl transacylase)
dcaf11	-0.60		-0.65			0.46								DDB1- and CUL4-associated factor 11
dcaf12	-0.54		-0.61											DDB1- and CUL4-associated factor 12
dcaf15	-0.58		-0.42											DDB1- and CUL4-associated factor 15
dcaf4	-0.55		-0.61										K11799	DDB1- and CUL4-associated factor 4
dcaf7				-0.52		-0.87							K11805	DDB1- and CUL4-associated factor 7
dcaf8	-0.48					-0.76								DDB1- and CUL4-associated factor 8
dcakd						-0.55								Dephospho-coa kinase
dcc						-0.69								Deleted in colorectal carcinoma
dck						-0.48							K00893	Deoxycitidine/deoxyadenosine/deoxyguanosine kinase
dclre1c				0.74		1.01							K10887	DNA cross-link repair 1C protein
dcp1a			-0.52			-0.49								Mrna-decapping enzyme 1A , mrna-decapping enzyme 1B
dctn2	0.41	0.32	0.37											Dynactin 2
dctpp1	0.99	0.93	1.09										K16904	Dctp diphosphatase
dcun1d3						-0.53								DCN1-like protein 3
dcun1d4						0.55							K17824	DCN1-like protein 4/5
ddah1						0.39								Dimethylargininase
ddb1				-0.24		-0.47							K10610	DNA damage-binding protein 1
ddhd1				0.58		0.82							K13619	Phospholipase DDHD1
ddias								Loss				Loss		
ddit3						-0.66							K04452	DNA damage-inducible transcript 3
ddost	0.58	0.36	0.54											Oligosaccharyltransferase complex subunit beta
ddx10				-0.36										ATP-dependent RNA helicase DDX10/DBP4

ddx11	0.78		0.57									Gain		Chromosome transmission fidelity protein 1
ddx20	0.37												K13131	ATP-dependent RNA helicase DDX20
ddx23	0.31													ATP-dependent RNA helicase DDX23/PRP28
ddx27						0.51							K13181	ATP-dependent RNA helicase DDX27
ddx28												Loss	K20096	ATP-dependent RNA helicase DDX28
ddx39a			0.42			0.44								ATP-dependent RNA helicase DDX39 , ATP-dependent RNA helicase UAP56/SUB2
ddx3x	0.73	0.51	0.41										K11594	ATP-dependent RNA helicase DDX3X
ddx41				-0.37		-0.32								ATP-dependent RNA helicase DDX41
ddx42	0.33			0.38		0.28							K12835	ATP-dependent RNA helicase DDX42
ddx47	0.58	0.40	0.57			0.51								ATP-dependent RNA helicase DDX47/RRP3
ddx5			0.39										K12823	ATP-dependent RNA helicase DDX5/DBP2
ddx51	0.47												K14807	ATP-dependent RNA helicase DDX51/DBP6
ddx52	0.65													ATP-dependent RNA helicase DDX52/ROK1
ddx54						0.48								ATP-dependent RNA helicase DDX54/DBP10
ddx56				-0.45		-0.35								ATP-dependent RNA helicase DDX56/DBP9
def8			-0.40											
dennd10						-0.48								
dennd1a				0.45		0.89								DENN domain-containing protein 1
dennd2a						-0.50							K20161	DENN domain-containing protein 2
dennd2c			0.47		-0.73						Gain	Gain	K20161	DENN domain-containing protein 2
dennd4a						0.82							K20163	DENN domain-containing protein 4
dennd5b	-0.46					0.61							K20164	DENN domain-containing protein 5
denr				-0.43										Density-regulated protein
derl1						0.41							K11519	Derlin-1
derl2				-0.50		-0.34								Derlin-2/3
des						-0.48					Gain	Gain		Desmin
dexi	-0.58		-0.62											
dffa			0.43									Gain		DNA fragmentation factor, 45 kd, alpha subunit
dgkd	-0.57		-0.44											Diacylglycerol kinase (ATP)
dgke						0.46							K00901	Diacylglycerol kinase (ATP)
dgkh						-0.36							K00901	Diacylglycerol kinase (ATP)
dgkz						-0.49							K00901	Diacylglycerol kinase (ATP)

dglucy				-0.50		-0.80							K22210	D-glutamate cyclase
dhcr24	0.39													Delta24-sterol reductase
dhdds	0.34		0.34	-0.36									K11778	Ditrans,polycis-polyprenyl diphosphate synthase
dhdh				-0.64		-0.49								Dihydrodiol dehydrogenase / D-xylose 1-dehydrogenase (NADP)
dhps				-0.31		-0.39							K00809	Deoxyhypusine synthase
dhrs1			-0.33											Dehydrogenase/reductase SDR family member 1
dhrs12	-0.37												K11168	Dehydrogenase/reductase SDR family member 12
dhrsx												Loss	K11170	Dehydrogenase/reductase SDR family member X
dhx29	0.36		0.35											ATP-dependent RNA helicase DHX29
dhx30	1.04	0.66	0.51			0.53								ATP-dependent RNA helicase DHX30
dhx33						-0.50							K17820	ATP-dependent RNA helicase DHX33
dhx34	-0.36													ATP-dependent RNA helicase DHX34
dhx35	0.56		0.46											ATP-dependent RNA helicase DDX35
dhx36	0.66	0.60	0.73			0.29							K14442, K21843	ATP-dependent RNA helicase DHX36 , tetratricopeptide repeat protein 7
dhx57	0.59												K13026	ATP-dependent RNA helicase DHX57
diablo						-0.39		Loss	Loss		Loss	Loss	K10522	Diablo
diaph1										Gain				Diaphanous 1
diaph2						0.82								Diaphanous 2
dicer1	0.60	0.46	0.34											Endoribonuclease Dicer
dido1	0.36		0.53											COMPASS component SPP1
dimt1			-0.37										K14191	18S rrna (adenine1779-N6/adenine1780-N6)-dimethyltransferase
dio1	-0.44													Type I thyroxine 5'-deiodinase
dip2c						0.60								
dipk1b									Loss					
dis3						0.52								Exosome complex exonuclease DIS3/RRP44
dkc1	0.85	0.50	0.56											H/ACA ribonucleoprotein complex subunit 4
dlg3												Loss	K21098	Discs large protein 3
dlg5						-0.47								Discs large protein 5
dlgap5	0.43		0.63											Disks large-associated protein 5
dll1	0.50													Delta
dmac2l						0.52							K07554	ATP synthase, H+ transporting, mitochondrial F0 complex, subunit s
dmap1	0.47		0.44											DNA methyltransferase 1-associated protein 1

dmgdh						-0.79							K00315	Dimethylglycine dehydrogenase
dmxl1				0.39		0.33							K24155	Rabconnectin-3a
dmxl2						0.47								Rabconnectin-3a
dna2	0.47		0.42								Gain			DNA replication ATP-dependent helicase/nuclease Dna2
dnaja2	0.38	0.31				0.33								Dnaj homolog subfamily A member 2
dnaja3				-0.37									K09504	Dnaj homolog subfamily A member 3
dnajb11	0.72	0.37	0.42											Dnaj homolog subfamily B member 11
dnajb4		-0.37										Gain		Dnaj homolog subfamily B member 4
dnajb5						0.79								Dnaj homolog subfamily B member 5
dnajc1	0.48	0.45	0.49			0.62								Dnaj homolog subfamily C member 1
dnajc12	-0.48					-0.96								Dnaj homolog subfamily C member 12
dnajc13						0.32							K09533	Dnaj homolog subfamily C member 13
dnajc14	-0.54					0.43								Dnaj homolog subfamily C member 14
dnajc17											Loss	Loss	K09537	Dnaj homolog subfamily C member 17
dnajc21	0.59	0.41	0.57											Dnaj homolog subfamily A member 5
dnajc22			0.58	0.49										Dnaj homolog subfamily C member 22
dnajc24	0.30												K17867	Diphthamide biosynthesis protein 4
dnajc25	0.43	0.37				0.51							K19371	Dnaj homolog subfamily C member 25
dnajc5g	0.43													Dnaj homolog subfamily C member 5
dnajc8			0.22	-0.42									K09528	Dnaj homolog subfamily C member 8
dnajc9	0.55													Dnaj homolog subfamily C member 9
dner							Loss	Loss	Loss			Gain	K24463	Delta and Notch-like epidermal growth factor-related receptor
dnm2	-0.34					-0.34								Dynamin 1/3
dnmt1	0.62	0.58	0.52										K00558	DNA (cytosine-5)-methyltransferase 1
dnmt3a						0.59						Gain	K17398	DNA (cytosine-5)-methyltransferase 3A
dnph1	0.78		0.69											2'-deoxynucleoside 5'-phosphate N-hydrolase
dnaaf1											Gain	Gain	K19750	Dynein axonemal assembly factor 1
dnaaf5	0.48		0.41										K19759	Dynein axonemal assembly factor 5
dock1				0.42		0.54							K13708	Dedicator of cytokinesis protein 1
dock4						-0.51							K17697	Dedicator of cytokinesis protein 4
dock5						-0.54							K17707	Dedicator of cytokinesis protein 5
dock7						-1.07								Dedicator of cytokinesis protein 6/7/8
dock8							Loss	Loss				Loss	K21852	Dedicator of cytokinesis protein 6/7/8

dolk				-0.52		-0.68							K00902	Dolichol kinase
dolpp1	0.31													Dolichyldiphosphatase
donson	0.97		0.52										K22422	Protein downstream neighbor of Son
dop1a	0.38		0.40											
dot1l						0.67								
dpf2				0.44		1.00							K13196	Zinc finger protein ubi-d4
dph2	0.78												K17866	Diphthamide biosynthesis protein 2
dph5						0.71							K00586	Diphthine methyl ester synthase
dph7	-0.75		-0.52											Diphthine methyl ester acylhydrolase
dpp7	-0.38					0.45							K01276	Dipeptidyl-peptidase II
dpp9						0.36								Dipeptidyl-peptidase 9
dpt									Gain	Loss		Loss	K25512	Dermatopontin
dpy19l3						0.53							K24553	C-mannosyltransferase DPY19L
dr1				-0.56		-1.07								Down-regulator of transcription 1
dram1	-0.53	-0.80												DNA damage-regulated autophagy modulator protein 1
draxin									Gain					Draxin
drosha						0.48							K03685	Ribonuclease III
dscc1	0.63		0.68										K11271	Sister chromatid cohesion protein DCC1
dse			0.37										K01794	Chondroitin-glucuronate 5-epimerase
dsel						-0.48								Chondroitin-glucuronate 5-epimerase
dstyk	-0.73	-0.61	-0.66											Dual serine/threonine and tyrosine protein kinase
dtd2	0.57	0.48	0.52										K07560	D-aminoacyl-trna deacylase
dtl	0.55	1.03	0.64											Denticleless
dtna			-0.59											
dtnb	-0.38													
dtwd1				-0.63		-0.80							K25070	Trna-uridine aminocarboxypropyltransferase
dtwd2	-0.52		-0.60											Trna-uridine aminocarboxypropyltransferase
duox1	-0.69												K13411	Dual oxidase
dus4l						0.28								Trna-dihydrouridine synthase 4
dusp1	-0.35		-0.36										K04459, K21278	Dual specificity MAP kinase phosphatase , dual specificity protein phosphatase 1
dusp13	-0.99													Atypical dual specificity phosphatase
dusp23						0.72								

dusp28				-0.49		-0.63								
dusp6			-0.46											Dual specificity MAP kinase phosphatase , dual specificity phosphatase 6
dut	0.53												K01520	Dutp pyrophosphatase
dvl1				0.53		0.81								Segment polarity protein dishevelled
dvl2				-0.50		-1.19								Segment polarity protein dishevelled
dxo				0.56										RAT1-interacting protein
dync1li2		0.30												Dynein cytoplasmic 1 light intermediate chain
dyrk2	-0.56		-0.55				Gain						K18669	Dual specificity tyrosine-phosphorylation-regulated kinase 2/3/4
daam2			-0.32										K04512	Dishevelled associated activator of morphogenesis
e2f1	0.73	0.65	0.58			0.36							K17454	Transcription factor E2F1
e2f2	0.54					-0.81							K09389	Transcription factor E2F2
e2f3	0.95	0.96	0.80				Gain + Loss	Loss	Loss				K06620	Transcription factor E2F3
e2f4	0.28	0.31	0.46										K04682	Transcription factor E2F4/5
e2f7	0.70	0.44	0.50											Transcription factor E2F7/8
e2f8	0.56	0.42	0.73										K09391	Transcription factor E2F7/8
eapp	-0.42													
ears2	0.77		0.53											Glutamyl-trna synthetase
ebna1bp2	0.69					0.49							K14823	Rrna-processing protein EBP2
ecd						0.31								
ece1	-0.45					-0.41							K01415	Endothelin-converting enzyme
ecel1						0.41	Loss							Endothelin-converting enzyme-like 1
ecm2						-0.72								Extracellular matrix protein 2
ecpas		0.33											K11886	Proteasome component ECM29
ecsit	0.58			-0.51										Evolutionarily conserved signaling intermediate in Toll pathway
ect2			0.42			-0.72								Protein ECT2
eda						-0.55								Ectodysplasin-A
edaradd						-0.52							K23324	Ectodysplasin-A receptor-associated adapter protein
edc4	0.33													Enhancer of mrna-decapping protein 4
edn3						-0.73							K05227	Endothelin-3
ednrb				-0.37									K04198	Endothelin receptor type B
eea1			-0.28										K12478	Early endosome antigen 1
eed	0.74	0.65	0.87										K11462	Polycomb protein EED

eef2k	-1.81	-0.59	-1.46			-0.76								Elongation factor 2 kinase
efcab14	-0.57													
efcab6						1.01							K23851	EF-hand calcium-binding domain-containing protein 6
efcab7	-0.41		-0.52											EF-hand calcium-binding domain-containing protein 7
efcc1						-0.40								
efhd2						-0.39								EF-hand domain-containing family member D2
efr3b			-0.34										K21842	Protein EFR3
eftud2	0.92	0.57	0.88											116 kda U5 small nuclear ribonucleoprotein component
egr3			-0.43										K12497	Early growth response protein 3
ehd2						-0.94							K12469, K12483	EH domain-containing protein 1, EH domain-containing protein 2
ehmt1	0.34					-0.50								
ehmt2			0.41										K11420	
ei24	-0.85	-0.40	-0.85										K10134	Etoposide-induced 2.4 mrna
eif1ad	0.36		0.32										K15025	Probable RNA-binding protein EIF1AD
eif2ak1	-0.39												K16194	Eukaryotic translation initiation factor 2-alpha kinase 1
eif2d						0.43								Translation initiation factor 2D
eif2s2						0.60							K03238	Translation initiation factor 2 subunit 2
eif2s3						0.53								Translation initiation factor 2 subunit 3
eif3a				-0.53		-0.57							K03254	Translation initiation factor 3 subunit A
eif3f						0.57							K03249	Translation initiation factor 3 subunit F
eif3i						0.41								Translation initiation factor 3 subunit I
eif3j	0.42													Translation initiation factor 3 subunit J
eif4a2	0.54												K03257	Translation initiation factor 4A
eif4b						0.50							K03258	Translation initiation factor 4B
eif4e2						0.67							K03259	Translation initiation factor 4E
eif4e3	-0.85		-0.78			0.53								Translation initiation factor 4E
eif4ebp2	-0.78		-0.80										K18644	Eukaryotic translation initiation factor 4E binding protein 2
eif4ebp3	-0.95		-0.71										K18645	Eukaryotic translation initiation factor 4E binding protein 3
eif4g2						-0.37							K03260	Translation initiation factor 4G
eif4g3	0.47	0.46	0.61									Gain	K03260	Translation initiation factor 4G
eif5	0.67		0.57										K03262	Translation initiation factor 5
eif5a						0.25							K03263	Translation initiation factor 5A

eif6	0.47													Translation initiation factor 6
elac2	0.81		0.36										K00784	Ribonuclease Z
ell						0.65							K15183	RNA polymerase II elongation factor ELL
eloa						-0.55								Elongin-A
elovl6			0.49										K10203	Elongation of very long chain fatty acids protein 6
elp1									Loss				K11373	Elongator complex protein 1
elp2											Gain		K11374	Elongator complex protein 2
elp4	-0.38												K11375	Elongator complex protein 4
emc1	0.53		0.44											ER membrane protein complex subunit 1
emc2						0.60								ER membrane protein complex subunit 2
emc3				-0.47		-0.54								ER membrane protein complex subunit 3
emc4						0.58								ER membrane protein complex subunit 4
emc8				-0.40		-0.44							K23569	ER membrane protein complex subunit 8/9
emc9	0.32		0.40											ER membrane protein complex subunit 8/9
eme1	1.05		0.93										K10882	Crossover junction endonuclease EME1
eme2	-0.54													
emg1	0.49					0.44							K14568	Rrna small subunit pseudouridine methyltransferase Nep1
emid1						0.83					Loss	Loss		EMI domain-containing protein 1
emilin1				-0.40		-0.68								Emilin
emilin2											Loss	Loss	K24246	Emilin
eml1	-0.53		-0.67										K18595	Echinoderm microtubule-associated protein-like 1/2
emp2	-0.88	-0.54	-0.87	-0.45										Peripheral myelin protein 22
emsy						-0.59							K25790	BRCA2-interacting transcriptional repressor EMSY
emx2						-0.61								Homeobox protein EMX
en1						0.73								Homeobox protein engrailed
enc1				-0.39		-0.44								Kelch-like protein 25/37 (ectoderm-neural cortex protein)
endog	0.63		0.32										K01173	Endonuclease G, mitochondrial
enoph1						0.51								Enolase-phosphatase E1
enpp4	-0.38													Ectonucleotide pyrophosphatase/phosphodiesterase family member 4
enpp6			0.62											Ectonucleotide pyrophosphatase/phosphodiesterase family member 6
entpd4						0.75								Ectonucleoside triphosphate diphosphohydrolase 4
entpd5											Gain	Gain		Ectonucleoside triphosphate diphosphohydrolase 5/6

entpd6			0.43										K01511	Ectonucleoside triphosphate diphosphohydrolase 5/6
eogt								Gain	Gain					EGF domain-specific O-glcnac transferase
ep400	0.41													E1A-binding protein p400
epb41l2						0.40							K23961	Band 4.1-like protein 1/2/3
epb41l4b				0.47									K21111	Band 4.1-like protein 4B
epc2			-0.35			-0.42							K11322	Enhancer of polycomb-like protein
epg5	-1.37	-1.06	-1.02	0.41										Ectopic P granules protein 5
epha8											Gain	Gain	K05109	Eph receptor A8
ephb4						0.71						Gain		Eph receptor B4
epm2a	-1.82		-1.20											
epn3			0.31										K12471	Epsin
eps15						-0.68								Epidermal growth factor receptor substrate 15
eps8	-0.47	-0.39	-0.47										K17277	Epidermal growth factor receptor kinase substrate 8
epyc					0.78	0.64					Gain	Gain		Epiphycan
erbb2	-0.32					-0.39								Receptor tyrosine-protein kinase erbb-2
erbb4				0.45									K05085	Receptor tyrosine-protein kinase erbb-4
erbin						-0.56							K12796	Erbb2-interacting protein
ercc2	0.89	0.45	0.56	-0.41		-0.49								DNA excision repair protein ERCC-2
ercc4	0.42					-0.43								DNA excision repair protein ERCC-4
ercc5				0.42										DNA excision repair protein ERCC-5
ercc6l			0.56										K20093	DNA excision repair protein ERCC-6-like
ergic1	0.44					0.43							K20365	Endoplasmic reticulum-Golgi intermediate compartment protein 1
ergic3						0.49								Endoplasmic reticulum-Golgi intermediate compartment protein 3
eri1			0.38											3'-5' exoribonuclease 1
eri2	0.42		0.43										K18417	ERI1 exoribonuclease 2
erich3				0.50										
ero1a								Gain				Loss		ERO1-like protein alpha
erp44		0.33	0.34			0.55								Endoplasmic reticulum resident protein 44
errfi1						-0.82								
esco2		0.57	0.67										K11268	N-acetyltransferase
esf1	0.37													
espl1	0.57		0.50											Separase

etf1				-0.39										Peptide chain release factor subunit 1
etfbkmt						0.93								ETFB lysine methyltransferase
etfrf1			-0.38											
etnk1											Loss	Loss		Ethanolamine kinase
etnppl								Loss				Loss		Ethanolamine-phosphate phospho-lyase
ets1						-0.46							K02678	C-ets-1
etv5		0.33												Ets translocation variant 5
etaa1	0.57													
evc2	-0.43					0.47							K19608	Ellis van creveld syndrome protein 2
evi5	-0.66	-0.56	-0.64			-0.45							K20242	Ecotropic viral integration site 5 protein
ewsr1	0.36		0.30	-0.38		-0.83								RNA-binding protein EWS
exd3	-0.45													
exo1	0.63	0.54	1.16											Exonuclease 1
exoc2	-0.35												K17637	Exocyst complex component 2
exoc5				0.39										Exocyst complex component 5
exoc6						0.39							K19985	Exocyst complex component 6
exoc7				0.36										Exocyst complex component 7
exosc10						0.42				Loss	Loss	Loss		Exosome complex exonuclease RRP6
exosc2	0.48													Exosome complex component RRP4
exosc3	0.35													Exosome complex component RRP40
exosc6	0.31			-0.39									K12587	Exosome complex component MTR3
exosc8	0.51		0.41										K12586	Exosome complex component RRP43
exosc9	0.42												K03678	Exosome complex component RRP45
ext1												Gain	K02366	Glucuronyl/N-acetylglucosaminyl transferase EXT1
ext2						0.58								Glucuronyl/N-acetylglucosaminyl transferase EXT2
extl3		0.47	0.51										K02370	Alpha-1,4-N-acetylglucosaminyltransferase EXTL3
eya4						-0.99					Loss			Eyes absent homolog 4
eys								Gain			Loss	Loss	K19601	Protein eyes shut
ezh1	-0.57		-0.50											
ezh2	0.78	0.54	0.89										K11430	
ezr				0.43		0.51							K08007	Ezrin
fabp1			-0.72			0.76							K08750	Fatty acid-binding protein 1, liver
fabp2						0.44							K08751	Fatty acid-binding protein 2, intestinal

fads6	-0.58		-0.38										K12419	Fatty acid desaturase 6
fah	0.53	0.51	0.70											Fumarylacetoacetase
faim						-0.49								
fam102a	-1.12	-0.44	-0.93			-0.92								
fam104a						0.37								
fam114a1						0.61								
fam118b	0.49													
fam120a	0.54		0.48											
fam124a											Loss	Loss		
fam126a	-0.32													Protein FAM126
fam135a						-0.32								
fam135b		0.42										Loss		
fam136a	0.65													
fam13a	-0.94	-0.37	-1.18											
fam160a1	-0.50													
fam160b1	-0.59		-0.49											
fam162a	-0.26													
fam167a						0.58								
fam167b						0.45								
fam168b						-0.89								
fam171a1						-0.76								
fam177a1	-0.43													
fam181b			-0.36											
fam184a						-0.75								
fam189b	0.47													
fam199x	-1.18	-0.46	-0.99											
fam207a	-0.37		-0.33			0.39								
fam20a				0.68									K21957	Pseudokinase FAM20A
fam210a				-0.41										
fam214a	-1.20	-0.93	-1.11											
fam214b	-1.03	-0.39	-0.39											
fam219a						-0.51								
fam222a	0.54									Gain				

fam222b						-0.55								
fam234b	0.38													
fam241a	-0.52													
fam32a						-0.44							K13120	Protein FAM32A
fam76b				0.33										
fam78a									Gain		Gain	Gain		
fam83c						-0.65								Protein FAM83
fam83d			0.60										K16805	Protein FAM83D
fam89a						-0.46								
fam91a1			0.29			-0.37								
fam92a	0.43												K23868	Protein FAM92
fam98b	0.94	0.38	0.41											Protein FAM98B
fanca	0.53		0.47										K10888	Fanconi anemia group A protein
fancb	0.52												K10889	Fanconi anemia group B protein
fancd2	0.99	0.84	0.81										K10891	Fanconi anemia group D2 protein
fance	-0.35	-0.35	-0.42										K10892	Fanconi anemia group E protein
fancf						-0.34							K10893	Fanconi anemia group F protein
fanci						0.58								Fanconi anemia group I protein
fastk			0.44			0.49							K08290	Fas-activated serine/threonine kinase
fastkd1	0.86													
fastkd3						0.42								
fastkd5				-0.42		-0.52								
fat1						-0.60								Protocadherin Fat 1/2/3
fat4						-0.69								Protocadherin Fat 4
fbl	0.62			-0.42									K14563	Rrna 2'-O-methyltransferase fibrillarin
fblim1						-0.50								Filamin-binding LIM protein 1
fbln5						0.58								Fibulin 5
fbn1						0.44								
fbrsl1						-0.51							K23214	Autism susceptibility gene 2 protein
fbxl12	-0.75	-0.37	-0.46	0.44		0.46								F-box and leucine-rich repeat protein 12
fbxl14	-0.38													F-box and leucine-rich repeat protein 14, F-box and leucine-rich repeat protein 2/20
fbxl15				-0.47		-0.42								F-box and leucine-rich repeat protein 15

fbxl17				0.44		0.74								F-box and leucine-rich repeat protein 17
fbxl18	0.49		0.54			-0.57							K10284	F-box and leucine-rich repeat protein 18
fbxl2	-0.95	-0.85	-0.97										K10268	F-box and leucine-rich repeat protein 2/20
fbxl20	-0.77	-0.49	-0.67											F-box and leucine-rich repeat protein 2/20
fbxl22				-0.42		-0.53						Loss		F-box and leucine-rich repeat protein 22
fbxl3			-0.52			0.65								F-box and leucine-rich repeat protein 3
fbxl4	-1.24	-0.78	-0.86										K10270	F-box and leucine-rich repeat protein 4
fbxl5	-0.63	-0.41	-0.72											F-box and leucine-rich repeat protein 5
fbxl6				0.42		0.42								F-box and leucine-rich repeat protein 6
fbxl7							Gain					Loss		F-box and leucine-rich repeat protein 2/20, F-box and leucine-rich repeat protein 7
fbxo16						-0.65								F-box protein 16
fbxo22						-0.41								F-box protein 22
fbxo25	-0.59	-0.39	-0.94										K10305	F-box protein 25/32
fbxo32	-3.77	-2.28	-4.35										K10305	F-box protein 25/32
fbxo33	-0.87		-0.64											F-box protein 33
fbxo40												Gain		F-box protein 40
fbxo41						-0.54								F-box protein 41
fbxo43						-0.53								F-box protein 43
fbxo46	-0.66	-0.30	-0.45											F-box protein 46
fbxo48	-1.82	-1.72	-1.82											
fbxo8	-0.33													F-box protein 8
fbxw4						0.46							K10262	F-box and WD-40 domain protein 4
fcer1g			-0.44			0.57							K07983	High affinity immunoglobulin epsilon receptor subunit gamma
fcf1	0.53			-0.43										U3 small nucleolar RNA-associated protein 24
fchsd2			-0.40										K20125	F-BAR and double SH3 domains protein
fcsk						0.52							K05305	Fucokinase
fdx1				-0.40		-0.64								Ferredoxin-1, mitochondrial
fdx2	0.53												K22071	Ferredoxin-2, mitochondrial
fdxacb1						0.54							K19307	25S rrna (uracil2634-N3)-methyltransferase
fdxr	0.61		0.50			0.48	Gain						K00528, K18914	Adrenodoxin-NADP+ reductase , ferredoxin/flavodoxin---NADP+ reductase
fen1	0.78	0.40	0.83											Flap endonuclease-1
fermt3						-0.65								Kindlin 3

fez2						0.58								Fasciculation and elongation protein zeta-2
fezf1												Loss		Fez family zinc finger protein, general transcription factor IIIA
fgd3	0.29	0.34										Loss	K05722	FYVE, rhogef and PH domain containing 3
fgf17		-0.53											K04358	Fibroblast growth factor
fgf22						-0.72					Gain		K04358	Fibroblast growth factor
fgf4						-0.84								Fibroblast growth factor
fgf5	0.74												K04358	Fibroblast growth factor
fgfbp1						-0.74							K25535	Fibroblast growth factor-binding protein
fgfr1op						0.32							K16546	FGFR1 oncogene partner
fgfr2						-1.24								Fibroblast growth factor receptor 2
fgfrl1												Gain	K26107	Fibroblast growth factor receptor-like 1
fhit	-0.63													Bis(5'-adenosyl)-triphosphatase
fhl2						-0.58							K14380	Four and a half LIM domains protein 2
fhl3	0.79	0.59	0.89											Four and a half LIM domains protein 3
fibp	-0.44	-0.32	-0.42											Acidic fibroblast growth factor intracellular-binding protein
fig4		-0.41											K22913	Phosphatidylinositol 3,5-bisphosphate 5-phosphatase
fitm2						0.56								
fkbp10	0.71	0.68	0.84			0.43								FK506-binding protein 9/10
fkbp11	0.74	0.45	1.10											FK506-binding protein 11
fkbp14						0.61								FK506-binding protein 14
fkbp2	0.55	0.37	0.60										K01802, K09569	FK506-binding protein 2 , peptidylprolyl isomerase
fkbp3	0.41			-0.55										FK506-binding protein 3
fkbp4	0.86												K09571	FK506-binding protein 4/5
fkbp6	0.77													FK506-binding protein 6
fkbp7	0.59	0.44	0.73											FK506-binding protein 7
fkbpl			-0.35											FK506-binding protein-like
fkrp				-0.58		-0.87							K19873	Fukutin-related protein
fktn		-0.32	-0.41										K19872	Fukutin
flcn	-1.62	-0.82	-1.57										K09594	Folliculin
fli1						0.68							K09436	Friend leukemia integration 1 transcription factor
flnb							Loss	Loss	Loss				K04437	Filamin
flt1						0.78								

flt3							Loss							Fms-related tyrosine kinase 3
flvcr1						0.74							K08220	MFS transporter, FLVCR family, feline leukemia virus subgroup C receptor-related protein
flvcr2	-0.47												K08220	MFS transporter, FLVCR family, feline leukemia virus subgroup C receptor-related protein
fnbp1	-0.47												K20121	Formin-binding protein 1
fnbp4				0.32										Formin-binding protein 4
fndc1											Loss	Loss	K24484	Fibronectin type III domain-containing protein 1
fndc3a						-0.41							K24485	Fibronectin type III domain-containing protein 3
fndc3b				-0.44		-0.98							K24485	Fibronectin type III domain-containing protein 3
fnip1	-0.56	-0.43	-1.00										K20400	Folliculin-interacting protein 1
fnta	0.32		0.31										K05955	Protein farnesyltransferase/geranylgeranyltransferase type-1 subunit alpha
foxd3	-0.42		-0.43	-0.49		-0.73							K09397	Forkhead box protein D
foxf2						0.58							K09399	Forkhead box protein F
foxk1	-0.37		-0.37			-0.76								Forkhead box protein K
foxm1			0.72										K09406	Forkhead box protein M
foxn2	-0.42	-0.31	-0.68											Forkhead box protein N
foxn3	-0.44		-0.33			-0.35							K09407	Forkhead box protein N
foxn4						-0.63								Forkhead box protein N
foxo3	-0.94		-0.73											Forkhead box protein O3
foxred1				-0.63								Gain	K18166	FAD-dependent oxidoreductase domain-containing protein 1
fpgt						-0.53							K00976	Fucose-1-phosphate guanylyltransferase
frem1						-0.95								FRAS1-related extracelluar matrix protein 1/2
frg1	-0.54	-0.34												Protein FRG1
frmd4a						0.65					Gain	Gain		FERM domain-containing protein 4
frmd8						0.75								FERM domain-containing protein 8
frmpd3								Loss						FERM and PDZ domain-containing protein 3
frrs1l	0.54													DOMON domain-contaning protein FRRS1L
frs2						0.53								Fibroblast growth factor receptor substrate 2
fry						0.72								
fryl				0.40										
fst						-0.78							K04661	Follistatin
fstl1						0.47							K23912	Follistatin-related protein 1

ftcdnl1	-0.44		-0.82											
fto						0.69								Mrna N6-methyladenine demethylase
ftsj3	0.56													Adomet-dependent rrna methyltransferase SPB1
fubp1	0.46	0.50				0.53								Far upstream element-binding protein
fut11						0.59							K11257	Galactoside 3-L-fucosyltransferase 11
fxn						-0.47								Frataxin
fyco1	-1.16		-0.79			-0.75							K21954	FYVE and coiled-coil domain-containing protein 1
fyn				0.34										
fzd3	0.46													Frizzled 3
fzd6						-0.56							K02376	Frizzled 6
fzd7				-0.46		-1.01							K02432	Frizzled 1/7
g3bp1	0.54												K17265, K24983	Ras gtpase-activating protein-binding protein 1 , Ras gtpase-activating protein-binding protein 2
g3bp2		0.26											K24983	Ras gtpase-activating protein-binding protein 2
g6pc3	0.31	0.46	0.48											Glucose-6-phosphatase
gab1			-0.30			-0.32							K09593	GRB2-associated-binding protein 1
gabarap	-1.21	-0.46	-1.16											GABA(A) receptor-associated protein
gabarapl2	-0.45		-0.48											GABA(A) receptor-associated protein
gabbr1				-0.41		-0.62								Gamma-aminobutyric acid type B receptor
gabrg2								Loss					K05186	Gamma-aminobutyric acid receptor subunit gamma
gadd45a	0.37					0.41							K04402	Growth arrest and DNA-damage-inducible protein
gadd45gip1	0.63		0.44											
gak			0.35										K08855	Cyclin G-associated kinase
gal3st1												Gain		Galactosylceramide sulfotransferase
gale	0.29													UDP-glucose 4-epimerase
galk1				0.47										Galactokinase
galk2						0.61								N-acetylgalactosamine kinase
galnt15	-0.66												K00710	Polypeptide N-acetylgalactosaminyltransferase
galnt18			0.43											Polypeptide N-acetylgalactosaminyltransferase
galnt2	0.57	0.55	0.62			0.42							K00710	Polypeptide N-acetylgalactosaminyltransferase
galnt5		0.36	0.66											Polypeptide N-acetylgalactosaminyltransferase
galnt7			0.51										K00710	Polypeptide N-acetylgalactosaminyltransferase
galnt9												Loss		Polypeptide N-acetylgalactosaminyltransferase

gamt	-0.50												K00542	Guanidinoacetate N-methyltransferase
ganab	0.49			-0.42		-0.59							K01187, K05546	Alpha-glucosidase , mannosyl-oligosaccharide alpha-1,3-glucosidase
gapdh				0.45									K00134	Glyceraldehyde 3-phosphate dehydrogenase (phosphorylating)
gar1	0.39			-0.36									K11128	H/ACA ribonucleoprotein complex subunit 1
garem2							Loss							
gas2	-0.40					0.45								Growth arrest-specific protein 2
gas6						-0.93							K05464	Growth arrest-specific 6
gatad1				-0.38									K23407	GATA zinc finger domain-containing protein 1
gatd1	-0.45													
gba	-0.57	-0.29	-0.43	0.42		0.36							K01201	Glucosylceramidase
gbe1				-0.44										1,4-alpha-glucan branching enzyme
gbf1	-0.34												K18443	Golgi-specific brefeldin A-resistance guanine nucleotide exchange factor 1
gck								Gain					K00844, K12407	Glucokinase , hexokinase
gclc						0.38								Glutamate--cysteine ligase catalytic subunit
gcn1				-0.40		-0.55								
gdap2	-0.39													Ganglioside-induced differentiation-associated protein 2
gdf3	0.77												K22672	Growth differentiation factor 3
gdf6							Loss	Loss	Loss	Loss	Loss	Loss		Growth differentiation factor 6
gdf9		-0.46	-0.47											Growth differentiation factor 9
gdpd1	-0.59	-0.38	-0.62	-0.48		-0.93							K22387	Lysophospholipase D
gemin4	0.40													Gem associated protein 4
gemin5	0.70					0.66								Gem associated protein 5
gemin7				-0.51		-0.76								Gem associated protein 7
gemin8	-0.42					-0.47								Gem associated protein 8
gen1	0.48													Holliday junction resolvase GEN1/YEN1
get1				-0.44										Tail-anchored protein insertion receptor
get3						-0.62							K01551	Arsenite/tail-anchored protein-transporting atpase
get4						0.55							K23387	Golgi to ER traffic protein 4
gfer	0.49												K17783	Mitochondrial FAD-linked sulfhydryl oxidase
gfm2				0.39		0.44							K02355	Elongation factor G
gfod2						-0.43								

ggact						-0.51								Gamma-glutamylaminecyclotransferase
ggcx			0.32	-0.37		-0.42								Vitamin K-dependent gamma-carboxylase
ggnbp2			0.31											
ggps1								Gain	Gain			Gain		Geranylgeranyl diphosphate synthase, type III
gigyf1				-0.35		-0.57							K18730	PERQ amino acid-rich with GYF domain-containing protein
gigyf2	0.55	0.45												PERQ amino acid-rich with GYF domain-containing protein
ginm1						0.32								
gins1	0.64	0.71	0.66											GINS complex subunit 1
gins2	0.77	0.84	0.87											GINS complex subunit 2
gins3	0.64	0.38	0.74	-0.40		-0.67								GINS complex subunit 3
gins4	0.67	0.57	0.61										K10735	GINS complex subunit 4
gipc1						-0.30							K20056	PDZ domain-containing protein GIPC
git1				0.46		0.38					Loss	Loss	K05737	G protein-coupled receptor kinase interactor 1
gja3						-0.69								Gap junction alpha-3 protein
gkap1						0.42								
gla	0.41					0.45				Gain	Gain	Gain	K01189, K07407	Alpha-galactosidase
glcci1						0.65								
gldc	-1.10		-0.61										K00281	Glycine dehydrogenase
glg1			0.31											Golgi apparatus protein 1
glis1						-0.85		Loss	Loss		Loss			Zinc finger protein GLIS1/3
glmp			-0.43											
glod5		-0.55												
glrb									Loss			Loss		Glycine receptor beta
glrx	-0.32													Glutaredoxin 3
glrx3						0.62								Monothiol glutaredoxin
glrx5	0.32												K07390	Monothiol glutaredoxin
glt1d1	-0.81					-0.58								
glt8d1								Gain			Gain			
gmcl1	-0.40		-0.46											BTB/POZ domain-containing protein 13 (germ cell-less protein-like 1)
gmnn	0.93	0.34	0.61										K10749	Geminin
gmppb			0.56											Mannose-1-phosphate guanylyltransferase
gmps	0.50	0.58	0.48	-0.38		-0.52							K01951	GMP synthase (glutamine-hydrolysing)

gna12						0.61							K04346	Guanine nucleotide-binding protein subunit alpha-12
gnaz						0.58								Guanine nucleotide-binding protein G(z) subunit alpha
gnb2				-0.37		-0.85							K04536, K04537	Guanine nucleotide-binding protein G(I)/G(S)/G(T) subunit beta-1, guanine nucleotide-binding protein G(I)/G(S)/G(T) subunit beta-2
gne				0.47										Bifunctional UDP-N-acetylglucosamine 2-epimerase / N-acetylmannosamine kinase
gng2		-0.36												Guanine nucleotide-binding protein G(I)/G(S)/G(O) subunit gamma-2
gnl1	-0.29		-0.47										K14539	Large subunit gtpase 1
gnl3	0.58													Nuclear GTP-binding protein
gnl3l				-0.40									K14538	Nuclear GTP-binding protein
gnmt	-0.69												K00552	Glycine N-methyltransferase
gns						-0.56								N-acetylglucosamine-6-sulfatase
golga3						-0.37								
golga7						-0.63								Golgin subfamily A member 7
golim4				-0.35		-0.54								Golgi integral membrane protein 4
golm1	0.49		0.28			0.47								
golph3						-0.53							K15620	Golgi phosphoprotein 3
gopc		0.40	0.48										K24054	Golgi-associated PDZ and coiled-coil motif-containing protein
gorab			0.55											RAB6-interacting golgin
gorasp1						-0.88								
gp1ba			-0.58										K06261	Platelet glycoprotein Ib alpha chain
gpalpp1	-0.27													
gpank1	-0.31							Loss			Gain	Gain		
gpat4	0.46		0.28								Gain	Gain		Glycerol-3-phosphate O-acyltransferase 3/4
gpatch11						-0.41								
gpatch2	-0.53	-0.43	-0.74				Gain		Gain			Gain + Loss		G patch domain-containing protein 2
gpatch2l	-1.96	-1.17	-1.89											G patch domain-containing protein 2
gpatch3	-0.40													
gpatch4	0.57		0.33											Pin2-interacting protein X1
gpcpd1			-0.64										K18695	Glycerophosphocholine phosphodiesterase GPCPD1
gpd2				0.56		0.74	Gain + Loss	Gain				Gain	K00111	Glycerol-3-phosphate dehydrogenase
gphn						-0.68								Gephyrin , molybdopterin molybdotransferase

gpn1	0.33					0.49								GPN-loop gtpase, U3 small nucleolar RNA-associated protein 15
gpn2		-0.36												GPN-loop gtpase
gpn3				-0.42									K24104	GPN-loop gtpase
gpr101				-0.44									K08423	G protein-coupled receptor 101
gpr12	0.51												K04315	G protein-coupled receptor 12
gpr137b						-0.70								Integral membrane protein GPR137
gpr139											Gain	Gain	K08428	G protein-coupled receptor 139
gpr141	-0.71	-0.41	-0.82			0.54							K08429	G protein-coupled receptor 141
gpr153				-0.46		-0.99								G protein-coupled receptor 153
gpr160	0.64													G protein-coupled receptor 160
gpr180		0.41	0.56											
gpr6									Loss					G protein-coupled receptor 6
gpx3				0.63										Glutathione peroxidase
gpx7	0.66	0.48	0.87											Glutathione peroxidase
gramd1c				0.43										
gramd2a						-0.50								
gramd4						0.59							K23554	GRAM domain-containing protein 4
grasp			-0.34			0.40							K24053	General receptor for phosphoinositides 1-associated scaffold protein
grem2				-0.41		-1.03	Loss					Loss		Gremlin
gria2						-0.72							K05198	Glutamate receptor 2
grin2a						0.63								Glutamate receptor ionotropic, NMDA 2A
grina	-0.44					-0.57							K24205	Protein lifeguard
grip1	0.52		0.44										K20251	Glutamate receptor-interacting protein
grip2								Loss			Gain	Gain + Loss	K20251	Glutamate receptor-interacting protein
grk3						-0.26								Beta-adrenergic-receptor kinase
grpel1	0.48													Molecular chaperone grpe
grpr								Gain			Gain	Gain	K04169	Gastrin-releasing peptide receptor
grsf1	0.49												K24984	G-rich sequence factor 1
grtp1						-0.51								TBC1 domain family member 6
grwd1	0.55					0.40							K14848	Ribosome assembly protein RRB1
gsap						0.65								
gskip			-0.38											

gstcd				-0.52										
gstk1	-0.47												K13299	Glutathione S-transferase kappa 1
gtdc1	0.49													
gtf2b				-0.40		-0.45								Transcription initiation factor TFIIB
gtf2e1	0.51												K03136	Transcription initiation factor TFIIE subunit alpha
gtf2f1			0.33										K03138	Transcription initiation factor TFIIF subunit alpha
gtf2f2	-0.36												K03139	Transcription initiation factor TFIIF subunit beta
gtf2h3						0.47								Transcription initiation factor TFIIH subunit 3
gtf3c1	0.43	0.49	0.41											General transcription factor 3C polypeptide 1
gtf3c2	0.33												K15200	General transcription factor 3C polypeptide 2
gtf3c3	0.42												K15201	General transcription factor 3C polypeptide 3 (transcription factor C subunit 4)
gtf3c6	0.43		0.58											General transcription factor 3C polypeptide 6
gtpbp2	-1.99	-0.71	-1.82				Gain	Gain						GTP-binding protein 2
gtpbp6						0.52							K03665	Gtpase
gtpbp8				-0.39									K03978	GTP-binding protein
gtse1			0.65											G-2 and S-phase expressed protein 1
gucd1						0.38								
gucy1b1		-0.42	-0.56										K12319	Guanylate cyclase soluble subunit beta
guf1						0.54								Translation factor GUF1, mitochondrial
guk1						-0.61							K00942	Guanylate kinase
gulp1						-0.71							K23285	PTB domain-containing engulfment adapter protein 1
gxylt2						-0.79							K13676	UDP-D-xylose:beta-D-glucoside alpha-1,3-D-xylosyltransferase
gaa						-0.55							K01187, K12316	Alpha-glucosidase , lysosomal alpha-glucosidase
hacd1	-0.55													Very-long-chain (3R)-3-hydroxyacyl-coa dehydratase
hacd4	-0.64												K10703	Very-long-chain (3R)-3-hydroxyacyl-coa dehydratase
hacl1				-0.44										2-hydroxyacyl-coa lyase
hadh	0.30													3-hydroxyacyl-coa dehydrogenase
hadha				-0.43		-0.56								Enoyl-coa hydratase / long-chain 3-hydroxyacyl-coa dehydrogenase
hadhb	0.34			-0.36										Acetyl-coa acyltransferase , acetyl-coa C-acetyltransferase
hamp	-0.56		-0.65											Hepcidin
haspin	0.41		0.52											Serine/threonine-protein kinase haspin

haus2	0.75		0.78											HAUS augmin-like complex subunit 2
haus4	0.56		0.68										K16587	HAUS augmin-like complex subunit 4
haus5	0.45		0.50											HAUS augmin-like complex subunit 5
haus8	0.38													HAUS augmin-like complex subunit 8
hax1			-0.31										K16220	HCLS1-associated protein X-1
hbp1	-1.66	-1.06	-1.56											HMG box transcription factor 1
hbs1l						0.35							K14416	Elongation factor 1 alpha-like protein
hck				0.49		0.49								
hdac10						0.57								Histone deacetylase 10
hdac2						-0.72								Histone deacetylase 1/2
hdac3	0.47		0.48										K11404	Histone deacetylase 3
hdac5	-0.88	-0.45	-0.67	0.43		0.39								
hddc2						0.60							K07023	5'-deoxynucleotidase
hdgfl2	0.63	0.58	0.62										K25065	Hepatoma-derived growth factor-related protein 2
hdhd3	-0.45													
hdlbp						0.50							K18666, K18756	Activating signal cointegrator complex subunit 1, protein bicaudal C
hdx				0.40		0.42							K24884	Highly divergent homeobox
heatr1	0.95	0.54	0.36										K14550	U3 small nucleolar RNA-associated protein 10
heatr3											Loss			HEAT repeat-containing protein 3
heatr5b						-0.89								HEAT repeat-containing protein 5
heatr6			0.41										K24815	HEAT repeat-containing protein 6
hebp1						-1.00								
heca	-1.49		-1.58										K23049	Headcase protein
hectd4	0.36		0.39											E3 ubiquitin-protein ligase HECTD4
hecw1											Loss	Loss		E3 ubiquitin-protein ligase HECW1
hells	1.40	1.15	1.26											ATP-dependent DNA helicase
helq	-0.39			0.58		0.44								POLQ-like helicase
hemk1	-0.58		-0.88			0.54								Release factor glutamine methyltransferase
henmt1		0.12	0.22											Small RNA 2'-O-methyltransferase
hepacam2												Loss		
herc3	-0.57	-0.53	-0.54	0.55										E3 ubiquitin-protein ligase HERC3
herc4	-0.41		-0.36											E3 ubiquitin-protein ligase HERC4

herpud1						0.54								Homocysteine-responsive endoplasmic reticulum-resident ubiquitin-like domain member 1 protein
herpud2	-0.34		-0.31											Homocysteine-responsive endoplasmic reticulum-resident ubiquitin-like domain member 1 protein
hgs	-0.28					-0.27								Hepatocyte growth factor-regulated tyrosine kinase substrate
hgsnat	-0.36					-0.36							K10532	Heparan-alpha-glucosaminide N-acetyltransferase
hhipl1						-1.00							K25557	HHIP-like protein
hic1						0.51								Hypermethylated in cancer protein
hif1a				0.44										Hypoxia-inducible factor 1 alpha
hikeshi						0.46								Protein Hikeshi
hinfp			0.38										K25794	Histone H4 transcription factor
hint3			-0.34			-0.39								
hip1						0.40								Huntingtin interacting protein 1
hivep1	-0.41					-0.68							K09239	Human immunodeficiency virus type I enhancer-binding protein
hivep2						0.52								Human immunodeficiency virus type I enhancer-binding protein
hlf	-1.84	-0.43	-0.69			-0.63								Hepatic leukemia factor
hltf				-0.42		-0.63							K15711	SWI/SNF-related matrix-associated actin-dependent regulator of chromatin subfamily A3
hmbs	0.68	0.39	0.44										K01749	Hydroxymethylbilane synthase
hmcn1						-0.46								Hemicentin
hmg20a						-0.70								High mobility group protein 20A
hmga1	0.47		0.42										K09282	High mobility group AT-hook protein 1
hmga2						-0.55							K09283	High mobility group AT-hook protein 2
hmgb2	0.30		0.39											
hmgxb4						-0.33								HMG domain-containing protein 4
hmox2				0.33										Heme oxygenase (biliverdin-producing, ferredoxin) , heme oxygenase 2
hnf1a				0.42	0.99			Gain	Gain		Gain	Gain + Loss		Transcription factor 1, hepatocyte nuclear factor 1-alpha
hnrnpa0	0.62	0.38	0.60											Heterogeneous nuclear ribonucleoprotein A0, heterogeneous nuclear ribonucleoprotein A1/A3
hnrnpab	0.36	0.30	0.33	-0.29								Gain	K13044	Heterogeneous nuclear ribonucleoprotein A/B/D
hnrnpd						0.72							K13044	Heterogeneous nuclear ribonucleoprotein A/B/D
hnrnph3				0.39									K12898	Heterogeneous nuclear ribonucleoprotein F/H
hnrnpk			0.31											Heterogeneous nuclear ribonucleoprotein K
hnrnpll							Gain + Loss	Gain	Gain		Gain + Loss	Gain + Loss	K13159	Heterogeneous nuclear ribonucleoprotein L

hnrnpr				-0.35		-0.47								Heterogeneous nuclear ribonucleoprotein R
hnrnpu	0.60	0.35	0.52			0.40							K12888	Heterogeneous nuclear ribonucleoprotein U
hnrnpul1			0.38	0.30									K15047	Heterogeneous nuclear ribonucleoprotein U-like protein 1
homer1			-0.43											Homer
homer2	-0.37												K15010	Homer
hook2						-0.63							K16611	Protein HOOK2
hook3						-0.68							K16536	Protein HOOK3
hoxa11						-0.44							K21951	Homeobox protein Hox-A11
hoxa13						-0.67							K09298	Homeobox protein hoxa/B/C/D13
hoxa3				0.42									K09303	Homeobox protein hoxa/B/D3
hoxb1						-0.98							K09301	Homeobox protein hoxa/B/D1
hoxb5						-0.99							K09305	Homeobox protein hoxa/B/C5
hoxb7	-0.55					-0.65							K09307	Homeobox protein hoxa/B7
hoxb8						-1.01							K09308	Homeobox protein hoxb/C/D8
hoxc13						-0.60							K09298	Homeobox protein hoxa/B/C/D13
hoxc9						-0.88								Homeobox protein hoxb/C/D9
hoxd3						-0.70							K09303	Homeobox protein hoxa/B/D3
hp1bp3	0.39	0.33	0.50			0.32							K11275	Histone H1/5
hpdl	1.13		0.72										K24788	4-hydroxyphenylpyruvate dioxygenase-like protein
hprt1				-0.41										Hypoxanthine phosphoribosyltransferase
hps1	-0.66													Hermansky-Pudlak syndrome 1 protein
hps5	-0.55		-0.49										K20191	Hermansky-Pudlak syndrome 5 protein
hs1bp3	-0.45		-0.65										K24094	HCLS1-binding protein 3
hs2st1				-0.48		-0.72								Heparan sulfate 2-O-sulfotransferase HS2ST1
hs3st1				-0.43		-1.00							K01024	
hsd11b2		-0.40												3-hydroxybutyrate dehydrogenase , corticosteroid 11-beta-dehydrogenase isozyme 2
hsdl1						0.56								
hsdl2	0.34			-0.49		-0.62								Citronellol/citronellal dehydrogenase
hsf2						0.53								Heat shock transcription factor 2
hspa13						0.52							K09491	Stress 70 protein chaperone microsome-associated 60kda protein
hspa14						0.50								
hspa4						0.46							K09489	Heat shock 70kda protein 4

hspa4l	0.63												K09485	Heat shock protein 110kda
hspa5	1.39	0.57	1.08											Endoplasmic reticulum chaperone bip
hspa9	0.80													Molecular chaperone dnak
hspb6						-0.51								
hspb7			-0.45										K09546	Heat shock protein beta-7
hspb8			-0.56	-0.64		-1.15								Heat shock protein beta-8
hspbap1	-0.46		-0.44										K19375	HSPB1-associated protein 1
hspbp1	0.67										Gain		K09562	Hsp70-interacting protein
hspd1	1.25	0.65	0.60											Chaperonin groel
hspg2						0.53								Basement membrane-specific heparan sulfate proteoglycan core protein
huwe1	0.34		0.30											E3 ubiquitin-protein ligase HUWE1
hvcn1						0.54							K22644	Voltage-gated hydrogen channel 1
hyi			0.40										K01816	Hydroxypyruvate isomerase
hyls1	-0.54	-0.43											K16472	Hydrolethalus syndrome protein 1
hyou1	0.93	0.83	0.79			0.66							K09486	Hypoxia up-regulated 1
iars2	0.45													Isoleucyl-trna synthetase
iba57	0.49													Transferase CAF17, mitochondrial
ibtk	0.33					0.32							K24810	Inhibitor of Bruton tyrosine kinase
ice1	0.31												K26143	Little elongation complex subunit 1
ice2						0.41								Little elongation complex subunit 2
ick			-0.43			-0.51							K08828, K08829	Intestinal cell (MAK-like) kinase , male germ cell-associated kinase
icmt						0.47								Protein-S-isoprenylcysteine O-methyltransferase
ide	0.60													Insulysin
idh3b	0.61			-0.63		-0.38							K00030	Isocitrate dehydrogenase (NAD+)
idh3g	0.27													Isocitrate dehydrogenase (NAD+)
idua				0.42									K01217	L-iduronidase
iffo1	0.54													
iffo2				0.40		0.87								
ift20				-0.57		-0.37								Intraflagellar transport protein 20
ift22						0.46								Intraflagellar transport protein 22
ift27				-0.41									K07934	Intraflagellar transport protein 27
ift52	-0.61	-0.44	-0.50			-0.57								Intraflagellar transport protein 52

ift57	0.60												K04638	Intraflagellar transport protein 57
ift74						0.44								Intraflagellar transport protein 74
ift81	0.43					0.45							K19677	Intraflagellar transport protein 81
igf1						0.59							K05459	Insulin-like growth factor 1
igf2r						0.53								Insulin-like growth factor 2 receptor
igfbp4						-0.88							K23576	Insulin-like growth factor-binding protein 4
igfbp7						-0.86								Insulin-like growth factor-binding protein 7
igsf10				-0.37		-0.63							K24400	Matrix remodeling-associated protein 5
igsf11			-0.40			-0.61								Immunoglobulin superfamily, member 11
igsf8				0.50										Immunoglobulin superfamily, member 8
ikbkg	-0.37		-0.30										K07210	Inhibitor of nuclear factor kappa-B kinase subunit gamma
il10						0.61							K05443	Interleukin 10
il16												Loss		Interleukin 16
il17ra							Loss					Gain	K05164	Interleukin 17 receptor A
ilf2	0.45												K13089	Interleukin enhancer-binding factor 2
ilf3	0.47		0.31										K13090	Interleukin enhancer-binding factor 3
ilkap	-1.03	-0.74	-0.96										K17500	Integrin-linked kinase-associated serine/threonine phosphatase 2C
ilrun	-0.50		-0.58											Next to BRCA1 gene 1 protein
ilvbl						-0.54								2-hydroxyacyl-coa lyase
immp2l	0.40												K09648	Mitochondrial inner membrane protease subunit 2
imp3	0.31												K14560	U3 small nucleolar ribonucleoprotein protein IMP3
imp4	0.33					0.30							K14561	U3 small nucleolar ribonucleoprotein protein IMP4
impad1	0.35	0.36	0.41										K15759	Golgi-resident PAP phosphatase
ina	1.26	0.47	0.69											
incenp	0.40		0.70										K11515	Inner centromere protein
inf2	-0.62	-0.35	-0.61										K23958	Inverted formin-2
ing4			-0.38										K11346	Inhibitor of growth protein 4
ing5						-0.47								Inhibitor of growth protein 4, inhibitor of growth protein 5
inka2						-0.47								
ino80						-0.65								Chromatin-remodeling atpase INO80
ino80b			0.45			0.59								INO80 complex subunit B
ino80d	-0.39		-0.40			-0.43							K11668	INO80 complex subunit D

inpp1						-0.33							K01107	Inositol polyphosphate 1-phosphatase
inpp4b			0.29			-0.27							K01109	Inositol polyphosphate-4-phosphatase
inpp5b	0.25		0.21										K01099	Inositol polyphosphate 5-phosphatase INPP5B/F
inppl1						0.28								Phosphatidylinositol-3,4,5-trisphosphate 5-phosphatase 2
insc	-0.41					0.64								
insig2	0.43	0.42	0.45											
insyn1						-0.56								
ints10	0.75	0.50	0.72										K13147	Integrator complex subunit 10
ints12	0.51		0.37											Integrator complex subunit 12
ints2	0.71	0.38	0.84										K13139	Integrator complex subunit 2
ints3												Gain		Integrator complex subunit 3
ints6						-0.43							K13143	Integrator complex subunit 6
ints9	0.43		0.48											Integrator complex subunit 9
ipo4	0.80	0.61	0.44										K20221	Importin-4
ipo5						0.49								Importin-5
ipo7			-0.42											Importin-7
ipo8			0.52											Importin-7, importin-8
ipo9	0.60	0.46	0.43											Importin-9
ipp						0.60							K13956	Actin-binding protein IPP
ippk						0.32								Inositol-pentakisphosphate 2-kinase
iqgap2	-0.44												K05767	Ras gtpase-activating-like protein IQGAP2/3
irak1bp1						0.66								
irf2				0.43		0.55							K10153	Interferon regulatory factor 2
irf2bp2						-0.33							K22383	Interferon regulatory factor 2-binding protein
irf8						0.48							K10155	Interferon regulatory factor 8
irf9	-1.31	-0.98	-1.30											Interferon regulatory factor 9
irx3						-0.47							K24889	Iroquois-class homeodomain protein IRX
isca1			-0.38	-0.49		-0.85							K22063	Iron-sulfur cluster assembly 1
ism1						-0.74							K24435	Isthmin
isoc2	-0.43													
ist1	-0.44													Vacuolar protein sorting-associated protein IST1
itfg1	-0.35													Integrin alpha FG-GAP repeat containing protein 1
itga2						-0.57								Integrin alpha 2

itga5						0.71								Integrin alpha 5
itga6	0.40													Integrin alpha 6
itga7						0.58							K06583	Integrin alpha 7
itga8				-0.37		-0.66							K06584	Integrin alpha 8
itgb1				-0.40		-0.60							K05719	Integrin beta 1
itgb6											Gain			Integrin beta 6
itgb8			-0.55			-0.45							K06591	Integrin beta 8
itm2a						0.78							K18241	Integral membrane protein 2A
itm2b						-0.69							K18264	Integral membrane protein 2B
itpk1								Loss		Gain	Loss		K00913	Inositol-1,3,4-trisphosphate 5/6-kinase / inositol-tetrakisphosphate 1-kinase
itpkb	-0.42	-0.36	-0.43										K00911	1D-myo-inositol-triphosphate 3-kinase
itprid2				0.45										
itsn1				0.38		0.49								Intersectin
ivd				-0.38		-0.46							K00249, K00253	Acyl-coa dehydrogenase , isovaleryl-coa dehydrogenase
iyd							Gain	Gain	Gain		Gain	Gain		Iodotyrosine deiodinase
izumo1												Gain	K25529	Izumo sperm-egg fusion protein 1
jade1	-0.90		-0.69								Loss	Loss		Protein Jade-1
jade2	-0.50													E3 ubiquitin-protein ligase Jade-2
jade3	0.38		0.40											Protein Jade-3
jag1						-0.54								Jagged-1
jak1						0.58							K11217	Janus kinase 1
jam3				-0.51										Junctional adhesion molecule 3
jarid2			0.32										K11478	Protein Jumonji
jcad	-0.50		-0.39			-0.81								
jdp2						-0.65							K09033	Jun dimerization protein 2
jmjd1c						-0.30							K11449	Jumonji domain-containing protein 1C
jmjd4	-1.07	-0.49	-0.75											Histone arginine demethylase JMJD6
jmjd6						-0.51							K11323	Histone arginine demethylase JMJD6
jmy	-1.91	-0.65	-1.97										K24045	Junction-mediating and -regulatory protein
josd2	-0.42		-0.38										K15235	Josephin
jph2				-0.52		-0.41							K19530	Junctophilin
jpt1			0.46											

jpt2	0.44		0.56											
jtb	-0.46	-0.44	-0.47											
kalrn				0.41									K15048	Kalirin
kank3	-0.47	-0.37									Loss		K22808	KN motif and ankyrin repeat domain-containing protein
kank4						0.65							K22808	KN motif and ankyrin repeat domain-containing protein
kat2a	0.62	0.40	0.42										K06062	Histone acetyltransferase
kat5						-0.63								Histone acetyltransferase HTATIP
kat6a			-0.30			-0.31							K11305	Histone acetyltransferase MYST3
kat6b	-0.72			-0.46									K11306	Histone acetyltransferase MYST4
katna1		-0.40											K07767	Katanin p60 atpase-containing subunit A1
katnb1	-0.32													Katanin p80 WD40 repeat-containing subunit B1
katnbl1			0.45			0.61								
kbtbd12	-1.94	-0.88	-1.81											Kelch repeat and BTB domain-containing protein 12
kbtbd4				-0.38		-0.33							K10472	Kelch repeat and BTB domain-containing protein 4
kbtbd8	-0.65	-0.39	-0.79										K10475	Kelch repeat and BTB domain-containing protein 8
kcnab1						-0.51							K04882, K04883	Potassium voltage-gated channel Shaker-related subfamily A, beta member 1, potassium voltage-gated channel Shaker-related subfamily A, beta member 2
kcnb2					0.86									
kcnd2	-0.73			-0.42		-0.94								
kcng3							Loss	Loss			Gain			Potassium voltage-gated channel subfamily G member 3
kcnj3			-0.55										K04997	Potassium inwardly-rectifying channel subfamily J member 3
kcnk1						-0.46								Potassium channel subfamily K member 1
kcnk18						0.69							K20007	Potassium channel subfamily K member 18
kcnn4		-0.65												Potassium intermediate/small conductance calcium-activated channel subfamily N member 4
kcp						0.79								Kielin/chordin-like protein
kctd2						-0.36								BTB/POZ domain-containing protein KCTD2/5/17
kctd6						-0.35							K21916	BTB/POZ domain-containing protein KCTD6
kdf1						-0.58							K23346	Keratinocyte differentiation factor 1
kdm1a						-0.45							K11450	
kdm1b	0.43					0.38							K19413	Lysine-specific histone demethylase 1B
kdm2a						-0.26								F-box and leucine-rich repeat protein 10/11
kdm3b	-0.54													

kdm4a	-0.56												K06709	
kdm6a						-0.36								Lysine-specific demethylase 6A
kdm6b						-0.46								Lysine-specific demethylase 6B
kdm8											Gain		K10277	
kdsr			-0.28										K04708	3-dehydrosphinganine reductase
keap1	-0.43	-0.59	-0.78			-0.54							K10456	Kelch-like protein 19
kera			0.45			-0.59							K08123	Keratocan
khdc4						0.58							K25780	Kelch domain-containing protein 4
khdrbs2								Loss	Gain		Gain	Gain		KH domain-containing, RNA-binding, signal transduction-associated protein 2
khsrp	0.36		0.29	-0.35		-0.37							K13210	Far upstream element-binding protein
kidins220	-0.39		-0.36											Ankyrin repeat-rich membrane spanning protein
kif11			0.57										K10398	Kinesin family member 11
kif15						0.49							K10400	Kinesin family member 15
kif18a	0.61		0.72											Kinesin family member 18A
kif1c	-0.35													Kinesin family member 1
kif20a			0.70											Kinesin family member 20
kif20b			0.53											Kinesin family member 20
kif21a						0.53							K24185	Kinesin family member 21
kif22	0.52		0.56											Kinesin family member 22
kif23			0.48										K17387	Kinesin family member 23
kif26a	0.46	0.39	0.43			-0.45								Kinesin family member 26
kif27						-0.66								
kif3a						-0.39							K10394	Kinesin family member 3A
kif4a	0.62	0.46	1.15										K10395	Kinesin family member 4
kif9							Loss	Loss			Loss	Loss		Kinesin family member 6/9
kirrel2	0.47					-0.36								Kirrel/Neph family protein
kirrel3						-0.84								Kirrel/Neph family protein
kiaa0040	-0.51													
kiaa0355						-0.31								
kiaa0825											Gain			Protein KIAA0825
kiaa0895l						-0.55	Gain							
kiaa1109						-0.65								Transmembrane protein KIAA1109

kiaa1841	0.55													
klc1	-0.26												K10407	Kinesin light chain
klf15	-0.82		-1.04										K09210	Krueppel-like factor 15
klf2						-0.49								Krueppel-like factor 2
klf5						-0.72							K09206	Krueppel-like factor 5
klf9	-1.30	-0.48	-1.77										K09208	Krueppel-like factor 9/13/14/16
klhdc1	-1.45	-0.43	-1.39											Kelch domain-containing protein 1
klhdc2	-0.50		-0.39											Kelch domain-containing protein 2
klhdc3	0.43												K25808	Kelch domain-containing protein 3
klhdc4	0.49													
klhl12	-0.54	-0.46	-0.76											Kelch-like protein 12
klhl15	0.34												K10452	Kelch-like protein 15
klhl21						0.75								Kelch-like protein 21
klhl22	-1.05	-0.74	-1.04											Kelch-like protein 22
klhl23	-0.76		-0.79										K10460, K11090	Kelch-like protein 23, lupus La protein
klhl24	-1.12		-0.69										K10461, K24539	Kelch-like protein 24/35, YEATS domain-contaning protein 2
klhl28			-0.35											Kelch-like protein 28
klhl30			-0.51			-0.58							K10466	Kelch-like protein 30
klhl31	-0.74		-0.65											Kelch-like protein 31
klhl6			-0.63										K10444	Kelch-like protein 6
kmt2d						0.72								
kmt5a						-0.61							K11428	
kndc1			-0.53			0.83								
knop1	0.85	0.40												
kntc1	0.49		0.53										K11577	Kinetochore-associated protein 1
kpna1	0.43	0.37	0.51										K15042, K23940	Importin subunit alpha-5, importin subunit alpha-6/7
kpna2	0.60		0.53			-0.43							K15043	Importin subunit alpha-1/8
kpna4						0.61								Importin subunit alpha-4/3
kpna5	-0.42		-0.49										K15042	Importin subunit alpha-6/7
kpnb1	0.76	0.55	0.66											Importin subunit beta-1
kri1	0.45	0.49												Protein KRI1

krr1						0.42								Ribosomal RNA assembly protein
krt222											Loss			
krtcap2	0.30	0.31	0.52											
ksr1			0.44											Kinase suppressor of Ras 1
kti12				-0.45									K15456	Protein KTI12
kyat1	0.42												K00816	Kynurenine---oxoglutarate transaminase / cysteine-S-conjugate beta-lyase / glutamine---phenylpyruvate transaminase
l3mbtl4				0.62									K24387	Lethal(3)malignant brain tumor-like protein
lactb	-0.40												K17382	Serine beta-lactamase-like protein LACTB, mitochondrial
lama2			-0.33											Laminin, alpha 1/2
lama5						-0.63								Laminin, alpha 3/5
lamb3						-0.78								Laminin, beta 3
lamb4						-0.90								Laminin, beta 4
lamc1								Gain			Gain	Gain		Laminin, gamma 1
lamp1	-0.50													Lysosomal-associated membrane protein 1/2
lamp2	-0.26													Lysosomal-associated membrane protein 1/2
lamtor1	-0.37		-0.53											Ragulator complex protein LAMTOR1
lamtor2	-0.29												K20398	Ragulator complex protein LAMTOR2
lamtor3	-0.42	-0.38										Gain + Loss	K04370	Ragulator complex protein LAMTOR3
lamtor4	-0.54												K20399	Ragulator complex protein LAMTOR4
lancl2	0.43													Glutathione transferase
lancl3				-0.45		-0.68								
lap3	0.65												K01255, K11142	Cytosol aminopeptidase , leucyl aminopeptidase
laptm4b	1.17												K12387	Lysosomal-associated transmembrane protein
larp1						-0.61								La-related protein 1
larp4	0.83	0.36	0.42	-0.39		-0.65							K18763	La-related protein 4
lars2	0.61													Leucyl-trna synthetase
las1l	0.50			-0.58		-0.56								Ribosomal biogenesis protein LAS1
lats2	-0.65	-0.39	-0.53										K08791	Serine/threonine-protein kinase LATS1/2
layn							Loss							Layilin
lck							Loss		Loss		Loss	Loss		Lymphocyte cell-specific protein tyrosine kinase
lcmt1	0.73												K18203	

lcorl				-0.35		-0.53								
ldah		0.32												
ldhd	0.48			0.38										D-lactate dehydrogenase (cytochrome)
ldlr		-0.83	-0.50											Low-density lipoprotein receptor
ldlrad3				-0.48		-0.78								
lef1						-0.48							K04492	Lymphoid enhancer-binding factor 1
lemd3			0.33			0.39								Inner nuclear membrane protein Man1
leo1	0.35		0.34											RNA polymerase-associated protein LEO1
lepr						0.63							K05062	Leptin receptor
letm1						0.34								LETM1 and EF-hand domain-containing protein 1, mitochondrial
letm2						-0.28								LETM1 and EF-hand domain-containing protein 1, mitochondrial
letmd1				-0.42		-0.51								LETM1 and EF-hand domain-containing protein 1, mitochondrial
lfng						0.50								Fringe
lgmn	-0.96	-0.53	-0.73								Loss	Loss		Legumain
lgr4						-0.46							K04309	Leucine-rich repeat-containing G protein-coupled receptor 4
lgr6	-0.60			-0.42		-1.08								
lhfpl2	0.39													LHFPL tetraspan subfamily member protein
lhfpl6						-0.64								LHFPL tetraspan subfamily member protein
lig1	0.92	0.75	0.99										K10747	DNA ligase 1
limch1												Loss	K24422	LIM and calponin homology domains-containing protein 1
limd1						-0.88								LIM domain-containing protein
limd2						-0.58								LIM domain and actin-binding protein 1
limk2						-0.80								LIM domain kinase 2
lin37	-0.30	-0.32												Protein lin-37
lin7a						-0.47								Protein lin-7
lin7c						-0.38								Protein lin-7
lin9						-0.33								Protein lin-9
lingo3	-0.49		-0.54											Leucine-rich repeat and immunoglobulin-like domain-containing nogo receptor-interacting protein
lins1	0.58					-0.58								Protein Lines
lipe						0.51								Hormone-sensitive lipase
lipt2			0.43	-0.59		-0.72							K03801, K23735	Lipoyl(octanoyl) transferase , lipoyl(octanoyl) transferase 2
lix1l				-0.54		-1.25								LIX1-like protein

llgl2						-0.85								Lethal(2) giant larvae protein
lman1	0.55	0.47	0.77											Lectin, mannose-binding 1
lman2						0.39								Lectin, mannose-binding 2
lmbrd1	-0.41					-0.40								LMBR1 domain-containing protein 1
lmf2	0.62					0.58								Lipase maturation factor 1
lmln						0.74								Leishmanolysin-like peptidase
lmnb1	0.74		0.68											Lamin B
lmnb2	0.52	0.56	0.51											Lamin B
lmo2						-0.49							K15612	Rhombotin-2
lmo7	-0.58	-0.30	-0.40	0.43		0.55							K06084	F-box protein 20
lmod1	-0.62		-0.49										K22030	Leiomodin
lmtk2	-0.45					-0.58								Lemur tyrosine kinase 2
lnp1								Gain				Gain + Loss		
LOC115528736						0.50								
LOC115528746						0.98								
LOC115528759				-0.58										
LOC115528786				-0.41		-0.48							K16197, K16198	14-3-3 protein beta/theta/zeta, 14-3-3 protein gamma/eta
LOC115528788						0.74								
LOC115528806				0.58										
LOC115528824	-0.80	-0.38	-0.77											Serine/threonine-protein kinase ULK2
LOC115528830	-0.34													Latent transforming growth factor beta binding protein 2/3/4
LOC115528831	0.31													Arf-GAP with coiled-coil, ANK repeat and PH domain-containing protein
LOC115528836						-0.66							K01415	Endothelin-converting enzyme
LOC115528839												Gain	K09191	General transcription factor IIIA
LOC115528848						0.56								
LOC115528863	-1.55												K02270	Cytochrome c oxidase subunit 7a
LOC115528870						-1.68								
LOC115528872						0.71								
LOC115528875						-0.53							K24363	Seizure protein 6
LOC115528884			0.73										K02176	Secreted frizzled-related protein 2
LOC115528887	-0.52													High affinity cgmp-specific 3',5'-cyclic phosphodiesterase 9

LOC115528890							Loss							Olfactory receptor
LOC115528901	-0.80												K04285, K04286	Melatonin receptor type 1A, melatonin receptor type 1B
LOC115528906											Gain	Gain	K08798	MAP/microtubule affinity-regulating kinase
LOC115528907						-0.77							K09191, K09228	General transcription factor IIIA, KRAB domain-containing zinc finger protein
LOC115528922												Loss		
LOC115528926						-0.69					Loss	Loss	K06087	Claudin
LOC115528936											Gain	Gain		
LOC115528945	-1.07					-0.67								Centrosomal protein CEP57
LOC115528950	-0.55		-0.49										K07880	Ras-related protein Rab-4B
LOC115528954				0.51									K09635	Transmembrane protease serine 4
LOC115528957	0.72									Loss	Loss	Loss		Ras homolog gene family, member U
LOC115528959	-2.61													
LOC115528967						-0.49								Barh-like homeobox
LOC115528978						-0.89								Integrin-linked kinase
LOC115528979						0.59								Cell adhesion molecule 1
LOC115529017	0.47	0.46	0.42			0.30							K11093	U1 small nuclear ribonucleoprotein 70kda
LOC115529041	0.38	0.23	0.70	0.34		0.67								
LOC115529043						0.55								
LOC115529057	-0.91	-0.62	-0.63											7SK snrna methylphosphate capping enzyme
LOC115529064												Gain		Endonuclease domain-containing 1 protein
LOC115529069	0.50					0.52							K20029	Palmitoyltransferase ZDHHC3/7/25
LOC115529070	-0.83	-0.61	-0.82										K13084	Phosphatidylinositol-4,5-bisphosphate 4-phosphatase
LOC115529082	-3.26	-3.01	-4.12			-0.97							K13781	Solute carrier family 7 (L-type amino acid transporter), member 8
LOC115529085						0.62								Ubiquitin-like protein Nedd8
LOC115529090		0.79												
LOC115529099	-0.66		-0.58			0.61								
LOC115529105						0.55								
LOC115529117	0.55					-0.55								
LOC115529120	-0.81	-0.60	-0.80	0.41								Loss		Ribonuclease ZC3H12
LOC115529122						-0.60								
LOC115529123				0.42		0.68								Receptor-interacting serine/threonine-protein kinase 3
LOC115529132						0.34								

LOC115529142				0.81		0.69							K01109	Inositol polyphosphate-4-phosphatase
LOC115529149				-0.50		-0.65								Coronin-2
LOC115529154												Loss	K09223	Growth factor independent 1
LOC115529156	-0.78		-0.75											E3 ubiquitin-protein ligase RNF130
LOC115529157	-1.59												K17505	Protein phosphatase 1K
LOC115529161				0.42		0.58								Arfaptin
LOC115529163			0.32											
LOC115529166	0.33		0.44											ATP-dependent RNA helicase DDX39 , ATP-dependent RNA helicase UAP56/SUB2
LOC115529167						0.40								
LOC115529168			0.51											
LOC115529174				0.41										NLR family CARD domain-containing protein 3
LOC115529176											Loss			
LOC115529178			-0.47											
LOC115529184												Loss		Insulin-like growth factor-binding protein 10
LOC115529187							Gain					Gain + Loss		
LOC115529206								Gain				Gain	K22527	Cbp/p300-interacting transactivator 1
LOC115529207	0.61													
LOC115529208									Loss				K25573	EH domain-binding protein 1-like protein 1
LOC115529220	-0.64										Gain			
LOC115529222						-0.62								
LOC115529226						-0.48								
LOC115529229								Gain	Gain			Gain		
LOC115529239						1.47								
LOC115529253						0.58							K04812	Nicotinic acetylcholine receptor beta-1
LOC115529255						-0.53							K04812	Nicotinic acetylcholine receptor beta-1
LOC115529258											Gain			G protein-coupled receptor 132
LOC115529261									Loss					
LOC115529263	-1.03	-0.74	-1.24											
LOC115529265	-0.66	-0.34	-0.68											
LOC115529267				0.66										
LOC115529275			-0.52											MHC class I antigen

LOC115529287								Gain			Gain	Gain + Loss		Nuclease HARBI1
LOC115529292		-0.40												Small subunit ribosomal protein s11e
LOC115529293						2.31								NLR family CARD domain-containing protein 3
LOC115529305	-0.66		-0.48											
LOC115529306						-0.72								Interferon receptor 2
LOC115529308						0.75								
LOC115529313				0.33										
LOC115529317				0.47		0.87								
LOC115529320									Gain		Gain	Gain	K11964	Pellino
LOC115529323						0.63								MHC class I antigen
LOC115529332				0.36										Mitogen-activated protein kinase kinase kinase 14
LOC115529351	-0.56												K21944	Period circadian protein 1
LOC115529353						-0.68								Procollagen C-endopeptidase enhancer
LOC115529357		-0.39												
LOC115529359						0.60								
LOC115529361												Gain		Zonadhesin
LOC115529367	-0.69					-0.67						Gain		
LOC115529374	-0.98			-0.79		-1.00								
LOC115529378							Loss						K24038	Docking protein 7
LOC115529379	-0.51													Kelch-like protein 33
LOC115529380						-1.00							K06843	Netrin 1
LOC115529381						0.74							K09191, K09228	General transcription factor IIIA, KRAB domain-containing zinc finger protein
LOC115529383											Loss		K06521	Semaphorin 4
LOC115529385						-0.55							K06691, K20456	26S proteasome regulatory subunit N13, oxysterol-binding protein 1
LOC115529390											Gain	Gain		Carboxypeptidase Z
LOC115529392		0.36	0.36										K13646	Lysyl hydroxylase/galactosyltransferase/glucosyltransferase
LOC115529402						-0.50								
LOC115529403			0.46			0.54							K08593	Sentrin-specific protease 3
LOC115529407											Gain	Gain	K03334	L-amino-acid oxidase
LOC115529408						0.59								
LOC115529410						0.62	Loss	Loss					K09677	Galactose-3-O-sulfotransferase 4

LOC115529413											Gain + Loss	Gain + Loss	K03334	L-amino-acid oxidase
LOC115529421						-0.74								
LOC115529424				2.17		1.77								
LOC115529431	-0.89					-0.77								Galactose-3-O-sulfotransferase 4
LOC115529436											Loss	Loss		Leukotriene B4 receptor 1
LOC115529437						0.39							K03257	Translation initiation factor 4A
LOC115529444						-0.35							K18266	Protein NDRG1
LOC115529445						0.55								MHC class I antigen
LOC115529450	0.66		0.50										K15445	Trna (guanine9-N1)-methyltransferase
LOC115529455	-0.67	-0.37	-0.52											
LOC115529458						-0.57							K09267	Transcription factor SOX1/3/14/21 (SOX group B)
LOC115529463	0.46					0.47							K15698	RING finger protein 121/175
LOC115529472	-0.55		-0.73										K09058	Thyrotrophic embryonic factor
LOC115529473						0.75								
LOC115529475												Gain		
LOC115529480				-0.93		-1.00								
LOC115529483									Gain			Gain		
LOC115529487						-0.58								
LOC115529500						0.88								
LOC115529503	-3.03	-0.34	-0.55										K23284	C1q-related factor
LOC115529506	0.42													
LOC115529508	-0.96		-0.57											Transmembrane 4 L6 family member 4
LOC115529513	-0.58													Protein phosphatase 1 regulatory subunit 27
LOC115529523			-0.50										K10510	Zinc finger and BTB domain-containing protein 38
LOC115529532						-0.62								
LOC115529536							Gain							
LOC115529546			-0.77										K16355	Leucine-rich repeat and fibronectin type-III domain-containing protein 2
LOC115529565	-0.38													Ankyrin
LOC115529567			0.55										K12820	Pre-mrna-splicing factor ATP-dependent RNA helicase DHX15/PRP43
LOC115529572						-0.70								ABC-2 type transport system ATP-binding protein, ATP-binding cassette, subfamily A (ABC1), member 1, ATP-binding cassette, subfamily A (ABC1), member 3

LOC115529574				0.57		0.62							K24504	Nuclear body protein Sp140
LOC115529582				-1.88		-5.48							K01424, K13278	60kda lysophospholipase , L-asparaginase
LOC115529583	0.59					0.45								ATP-binding cassette, sub-family E, member 1
LOC115529587		-0.18		-3.30		-7.51								60kda lysophospholipase , L-asparaginase
LOC115529588						-1.37							K01424, K13278	60kda lysophospholipase , L-asparaginase
LOC115529589						0.41								
LOC115529590						0.64								
LOC115529604	0.51		0.52											
LOC115529607	-0.77	-0.42	-0.53											
LOC115529614	-0.79													
LOC115529619			-0.96	-0.54		-1.04								
LOC115529623			0.73	0.40		2.15								
LOC115529629	-0.41													Synaptopodin-2
LOC115529631	0.64	0.68	0.67										K14007	Protein transport protein SEC24
LOC115529636											Gain			
LOC115529637							Loss		Loss					
LOC115529639	0.47													
LOC115529641			0.40	-0.46		-0.52							K13187	RNA-binding protein 4
LOC115529645				0.52										Tudor domain-containing protein 1/4/6/7
LOC115529654	-1.01	-0.42	-0.82											Insulin receptor substrate 2
LOC115529655						0.62							K22816	Protein canopy 3/4
LOC115529656				1.97									K08852	Serine/threonine-protein kinase/endoribonuclease IRE1
LOC115529659			-0.64											
LOC115529660		-0.29												
LOC115529662	-0.42						Loss		Loss		Gain			
LOC115529664	-0.97		-0.72											
LOC115529672	-0.41													
LOC115529676							Gain							
LOC115529678						-0.41							K00948	Ribose-phosphate pyrophosphokinase
LOC115529680						-1.26								
LOC115529686	-0.62			0.42										

LOC115529690								Gain			Gain	Gain + Loss		Putative transposase
LOC115529691						0.87								
LOC115529695	-0.63													
LOC115529708						0.70								
LOC115529717												Loss	K23014	Inactive dipeptidyl peptidase 10
LOC115529718							Gain				Loss	Loss		
LOC115529725	0.41	0.37	0.37											
LOC115529737	0.37													
LOC115529741												Loss		
LOC115529743	0.62					-1.05							K13187	RNA-binding protein 4
LOC115529747												Loss		
LOC115529749						0.64						Gain		Solute carrier family 29 (equilibrative nucleoside transporter), member 1/2/3
LOC115529754	0.49		0.36										K03259	Translation initiation factor 4E
LOC115529757	-0.81					-0.57		Gain						
LOC115529761	1.26													Purine-nucleoside phosphorylase
LOC115529766	-0.53													Insulin receptor substrate 1
LOC115529767												Loss		
LOC115529784	0.36					-0.35								RNA-binding protein 14
LOC115529807								Gain	Gain					
LOC115529808											Gain	Gain		
LOC115529810		-0.40												
LOC115529817						0.51								
LOC115529832	0.53												K12168	E3 ubiquitin-protein ligase HECW2
LOC115529839	-1.70	-0.42	-1.30											
LOC115529847	-0.43													
LOC115529850						0.71								Phosphatidate phosphatase
LOC115529851						-1.02								P24 family protein gamma-1
LOC115529855	-2.52												K08574	Calpain-5
LOC115529860	-0.52												K15078	Structure-specific endonuclease subunit SLX1
LOC115529861						0.70								
LOC115529868						0.62							K06071	Serine/threonine-protein kinase N1

LOC115529887						0.81							K00922	Phosphatidylinositol-4,5-bisphosphate 3-kinase catalytic subunit alpha/beta/delta
LOC115529889						-0.90								Notch 3
LOC115529890						0.63								
LOC115529909				-0.38		-0.60								E3 SUMO-protein ligase CBX4
LOC115529920			-0.35											Chromobox protein 8
LOC115529924	0.64		0.56			-0.71								
LOC115529931	-1.60		-1.76									Gain + Loss		Kelch-like protein 33
LOC115529941				0.46		1.23					Loss			
LOC115529944						0.71								
LOC115529949												Gain + Loss		
LOC115529952				0.39										
LOC115529958						0.78								
LOC115529969				-0.47	-1.14	-1.18								
LOC115529990												Loss	K21853	Dedicator of cytokinesis protein 9/10/11
LOC115529993						-0.57							K06593	Poliovirus receptor-related 4
LOC115529994			0.39				Loss					Loss		
LOC115530000				-0.44		-0.84							K13187	RNA-binding protein 4
LOC115530001	0.97	0.63	0.72			0.46							K13187	RNA-binding protein 4
LOC115530008			0.82											
LOC115530010							Gain	Gain	Gain			Gain		
LOC115530018						0.60							K05849	Solute carrier family 8 (sodium/calcium exchanger)
LOC115530020	0.56			-0.41		-0.62								Dnaj homolog subfamily B member 1
LOC115530021											Loss	Loss		
LOC115530027						-0.62		Loss				Loss		Very-long-chain enoyl-coa reductase
LOC115530030												Gain		
LOC115530042						0.61								
LOC115530065						1.13								
LOC115530238				0.39		0.93								Autophagy-related protein 18
LOC115530351	-0.55		-0.60			0.64							K04738	Cytokine receptor common subunit beta
LOC115530387	-0.48		-0.38	-0.42		-0.47								Solute carrier family 6 (neurotransmitter transporter, amino acid/orphan) member 15/16/17/18/20

LOC115530403				-0.63										MFS transporter, ACS family, solute carrier family 17 (sodium-dependent inorganic phosphate cotransporter), member 6/7/8
LOC115530419						0.62								
LOC115530430	-0.53	-0.32	-0.55			0.38								V-type H+-transporting atpase subunit a
LOC115530492				0.37									K04430	Mitogen-activated protein kinase kinase 4
LOC115530622						0.47							K17480	Lemur tyrosine kinase 1
LOC115530624						0.40								Charged multivesicular body protein 6
LOC115530632						0.44								BTB/POZ domain-containing protein 17
LOC115530634						-1.08							K21878	Usher syndrome type-1G protein
LOC115530652						0.71							K00261	Glutamate dehydrogenase (NAD(P)+)
LOC115530675						-0.34							K17095	Annexin A7/11
LOC115530677						-1.15								
LOC115530680											Gain	Gain		
LOC115530687	-0.42												K00789	S-adenosylmethionine synthetase
LOC115530689				-0.46										
LOC115530693				0.33										
LOC115530696			0.45											Ataxin 2/2L
LOC115530709						0.60								C-Jun-amino-terminal kinase-interacting protein 4
LOC115530726											Loss			
LOC115530730	0.43													Cytochrome b-245 chaperone 1
LOC115530732			0.33			0.56								Histone H3
LOC115530752						-0.56							K03026	DNA-directed RNA polymerase III subunit RPC4
LOC115530759	-2.21	-1.58	-2.51											Shingomyelin synthase
LOC115530760	0.67	0.67	0.76											Prolyl 4-hydroxylase
LOC115530763						0.55							K06843	Netrin 1
LOC115530764												Gain	K09401	Forkhead box protein I
LOC115530765	-0.93	-0.61	-0.85										K17987	Next to BRCA1 gene 1 protein
LOC115530769						0.79								
LOC115530776	0.68		0.47									Loss		
LOC115530777				-0.60		-0.52								
LOC115530780						-0.52								Supervillin
LOC115530782						-0.49						Gain	K05648	ATP-binding cassette, subfamily A (ABC1), member 5
LOC115530789	-0.48	-0.58	-0.41			-0.68								Discs large protein 5

LOC115530790						-0.58								Potassium large conductance calcium-activated channel subfamily M alpha member 1
LOC115530797											Loss	Loss		NADPH oxidase organizer 1
LOC115530799				-0.42		-0.80								
LOC115530817			-0.82											
LOC115530841						0.73							K04429	Thousand and one amino acid protein kinase
LOC115530842	-0.40			-0.51		-1.31								General transcription factor IIIA
LOC115530851												Gain	K09314	Homeobox protein DLX1/4/6
LOC115530857						0.54							K07759	Poly(ADP-ribose) glycohydrolase
LOC115530858												Loss	K06834	Disintegrin and metalloproteinase domain-containing protein 9
LOC115530861	-1.76		-1.42											
LOC115530864			-0.46											
LOC115530872				-0.42		-1.18								
LOC115530887	-1.12	-0.38	-0.68			-0.47								
LOC115530900			0.54											Collagen type I alpha
LOC115530902											Loss	Loss	K09516	All-trans-retinol 13,14-reductase
LOC115530913						-0.47								
LOC115530919						-0.48								KRAB domain-containing zinc finger protein
LOC115530920	-0.46			-0.54		-1.12								
LOC115530921						-0.64								General transcription factor IIIA, KRAB domain-containing zinc finger protein
LOC115530935									Loss					
LOC115530936			-0.54			-0.72								Signal transducer and activator of transcription 5B
LOC115530941		-0.43											K10334	Ankyrin repeat and SOCS box protein 12
LOC115530956						-0.77								Mitogen-activated protein kinase kinase 4
LOC115530959			0.44										K14005	Protein transport protein SEC31
LOC115530972	-0.73		-0.66			-0.97								
LOC115530976						-0.68								
LOC115530981						0.75								
LOC115530990	0.61	0.40	0.48											Helicase SRCAP/SWR1
LOC115530992						0.71								
LOC115531009				0.50									K00907	Myosin-light-chain kinase
LOC115531014		0.41	0.45										K10949	ER lumen protein retaining receptor
LOC115531020						0.61								Dnaj homolog subfamily C member 7

LOC115531025												Loss	K23712	SH3 and cysteine-rich domain-containing protein 2
LOC115531027	-1.48	-1.34	-0.96				Gain + Loss					Gain		
LOC115531028							Gain							
LOC115531040	0.37		0.39			0.31							K08486, K13502	Syntaxin 1B/2/3, syntaxin 4
LOC115531065						0.63							K03252	Translation initiation factor 3 subunit C
LOC115531068												Gain	K19721	Collagen type V/XI/XXIV/XXVII, alpha
LOC115531074				0.42										Next to BRCA1 gene 1 protein
LOC115531075		-0.69	-0.53											Tankyrase , uncharacterized protein
LOC115531081				0.43										Rho family gtpase 2
LOC115531087	-0.95					-0.67								Sesquipedalian
LOC115531088												Loss		Fast skeletal myosin light chain 2
LOC115531089						0.51								Elongin-B
LOC115531095	-1.04													
LOC115531096						0.73								BTB/POZ domain-containing protein KCTD2/5/17
LOC115531098			0.33			-0.73					Loss		K20636	Rho gtpase-activating protein 12/27
LOC115531101						0.49							K02155	V-type H+-transporting atpase 16kda proteolipid subunit
LOC115531102	-0.29												K19387	Polymerase I and transcript release factor
LOC115531104	-0.30	-0.27	-0.45											
LOC115531106									Gain		Gain			
LOC115531114						0.45								
LOC115531119	0.27		0.47											ADP-ribosylation factor 1/2
LOC115531130	-0.87		-0.87										K11862	Ubiquitin thioesterase ZRANB1
LOC115531134								Loss						Ubinuclein
LOC115531135	-0.69													Atypical dual specificity phosphatase
LOC115531138	0.38	0.36	0.40										K08106	N-acetylgalactosamine 4-sulfate 6-O-sulfotransferase
LOC115531140						0.62								Histone H1/5
LOC115531141		-0.39												Ubiquitin carboxyl-terminal hydrolase 22/27/51
LOC115531145						-0.53								Phosphatidylinositol phospholipase C, delta
LOC115531146											Loss	Loss	K20315	Rho gtpase-activating protein 21/23
LOC115531148						-0.76								
LOC115531151						-0.40								
LOC115531153						-0.65								

LOC115531155	-1.76	-0.48	-2.14										K00898	Pyruvate dehydrogenase kinase 2/3/4
LOC115531156	0.63													Solute carrier family 25, member 38
LOC115531158						0.37							K11660	Metastasis-associated protein MTA
LOC115531160				0.46									K21067	Rho gtpase-activating protein 44
LOC115531164						-0.81								Medium-chain acyl-coa ligase, mitochondrial
LOC115531165	0.91	0.42	0.37										K18441	Cytohesin
LOC115531167	-2.01	-1.61	-2.38			-0.70								Nuclear distribution protein nude-like 1
LOC115531173				0.51										A disintegrin and metalloproteinase with thrombospondin motifs 14
LOC115531176						-0.53							K08796	BR serine/threonine kinase
LOC115531178						0.89								
LOC115531188						0.62								Mitogen-activated protein kinase kinase 6
LOC115531190	-0.45		-0.44											Inhibitor of nuclear factor kappa-B kinase subunit alpha
LOC115531191				-0.56		-1.04								
LOC115531197						-0.92								
LOC115531201						0.66								Vitamin-K-epoxide reductase (warfarin-sensitive)
LOC115531205	0.75	0.41	0.54			-0.57								Solute carrier family 6 (neurotransmitter transporter, amino acid/orphan) member 15/16/17/18/20
LOC115531209	0.41		0.50										K00507	Stearoyl-coa desaturase (Delta-9 desaturase)
LOC115531210	0.62		0.66											FK506-binding protein 9/10
LOC115531211	-0.49					-0.71							K24242	7-methylguanosine nucleotidase
LOC115531212						-0.47								
LOC115531221				0.76		0.82							K05152	Tumor necrosis factor receptor superfamily member 14
LOC115531249						-0.60							K16360	Netrin-G3 ligand
LOC115531254							Loss							Parathyroid hormone receptor 1
LOC115531257	-0.73		-1.25			0.86								Chromobox protein 7
LOC115531265				-0.51										
LOC115531272												Loss		
LOC115531273			0.60											
LOC115531276						0.69								Integrin alpha M
LOC115531287	0.81		0.63						Gain			Gain		MFS transporter, MCT family, solute carrier family 16 (monocarboxylic acid transporters), member 6
LOC115531291						0.92								
LOC115531292	0.43	0.29	0.40											

LOC115531295						0.44							K09228	KRAB domain-containing zinc finger protein
LOC115531296							Gain + Loss	Loss					K09228	KRAB domain-containing zinc finger protein
LOC115531298												Gain		
LOC115531299			0.56											Collagen type I alpha
LOC115531301		0.34	0.51										K10949	ER lumen protein retaining receptor
LOC115531302							Loss		Loss		Loss	Gain + Loss		
LOC115531310						-0.80								Centromere protein T
LOC115531311	-0.44		-0.33			-0.43								Josephin
LOC115531314	-0.40												K13365	Sodium/hydrogen exchange regulatory cofactor NHE-RF1
LOC115531315						-0.65	Loss							Ubiquitin-conjugating enzyme E2 D
LOC115531316	0.51													Ras-related protein Rab-37
LOC115531317												Gain		
LOC115531318	-0.63													
LOC115531330	-1.52					-1.14								SPRY domain-containing SOCS box protein 3
LOC115531333	-0.55													Parvalbumin alpha
LOC115531343	-0.73													
LOC115531348	-0.54					-0.99							K19347	SUN domain-containing protein 1/2
LOC115531368		0.42												Neuronal pentraxin
LOC115531373	-1.46	-0.46	-1.36										K14165	Atypical dual specificity phosphatase
LOC115531377	-0.70												K07520	Actin-binding LIM protein
LOC115531378	-0.79													WNK lysine deficient protein kinase
LOC115531381	0.85												K15040	Voltage-dependent anion channel protein 2
LOC115531396	-0.37		-0.62										K14360	Organic solute transporter subunit alpha
LOC115531400			-0.49			0.57								
LOC115531405											Loss	Loss		
LOC115531411						0.93					Gain	Gain	K04429	Thousand and one amino acid protein kinase
LOC115531412	-0.39		-0.41			-0.51							K01424, K13278	60kda lysophospholipase , L-asparaginase
LOC115531415	-0.70		-0.72										K24387	Lethal(3)malignant brain tumor-like protein
LOC115531418						-0.40							K09058	Thyrotrophic embryonic factor
LOC115531421						0.38								Pleckstrin homology domain-containing family A member 4/5/6/7
LOC115531424				-0.43										

LOC115531431	0.35						Loss				Gain	Loss		ATP-dependent RNA helicase DHX8/PRP22
LOC115531432	-0.59													Thyroid hormone receptor alpha
LOC115531435											Gain	Gain	K09083	Atonal protein 1/7
LOC115531442	-0.71		-0.61											
LOC115531445				-0.37									K02270	Cytochrome c oxidase subunit 7a
LOC115531447	-0.62	-0.32	-0.56											Lethal(3)malignant brain tumor-like protein
LOC115531459			0.61										K17551	Neurabin
LOC115531462			0.46											Protein DBF4
LOC115531463						0.56								Protein FRA10AC1
LOC115531465												Gain		
LOC115531468						0.48			Loss					
LOC115531475				0.47									K01623	Fructose-bisphosphate aldolase, class I
LOC115531476						-0.70								
LOC115531478						0.58								SH3-domain binding protein 1
LOC115531479						0.44								
LOC115531480	0.64													Deoxyribonuclease IV
LOC115531481	0.52	0.37	0.40											Trinucleotide repeat-containing gene 6 protein
LOC115531484				-0.41		-0.52								Cytochrome c oxidase assembly protein subunit 11
LOC115531490	-4.44	-0.83	-4.02											
LOC115531491						-0.55								
LOC115531494				0.43									K20636	Rho gtpase-activating protein 12/27
LOC115531495								Gain	Gain				K01464, K07528	Dihydropyrimidinase , dihydropyrimidinase-like 2
LOC115531496	0.41		0.36			-0.44								Arf-GAP with dual PH domain-containing protein
LOC115531497				-0.47		-0.58								Disintegrin and metalloproteinase domain-containing protein 11
LOC115531499							Loss							V-type H+-transporting atpase subunit a
LOC115531509				0.72		0.58								
LOC115531510				-0.46		-0.59								
LOC115531513	-1.47						Loss		Loss		Loss	Loss		Alpha-N-acetylgalactosaminide alpha-2,6-sialyltransferase (sialyltransferase 7B)
LOC115531519						0.64							K04246	G-protein coupled estrogen receptor 1
LOC115531522	0.41			-0.40										
LOC115531528			0.39											Histone H3
LOC115531538	-0.90		-0.50								Loss	Loss		Mitogen-activated protein kinase kinase kinase 14

LOC115531543	-0.40	-0.35	-0.35	-0.34				Gain						Alpha-N-acetylgalactosaminide alpha-2,6-sialyltransferase (sialyltransferase 7A) , matrix remodeling-associated protein 7
LOC115531545	0.45		0.48										K12891	Serine/arginine-rich splicing factor 2/8
LOC115531548						0.89								
LOC115531560	-0.71		-0.77										K05005	Potassium inwardly-rectifying channel subfamily J member 12/18
LOC115531562				-0.46										Growth differentiation factor 10
LOC115531568	-1.03													
LOC115531590						-0.53								
LOC115531592												Loss		
LOC115531607											Gain	Gain	K26153	Proton channel OTOP
LOC115531608									Gain		Loss	Loss		
LOC115531623				0.42		-0.61								
LOC115531628											Gain	Gain		
LOC115531639				-0.45		-1.32								Dickkopf 1/2/4
LOC115531643						0.63							K25163	Wings apart-like protein
LOC115531646												Gain		Nuclease HARBI1
LOC115531649											Gain		K12327	Caldesmon
LOC115531666						0.35							K23792	Myosin phosphatase Rho-interacting protein
LOC115531671	-0.47													Dehydrogenase/reductase SDR family member 7C
LOC115531682						0.74								
LOC115531689						-0.56							K00623	Choline O-acetyltransferase
LOC115531690						-0.76								
LOC115531699												Gain		
LOC115531706		-0.44	-0.66											
LOC115531711												Gain		Prostasin , serine protease 27 , trypsin
LOC115531717						-0.69							K09402	Forkhead box protein J1
LOC115531719				-0.41		-0.50								Elongation of very long chain fatty acids protein 6
LOC115531785				-0.61									K13349	Mpv17-like protein
LOC115531850						0.58								
LOC115531851									Loss			Gain		Glutamine synthetase
LOC115531870	-0.50					-0.74								
LOC115531875								Gain			Gain			
LOC115531878				-0.40		-0.64							K05676	ATP-binding cassette, subfamily D (ALD), member 2

LOC115531887	-0.39												K17291	MARVEL domain-containing protein 2
LOC115531895			0.47											
LOC115531896	-0.51		-0.54											
LOC115531898			0.56											
LOC115531902	0.42													Dnaj homolog subfamily B member 9
LOC115531907	-1.15		-0.62			0.68								
LOC115531912	-0.29	-0.35	-0.31											MAD, mothers against decapentaplegic interacting protein
LOC115531918	-0.63												K01137	N-acetylglucosamine-6-sulfatase
LOC115531927	0.47		0.61											
LOC115531929						4.73							K11251	Histone H2A
LOC115531932				-0.35									K04552	Ubiquitin-conjugating enzyme E2 L3
LOC115531937	0.37	0.34	0.40											Calumenin
LOC115531948						-0.55							K05724	FYVE, rhogef and PH domain containing 5/6
LOC115531949								Loss			Loss		K04861	Voltage-dependent calcium channel alpha-2/delta-4
LOC115531958	0.50	0.34	0.41	-0.40		-0.53							K00088	IMP dehydrogenase
LOC115531960												Loss	K19572	Adenosine deaminase CECR1
LOC115531964						0.91								
LOC115531966	0.52			-0.52										
LOC115531968	-0.42		-0.42	-0.67										
LOC115531979			-0.49										K15465	BCL2/adenovirus E1B 19 kda protein-interacting protein 3-like
LOC115531990	-0.57		-0.48										K10421	CAP-Gly domain-containing linker protein 1
LOC115532001	2.05													
LOC115532002						-0.43							K04511	Prickle
LOC115532022	0.54		0.59											
LOC115532037						-0.36							K02085	Adenomatosis polyposis coli protein
LOC115532038				-0.49		-1.04							K05056	Interleukin 11 receptor alpha
LOC115532040						-0.54								
LOC115532042						-0.44								
LOC115532052	-1.44	-0.53	-1.35											Ankyrin repeat and SOCS box protein 13
LOC115532054				0.46										
LOC115532060	0.62		0.59								Gain	Gain		RAD52 motif-containing protein 1
LOC115532063						0.82						Gain		
LOC115532074	-0.41		-0.62			-0.53								

LOC115532079	-0.47												K17537	Tyrosine-protein kinase sgk223
LOC115532091						0.63							K01992	ABC-2 type transport system permease protein
LOC115532093	-0.42	-0.36	-0.37											Tuberous sclerosis 1
LOC115532096				-0.68		-1.01								
LOC115532099	0.55		0.33			-0.34								COMPASS component SWD3
LOC115532102				0.53		0.81							K11349, K11380	Bromodomain-containing protein 1, nua3 HAT complex component NTO1
LOC115532105			-0.33											CDK5 regulatory subunit-associated protein 2
LOC115532110											Loss	Loss	K16519	A-kinase anchor protein 2
LOC115532111							Loss		Loss		Gain			
LOC115532113						-0.45								Sorting nexin-9/18/33
LOC115532119	-1.02	-0.70	-0.83			-0.47								
LOC115532124											Loss	Loss	K05046	Solute carrier family 6 (neurotransmitter transporter, GABA) member 13
LOC115532132				-0.42										
LOC115532134				-0.54										
LOC115532138	0.48													N-acetylgalactosaminide alpha-2,6-sialyltransferase (sialyltransferase 7D)
LOC115532141						-0.60							K21392	Adipocyte enhancer-binding protein 1
LOC115532143						-0.74							K13676	UDP-D-xylose:beta-D-glucoside alpha-1,3-D-xylosyltransferase
LOC115532144						-0.77								YY1-associated factor 2
LOC115532153									Loss					
LOC115532161								Loss						Kinesin family member 21
LOC115532166			-0.46											Mannose receptor, C type
LOC115532177			-0.43											5'-AMP-activated protein kinase, regulatory beta subunit
LOC115532181											Loss	Loss		Amine sulfotransferase
LOC115532183						0.63							K17821	E3 ubiquitin-protein ligase RNF165
LOC115532203	-0.56													Apolipoprotein L
LOC115532204						0.64								Heat shock protein 90kda beta, molecular chaperone htpg
LOC115532211			-0.88											
LOC115532212						-0.47							K02599	Notch 1
LOC115532220						-0.74							K16815	Calcium-independent phospholipase A2-gamma
LOC115532221						-0.57								Neuronal cell adhesion molecule
LOC115532225						0.67								Anthrax toxin receptor

LOC115532235	-0.56		-0.65	0.46		0.49							K09191, K09228	General transcription factor IIIA, KRAB domain-containing zinc finger protein
LOC115532245						-0.77								
LOC115532249	-0.47													
LOC115532250												Loss		
LOC115532255						-1.52								
LOC115532256	-0.59			-0.38										
LOC115532259						-0.61								
LOC115532266	-0.66					0.72							K01754	Threonine dehydratase
LOC115532277						-0.89		Gain				Gain		
LOC115532284	-1.44	-0.89	-1.61											
LOC115532288						-0.47								Protein piccolo
LOC115532291						-0.65								Adh transcription factor 1
LOC115532302	-0.43	-0.38	-0.49			0.43								
LOC115532313	0.75	0.34	0.39			0.36								Type I protein arginine methyltransferase
LOC115532320	0.74													Dynamin 1-like protein
LOC115532326						-0.53								V-type H+-transporting atpase subunit G
LOC115532327	-0.67					0.69								
LOC115532340				0.52										Galectin-3-binding protein
LOC115532345			-0.48											Zinc finger SWIM domain-containing protein 6
LOC115532347										Gain				Myosin phosphatase Rho-interacting protein
LOC115532351	0.40	0.55	0.50			0.41							K07870	Mitochondrial Rho gtpase 1
LOC115532354						0.60								
LOC115532356												Gain		General transcription factor IIIA, KRAB domain-containing zinc finger protein
LOC115532361	0.34		0.49			-0.58							K24031	Lamina-associated polypeptide 2
LOC115532366							Gain	Loss					K11846	Ubiquitin carboxyl-terminal hydrolase 18/41
LOC115532389						0.37								Fructose-2,6-bisphosphatase
LOC115532391						1.33							K11251	Histone H2A
LOC115532396						0.61							K11275	Histone H1/5
LOC115532405						0.54							K11251	Histone H2A
LOC115532418												Gain		Tetratricopeptide repeat protein 28
LOC115532452	-0.66			0.75										Cholesterol 7-desaturase
LOC115532462						-0.71								

LOC115532467				1.92		0.71								NLR family CARD domain-containing protein 3
LOC115532468						0.57								
LOC115532479	0.52										Loss			Ceramide kinase
LOC115532491								Loss					K04907	Potassium voltage-gated channel Eag-related subfamily H member 4
LOC115532500											Loss			Alanine-glyoxylate transaminase / (R)-3-amino-2-methylpropionate-pyruvate transaminase
LOC115532502						0.95								Sphingosine 1-phosphate receptor 3
LOC115532515				0.49										Protein Shroom
LOC115532525						-0.46		Gain						
LOC115532536												Loss		
LOC115532538						-0.87								Hepatocyte growth factor
LOC115532544											Loss	Loss		Proton channel OTOP
LOC115532547						-0.62						Gain		Type I protein arginine methyltransferase
LOC115532558							Gain	Loss			Gain + Loss	Gain + Loss		NLR family CARD domain-containing protein 3
LOC115532568									Loss					
LOC115532577						0.50								
LOC115532584							Gain	Gain						
LOC115532585											Gain	Gain		
LOC115532589				-0.33		-0.59							K25423	Dynein axonemal assembly factor 8
LOC115532605			-0.41			0.63								Cyclin-dependent kinase 17
LOC115532606						-0.63								ETS domain-containing protein Elk-3 (SRF accessory protein 2)
LOC115532629	0.73													
LOC115532633	-0.97	-0.45												
LOC115532634	-0.95		-0.67											
LOC115532641												Loss	K16302	Metal transporter CNNM
LOC115532645				0.89		0.47							K06106	Cortactin
LOC115532646							Gain							
LOC115532649							Loss							
LOC115532652											Gain			
LOC115532658						-0.80							K18484, K18485	Myogenic factor 5, myogenic factor 6
LOC115532661				0.89		0.58								
LOC115532672							Loss	Loss	Loss			Loss		

LOC115532675	-0.79		-0.47										K10486	BTB/POZ domain-containing protein 14
LOC115532689						0.76								
LOC115532710				-0.45		-1.05	Loss	Loss			Loss	Loss		Homeobox protein HB9
LOC115532718	0.44													
LOC115532728											Loss	Loss	K16499	Protocadherin delta 2
LOC115532738	-0.37													Aryl hydrocarbon receptor
LOC115532755						-0.78							K17477	FERM, rhogef and pleckstrin domain-containing protein 1
LOC115532763							Loss					Gain		
LOC115532764					0.97									
LOC115532770						0.69							K07888, K07889	Ras-related protein Rab-5B, Ras-related protein Rab-5C
LOC115532772											Loss			
LOC115532773	0.58		0.40										K11718	UDP-glucose:glycoprotein glucosyltransferase
LOC115532775			-0.52											
LOC115532781				-0.42		-0.83								Calcitonin receptor-like
LOC115532792				0.48										Voltage-dependent calcium channel P/Q type alpha-1A
LOC115532793						-0.60								Immunoglobulin-like domain-containing receptor 1
LOC115532808						-0.63								Transcription factor Dp-1
LOC115532810	-0.63		-0.79										K17822	DCN1-like protein 1/2
LOC115532829			0.60											
LOC115532830				0.52										ABC-2 type transport system permease protein
LOC115532838						0.94							K08383	G protein-coupled receptor 34
LOC115532841				0.43		0.73						Gain		
LOC115532855				1.96		1.17								
LOC115532863									Loss	Loss	Loss	Loss		Cell surface glycoprotein CD200 receptor
LOC115532868							Gain	Gain						
LOC115532875						-1.09								
LOC115532876						0.48							K11406	Histone deacetylase 4/5
LOC115532879			-0.72										K15731	Carboxy-terminal domain RNA polymerase II polypeptide A small phosphatase
LOC115532881				-0.38		-0.40								Kelch repeat and BTB domain-containing protein 6/7
LOC115532887		-0.81	-0.50											
LOC115532892						0.42							K19868	Thioredoxin domain-containing protein 3
LOC115532895						1.12							K23575	Insulin-like growth factor-binding protein 2

LOC115532898				-0.71		-1.06								
LOC115532918						-0.41								Camp-specific phosphodiesterase 4
LOC115532920				-0.48		-0.64								Hydroxyacylglutathione hydrolase , paroxysmal nonkinesiogenic dyskinesia protein
LOC115532921							Loss				Loss	Gain + Loss	K24205	Protein lifeguard
LOC115532939	-1.15	-0.49	-0.54											Poly
LOC115532948											Loss	Loss		Tripartite motif-containing protein 39
LOC115532969												Loss		
LOC115532987						-1.27								Collagen type XXVIII alpha
LOC115532994											Gain	Gain		
LOC115532998						0.62								Dipeptidyl-peptidase 4
LOC115533003							Gain							CD302 antigen
LOC115533007	0.90													Bromodomain adjacent to zinc finger domain protein 2B
LOC115533008	-0.45												K19008	Serine/threonine-protein kinase SIK1
LOC115533034				-0.46									K18178	Cytochrome c oxidase assembly factor 5
LOC115533042			-0.29										K04699	Suppressor of cytokine signaling 6/7
LOC115533044	-0.33					-0.42								Double stranded RNA-specific editase B
LOC115533046	0.68	0.35	0.58											
LOC115533049						0.71							K07621	Gap junction beta-2 protein
LOC115533052											Gain + Loss	Gain		
LOC115533061	0.46												K13172	Serine/arginine repetitive matrix protein 2
LOC115533071						-1.02								
LOC115533076				4.04		2.17								
LOC115533091						-0.39								
LOC115533101							Gain		Gain		Loss	Loss	K22438	Trans-aconitate 3-methyltransferase
LOC115533110						0.56								BTB/POZ domain-containing protein 14
LOC115533112				1.38									K24135	MORC family CW-type zinc finger protein
LOC115533123						-0.38								
LOC115533134	-1.57	-0.52	-0.99										K00488	Cholestanetriol 26-monooxygenase
LOC115533135						0.45							K08900	Mitochondrial chaperone BCS1
LOC115533137						0.91								Phosphoethanolamine/phosphocholine phosphatase
LOC115533139			0.43			-0.88							K06823	Collagen type XVIII alpha

LOC115533146						0.55								
LOC115533147						-0.57								
LOC115533155	0.48					-0.50							K04374	Cyclic AMP-dependent transcription factor ATF-4
LOC115533159	0.36		0.34										K07374	Tubulin alpha
LOC115533165	0.43													
LOC115533170						0.90							K24220	Myosin heavy chain 1/2/3/4/8/13/7B/15
LOC115533175											Gain			C-X-C chemokine receptor type 2
LOC115533190	0.53													Chaperonin groes, MOB kinase activator 1
LOC115533195						-0.78							K00779	Beta-galactoside alpha-2,6-sialyltransferase (sialyltransferase 2)
LOC115533200	-0.56		-0.48											
LOC115533202										Gain	Gain	Gain		
LOC115533204						0.65								
LOC115533219	-0.46		-0.50										K06528	Lysosomal-associated membrane protein 1/2
LOC115533225						-0.34	Loss						K01425	Glutaminase
LOC115533237	-0.76	-0.40	-0.69										K12309, K25543	Beta-galactosidase , beta-galactosidase-1-like protein
LOC115533279		0.42												
LOC115533282				-0.96		-2.09								
LOC115533286				0.42				Loss						Solute carrier family 15 (oligopeptide transporter), member 2
LOC115533287	-0.56													Cholestanetriol 26-monooxygenase
LOC115533289						0.75							K04855	Voltage-dependent calcium channel T type alpha-1H
LOC115533329			0.70			-0.84							K07604	Type I keratin, acidic
LOC115533333						-0.67							K23310	PHD finger protein 6
LOC115533341	-0.79													
LOC115533343	0.39	0.30	0.32				Loss			Loss	Loss	Loss	K11585	Chromobox protein 1
LOC115533345	-0.81		-0.76			-0.62							K20641	Rho gtpase-activating protein 20
LOC115533346							Loss	Loss			Gain + Loss	Gain		
LOC115533350				-0.36		-0.63							K07616	Gap junction gamma-1 protein
LOC115533354				-0.41		-0.58								Rac gtpase-activating protein 1
LOC115533365	-0.45													Alpha-1,2-mannosyltransferase
LOC115533371											Loss	Loss		
LOC115533372						-0.77							K00546	Histamine N-methyltransferase
LOC115533377	0.70													U3 small nucleolar RNA-associated protein 14

LOC115533379												Gain		
LOC115533383			0.47										K01852	Lanosterol synthase
LOC115533385												Gain		Ribonuclease ZC3H12
LOC115533404	0.49	0.31											K02131	F-type H+-transporting atpase subunit 6
LOC115533407			-0.41									Loss	K00901	Diacylglycerol kinase (ATP)
LOC115533419						-0.63								Poly(rc)-binding protein 3/4
LOC115533422						-0.60								
LOC115533423											Loss	Loss		
LOC115533430			-0.45											G protein-coupled receptor family C group 5 member B
LOC115533439				-0.39		-0.46								Rho family gtpase 3
LOC115533451						4.67							K23483	Gamma-crystallin
LOC115533452						0.44							K23483	Gamma-crystallin
LOC115533455	0.53		0.60											
LOC115533456	0.35													Calsequestrin 2
LOC115533457						0.90				Gain	Gain	Gain	K04896	Potassium voltage-gated channel Isk-related subfamily E member 2
LOC115533459	-0.37			-0.42										
LOC115533468	-0.43		-0.43											
LOC115533476						0.65								
LOC115533487												Gain		
LOC115533488											Gain	Gain		
LOC115533493					-1.82	-0.59								Caspase 8
LOC115533498											Gain			
LOC115533501											Gain			
LOC115533505				0.53		0.78								
LOC115533511	-0.46		-0.36										K11795	DDB1- and CUL4-associated factor 6
LOC115533512						0.58					Loss	Loss		
LOC115533515						0.61							K00803	Alkyldihydroxyacetonephosphate synthase
LOC115533537						-0.94							K04831	Acid-sensing ion channel 4
LOC115533540				-0.42		-0.75								Pyridoxine kinase
LOC115533550						0.79								Obscurin-like protein 1
LOC115533566						0.54							K22193	Golgi ph regulator
LOC115533567			0.42			0.58								Ribulose-phosphate 3-epimerase

LOC115533570							Loss	Loss			Gain + Loss	Gain + Loss		
LOC115533581	-0.60	-0.66	-0.85										K23354	LIM and senescent cell antigen-like-containing domain protein 1/2
LOC115533582				0.44									K04398	Caspase 8
LOC115533592				-0.39		-0.59								Zinc finger homeobox protein 2
LOC115533622						-0.79							K09628	Serine protease 27
LOC115533634						-0.54							K09191	General transcription factor IIIA
LOC115533635			-0.55											Myosin X
LOC115533644											Gain	Gain	K08103	Heparan sulfate 6-O-sulfotransferase HS6ST3
LOC115533645									Gain				K18977	Hemopexin
LOC115533651	0.54	0.34	0.43			0.40					Gain		K12172	E3 SUMO-protein ligase ranbp2
LOC115533657						-0.76								Inhibin beta B chain
LOC115533659							Gain						K07817	Receptor-type tyrosine-protein phosphatase N
LOC115533660								Gain						
LOC115533664						-0.49								MFS transporter, NAG-T family, sugar:H+ symporter
LOC115533667						-0.58								
LOC115533669						0.31							K23483	Gamma-crystallin
LOC115533681							Loss				Gain	Gain		
LOC115533688						-0.60								Solute carrier family 5 (high affinity choline transporter), member 7
LOC115533694										Loss				
LOC115533708						0.82							K23455	Collagen type VIII alpha
LOC115533729												Loss		
LOC115533731						-1.07								
LOC115533897				0.44		0.55								Phospholipase B1, membrane-associated
LOC115533900							Gain							
LOC115533921				0.41		0.57							K04899	Potassium voltage-gated channel subfamily F member 1
LOC115533923						0.66								
LOC115533936	-1.50	-1.01	-1.60										K15626	Nuclear protein 1
LOC115533939						0.59								
LOC115533945	1.17	0.77	0.90										K03036	26S proteasome regulatory subunit N6
LOC115533950						0.70								
LOC115533975	-0.97	-0.88	-1.36	0.45		0.96						Loss		

LOC115533976				-4.04		-2.17								
LOC115533977				-0.67		-1.06								
LOC115533981				0.44										Sushi domain-containing protein 3
LOC115533982						-0.58							K00967	Ethanolamine-phosphate cytidylyltransferase
LOC115533987								Gain				Loss		
LOC115533989	-0.60			-0.41		-1.01								
LOC115533999	-0.56	-0.44	-0.95			-0.68							K19363	Lipopolysaccharide-induced tumor necrosis factor-alpha factor
LOC115534000						-0.81							K08815	Tau tubulin kinase
LOC115534001								Loss					K08573	Calpain-3
LOC115534005												Loss		
LOC115534008				-0.32										
LOC115534014											Loss	Gain		
LOC115534018				0.47		0.59								Cathepsin B
LOC115534020						0.80							K01363	Cathepsin B
LOC115534025	-0.87		-0.97										K15789	Threonine 3-dehydrogenase
LOC115534053	0.53					0.73							K04079	Molecular chaperone htpg
LOC115534054	0.74												K04079	Molecular chaperone htpg
LOC115534063	-0.56		-0.50										K22382	WD repeat-containing protein 26
LOC115534066				-0.52		-0.46								Malonate-semialdehyde dehydrogenase (acetylating) / methylmalonate-semialdehyde dehydrogenase
LOC115534070				-0.48		-0.96							K11964	Pellino
LOC115534101						-0.69								Left-right determination factor
LOC115534104	-0.54		-0.43											
LOC115534105				-0.54		-0.42								
LOC115534113						-0.66								
LOC115534116				0.39									K08342	Cysteine protease ATG4
LOC115534120						0.68								
LOC115534123	-0.36													
LOC115534124						-0.59							K17388	Rho-associated protein kinase 2
LOC115534137				0.38		0.42								
LOC115534145			-0.55										K09303	Homeobox protein hoxa/B/D3
LOC115534165							Loss				Loss	Gain + Loss		Transforming growth factor beta-3
LOC115534167			-0.37											

LOC115534169	0.56					-0.59						Loss		
LOC115534176				0.39									K25442	Nuclear receptor coactivator 7
LOC115534181	-0.47		-0.38											
LOC115534183	0.76		0.49											Ribonucleoside-diphosphate reductase beta chain , ribonucleoside-diphosphate reductase subunit M2
LOC115534184	-0.49	-0.58	-0.65											Ribonucleoside-diphosphate reductase beta chain , ribonucleoside-diphosphate reductase subunit M2
LOC115534185				0.68		0.61								AT-rich interactive domain-containing protein 1
LOC115534186			-0.44			-0.54								T-lymphoma invasion and metastasis-inducing protein 2
LOC115534190		-0.54											K16675	Palmitoyltransferase ZDHHC14/18
LOC115534194				0.46										Galectin-8
LOC115534200	-0.61							Gain + Loss			Gain + Loss	Gain + Loss		
LOC115534225	-0.93		-0.37			-0.71							K00649	Glyceronephosphate O-acyltransferase
LOC115534228	0.51	0.31	0.35	0.37		0.47								Uridine kinase
LOC115534243						-0.54							K04603	Metabotropic glutamate receptor 1
LOC115534249	0.37													Sphingosine-1-phosphate phosphatase 1
LOC115534250	0.51		0.65										K11498	Centromeric protein E
LOC115534272	0.48												K13160	Heterogeneous nuclear ribonucleoprotein Q
LOC115534274		-0.58		-0.41										
LOC115534275						-0.41							K11751, K19970	5'-nucleotidase , 5'-nucleotidase / UDP-sugar diphosphatase
LOC115534278						0.59								
LOC115534286						0.57								
LOC115534291	-0.52													MFS transporter, OCT family, solute carrier family 22 (organic cation transporter), member 4/5
LOC115534302												Gain		Platelet-activating factor acetylhydrolase
LOC115534326	0.50												K05157	Tumor necrosis factor receptor superfamily member 21
LOC115534327	-0.39													
LOC115534328												Loss		Gap junction delta-2 protein
LOC115534352	-0.41												K11995	Deoxyribonuclease-1-like protein
LOC115534365			-0.49			0.56								
LOC115534366	0.39												K11162	Retinol dehydrogenase 14
LOC115534377						-1.40								
LOC115534381	-0.27												K14376	Poly(A) polymerase

LOC115534395						0.71								
LOC115534408						0.52								
LOC115534415							Gain	Gain						Cgmp-dependent protein kinase 2
LOC115534418							Gain						K09191, K22402	GDNF-inducible zinc finger protein 1, general transcription factor IIIA
LOC115534421				-0.66										Actin beta/gamma 1, actin, alpha cardiac muscle, actin, alpha skeletal muscle, centractin
LOC115534424						0.51								Cytohesin
LOC115534440						-1.02								
LOC115534441						0.36							K08875	Nuclear receptor-binding protein
LOC115534443								Loss						
LOC115534447								Gain	Gain	Gain	Gain			
LOC115534464	0.38		0.37			-0.38							K23399	MAX gene-associated protein
LOC115534465											Gain		K08048	Adenylate cyclase 8
LOC115534470						0.62							K11542	Protein CASC5
LOC115534488	0.48		0.41			-0.76								
LOC115534490			0.60											
LOC115534493	-0.71		-0.74											Proline-rich nuclear receptor coactivator 1
LOC115534495							Loss						K23449	Triadin
LOC115534506	-1.51	-0.69	-1.59										K09040	Nuclear factor erythroid 2-related factor 1/3
LOC115534508						-0.81								Coatomer subunit zeta
LOC115534521			0.45										K06059	Disintegrin and metalloproteinase domain-containing protein 17
LOC115534539						0.74								Apolipoprotein B
LOC115534562				0.62		0.86								Formic-like protein
LOC115534564	-0.58	-0.43	-0.52										K21989	Calcium permeable stress-gated cation channel
LOC115534569						-0.73							K09033	Jun dimerization protein 2
LOC115534571												Gain		
LOC115534573									Gain			Gain		
LOC115534576				-0.42									K09627	Serine protease 23
LOC115534579				0.46										Alpha-galactosidase , alpha-N-acetylgalactosaminidase
LOC115534584	-3.84	-0.70	-3.78											
LOC115534589	-0.67		-0.38											Consortin
LOC115534600						-0.62								
LOC115534601						0.68								

LOC115534605			-0.49											Terminal nucleotidyltransferase 5A/B
LOC115534606						-0.61							K09390	Transcription factor E2F6
LOC115534609				-0.39										Microtubule-associated protein, RP/EB family
LOC115534615						-0.51							K11584	Serine/threonine-protein phosphatase 2A regulatory subunit B'
LOC115534620						-0.79								
LOC115534628				-0.55		-1.20								
LOC115534632												Loss	K07440	Cholesterol 24-hydroxylase
LOC115534636						-0.71								Cholesterol 24-hydroxylase
LOC115534661						-0.32								
LOC115534664						-0.66								Lactoylglutathione lyase
LOC115534668	-0.56		-0.51											Tetraspanin-16
LOC115534672												Gain		
LOC115534674						-0.53							K05702	Afadin
LOC115534682												Gain		
LOC115534692							Loss							
LOC115534696	-0.41			0.40										Ankyrin repeat domain-containing protein 9
LOC115534698	-1.52		-0.65											Galectin-3
LOC115534724			-0.48											
LOC115534742											Gain	Gain	K19467	Matrilin
LOC115534765	-0.77					1.13								
LOC115534766						-0.48								Solute carrier family 25 (mitochondrial carnitine/acylcarnitine transporter), member 20/29
LOC115534773				0.39			Gain		Gain			Gain	K08797	Hormonally upregulated Neu-associated kinase
LOC115534774	-0.90													Thyroxine 5-deiodinase
LOC115534779	-0.51					-1.05								
LOC115534793	0.37		0.33											
LOC115534800												Gain		
LOC115534801	-0.58		-0.59										K08869	Aarf domain-containing kinase
LOC115534803						0.57								EH domain-containing protein 1, EH domain-containing protein 3
LOC115534806	-0.77	-0.43	-0.82											
LOC115534811						-1.29								BRG1-associated factor 45B/C, zinc finger protein ubi-d4
LOC115534814	0.43		0.63											
LOC115534816											Loss			Low density lipoprotein receptor adapter protein 1

LOC115534818	0.65		0.75										K17306	Armadillo repeat-containing protein 1
LOC115534825			0.47										K07604	Type I keratin, acidic
LOC115534839				0.52										
LOC115534845												Gain		Adhesion G-protein coupled receptor F3
LOC115534864											Gain		K10404	Kinesin family member 26
LOC115534866	-0.49													Solute carrier family 25, member 45/47
LOC115534869							Loss		Loss			Loss		
LOC115534873							Loss					Loss		Discs, large-associated protein 1
LOC115534877						-0.64								
LOC115534881	-0.58												K04456	RAC serine/threonine-protein kinase
LOC115534884							Gain	Loss					K24501	PR domain zinc finger protein 1
LOC115534885						-0.58							K07604	Type I keratin, acidic
LOC115534896				0.42									K15196	Transcription factor IIIB 90 kda subunit
LOC115534899	-0.41	-0.67	-0.75										K10478	BTB/POZ domain-containing protein 3/6
LOC115534911				0.39										
LOC115534946						0.58								
LOC115534948						0.51								
LOC115534961	-0.38	-0.37											K03113	Translation initiation factor 1
LOC115534977						-0.99								Mediator of RNA polymerase II transcription subunit 15
LOC115535036						0.55								Claudin
LOC115535052	-1.16													Apolipoprotein L
LOC115535124	-0.64	-0.47	-0.55	-0.45		-0.97								Phosphatidylinositol phospholipase C, delta
LOC115535150	0.64		0.48			0.42							K09516	All-trans-retinol 13,14-reductase
LOC115535245							Gain	Loss			Loss	Loss		
LOC115535252						-0.57							K11972	E3 ubiquitin-protein ligase RNF19A
LOC115535259							Gain						K05226	Cholecystokinin
LOC115535267	-0.73		-0.47											
LOC115535271												Gain	K12366	Engulfment and cell motility protein 1
LOC115535325				-0.53		-1.47								
LOC115535336	-0.47												K17936	Sorting nexin-27
LOC115535337	-1.24	-0.84	-1.22			-0.60								Histone acetyltransferase
LOC115535338			0.56											Shugoshin-like 1
LOC115535339	-0.42		-0.49											Ras-related protein Rab-5A, Ras-related protein Rab-5C

LOC115535345	0.27										Gain	Gain	K17621	Eyes absent homolog 3
LOC115535362			-0.40											Semaphorin 4
LOC115535368	-0.48		-0.50											Protein NDRG1
LOC115535376	0.55			-0.89									K03283	Heat shock 70kda protein 1/2/6/8
LOC115535397	-0.46												K23804	GON-4-like protein
LOC115535400	-0.38													Phosphatidylinositol 4-kinase B
LOC115535403				-0.41		-0.49								Bromodomain-containing protein 2
LOC115535407												Loss	K06751	MHC class I antigen
LOC115535422						-0.69								Pleckstrin homology domain-containing family O
LOC115535424	0.50												K08056	Protein disulfide-isomerase A3
LOC115535425						0.62								Retinoid X receptor beta
LOC115535427							Loss	Loss	Loss					
LOC115535441				0.44										
LOC115535452								Loss	Loss					MHC class I antigen
LOC115535455						0.55								MHC class I antigen
LOC115535458												Loss		MHC class I antigen
LOC115535480												Loss		
LOC115535483	-2.13	-0.41	-0.56			-0.70								
LOC115535484						1.56								
LOC115535485				-0.64		-0.55								
LOC115535493		-0.67												
LOC115535500							Loss		Loss		Gain	Loss		
LOC115535503	-0.49													
LOC115535515						0.72								Zinc finger protein 687
LOC115535524						-0.77								Aquaporin-10
LOC115535525						-0.93							K21599	Circadian-associated transcriptional repressor
LOC115535530												Gain		
LOC115535535		-0.34	-0.44											
LOC115535549			-0.61											Citronellyl-coa dehydrogenase
LOC115535557	-0.44		-0.49											Homocysteine-responsive endoplasmic reticulum-resident ubiquitin-like domain member 1 protein
LOC115535558				-0.47		-0.45							K14443	Protein Tob/BTG
LOC115535567	0.77		0.34											

LOC115535572						-1.04								Bone morphogenetic protein 8
LOC115535574						0.48								
LOC115535582						0.66							K09304	Homeobox protein hoxa/B/C/D4
LOC115535585												Loss		
LOC115535589			0.56										K02219	Cyclin-dependent kinase regulatory subunit CKS1
LOC115535594							Loss	Loss					K23896	Proline-rich transmembrane protein 1
LOC115535597						-0.88								Glucocorticoid modulatory element-binding protein
LOC115535602							Loss	Loss						
LOC115535605	0.49		0.55			-0.51							K09040	Nuclear factor erythroid 2-related factor 1/3
LOC115535607			0.45										K00710	Polypeptide N-acetylgalactosaminyltransferase
LOC115535610		0.38	0.46											
LOC115535614	-0.34		-0.32											Eukaryotic translation initiation factor 2C
LOC115535617				-0.42										
LOC115535618	-0.67													MHC class I antigen
LOC115535630						0.66								Solute carrier family 6 (neurotransmitter transporter) member 19, solute carrier family 6 (neurotransmitter transporter, amino acid/orphan) member 15/16/17/18/20
LOC115535636				-0.31		-0.49							K13219	Splicing factor, proline- and glutamine-rich
LOC115535637	-0.63	-0.40						Gain + Loss			Gain + Loss	Gain + Loss	K13219	Splicing factor, proline- and glutamine-rich
LOC115535639						0.83						Gain		
LOC115535646				0.32										Ankyrin repeat and IBR domain-containing protein 1
LOC115535658												Gain		
LOC115535666	0.29	0.40		0.38										Oxysterol-binding protein-related protein 9/10/11
LOC115535667				0.53										Hormone-sensitive lipase
LOC115535669	0.84													Mitochondrial import receptor subunit TOM40
LOC115535672	0.63	0.66	0.65											Eukaryotic translation initiation factor 2C
LOC115535694	0.50		0.51										K15502	Serine/threonine-protein phosphatase 6 regulatory ankyrin repeat subunit A
LOC115535703				0.36		0.36								26S proteasome regulatory subunit N13, oxysterol-binding protein-related protein 3/6/7
LOC115535710	0.66	0.44	0.39									Gain		Dolichyl-diphosphooligosaccharide---protein glycosyltransferase
LOC115535714		0.49	0.55			-0.53								Protein S100-A16
LOC115535718			0.60											Borealin
LOC115535719											Gain		K06087	Claudin

LOC115535723												Loss		
LOC115535730		-0.36												C-Myc-binding protein
LOC115535732				0.47			Gain		Gain			Gain	K10383	Envoplakin
LOC115535733	0.42	0.39	0.60	-0.37		-0.51								Histone deacetylase 1/2
LOC115535736	-0.53		-0.53											SH3-domain binding protein 5
LOC115535738						-0.69								
LOC115535750	-0.46													Zinc finger and BTB domain-containing protein 22
LOC115535756						-0.54								Myelin basic protein
LOC115535761						-0.58							K13813	Syntaxin 12/13
LOC115535767	-0.85	-0.45	-0.83											E3 ubiquitin-protein ligase ZNRF1/2
LOC115535777	-0.63		-0.57											
LOC115535782	1.37												K13216	Nuclear inhibitor of protein phosphatase 1
LOC115535801	0.96	0.80	1.28											FK506-binding protein 9/10
LOC115535804	1.03	0.69	0.74											
LOC115535810				0.45		0.52								Spire
LOC115535827						-0.47								
LOC115535829						-0.75								CCR4-NOT transcription complex subunit 3
LOC115535847	-0.49		-0.57											Signal transducer and activator of transcription 5B
LOC115535857						-0.67								
LOC115535886						0.78								C1q-related factor
LOC115535896		-0.43												Glucuronyl/N-acetylglucosaminyl transferase EXT1
LOC115535898	-0.58		-0.50	-0.43		-1.58								Carnitine O-acetyltransferase
LOC115535899	-0.64		-0.78			-0.53								Palmitoyl-protein thioesterase
LOC115535906	0.46		0.43											
LOC115535912	-0.85	-0.60												
LOC115535934												Gain		Nuclease HARBI1
LOC115535937						0.56								
LOC115535939											Loss	Loss		
LOC115535963	0.52													
LOC115535975							Loss				Gain		K20189	Dysbindin
LOC115535977	-0.87		-0.56										K12474	Low density lipoprotein receptor adapter protein 1
LOC115535979							Loss							
LOC115535981												Gain		

LOC115535983						-0.76								
LOC115536003						0.76								
LOC115536009												Loss		
LOC115536015				0.45		0.85								
LOC115536026						0.54								
LOC115536030	2.03													Chitin synthase
LOC115536033				-0.54		-0.55							K25417	Coiled-coil domain-containing protein 134
LOC115536035						-0.66							K08131	Collagen type IX alpha
LOC115536046							Gain							
LOC115536056						0.63								
LOC115536097						-0.66								
LOC115536102						-0.47								Polyhomeotic-like protein 1
LOC115536110				-0.50		-0.63								KH domain-containing, RNA-binding, signal transduction-associated protein 1
LOC115536120						0.85							K07616	Gap junction gamma-1 protein
LOC115536122			0.45											Borealin
LOC115536123	-0.36					0.40								
LOC115536130				0.47		0.81								
LOC115536134				-0.71		-0.88								RNA-binding protein 24/38
LOC115536141	-0.58		-0.44	-0.47		-0.82								Kelch-like protein 31
LOC115536142	-0.53													Elongation factor 1-alpha
LOC115536150								Loss		Loss		Gain		
LOC115536157				-0.47		-0.51								F-actin-capping protein subunit alpha
LOC115536160	0.44		0.32											
LOC115536166	0.72		0.70										K15143	Mediator of RNA polymerase II transcription subunit 30
LOC115536170	-0.83					0.65								Transmembrane glycoprotein NMB
LOC115536173						-0.58							K14620	Riboflavin transporter 2
LOC115536176							Gain		Gain		Loss			Lysophosphatidylcholine acyltransferase / lyso-PAF acetyltransferase
LOC115536177							Loss							
LOC115536180				0.47		0.70								
LOC115536186	0.55													
LOC115536190						-0.30							K24908	Disco-interacting protein 2 homolog B
LOC115536193						-0.62								Protein FAM84B

LOC115536194						-0.55								
LOC115536196						-0.57							K12485	Rab11 family-interacting protein 3/4
LOC115536202						-0.52								Tripartite motif-containing protein 46
LOC115536205						-0.30							K16197	14-3-3 protein beta/theta/zeta
LOC115536212	-0.54													
LOC115536213						0.73								
LOC115536214			0.38											
LOC115536215				-0.38		-0.85	Loss	Loss	Loss					MADS-box transcription enhancer factor 2D
LOC115536219	-0.65	-0.29	-0.45			-0.29								1-phosphatidylinositol-4-phosphate 5-kinase
LOC115536225	-0.45													E3 ubiquitin-protein ligase RNF115/126
LOC115536231						0.72								
LOC115536238						0.55							K02978	Small subunit ribosomal protein s27e
LOC115536239				-0.57		-0.96								
LOC115536240		-0.53												
LOC115536249				-0.45		-0.64								
LOC115536251						-0.73								Adhesion G protein-coupled receptor B1
LOC115536259						-0.79								Connective tissue growth factor (insulin-like growth factor-binding protein 8)
LOC115536265											Loss	Loss	K00771	Protein xylosyltransferase
LOC115536274							Loss						K15538	Glycoprotein endo-alpha-1,2-mannosidase
LOC115536293						-0.68								DENN domain-containing protein 3
LOC115536298	-0.41					-0.66								Phosphatase and actin regulator
LOC115536300				0.43		0.66							K15378	Solute carrier family 45, member 1/2/4
LOC115536303												Gain	K14710	Solute carrier family 39 (zinc transporter), member 4
LOC115536310						0.47								Cytosolic nonspecific dipeptidase
LOC115536312						-0.25								Cytosolic nonspecific dipeptidase
LOC115536321				-0.41		-0.56								
LOC115536326						-0.46								Membrane progestin receptor alpha
LOC115536330						-0.42								Transmembrane protein 222
LOC115536361	-0.53													4-galactosyl-N-acetylglucosaminide 3-alpha-L-fucosyltransferase
LOC115536368	0.53													
LOC115536374		-0.43											K20394	Sestrin 2
LOC115536376						0.78								

LOC115536425						0.46								
LOC115536435	0.61													
LOC115536626	-0.28													
LOC115536776	0.59					0.59								
LOC115536792	0.36													
LOC115536824											Gain	Gain		
LOC115536838						-0.64							K01068	Acyl-coenzyme A thioesterase 1/2/4
LOC115536841	-0.90													NLR family CARD domain-containing protein 3
LOC115536845						1.10								NLR family CARD domain-containing protein 3
LOC115536848				0.71										
LOC115536850	-0.53													ADP-ribosyl cyclase 1
LOC115536852											Gain			Nuclease HARBI1
LOC115536854			0.36											Palmitoyltransferase ZDHHC2/15/20
LOC115536863	0.34													
LOC115536887						0.59							K11255	Nuclear receptor coactivator 2
LOC115536892		0.64	0.51	0.73		0.58								
LOC115536894	0.80	0.71	0.96											Translocating chain-associated membrane protein 1
LOC115536904						0.71							K19530	Junctophilin
LOC115536907						-1.50								
LOC115536908												Loss		
LOC115536911						0.65								
LOC115536920			-0.58							Loss	Loss	Loss		CD22 antigen
LOC115536930												Gain		
LOC115536937						0.87								
LOC115536947			0.58										K08819	Cyclin-dependent kinase 12/13
LOC115536954	-0.37												K00814	Alanine transaminase
LOC115536960						-0.88	Gain						K20644	Rho gtpase-activating protein 29/45
LOC115536966	0.57													
LOC115536968			-0.55										K10258	Very-long-chain enoyl-coa reductase
LOC115536977						-0.64								
LOC115536986				-0.41		-1.05							K24524	Copine 1/2/3
LOC115536987						-0.39							K24524	Copine 1/2/3
LOC115537005	0.50					-0.79							K07375	Tubulin beta

LOC115537007						0.50							K10338	Ankyrin repeat and SOCS box protein 16
LOC115537017				0.47									K13882	Coronin-1A
LOC115537032						0.58								
LOC115537036						-0.93								
LOC115537039	-0.51													Endophilin-B1
LOC115537056						-0.50							K24744	WD repeat-containing protein 37
LOC115537061						0.77								NLR family CARD domain-containing protein 3
LOC115537062		0.54												NLR family CARD domain-containing protein 3
LOC115537066					1.11									
LOC115537076				-0.51		-0.62								
LOC115537077								Loss						
LOC115537078						0.62								
LOC115537079	-0.50													
LOC115537103						0.59							K15008	Discs, large-associated protein 1
LOC115537106			-0.42	0.36									K24348	UBX domain-containing protein 1/4
LOC115537151	0.48			-0.52		-0.51								
LOC115537154	0.46												K22137	Mitochondrial calcium uniporter regulator 1
LOC115537157							Gain							Putative transposase
LOC115537169	-0.56													
LOC115537190												Loss	K24360	Neuropilin and tolloid-like protein
LOC115537194						-0.62		Loss			Gain	Gain		Claudin
LOC115537205								Loss			Loss	Loss		
LOC115537226	-0.41												K01307	Gamma-glutamyl hydrolase
LOC115537227	0.56												K01672	Carbonic anhydrase
LOC115537228			-0.41											Voltage-dependent calcium channel alpha-2/delta-2
LOC115537235				0.46										Transferrin receptor
LOC115537241						-0.56							K23794	Pleckstrin homology-like domain family B
LOC115537246	-0.67	-0.33	-0.42	-0.37		-0.47								
LOC115537254			-0.54											
LOC115537255	-0.55		-1.01											
LOC115537256							Loss	Loss	Loss		Gain + Loss	Loss		
LOC115537257									Gain			Gain		Cytochrome c oxidase subunit 6a

LOC115537258	-3.30		-4.59											
LOC115537263	-0.93		-2.70											
LOC115537267	-4.75		-2.42											
LOC115537269								Gain						
LOC115537278						-0.66								Cytochrome b5
LOC115537291			-0.44										K18584	Actin-related protein 3
LOC115537294	0.27			-0.40		-0.52								Polypyrimidine tract-binding protein 2
LOC115537296						0.53								
LOC115537310						-0.84								Ras-responsive element-binding protein 1
LOC115537317						0.69							K09191	General transcription factor IIIA
LOC115537324	-0.71	-0.56	-0.75	-0.49										
LOC115537331			-0.43	-0.33		-0.36							K17494	Cysteine/serine-rich nuclear protein
LOC115537340				-0.47										
LOC115537346					0.80	4.21								
LOC115537349						0.62								Toll-like receptor 13
LOC115537352						-0.69								
LOC115537356	-0.43	-0.38	-0.54	-0.51		-0.48								
LOC115537375	0.30												K00161	Pyruvate dehydrogenase E1 component alpha subunit
LOC115537376						0.57								CD22 antigen
LOC115537379							Loss	Loss						Axin 1
LOC115537387						-0.85								
LOC115537391						-0.60								Aryl hydrocarbon receptor repressor
LOC115537393			0.60										K01880	Glycyl-trna synthetase
LOC115537409	-1.23	-0.69	-1.91											LIM domain and actin-binding protein 1
LOC115537411	-0.38		-0.43											TGF-beta receptor type-1
LOC115537418						-0.99							K04721	Tumor necrosis factor ligand superfamily member 10
LOC115537425	-3.46						Loss							
LOC115537427						-0.57							K13354	Solute carrier family 25 (peroxisomal adenine nucleotide transporter), member 17
LOC115537441									Loss				K13164	Male-specific lethal 2
LOC115537452			0.60										K01092	Myo-inositol-1(or 4)-monophosphatase
LOC115537453	0.47					-0.50								Polyhomeotic-like protein 3
LOC115537455						-2.44								Solute carrier family 7 (cationic amino acid transporter), member 14

LOC115537456				0.39										Guanine nucleotide-binding protein G(I)/G(S)/G(O) subunit gamma-13
LOC115537461				-0.43		-1.30								Neuropilin and tolloid-like protein
LOC115537475									Loss				K13963	Serpin B
LOC115537479			0.36										K18619	Coronin-7
LOC115537480							Gain	Gain						Toll-like receptor 13
LOC115537488						1.31							K23483	Gamma-crystallin
LOC115537491												Loss		
LOC115537492						-0.58								Voltage-dependent calcium channel L type alpha-1S
LOC115537500	-0.94	-0.65	-0.95											
LOC115537504	0.36			-0.46										Cytochrome c heme-lyase
LOC115537507				-0.52		-1.41							K09225	Zinc finger protein ZIC 4
LOC115537516								Gain			Gain	Gain	K01047	Secretory phospholipase A2
LOC115537521						-0.70								
LOC115537534												Gain	K04264	Thromboxane A2 receptor
LOC115537536						-0.54					Loss		K06802	Cadherin 10, type 2, T2-cadherin
LOC115537539	-0.84	-0.41	-0.49											
LOC115537547				-0.47		-0.37								
LOC115537554	-0.70		-0.68								Loss	Loss		3-hydroxybutyrate dehydrogenase
LOC115537565	-0.37		-0.59										K24478	Ankyrin repeat and fibronectin type-III domain-containing protein 1
LOC115537566						-0.80								
LOC115537569							Loss							Diamine N-acetyltransferase
LOC115537572										Gain	Gain	Gain	K08446	CD97 antigen
LOC115537576			0.39											
LOC115537581	-0.46			-0.64		-1.25								
LOC115537586	-0.30		-0.30										K13627, K23967	Solute carrier family 12 (potassium/chloride transporter), member 5, solute carrier family 12 (potassium/chloride transporter), member 7
LOC115537587						-0.69								
LOC115537591						2.57							K07374	Tubulin alpha
LOC115537609	0.35			0.43										6-phosphofructokinase 1
LOC115537620												Gain		
LOC115537621						0.48							K03236	Translation initiation factor 1A
LOC115537624						0.55								THAP domain-containing protein 6

LOC115537632	-0.53													
LOC115537633	0.35													General transcription factor IIIA, zinc finger and BTB domain-containing protein 40
LOC115537636	-0.51													
LOC115537650				2.34									K06467	CD22 antigen
LOC115537656						-0.51								Zinc finger protein GLI3
LOC115537663											Gain			
LOC115537672						0.79								A-kinase anchor protein 2
LOC115537681						-0.44							K10369	Supervillin
LOC115537682				0.43		0.91								
LOC115537690	-0.58													NLR family CARD domain-containing protein 3
LOC115537694	-0.82													
LOC115537707	0.65												K03098	Apolipoprotein D and lipocalin family protein
LOC115537708						-0.62								Agouti related protein
LOC115537709						1.47								
LOC115537710						0.60								
LOC115537713						0.73								Forkhead box protein K
LOC115537737				0.37		1.34								
LOC115537782				-0.71										
LOC115537790				0.76										NLR family CARD domain-containing protein 3
LOC115537824						-0.70								
LOC115538007	-0.78		-0.84											L-asparaginase / beta-aspartyl-peptidase
LOC115538009						0.49								Cathepsin D
LOC115538021	0.71	0.45				0.63								Chondroitin 4-sulfotransferase 12
LOC115538025						0.59								
LOC115538067											Gain			
LOC115538078							Loss							
LOC115538122												Gain		
LOC115538149							Loss							
LOC115538206						0.78							K22614	NLR family CARD domain-containing protein 3
LOC115538208				0.41										
LOC115538212						0.98					Loss	Loss		NLR family CARD domain-containing protein 3
LOC115538213	-0.51													NLR family CARD domain-containing protein 3

LOC115538221						0.58								NLR family CARD domain-containing protein 3
LOC115538223						0.71						Gain		
LOC115538229									Gain		Gain			
LOC115538241	1.38	0.52	1.37											
LOC115538244						0.38								
LOC115538253	-0.70		-0.43			0.42								
LOC115538256	0.49												K00222, K19532	Delta14-sterol reductase , Delta14-sterol reductase (lamin-B receptor)
LOC115538257						-0.45								
LOC115538258	0.52													
LOC115538261				-0.50										
LOC115538262												Gain		MHC class I antigen
LOC115538267								Loss	Loss			Loss		Solute carrier family 26 (sulfate anion transporter), member 6
LOC115538276							Gain		Gain					
LOC115538415							Gain		Gain					
LOC115538418		0.48												Kinesin family member 5
LOC115538420						0.74								
LOC115538429												Loss		Tumor necrosis factor ligand superfamily member 13B
LOC115538433	0.52													
LOC115538440									Gain		Gain	Loss		
LOC115538455				0.56		0.76								Syntaxin-binding protein 5
LOC115538461											Loss	Loss	K04258	Prostaglandin E receptor 1
LOC115538465												Gain		
LOC115538477											Gain		K23560	Zinc finger homeobox protein 2
LOC115538651	-0.76					0.56							K06711	Hypoxia-inducible factor prolyl 4-hydroxylase
LOC115538696											Gain	Gain		
LOC115538711						-0.62								
LOC115538775				-0.43										
LOC115538792				-0.49		-0.81								
LOC115538818												Loss		
LOC115538874						0.59								
LOC115538880	1.57	0.89	1.61										K23408	Cell division cycle-associated protein 7
LOC115538885											Loss	Loss		

LOC115538891				0.44										
LOC115538892												Loss	K12230	Phosphoinositide 3-kinase adapter protein 1
LOC115538898				0.40		0.67								
LOC115538902				0.67		0.78							K17914	Kinesin family member 13
LOC115538907				-0.73		-1.52								
LOC115538930	-0.73		-0.50											Hepatic leukemia factor
LOC115538936			0.38											Vesicle-fusing atpase
LOC115538941				0.58									K05009	Potassium inwardly-rectifying channel subfamily J member 16
LOC115538942						0.47								Potassium inwardly-rectifying channel subfamily J member 2
LOC115538945							Gain							
LOC115538948						0.55								Cbp/p300-interacting transactivator 4
LOC115538952	-18.07													
LOC115538958			-0.52											Nuclear distribution protein nude-like 1
LOC115538971	-1.29					-0.95								F-type H+-transporting atpase subunit d
LOC115538985											Loss	Loss		
LOC115538989	0.55	0.86				0.67								
LOC115538997											Loss	Loss		
LOC115539001						0.76								Gamma-crystallin
LOC115539015									Loss		Loss	Loss	K17455	Fascin2
LOC115539032						0.66							K09103	Early B-cell factor
LOC115539051						-0.91							K25185	Metalloprotease TIKI
LOC115539060				0.42										Alkyldihydroxyacetonephosphate synthase
LOC115539083				0.34										NLR family CARD domain-containing protein 3
LOC115539118								Gain				Loss		
LOC115539137												Gain		
LOC115539170						-0.51								
LOC115539171						-0.70								
LOC115539230											Loss	Loss		
LOC115539252						0.74								
LOC115539279				0.40		0.68								
LOC115539281											Gain	Gain		Tumor necrosis factor receptor superfamily member 14
LOC115539282				0.33		0.89								
LOC115539283				0.47										

LOC115539285	-0.77													
LOC115539287				0.44										
LOC115539293				0.58										
LOC115539296				0.32	0.83									
LOC115539306							Loss	Loss	Loss					
LOC115539349	0.61												K15208	Snrna-activating protein complex subunit 1
LOC115539350						1.08								NLR family CARD domain-containing protein 3
LOC115539352						-0.58								MHC class I antigen
LOC115539364												Loss		MHC class I antigen
LOC115539371						-0.92								
LOC115539399	-0.55			-0.75		-1.20		Gain						
LOC115539403							Loss	Loss	Loss			Loss	K07497	Putative transposase
LOC115539412	0.60	0.69	0.63											Peptide chain release factor subunit 3
LOC115539414	-0.41													
LOC115539415											Loss	Loss		
LOC115539443						-0.71							K09455	Microphthalmia-associated transcription factor
LOC115539471	0.62					0.65								
LOC115539476						0.82							K09850	Ras association domain-containing protein 1
LOC115539692	-1.77	-0.64	-1.54											ATP-binding cassette, subfamily A (ABC1), member 1, ATP-binding cassette, subfamily A (ABC1), member 3
LOC115539697	0.63					-0.66								Collagen type IX alpha
LOC115539702	0.82		0.68											
LOC115539714	-0.62		-0.55										K05069	Interleukin 2 receptor beta
LOC115539723	-0.40		-0.45											Arginine-glutamic acid dipeptide repeats protein
LOC115539726	-0.69		-0.51			0.92								
LOC115539732	0.77													
LOC115539734	-0.57													
LOC115539736						-0.60								
LOC115539738						0.67							K07604	Type I keratin, acidic
LOC115539741											Gain		K12486	Stromal membrane-associated protein
LOC115539749	-0.49		-0.64											WW domain-binding protein 1
LOC115539753						0.47								Type I keratin, acidic
LOC115539768							Loss		Loss					

LOC115539779	0.35		0.33	0.42		0.35								Interleukin enhancer-binding factor 3
LOC115539781						-0.53								Solute carrier family 44 (choline transporter-like protein), member 2/4/5
LOC115539787	-0.62	-0.89	-1.02			-0.60								Nuclear factor, interleukin 3 regulated
LOC115539791	-0.51					-0.49								
LOC115539795						0.77					Gain	Gain		Htra serine peptidase 3
LOC115539797				-0.61		-0.57	Loss				Loss	Gain + Loss		Prostaglandin E receptor 1
LOC115539798	-0.81													
LOC115539803		-0.42					Gain	Gain			Gain + Loss		K08411	G protein-coupled receptor 26
LOC115539819							Loss	Loss	Gain					
LOC115539834						0.82								
LOC115539840						-0.59								Hairy and enhancer of split 5
LOC115539857	0.50						Gain						K17710	Pentatricopeptide repeat domain-containing protein 1
LOC115539858						0.48								
LOC115539859						0.42								Mitochondrial dynamics protein MID51/49
LOC115539861	-0.40	-0.35	-0.48			0.41							K13751	Solute carrier family 24 (sodium/potassium/calcium exchanger), member 3
LOC115539865	0.44												K17493	Calmodulin-regulated spectrin-associated protein
LOC115539866							Gain							
LOC115539868						-0.45								Bromodomain-containing protein 4
LOC115539884						0.43							K22641	Protein tweety
LOC115539901				0.39										Coiled-coil domain-containing protein 50
LOC115539903						-0.92								CD97 antigen
LOC115539909				-0.32										
LOC115539910								Gain						
LOC115539915			-0.40											
LOC115539952				-0.56										Solute carrier family 9B (sodium/hydrogen exchanger), member 1/2
LOC115539962						-1.19								Krueppel-like factor 3
LOC115539969	-0.89	-0.53	-0.49											ESCRT-I complex subunit VPS37
LOC115539970	-0.47													
LOC115539990								Loss			Loss	Loss	K05718	Integrin alpha L
LOC115539992	0.38													

LOC115539995				0.57		0.30								Ribosomal protein S6 kinase beta
LOC115539996						0.95								Type I keratin, acidic
LOC115540012	-0.54	-0.48	-0.93			-0.51								
LOC115540015						-0.46							K21290	Phosphoinositide-3-kinase regulatory subunit 5/6
LOC115540019	-0.79		-0.44											
LOC115540025						-0.92								Nidogen (entactin)
LOC115540032						0.73							K18162	NADH dehydrogenase
LOC115540060	-1.00	-0.48	-1.23			-0.58								Glycerophosphoinositol glycerophosphodiesterase , glycerophosphoryl diester phosphodiesterase
LOC115540062						0.56							K07605	Type II keratin, basic
LOC115540074	0.39	0.33	0.66											Ribosome-binding protein 1
LOC115540075						0.33							K21989	Calcium permeable stress-gated cation channel
LOC115540076						-0.66							K07605	Type II keratin, basic
LOC115540084	-1.40	-0.45	-0.56										K08538	Farnesoid X receptor beta
LOC115540086								Loss			Loss	Loss		
LOC115540092												Loss	K01512	Acylphosphatase
LOC115540096						-0.46							K22383	Interferon regulatory factor 2-binding protein
LOC115540104						0.56								
LOC115540105						-0.68								3-oxo-5-alpha-steroid 4-dehydrogenase 2
LOC115540106						-0.37								Serine/threonine-protein phosphatase 2B regulatory subunit
LOC115540110						0.87								
LOC115540111	-0.55												K15107	Solute carrier family 25 (mitochondrial glutamate transporter), member 18/22
LOC115540113						-0.39								
LOC115540121						-0.48								Solute carrier family 44 (choline transporter-like protein), member 1
LOC115540122											Loss	Loss	K04001	C1 inhibitor
LOC115540123								Gain			Gain	Gain	K08136	Testican
LOC115540129	-0.48		-0.52										K17354	Tetraspanin-2
LOC115540144	-1.76	-0.40	-1.53											Programmed cell death protein 4
LOC115540145		-0.52											K23708	Sorbin and SH3 domain-containing protein 2
LOC115540146				0.43		0.91							K07293	Tyrosine-protein phosphatase non-receptor type 11
LOC115540150												Loss		
LOC115540155				-0.31										Dnaj homolog subfamily A member 3

LOC115540156				-0.32		-1.37						Loss		
LOC115540171	0.40	0.32	0.42										K12820	Pre-mrna-splicing factor ATP-dependent RNA helicase DHX15/PRP43
LOC115540178	0.31													Cyclic AMP-dependent transcription factor ATF-4
LOC115540179								Loss			Gain	Gain		
LOC115540180							Loss	Loss	Loss		Loss			
LOC115540183						0.38								Oligosaccharyltransferase complex subunit gamma
LOC115540184									Loss					
LOC115540189	0.59	0.62	0.70											Transportin-1, transportin-2
LOC115540191						0.57								
LOC115540196	0.53													
LOC115540204							Loss	Loss			Gain	Gain + Loss		
LOC115540207												Gain	K15118	Solute carrier family 25, member 38
LOC115540210	-1.31	-1.08	-1.64											Serine/threonine-protein kinase/endoribonuclease IRE1
LOC115540211	-1.02													
LOC115540215		-0.40	-0.51											Camp-specific phosphodiesterase 4
LOC115540218	0.58		0.43			-0.59					Loss	Loss		General transcription factor IIIA, KRAB domain-containing zinc finger protein
LOC115540221	-0.36	-0.46	-0.63										K11588	Methyl cpg binding protein 2
LOC115540235				-0.40		-0.45								
LOC115540237						0.67							K10276	F-box and leucine-rich repeat protein 10/11
LOC115540244	-1.06	-0.41	-1.32											Heme oxygenase (biliverdin-producing, ferredoxin) , heme oxygenase 1
LOC115540245	-1.07	-0.43	-0.78	0.38										E3 ubiquitin-protein ligase RNF11
LOC115540250				-0.64		-1.06								
LOC115540268						0.57								L1 cell adhesion molecule
LOC115540281						0.57								2-epi-5-epi-valiolone synthase
LOC115540287				-0.36		-0.86								Protein tyrosine phosphatase type IVA
LOC115540301	-0.35													TBC1 domain family member 1
LOC115540302	0.64		0.57										K22823	Non-structural maintenance of chromosomes element 3
LOC115540315						-0.55							K16453	Centrosomal protein CEP110
LOC115540321						0.58								MAX dimerization protein
LOC115540333							Loss				Gain	Gain		
LOC115540335			-0.48			0.83							K08836	STE20-like kinase

LOC115540355			-1.01	0.41									K06867, K10380	Ankyrin, uncharacterized protein
LOC115540357												Loss	K03990	Complement component 3
LOC115540358						-1.12								
LOC115540361			-1.34											
LOC115540365						0.77								
LOC115540393	0.75	0.45	0.57										K15014	Solute carrier family 29 (equilibrative nucleoside transporter), member 1/2/3
LOC115540397							Loss						K04224	Tachykinin receptor 3
LOC115540411	0.36											Gain	K24473	Teneurin
LOC115540413	-0.55		-0.49										K01187, K12316	Alpha-glucosidase , lysosomal alpha-glucosidase
LOC115540416						0.78							K17305	Beta-2-glycoprotein 1
LOC115540417	0.24													A-kinase anchor protein 8-like protein
LOC115540426											Loss	Loss		Platelet/endothelial cell adhesion molecule
LOC115540431	0.54													Delta
LOC115540437				-0.67									K16454	Centriole, cilia and spindle-associated protein
LOC115540438											Loss	Loss		
LOC115540441	0.34												K12881	THO complex subunit 4
LOC115540443			0.54											Rho GDP-dissociation inhibitor
LOC115540444	-1.25	-0.96	-1.68	-0.50										Protein phosphatase 1 regulatory subunit 27
LOC115540452	-0.55												K09114	MAX dimerization protein
LOC115540462						0.62							K26118	Atpase family AAA domain-containing protein 5
LOC115540465	-0.38		-0.39											
LOC115540474						0.92	Gain					Loss	K06777	Receptor-type tyrosine-protein phosphatase delta
LOC115540498							Loss	Loss	Loss		Gain			
LOC115540503		-0.41												
LOC115540506	-0.41		-0.47									Gain	K05084	Receptor tyrosine-protein kinase erbb-3
LOC115540508								Gain			Gain	Loss	K15291	Regulating synaptic membrane exocytosis protein 1
LOC115540509				0.43		0.61								
LOC115540511						0.60		Loss	Gain		Gain	Gain		Protocadherin delta 2
LOC115540514	0.64	0.50	0.47											
LOC115540516						-0.49								Catenin (cadherin-associated protein), delta 1
LOC115540521						0.74								

LOC115540522	0.83	0.51	0.63										K20792	N-alpha-acetyltransferase 15/16, nata auxiliary subunit
LOC115540524									Loss		Gain	Gain		
LOC115540525	0.54		0.41			0.58								Calmegin, calnexin
LOC115540530			-0.45	0.38										E74-like factor 1/2/4
LOC115540531												Gain		Tensin
LOC115540533	-0.94		-0.37			-0.79							K14387	Solute carrier family 5 (high affinity choline transporter), member 7
LOC115540547						-0.92								Transcription factor Sp6
LOC115540550						4.36							K23482	Beta-crystallin
LOC115540551						0.76							K23482	Beta-crystallin
LOC115540553						0.44								
LOC115540561								Gain					K04930	Potassium voltage-gated channel KQT-like subfamily member 5
LOC115540562								Gain						Von Willebrand factor A domain-containing protein 7
LOC115540568			-0.27											Transcription elongation factor 1
LOC115540572						0.61							K09068, K15604	Protein lyl-1, T-cell acute lymphocytic leukemia protein
LOC115540584			0.57											
LOC115540601	-0.29			-0.36							Gain			General transcription factor IIIA
LOC115540611	0.31		0.35			0.31							K14405	Pre-mrna 3'-end-processing factor FIP1
LOC115540616	-0.45		-0.42											Phosphatidylinositol 4-kinase type 2
LOC115540622	0.66	0.48												
LOC115540624				-1.28		-2.49								
LOC115540633						0.94							K04594	Adhesion G protein-coupled receptor L3
LOC115540643	-1.12													
LOC115540648						-0.47							K20640	Rho gtpase-activating protein 19
LOC115540653	0.61													Fanconi anemia group E protein
LOC115540661						-0.51							K09233	Zinc finger protein GLIS2
LOC115540662				-1.15		-0.88		Gain + Loss						Beta-1,3-galactosyltransferase 2
LOC115540663	0.97	0.73	0.89										K14290	Exportin-1
LOC115540668											Gain		K04456	RAC serine/threonine-protein kinase
LOC115540670						-0.63								
LOC115540671							Loss					Loss		
LOC115540673								Gain			Gain	Loss		

LOC115540679	0.35			-0.36		-0.65							K07928	Ras-related protein Rab-40
LOC115540686						-0.94								
LOC115540697	-0.43					-0.67							K04542	Guanine nucleotide-binding protein G(I)/G(S)/G(O) subunit gamma-5
LOC115540698						0.88							K00286	Pyrroline-5-carboxylate reductase
LOC115540701	-0.44													
LOC115540702	-0.72		-0.75			0.58								Stromal interaction molecule 2
LOC115540703						-0.60								
LOC115540712						-0.81								
LOC115540725	-0.98													Cytochrome P450 family 3 subfamily A
LOC115540728			0.51										K23781	Arf-GAP with dual PH domain-containing protein
LOC115540729			0.52										K21764	Sperm-associated antigen 5
LOC115540739	0.43		0.49										K14006	Protein transport protein SEC23
LOC115540741	0.36		0.50										K12741	Heterogeneous nuclear ribonucleoprotein A1/A3
LOC115540746												Loss	K04703	Sprouty-related, EVH1 domain-containing protein
LOC115540764						-0.85								Glutamate receptor 2
LOC115540766	0.63		0.66											
LOC115540780				0.41										
LOC115540793						0.63								KRAB domain-containing zinc finger protein
LOC115540794						0.68								Transmembrane protein 132
LOC115540806				0.42										Tripartite motif-containing protein 25
LOC115540807									Gain				K05718	Integrin alpha L
LOC115540808	0.60	0.53	0.56								Gain			
LOC115540814				0.46										
LOC115540817												Loss		
LOC115540839						0.55								
LOC115540846				-0.48		-0.75							K11247	Endophilin-A
LOC115540851						-0.55							K04385	Axin 2
LOC115540853	-0.63												K21554	CREB3 regulatory factor
LOC115540854	0.39													Solute carrier family 25 (mitochondrial iron transporter), member 28/37
LOC115540858						0.58								Nuclease HARBI1
LOC115540863	0.69		0.68											
LOC115540880						-0.84								Sphingosine 1-phosphate receptor 5

LOC115540881						0.77								
LOC115540890						-0.67							K09341	Homeobox protein MSX
LOC115540892				-0.47		-0.77								
LOC115540894		-0.64	-0.66											
LOC115540895		-0.34	-0.39											
LOC115540896		0.55	0.69											
LOC115540899				-0.43		-0.85	Loss	Loss	Loss					Deleted in malignant brain tumors 1 protein
LOC115540918	0.41												K06174	ATP-binding cassette, sub-family E, member 1
LOC115540953				-1.78		-3.27								
LOC115540956						-0.49								Wolfamin
LOC115540986	1.19					1.56							K09863	Aquaporin-0
LOC115540988	-0.77		-0.59											Tripartite motif-containing protein 16
LOC115540989	-0.50		-0.37	0.43									K04992	Mucolipin 1
LOC115540990	0.48		0.51	0.60									K12006	Tripartite motif-containing protein 16
LOC115540992				0.44										Sphingosine kinase
LOC115540997								Gain	Gain					
LOC115540999	-0.35		-0.59			0.47								Insulin-like growth factor-binding protein 10
LOC115541002	-0.47												K00948	Ribose-phosphate pyrophosphokinase
LOC115541010								Loss	Loss				K09223	Growth factor independent 1
LOC115541026						-0.71							K04583	Glucagon receptor
LOC115541029						0.66							K08179	MFS transporter, MCT family, solute carrier family 16 (monocarboxylic acid transporters), member 1
LOC115541031						0.66								Galectin-1
LOC115541032				0.52		0.76								TBC1 domain family member 24
LOC115541042						-0.80								
LOC115541046	-0.40	-0.44	-0.45										K02187	Caspase 3
LOC115541051						-0.57								Leucine-rich repeat LGI family member 2
LOC115541058	-0.61		-0.48										K15699	RING finger protein 122
LOC115541070						0.68							K05210	Glutamate receptor ionotropic, NMDA 2B
LOC115541073							Gain				Loss	Loss	K12473	Low-density lipoprotein receptor
LOC115541075	-0.40												K01897	Long-chain acyl-coa synthetase
LOC115541076			-0.44			-0.49							K19347	SUN domain-containing protein 1/2
LOC115541087						-0.36					Gain		K14946	RNA binding protein fox-1

LOC115541091	0.53	0.55	0.53											
LOC115541099	-0.52		-0.81	0.52		0.84								
LOC115541107						-0.73						Loss		
LOC115541119								Loss						
LOC115541126	-0.61		-0.51			0.39							K10303, K17345	F-box protein 23, tetraspanin-5
LOC115541131											Loss		K19882	O-palmitoleoyl-L-serine hydrolase
LOC115541152						-0.85								Natural resistance-associated macrophage protein 2
LOC115541220						0.50							K07374	Tubulin alpha
LOC115541252	-0.86	-0.61	-0.96											Carboxy-terminal domain RNA polymerase II polypeptide A small phosphatase
LOC115541272						-0.56								Netrin receptor unc-5
LOC115541275	-0.57													
LOC115541282		-0.39	-0.43											Transmembrane channel-like protein
LOC115541283											Loss			
LOC115541300				-0.36										General transcription factor IIIA
LOC115541301				0.40										
LOC115541304	-0.73		-0.57										K09191, K09228	General transcription factor IIIA, KRAB domain-containing zinc finger protein
LOC115541306						-0.70								
LOC115541309												Loss		
LOC115541313											Loss	Loss		
LOC115541315								Loss						
LOC115541316							Loss							
LOC115541335						0.75								
LOC115541340				1.32		0.44								
LOC115541343				-0.43		-0.87		Gain					K04402	Growth arrest and DNA-damage-inducible protein
LOC115541346											Gain			
LOC115541349											Loss		K00515	Beta-carotene 15,15'-dioxygenase
LOC115541359				0.52		0.73								Small G protein signaling modulator 1
LOC115541360						-0.97							K12487	G protein-coupled receptor kinase interactor 2
LOC115541367						0.55								
LOC115541374									Loss					
LOC115541385											Gain	Gain		

LOC115541386				-0.60									K03926	Periplasmic divalent cation tolerance protein
LOC115541389										Gain		Gain		
LOC115541396							Gain	Gain				Gain	K08375	Neuropeptide FF receptor 2
LOC115541412						0.65	Loss			Loss	Loss		K04437	Filamin
LOC115541415												Loss	K09191, K09228	General transcription factor IIIA, KRAB domain-containing zinc finger protein
LOC115541439						0.63								NLR family CARD domain-containing protein 3
LOC115541441						-1.59							K04732	Interleukin-1 receptor-associated kinase 3
LOC115541444											Gain	Gain		
LOC115541453						0.51								Ethanolamine kinase
LOC115541466						-0.77								
LOC115541470	0.68	0.40	0.59									Gain		Protein SCO1, protein SCO2
LOC115541471	-0.76		-0.56										K04695	Suppressor of cytokine signaling 2
LOC115541474											Gain	Gain		
LOC115541476						-0.57								Tetraspanin-9
LOC115541479						-0.61								
LOC115541481	-0.58	-0.33	-0.74										K07766	Diphosphoinositol-polyphosphate diphosphatase
LOC115541485										Gain	Gain	Gain	K20161	DENN domain-containing protein 2
LOC115541488				1.65										
LOC115541489						0.61								Ubiquitin-conjugating enzyme E2 N
LOC115541494	-0.64		-1.32											Serine/threonine-protein kinase SBK
LOC115541500											Loss	Loss		
LOC115541512	0.43													Proto-oncogene serine/threonine-protein kinase Pim-1 , proto-oncogene serine/threonine-protein kinase Pim-3
LOC115541514				0.37										
LOC115541517						-0.82								
LOC115541520								Gain	Gain		Loss			
LOC115541523				0.41										Hexosaminidase
LOC115541524						-0.68								Hexosaminidase
LOC115541532				0.41									K06453	CD3Z antigen, zeta polypeptide
LOC115541533						-0.46					Gain	Gain		
LOC115541542						-0.64							K00016	L-lactate dehydrogenase
LOC115541544	0.43										Loss		K25694	Spexin
LOC115541549	0.42					-0.53							K00021	Hydroxymethylglutaryl-coa reductase (NADPH)

LOC115541550						0.47							K08283	Collagen type IV alpha-3-binding protein
LOC115541557				0.40		0.73								
LOC115541561	-1.14	-0.97	-1.40										K05080	Growth hormone receptor
LOC115541563		-0.38												
LOC115541568						-0.46								
LOC115541574							Gain		Gain				K22376	E3 ubiquitin-protein ligase KCMF1
LOC115541581	0.70	0.39	0.44											Periodic tryptophan protein 2
LOC115541583											Loss	Loss	K06556	CD200 antigen
LOC115541584	0.44												K01930	Folylpolyglutamate synthase
LOC115541590						-0.96							K17477	FERM, rhogef and pleckstrin domain-containing protein 1
LOC115541591						0.67								
LOC115541593	-0.76		-0.98										K02649	Phosphoinositide-3-kinase regulatory subunit alpha/beta/delta
LOC115541596						-0.67							K03844	Alpha-1,2-mannosyltransferase
LOC115541602						0.67								
LOC115541605						-0.39								
LOC115541608						0.61								General transcription factor IIIA
LOC115541610											Gain		K09293	Paired box protein 8
LOC115541614						-0.27							K24775	WW domain-binding protein 1
LOC115541641												Gain		
LOC115541647							Gain	Gain	Gain		Gain	Gain + Loss		Nuclease HARBI1
LOC115541650												Loss		Fibronectin type III domain-containing protein 4/5
LOC115541661								Gain					K07294	Peroxisome proliferator-activated receptor alpha
LOC115541667							Loss					Gain		
LOC115541695								Gain						
LOC115541713						-0.85					Loss	Loss	K08809	Striated muscle-specific serine/threonine protein kinase
LOC115541736						0.57								Additional sex combs-like protein
LOC115541747	-1.04	-0.79	-0.89											
LOC115541750							Loss							
LOC115541757						-0.88								
LOC115541759						-0.52								
LOC115541763						0.61								NLR family CARD domain-containing protein 3
LOC115541765						-0.53								General transcription factor IIIA, KRAB domain-containing zinc finger protein

LOC115541768						0.64							K23219	Methyl-cpg-binding domain protein 5
LOC115541780						0.79								
LOC115541792						0.37							K11406	Histone deacetylase 4/5
LOC115541796			0.38											
LOC115541798						0.59								Janus kinase 2
LOC115541799						-0.61			Loss				K10505	Zinc finger and BTB domain-containing protein 26
LOC115541800						-1.16								Relaxin 3
LOC115541803								Loss					K15188	Cyclin T
LOC115541809						0.87								
LOC115541810												Gain		
LOC115541817				0.45									K23797	Pleckstrin homology domain-containing family A member 4/5/6/7
LOC115541818				1.74									K25552	Cilia- and flagella-associated protein 47
LOC115541820	-0.38					-0.81							K21853	Dedicator of cytokinesis protein 9/10/11
LOC115541830	-0.44												K22808	KN motif and ankyrin repeat domain-containing protein
LOC115541838			0.32										K16574	Mitotic-spindle organizing protein 2A/2B
LOC115541840	0.65		0.63											
LOC115541847						-0.67							K00624	Carnitine O-acetyltransferase
LOC115541862						0.60								
LOC115541864	-0.53		-0.32											Serine/threonine-protein kinase ULK1 , serine/threonine-protein kinase ULK2
LOC115541865			0.59											
LOC115541877						0.60							K04722	Tumor necrosis factor receptor superfamily member 10A/B
LOC115541883	-0.66												K11426	
LOC115541885		-0.42												LHFPL tetraspan subfamily member protein
LOC115541886				0.60		0.81								
LOC115541898				0.45										
LOC115541905	0.64												K20910	Thioredoxin-interacting protein
LOC115541906	-0.52					1.92							K24220	Myosin heavy chain 1/2/3/4/8/13/7B/15
LOC115541913						-0.54								
LOC115541915	0.65					-0.60								
LOC115541933	0.52													Pleckstrin homology-like domain family A
LOC115541935				0.56									K08014	Rap guanine nucleotide exchange factor 3
LOC115541940									Loss					Activin receptor type-2A

LOC115541941												Gain		
LOC115541947			-0.41											AN1-type zinc finger protein 5/6
LOC115541949			0.44										K11447	Lysine-specific demethylase 6A
LOC115541953			-0.75											
LOC115541956						-0.40							K24099	LIM domain transcription factor LMO4
LOC115541961	-1.08												K07297	Adiponectin receptor
LOC115541966						0.66								Tyrosine-protein phosphatase non-receptor type 12/18/22
LOC115541967				-0.46										
LOC115541974			0.55										K04348	Serine/threonine-protein phosphatase 2B catalytic subunit
LOC115541980	0.42		0.27	-0.30									K13201	Nucleolysin TIA-1/TIAR
LOC115541984						-0.50								ATP-binding cassette, subfamily C (CFTR/MRP), member 4
LOC115541986						-0.64	Gain	Gain						
LOC115541987						-1.08								
LOC115541997				-0.46		-0.70								MADS-box transcription enhancer factor 2C
LOC115542012						-0.60							K24465	Epidermal growth factor-like protein 7/8
LOC115542014				-0.55										1-acyl-sn-glycerol-3-phosphate acyltransferase , lysophosphatidate acyltransferase
LOC115542023	0.51													
LOC115542027						0.80								Ras-associated and pleckstrin homology domains-containing protein 1
LOC115542028	0.52													Phosphoribosylaminoimidazole carboxylase / phosphoribosylaminoimidazole-succinocarboxamide synthase
LOC115542035											Loss	Loss		FYN binding protein
LOC115542037						1.15								
LOC115542047	0.69		0.50										K11290	Template-activating factor I
LOC115542049	0.83		0.51										K14684	Solute carrier family 25 (mitochondrial phosphate transporter), member 23/24/25/41
LOC115542050	-0.52		-0.47	-0.57		-1.11							K00972	UDP-N-acetylglucosamine/UDP-N-acetylgalactosamine diphosphorylase
LOC115542052						-0.75								Nuclear apoptosis-inducing factor 1
LOC115542055	-0.67		-0.54				Gain							Interleukin 6 signal transducer
LOC115542063				0.54		0.52							K20520	Drebrin-like protein
LOC115542064						-0.66							K02265	Cytochrome c oxidase subunit 5b
LOC115542065	0.54		0.59										K02265	Cytochrome c oxidase subunit 5b
LOC115542067	0.40	0.32	0.50											Centractin

LOC115542089	0.40		0.31											Mitogen-activated protein kinase kinase kinase kinase 4
LOC115542090	-0.54		-0.48	-0.42		-0.73								Ribonuclease ZC3H12
LOC115542093							Loss					Loss	K18977	Hemopexin
LOC115542114	-0.69		-0.44										K07604	Type I keratin, acidic
LOC115542117				0.46										Rod cgmp-specific 3',5'-cyclic phosphodiesterase subunit beta
LOC115542156	0.41												K01641	Hydroxymethylglutaryl-coa synthase
LOC115542159			0.56			-0.69								Collagen type XXVIII alpha
LOC115542170												Loss		
LOC115542175	-0.51		-0.44			-0.90								Ras association domain-containing protein 7/8
LOC115542190				0.52		0.77								
LOC115542192		0.30												Protein TASOR
LOC115542200												Loss	K17591	RIMS-binding protein 2
LOC115542201						-0.74								
LOC115542217							Loss		Loss				K05614	Solute carrier family 1 (glial high affinity glutamate transporter), member 3
LOC115542219	-1.35	-0.48												
LOC115542222	-1.22	-0.52	-1.42	0.45										E3 ubiquitin-protein ligase RNF180
LOC115542230	-0.27		-0.29											Ras association domain-containing protein 3
LOC115542231	-0.58													Adipocyte plasma membrane-associated protein
LOC115542233			-0.47										K10336	Ankyrin repeat and SOCS box protein 14
LOC115542238	-1.23	-0.60	-1.73										K00318	Proline dehydrogenase
LOC115542239	-0.43		-0.62										K04577	Calcitonin receptor-like
LOC115542240						0.59							K11422	
LOC115542244						-0.56							K16056	Calcium release-activated calcium channel protein 1
LOC115542253	0.62		0.71											
LOC115542255	0.65	0.46	0.59											
LOC115542260						-0.46								Nidogen (entactin)
LOC115542261						0.74							K12168	E3 ubiquitin-protein ligase HECW2
LOC115542262				0.32										CLIP-associating protein 1/2
LOC115542266	0.36												K23718	Protein SON
LOC115542268							Gain							Phospholipid-translocating atpase
LOC115542271	-2.21	-1.10	-2.46			-0.71								Insulin receptor substrate 2
LOC115542272			-0.64				Gain				Gain		K05871	Focal adhesion kinase 2

LOC115542275						0.70							K13710	Rho guanine nucleotide exchange factor 7
LOC115542280						0.50							K09191, K09228	General transcription factor IIIA, KRAB domain-containing zinc finger protein
LOC115542291												Gain		Cytochrome P450 family 4 , docosahexaenoic acid omega-hydroxylase
LOC115542292	-0.53										Loss	Loss		
LOC115542293				0.51		0.82								
LOC115542326												Loss		
LOC115542331	-0.91		-1.05											Integral membrane protein GPR137
LOC115542343							Gain				Loss		K09191, K09228	General transcription factor IIIA, KRAB domain-containing zinc finger protein
LOC115542347						-0.59							K22687	Inhibin beta B chain
LOC115542348						0.91								NLR family CARD domain-containing protein 3
LOC115542357	-0.46	-0.70	-0.69	-0.71		-1.24								
LOC115542374						0.79								
LOC115542379				-0.35										
LOC115542382						0.78								
LOC115542396												Loss		NLR family CARD domain-containing protein 3
LOC115542401				-3.27										
LOC115542408						0.81								
LOC115542434						0.55							K03097	Casein kinase II subunit alpha
LOC115542463	-0.50												K00758	Thymidine phosphorylase
LOC115542472				-0.44		-0.78								SIN3-HDAC complex-associated factor
LOC115542477						0.68								Interleukin 22
LOC115542486						0.61						Loss		Tenascin
LOC115542487						0.71		Gain			Loss			
LOC115542506				-0.62		-0.74								Thrombospondin 2/3/4/5
LOC115542526						0.71							K24220	Myosin heavy chain 1/2/3/4/8/13/7B/15
LOC115542530		-1.33	-1.85										K24220	Myosin heavy chain 1/2/3/4/8/13/7B/15
LOC115542532		-0.20											K24220	Myosin heavy chain 1/2/3/4/8/13/7B/15
LOC115542536		-0.44	-0.51			0.62							K24220	Myosin heavy chain 1/2/3/4/8/13/7B/15
LOC115542538						-0.66							K24220	Myosin heavy chain 1/2/3/4/8/13/7B/15
LOC115542557						0.45							K24220	Myosin heavy chain 1/2/3/4/8/13/7B/15
LOC115542558						0.93							K24220	Myosin heavy chain 1/2/3/4/8/13/7B/15

LOC115542560		-0.42				0.67								
LOC115542598	-0.54												K18080	Tensin
LOC115542600						-0.41								
LOC115542611				0.71		1.16								
LOC115542616	-0.54												K20411	Proline-rich protein 5
LOC115542621				0.94										Claudin
LOC115542622						0.65								Interleukin 1 receptor accessory protein-like
LOC115542631						-0.47								Midline 1
LOC115542633				0.39										NLR family CARD domain-containing protein 3
LOC115542636				-0.44										Purine-rich element-binding protein gamma
LOC115542642										Gain	Gain			
LOC115542660						-0.66								
LOC115542664							Loss							Poly
LOC115542668				2.41									K15259	Poly
LOC115542670	-0.75													Poly
LOC115542683				0.44		1.83								
LOC115542690											Gain			Arginine vasopressin receptor 1A
LOC115542696				1.17										Poly
LOC115542702												Gain		
LOC115542705	-0.42		-0.50										K16866	Metalloproteinase inhibitor 3
LOC115542707	0.36	0.31	0.44	-0.51										ADP-ribosylation factor 4
LOC115542713										Gain	Gain			
LOC115542724						-0.49								Ankyrin
LOC115542725	-0.38		-0.55										K12484	Rab11 family-interacting protein 1/2/5
LOC115542727						-0.57							K08853	AP2-associated kinase
LOC115542730	-1.68					0.55								Eosinophil peroxidase
LOC115542732	-1.84		-1.49										K10788	Eosinophil peroxidase
LOC115542744	0.39	0.33				0.35							K20102	YTH domain-containing family protein
LOC115542751	-0.86		-0.64											Saposin
LOC115542755	-1.78	-0.68	-2.12										K07205	Eukaryotic translation initiation factor 4E binding protein 1
LOC115542757	-1.31		-0.63											
LOC115542759						-0.59							K23956	FERM and PDZ domain-containing protein 4
LOC115542766							Loss	Loss				Loss		

LOC115542771	0.42		0.35			-0.41							K11648	SWI/SNF-related matrix-associated actin-dependent regulator of chromatin subfamily B member 1
LOC115542789											Loss		K04429	Thousand and one amino acid protein kinase
LOC115542790						-0.39		Loss			Gain + Loss	Loss	K06840	Semaphorin 3
LOC115542798							Gain	Gain	Gain					Coagulation factor II (thrombin) receptor
LOC115542803	1.28	0.45	0.62											
LOC115542807		0.36												
LOC115542817	-0.39					-0.67							K04634	Guanine nucleotide-binding protein G(q) subunit alpha
LOC115542821						0.50								Heterogeneous nuclear ribonucleoprotein K
LOC115542825						-0.95								
LOC115542829						0.47								Serine/threonine-protein phosphatase 6 regulatory subunit 2
LOC115542847						-0.61							K11432	
LOC115542851	-0.80		-0.56	0.68		0.88							K21644	HMG box transcription factor 1
LOC115542860		-0.39	-0.54			-0.85								
LOC115542862	-0.67		-0.53			0.83								Ankyrin repeat and SOCS box protein 13
LOC115542863						0.70								
LOC115542875						-0.69						Loss		
LOC115542882						-0.57							K12384	Lysosome membrane protein 2
LOC115542884						-0.73							K09365, K09367	POU domain transcription factor, class 3, POU domain transcription factor, class 5
LOC115542885	0.42													Solute carrier family 20 (sodium-dependent phosphate transporter)
LOC115542886						-0.40							K05906	Prenylcysteine oxidase / farnesylcysteine lyase
LOC115542887	-0.60	-0.51	-0.70											
LOC115542892								Loss	Loss					
LOC115542911	-1.35		-0.80	-0.42		-1.13								
LOC115542915	-0.55													NLR family CARD domain-containing protein 3
LOC115542921	-0.51												K06098	Tight junction protein 2
LOC115542924						-2.05								
LOC115542935											Gain	Gain		Matrix metalloproteinase-17 (membrane-inserted)
LOC115542938												Loss		
LOC115542953						-0.60							K23338	Glucose-induced degradation protein 8
LOC115542960				0.60									K22614	NLR family CARD domain-containing protein 3
LOC115542962	-0.50		-0.70			-0.53					Loss	Loss	K09045	Activating transcription factor 7

LOC115542966				0.37		0.39								
LOC115542968						0.60								
LOC115542972				0.40										NLR family CARD domain-containing protein 3
LOC115542973						0.61							K22614	NLR family CARD domain-containing protein 3
LOC115542977	0.56		0.43											
LOC115542982						0.95							K11428	
LOC115542984	-1.73												K01697,<br>K01738	Cystathionine beta-synthase , cysteine synthase
LOC115542985				0.47										
LOC115542994	-0.45		-0.40			-0.62								
LOC115542995						-0.50							K00006	Glycerol-3-phosphate dehydrogenase (NAD+)
LOC115542997	0.49		0.61										K12836	Splicing factor U2AF 35 kda subunit
LOC115543006						0.77								
LOC115543015						-0.57							K17494	Cysteine/serine-rich nuclear protein
LOC115543016			-0.47											
LOC115543023	-0.82													
LOC115543025	-0.59		-0.49											TNF receptor-associated factor 2
LOC115543030						-0.67								
LOC115543034						0.67								
LOC115543039			0.39			0.30	Gain							Serine/arginine-rich splicing factor 9
LOC115543055									Gain			Gain		Rho gtpase-activating protein 20
LOC115543067						0.59								
LOC115543069						0.53								
LOC115543071				0.40										
LOC115543080					-0.71	-0.40								
LOC115543082						-0.59								
LOC115543084												Loss		
LOC115543087											Loss			
LOC115543088						0.74								
LOC115543094	-0.50													
LOC115543097						0.90								
LOC115543099							Loss							
LOC115543111				-0.28		-0.29								Serine/threonine-protein kinase 24/25/MST4

LOC115543115				-0.31									K15259	Poly
LOC115543123					0.80	1.23					Loss			
LOC115543125						0.79								Poly
LOC115543138				-0.59		-0.81					Loss	Loss		
LOC115543141	-0.61			-1.23		-2.16								Folylpolyglutamate synthase
LOC115543153						-0.58								
LOC115543157						0.62								
LOC115543396				-0.45		-1.31			Loss			Gain		
LOC115543398	-0.67	-0.35	-0.71											
LOC115543401						0.40								Protein Daple
LOC115543406											Loss			
LOC115543407						0.73							K08638	Carboxypeptidase X1
LOC115543411											Gain		K19941	Synapsin
LOC115543433												Gain		Integrin alpha 5
LOC115543445				0.54		0.50						Gain	K12484	Rab11 family-interacting protein 1/2/5
LOC115543446	-0.58			-0.53									K23503	Sideroflexin-5
LOC115543450						0.61								
LOC115543454			-0.61									Gain		
LOC115543475												Gain	K04908	Potassium voltage-gated channel Eag-related subfamily H member 5
LOC115543502				0.51		0.70					Gain			
LOC115543517						0.66							K20690	Rho guanine nucleotide exchange factor 25
LOC115543524	-0.53	-0.34	-0.65											F-box protein 30
LOC115543543	-0.57	-0.36												
LOC115543545	-0.58													
LOC115543555						-0.59							K23687	Ras and Rab interactor 2/3
LOC115543564	-1.62								Gain				K20363	Protein YIPF5/7
LOC115543566						-0.78								
LOC115543581		0.31	0.30											Histone deacetylase 1/2 , histone deacetylase 3
LOC115543593	-0.45		-0.44											
LOC115543597	-0.48		-0.58			-0.70								
LOC115543599						-0.72								Adaptor protein CIKS
LOC115543611			0.47			0.46								Actinin alpha 1/4

LOC115543625	-0.79		-0.43										K24456	Kyphoscoliosis peptidase
LOC115543628						0.55								
LOC115543632	0.58	0.36	0.51					Loss						
LOC115543644												Gain		
LOC115543646			-0.36											
LOC115543655	0.57												K14403	Cleavage and polyadenylation specificity factor subunit 3
LOC115543658						-0.68								Thrombospondin 1
LOC115543666		-0.47												Apolipoprotein B
LOC115543668						0.58								
LOC115543673	0.42	0.36	0.38										K08832	Serine/threonine-protein kinase SRPK1 , serine/threonine-protein kinase SRPK3
LOC115543677			0.77										K06637	Mitotic checkpoint serine/threonine-protein kinase BUB1 beta
LOC115543679												Loss		
LOC115543683						-0.73								
LOC115543685		0.31	0.50										K13218	Polypyrimidine tract-binding protein 1
LOC115543707	-1.61	-0.87	-1.72											Protein yippee-like 5
LOC115543720				0.46		0.63								
LOC115543723				-0.36										Kindlin 2
LOC115543724						-0.75							K09042	Transcription regulator BACH
LOC115543732						0.65								
LOC115543733								Loss			Gain	Gain		Muscarinic acetylcholine receptor M5
LOC115543735	-0.57													
LOC115543736						0.79						Loss		
LOC115543738						-0.77					Loss	Loss		
LOC115543739	-2.95	-0.85	-2.17											
LOC115543745							Gain					Gain	K07623	Gap junction beta-4 protein
LOC115543746				0.43		0.58								
LOC115543750						-0.38								
LOC115543753						-0.43								
LOC115543766						0.82								
LOC115543769						-0.35							K04536, K04537	Guanine nucleotide-binding protein G(I)/G(S)/G(T) subunit beta-1, guanine nucleotide-binding protein G(I)/G(S)/G(T) subunit beta-2
LOC115543774	-0.63					0.55								
LOC115543780						0.75								

LOC115543785			0.52											EH domain-containing protein 1, EH domain-containing protein 4
LOC115543788				0.42										Pericentrin
LOC115543790				-0.43									K03345, K08592	Sentrin-specific protease 1 , sentrin-specific protease 2 (axin associating molecule)
LOC115543791			0.44											P21-activated kinase 2
LOC115543798	-0.81	-0.41	-0.97											Human immunodeficiency virus type I enhancer-binding protein
LOC115543802			0.35				Gain	Gain	Gain					Solute carrier family 5 (sodium-coupled monocarboxylate transporter), member 8/12, solute carrier family 5 (sodium-dependent multivitamin transporter), member 6, solute:Na+ symporter, SSS family
LOC115543803	-0.76		-0.57											
LOC115543810			0.32											Dnaj homolog subfamily C member 5
LOC115543813								Loss						
LOC115543815	0.63		0.44										K18798	Peroxisome-assembly atpase
LOC115543818				0.42		0.81							K16463	Centrosomal protein CEP170
LOC115543819						0.46							K23859	Pleckstrin homology domain-containing family G member 1/2/3
LOC115543824	0.40												K03104	Signal recognition particle subunit SRP14
LOC115543825						0.65							K03104	Signal recognition particle subunit SRP14
LOC115543829	-0.64	-0.39	-0.68			-0.91								
LOC115543833	0.30			-0.36		-0.33								Serine/threonine-protein phosphatase PP1 catalytic subunit
LOC115543838	-0.46		-0.50			-0.71								Bcl-2-modifying factor
LOC115543842	-0.74													
LOC115543851	-0.49		-0.50			0.61					Loss			Interleukin 2 receptor gamma
LOC115543859	-2.09	-1.33	-2.04										K17603	Zinc finger FYVE domain-containing protein 1
LOC115543862			-0.38			-0.65							K04453	Max protein
LOC115543878								Loss				Gain + Loss		
LOC115543879							Loss	Loss	Loss		Loss	Loss	K04963	Ryanodine receptor 3
LOC115543882	-0.58		-0.40											Uncharacterized protein
LOC115543884		-0.44	-0.73			-1.09								
LOC115543893			0.66			-1.16								
LOC115543908			-0.84											
LOC115543919				-0.39		-0.49							K02564	Glucosamine-6-phosphate deaminase
LOC115543920												Loss		
LOC115543922	0.88	0.45	0.59											Ornithine decarboxylase

LOC115543939			-0.45											
LOC115543962	-0.65	-0.51	-0.69										K23487	Ena/VASP-like protein
LOC115543967						-0.57			Loss			Loss	K09275	Transcription factor CP2 and related proteins
LOC115543969				-0.42		-0.73								
LOC115543971						-0.70								Super conserved receptor expressed in brain 3
LOC115543992	0.43													Ataxin-3
LOC115543999						-0.50							K17338	Receptor expression-enhancing protein 1/2/3/4
LOC115544002	-0.66		-0.49										K00573	Protein-L-isoaspartate(D-aspartate) O-methyltransferase
LOC115544018								Loss	Loss		Gain			Neurexin
LOC115544021						-0.53								Fyn-related kinase , tyrosine-protein kinase Fyn
LOC115544036	0.60												K12160	Small ubiquitin-related modifier
LOC115544056	0.46												K12822	RNA-binding protein 25
LOC115544070			0.37											Presenilin 1
LOC115544075							Gain	Gain			Loss	Gain + Loss		
LOC115544082	-0.63		-0.39			-0.44								Retinol dehydrogenase 12
LOC115544091						-0.85								
LOC115544102						-0.98	Gain							
LOC115544119				0.56		0.84								Microtubule-associated protein 1
LOC115544120	0.46					0.37					Gain		K01411	Nardilysin
LOC115544121	0.73		0.77										K15183	RNA polymerase II elongation factor ELL
LOC115544122				0.48									K10456	Kelch-like protein 19
LOC115544123						-0.60							K09193	Transcription factor Sp3
LOC115544127						0.52								
LOC115544128											Loss	Loss		
LOC115544131	0.37													SH2 domain-containing adapter protein B/D/E/F
LOC115544133											Loss	Loss		
LOC115544134				-0.58		-0.82								Solute carrier family 6 (neurotransmitter transporter, GABA) member 1
LOC115544135	0.28					-0.57							K04634, K04635, K04636	Guanine nucleotide-binding protein G(q) subunit alpha, guanine nucleotide-binding protein subunit alpha-11, guanine nucleotide-binding protein subunit alpha-14
LOC115544138				-0.39		-0.78							K01080	Phosphatidate phosphatase
LOC115544143	1.28		0.47										K23616	Ataxin 1/1L
LOC115544144						0.45							K24724	TLE family member 5 (amino-terminal enhancer of split)

LOC115544155				-0.46		-0.94								Zinc finger and BTB domain-containing protein 18/42
LOC115544175								Loss						
LOC115544176	0.34		0.41			0.50								Glycoprotein endo-alpha-1,2-mannosidase
LOC115544187	-0.73					-0.48								
LOC115544200						-0.57							K24720	Tubby-related protein 4
LOC115544201						0.81								
LOC115544203											Gain			
LOC115544204							Gain					Loss		
LOC115544206	-0.71		-0.76			0.66								Cathepsin L
LOC115544211	0.52			-0.53		-0.48								Myristoylated alanine-rich C-kinase substrate
LOC115544219	-1.12		-0.62											
LOC115544225						0.42								Cytospin
LOC115544249						0.71								
LOC115544255												Loss		
LOC115544258	-0.81	-0.55	-0.84											
LOC115544261		-0.59												
LOC115544277				0.43		1.01							K05702	Afadin
LOC115544288				-0.42		-1.26							K25795	Insulinoma-associated protein
LOC115544292				-0.53		-0.66							K11162	Retinol dehydrogenase 14
LOC115544293						0.77								
LOC115544303				1.16		0.72								NLR family CARD domain-containing protein 3
LOC115544312				0.47									K08332	Vacuolar protein 8
LOC115544315						-0.67							K24518	Cysteine-rich motor neuron 1 protein
LOC115544319			0.54											Rho gtpase-activating protein 11
LOC115544321											Loss			Formin 1
LOC115544338							Gain	Gain	Gain		Gain		K05181	Gamma-aminobutyric acid receptor subunit beta
LOC115544347						-0.89								Odd-skipped
LOC115544350				-1.33		-1.16					Loss	Loss		Potassium channel subfamily K member 10
LOC115544352											Loss		K05190	Gamma-aminobutyric acid receptor subunit rho
LOC115544354						-0.60								ETS domain-containing protein Elk-4
LOC115544369						-0.51								
LOC115544375				0.44			Loss							
LOC115544376				1.65		0.81								

LOC115544383							Gain	Gain				Gain	K19467	Matrilin
LOC115544394	-0.61													
LOC115544414						0.49								
LOC115544419						-0.74								
LOC115544427						0.99								Paired box protein 1/9
LOC115544428						-0.61								
LOC115544440	-0.97		-2.28											Glutathione-specific gamma-glutamylcyclotransferase
LOC115544442	-0.71			0.65		1.39							K01424, K13278	60kda lysophospholipase , L-asparaginase
LOC115544443	0.37		0.38											ADP-ribosylation factor 6
LOC115544444							Loss	Loss	Loss					
LOC115544446						-1.20								
LOC115544457				0.48		0.83		Loss						MHC class I antigen
LOC115544461				-0.71		-1.50							K18757	La-related protein 1
LOC115544463						-0.39							K05094	Fibroblast growth factor receptor 3
LOC115544471											Loss	Loss	K19989	Tumor necrosis factor alpha-induced protein 2
LOC115544472						-0.42								MHC class I antigen
LOC115544474				1.15		1.04								MHC class I antigen
LOC115544476										Gain		Gain		MHC class I antigen
LOC115544478	0.78	0.40	0.68			0.34								Activator of Hsp90 atpase protein 1
LOC115544480	0.55	0.71	0.51			-0.81								Lysine-specific demethylase 9
LOC115544487	-1.21	-0.72	-0.77											Claudin
LOC115544501						0.67				Gain	Gain	Gain		
LOC115544522											Gain	Gain		
LOC115544528						0.58								
LOC115544531				0.41									K04622	G protein-coupled receptor family C group 6 member A
LOC115544537							Loss							5'-nucleotidase , 5'-nucleotidase / UDP-sugar diphosphatase
LOC115544541						0.61								
LOC115544548			0.65											DNA topoisomerase II
LOC115544555	0.54	0.39	0.62			-0.85								Histone-binding protein RBBP4
LOC115544556				-0.38		-0.53							K13198	KH domain-containing, RNA-binding, signal transduction-associated protein 1
LOC115544558		-0.48												
LOC115544561		0.42									Loss	Loss		

LOC115544643						0.44								
LOC115544649				-0.49										
LOC115544726						1.10							K01346	Pancreatic elastase II
LOC115544941						-0.73								
LOC115544944	-0.50	-0.58	-0.99			-0.47								Cytosolic arginine sensor for mtorc1 subunit 1
LOC115544945			-0.47											
LOC115544946	-0.67													
LOC115544948	-0.51					0.55								
LOC115544951						-0.42							K00901	Diacylglycerol kinase (ATP)
LOC115544955		0.38	0.39			-0.47								Methylcytosine dioxygenase TET2/3
LOC115544956				-0.55		-1.06								SET-binding protein
LOC115544972	0.26													Palmitoyltransferase ZDHHC14/18
LOC115544974	-0.80		-0.69										K24727	Myogenesis-regulating glycosidase
LOC115544976						0.42							K09070	Transcription factor 15
LOC115544979						-0.62							K24901	Homeobox protein orthopedia
LOC115544980						-0.83								Glial cell derived neurotrophic factor
LOC115544984	0.60													
LOC115545000						-0.42							K24058	PDZ domain-containing protein 2
LOC115545001											Loss			
LOC115545002	0.30												K02649	Phosphoinositide-3-kinase regulatory subunit alpha/beta/delta
LOC115545003										Gain				MFS transporter, SP family, solute carrier family 2 (facilitated glucose transporter), member 1
LOC115545012	0.47		0.74	-0.43		-0.85								
LOC115545018						-0.48								
LOC115545025			-0.54			-1.01							K07868	Rho-related BTB domain-containing protein 1/2
LOC115545031										Gain		Gain		
LOC115545032		0.23											K13210	Far upstream element-binding protein
LOC115545033		0.50	0.57											ER-derived vesicles protein
LOC115545037											Gain	Gain + Loss		
LOC115545038												Gain		
LOC115545044				-0.29		-0.48							K11285	TSPY-like 2
LOC115545055	-1.88	-1.18	-1.78									Loss		Serine/threonine-protein kinase ULK2

LOC115545056	1.08										Loss	Loss		Solute carrier family 38 (sodium-coupled neutral amino acid transporter), member 5
LOC115545062	-0.79												K08486	Syntaxin 1B/2/3
LOC115545066	0.49	0.31												Protein bicaudal D
LOC115545073						0.41							K05220	P2X purinoceptor 7
LOC115545081						-0.75							K00981	Phosphatidate cytidylyltransferase
LOC115545084	0.36					0.32							K11290	Template-activating factor I
LOC115545091											Loss	Gain		
LOC115545096	-0.44												K11647	SWI/SNF-related matrix-associated actin-dependent regulator of chromatin subfamily A member 2/4
LOC115545101							Gain							Tankyrase , uncharacterized protein
LOC115545114	-0.48	-0.35											K04501	Mothers against decapentaplegic homolog 4
LOC115545118	0.49		0.66	-0.43		-0.91							K17046	Protein DEK
LOC115545122		0.28												
LOC115545124								Loss						
LOC115545128	-0.86	-0.50	-1.68										K03096	Frequently rearranged in advanced T-cell lymphomas 2
LOC115545131						-0.69								
LOC115545134							Gain				Gain	Gain		RILP-like protein 1
LOC115545135			0.45											
LOC115545137							Loss	Gain			Gain	Gain		
LOC115545142	0.35		0.56											
LOC115545145				0.48										Citron Rho-interacting kinase
LOC115545159											Gain	Gain	K24889	Iroquois-class homeodomain protein IRX
LOC115545164			0.46											Myosin I
LOC115545167						-0.80								F-box and leucine-rich repeat protein 10/11
LOC115545181	0.50		0.45											Acidic leucine-rich nuclear phosphoprotein 32 family member E
LOC115545184						0.60								Betaine-homocysteine S-methyltransferase
LOC115545185						0.45								Betaine-homocysteine S-methyltransferase
LOC115545190						-0.62								
LOC115545191				-0.40		-0.58								
LOC115545192	0.29													2-oxoglutarate dehydrogenase E1 component
LOC115545194						-1.00								Deltex
LOC115545196											Gain		K21432	NADPH oxidase activator 1
LOC115545197						-0.59								Neurofascin

LOC115545199				0.51										
LOC115545200						-0.63								
LOC115545201			-0.62											
LOC115545239						0.67								Solute carrier family 6 (neurotransmitter transporter, GABA) member 13
LOC115545240						0.76								Saposin
LOC115545249			0.49											Long-chain acyl-coa synthetase
LOC115545252						-0.88							K02165	Dickkopf 1/2/4
LOC115545257						0.58								Eukaryotic translation initiation factor 2C
LOC115545258								Loss	Loss			Gain		Eukaryotic translation initiation factor 2C
LOC115545265	-0.61	-0.55	-0.61											
LOC115545272	-0.46													
LOC115545278				0.37										
LOC115545281						0.61								Hyaluronoglucosaminidase
LOC115545283				-0.44		-1.18								Serine/threonine-protein phosphatase 6 catalytic subunit
LOC115545287						-0.60						Loss		
LOC115545299	0.40													Acylaminoacyl-peptidase
LOC115545302			-0.43											
LOC115545306	0.93					-0.65								
LOC115545317			-0.29						Loss		Gain			
LOC115545323				0.90										
LOC115545327				2.93										
LOC115545331				0.41		0.84								Focal adhesion kinase 1
LOC115545336	0.71		0.38										K24169	Thyroid adenoma-associated protein
LOC115545339					1.09									
LOC115545345	-0.68		-0.36											Upstream stimulatory factor
LOC115545377						0.43							K06691, K20456, K20462	26S proteasome regulatory subunit N13, oxysterol-binding protein 1, oxysterol-binding protein 2
LOC115545380						-0.49							K00904	Deoxyguanosine kinase
LOC115545382	0.63													
LOC115545383	-0.88													
LOC115545385			0.45										K05768	Gelsolin
LOC115545389	0.53					-0.64							K13112	Thyroid hormone receptor-associated protein 3

LOC115545394			0.42											
LOC115545396	-0.69		-0.59											
LOC115545397			0.35											Glutamine---fructose-6-phosphate transaminase (isomerizing)
LOC115545400	-0.39	-0.41	-0.65										K07206	Tuberous sclerosis 1
LOC115545407						0.70							K08732	Ral guanine nucleotide dissociation stimulator
LOC115545415							Loss						K12186	ESCRT-I complex subunit MVB12
LOC115545417						-0.85								
LOC115545452						-0.44								
LOC115545453				-0.40		-0.62								
LOC115545461	-0.55													Aspartyl-trna(Asn)/glutamyl-trna(Gln) amidotransferase subunit C
LOC115545474						0.41								
LOC115545475	0.47													Clathrin heavy chain
LOC115545480												Gain	K06238	Collagen type VI alpha
LOC115545487						0.62								Solute carrier family 4 (sodium bicarbonate cotransporter), member 7
LOC115545489				-0.79		-1.12								
LOC115545496						0.69								
LOC115545501								Loss			Gain	Gain		
LOC115545502											Loss	Gain		
LOC115545508						-0.69								General transcription factor IIIA
LOC115545509						0.67						Gain		
LOC115545516						-0.75								Potassium channel subfamily K member 5
LOC115545517							Loss						K12354	Ectonucleotide pyrophosphatase/phosphodiesterase family member 7
LOC115545520			-0.54											CCAAT/enhancer binding protein (C/EBP), beta
LOC115545521						0.70								
LOC115545522						0.40							K03097	Casein kinase II subunit alpha
LOC115545523	-0.52	-0.40		-0.40										
LOC115545530						0.72								
LOC115545532	0.83													Ribonucleases P/MRP protein subunit RPP25
LOC115545538	-0.53													
LOC115545539									Gain		Loss	Loss	K04511	Prickle
LOC115545549						0.75								
LOC115545559								Loss				Loss		

LOC115545564						0.92	Loss					Gain + Loss		
LOC115545571	-0.72		-0.58											Gamma-glutamyl hydrolase
LOC115545576						-0.45								
LOC115545586						-1.06								
LOC115545590	-1.05	-0.75	-1.10											Iporin
LOC115545594						-0.77							K15686	RNA-binding protein MEX3
LOC115545600	0.58		0.47											
LOC115545605		-0.52	-0.38											Breakpoint cluster region protein
LOC115545613						-0.72							K17700	RAP1 gtpase activating protein 1
LOC115545614	0.56		0.67										K07375	Tubulin beta
LOC115545615			-0.54											
LOC115545616				-0.44		-0.82							K09048	Cyclic AMP-responsive element-binding protein 3
LOC115545620						-0.30								
LOC115545623	0.71		0.54								Loss			
LOC115545632				-0.42										
LOC115545633			-0.48											
LOC115545636	-1.24	-0.80	-1.19											
LOC115545646	-0.37													Merlin
LOC115545678	0.44		0.46										K06641	Serine/threonine-protein kinase CHEK2
LOC115545679						0.38								COMPASS component SWD3
LOC115545681	-0.60												K22904	Presqualene diphosphate phosphatase
LOC115545682						0.62								Glutathione S-transferase
LOC115545684				-0.44										Phosphatidylinositol 4-phosphatase
LOC115545690						-0.59								Glioma pathogenesis-related protein 2
LOC115545691						-0.57								Glioma pathogenesis-related protein 2
LOC115545698						-1.06							K19901	Disabled homolog 2-interacting protein
LOC115545703	0.29													Atpase family AAA domain-containing protein 1
LOC115545704											Loss			
LOC115545714						0.88								
LOC115545721						0.87					Loss	Loss		
LOC115545722				-0.41		-0.89								Growth arrest-specific 1
LOC115545727	-0.65		-0.69	-0.41		-1.07						Gain + Loss	K05058	Leukemia inhibitory factor receptor

LOC115545738									Gain			Gain		Derlin-2/3
LOC115545744	0.56		0.57			-0.50							K03163	DNA topoisomerase I
LOC115545747		0.28	0.52											
LOC115545753						0.40								
LOC115545757						-0.69							K16056	Calcium release-activated calcium channel protein 1
LOC115545767				-0.49		-1.08								
LOC115545774										Loss	Loss		K22614	NLR family CARD domain-containing protein 3
LOC115545782			-0.40			0.51								E3 ubiquitin-protein ligase RNF146
LOC115545792						-0.49							K00572	Wingless-type MMTV integration site family, member 7
LOC115545793											Gain	Gain		
LOC115545799												Gain		Voltage-dependent calcium channel N type alpha-1B
LOC115545805	-0.52										Gain			Cardiomyopathy-associated protein 5
LOC115545806	-0.35					-0.48							K24630	Protein kinase C-binding protein 1
LOC115545818												Loss	K10505	Zinc finger and BTB domain-containing protein 26
LOC115545820	0.59		0.58											Survival motor neuron protein
LOC115545825	0.50		0.51											AP2-associated kinase
LOC115545832						0.58						Loss		
LOC115545833						0.68								
LOC115545854						0.64								Early B-cell factor
LOC115545875							Loss		Loss				K05208	Glutamate receptor ionotropic, NMDA 1
LOC115545885						0.58							K01530	Phospholipid-translocating atpase
LOC115545887			-0.42											
LOC115545893	-0.73													
LOC115545896						-1.13							K09092	Transcription factor-like 5
LOC115545904												Loss		Potassium channel subfamily K member 15
LOC115545906								Loss				Loss	K07851	Ras-like protein family member 10B
LOC115545919						0.54								8-oxo-dgtp diphosphatase
LOC115545927	0.45													
LOC115545931						1.20								
LOC115545941							Gain + Loss	Gain	Gain + Loss			Gain		
LOC115545948	-2.29	-1.14	-3.21										K03841	Fructose-1,6-bisphosphatase I
LOC115545951	-0.60													Histone H2B

LOC115545954								Gain						Netrin-G2
LOC115545961					1.18									Collagen type I alpha, collagen type II alpha
LOC115545971											Gain + Loss	Loss	K06732	CUB domain-containing protein 1
LOC115545976											Gain			
LOC115545979				0.46		0.69								C-type lectin domain family 3 member B
LOC115545996						-0.75								Gamma-glutamyltranspeptidase / glutathione hydrolase / leukotriene-C4 hydrolase
LOC115546002											Gain	Gain		
LOC115546004							Loss		Gain					
LOC115546012	-0.87	-0.62	-0.80	0.35										
LOC115546014						0.75						Loss		
LOC115546015						0.92								
LOC115546016						-0.55							K12487	G protein-coupled receptor kinase interactor 2
LOC115546027			0.64										K23819	Sushi domain-containing protein 1
LOC115546032	-0.54												K24045	Junction-mediating and -regulatory protein
LOC115546038	-1.00		-0.67			-0.59								Lysophospholipid hydrolase , NTE family protein
LOC115546040						-0.41							K13575	Solute carrier family 4 (sodium bicarbonate cotransporter), member 4
LOC115546042		0.39												
LOC115546046												Gain	K23844	NMDA receptor synaptonuclear signaling and neuronal migration factor
LOC115546047	0.44													Calcium/calmodulin-dependent protein kinase (cam kinase) II
LOC115546065						-0.52								Transcriptional activator protein Pur-alpha, transcriptional activator protein Pur-beta
LOC115546069	0.37													Histone H2A
LOC115546071	-0.50		-0.61											
LOC115546073											Gain		K21879	Whirlin
LOC115546079				0.64		0.76								
LOC115546084												Gain		
LOC115546085						2.24								Histone H3
LOC115546107	-0.85		-0.56											
LOC115546124	-0.86	-0.53	-0.94											
LOC115546133				-0.36									K22074	NFU1 iron-sulfur cluster scaffold homolog, mitochondrial
LOC115546138			0.51											Aquaporin-3

LOC115546140									Loss		Gain	Gain	K24822	Lipoxygenase homology domain-containing protein 1
LOC115546147			-0.75											Glutaminase
LOC115546162								Gain	Gain			Gain + Loss		Zymogen granule membrane protein 16
LOC115546163	-0.54												K25747	Zymogen granule membrane protein 16
LOC115546172				0.40									K19029	6-phosphofructo-2-kinase / fructose-2,6-biphosphatase 2
LOC115546173						-0.44							K25568	Kazal-type serine protease inhibitor domain-containing protein 1
LOC115546174							Loss				Gain			
LOC115546178						-0.60								Cell division cycle 14
LOC115546185				-0.53										
LOC115546212				0.45									K00036	Glucose-6-phosphate 1-dehydrogenase
LOC115546328						0.66								Poly(rc)-binding protein 2, poly(rc)-binding protein 3/4
LOC115546386				0.60										
LOC115546442						0.81								
LOC115546458						-0.98								Discs, large-associated protein 1
LOC115546460	-0.50													
LOC115546462						-0.59								Homeobox protein MSX
LOC115546468	0.57	0.57	0.72										K13195	Cold-inducible RNA-binding protein
LOC115546469			0.47										K11251	Histone H2A
LOC115546471												Loss		
LOC115546481											Loss			
LOC115546488				0.44		0.63								NLR family CARD domain-containing protein 3
LOC115546491		0.31				0.33								Protein transport protein SEC61 subunit alpha
LOC115546504						0.53							K26006	Cobalamin trafficking protein cbld
LOC115546505							Loss		Loss					3-hydroxybutyrate dehydrogenase
LOC115546510			0.40											
LOC115546514											Gain			Transcription factor 15
LOC115546516							Gain		Gain			Gain		
LOC115546517						-0.48					Loss			Phospholamban
LOC115546518						0.51								
LOC115546523	-0.59		-0.51						Loss		Gain	Gain + Loss		
LOC115546524	-0.63													
LOC115546525	-0.56		-0.67										K09115	MAX-binding protein

LOC115546526						-0.58								
LOC115546529	-1.21													
LOC115546533				0.45										Rho gtpase-activating protein 42
LOC115546538						2.59								
LOC115546543											Gain	Gain	K09191	General transcription factor IIIA
LOC115546544				0.31										
LOC115546550								Gain			Loss			
LOC115546553	-0.37													Transcriptional coactivator YAP1
LOC115546559						0.49								General transcription factor IIIA, KRAB domain-containing zinc finger protein
LOC115546563						0.55								Vacuole membrane protein 1
LOC115546577						1.33								NLR family CARD domain-containing protein 3
LOC115546589												Loss		
LOC115546623	-0.89	-0.47	-0.98											Solute carrier family 48 (heme transporter), member 1
LOC115546641						0.53								NLR family CARD domain-containing protein 3
LOC115546644			-0.33											
LOC115546652						0.58								NLR family CARD domain-containing protein 3
LOC115546655						-0.85							K13864	Solute carrier family 7 (cationic amino acid transporter), member 2
LOC115546666			-2.11										K15103	Solute carrier family 25 (mitochondrial uncoupling protein), member 8/9
LOC115546671												Loss		
LOC115546685	-0.55												K00933	Creatine kinase
LOC115546694						0.45								Tetraspanin-2
LOC115546700			-0.64										K17708	RAP1 gtpase activating protein 2
LOC115546706	0.45			-0.37										Mitochondrial calcium uniporter regulator 1
LOC115546708							Loss						K07863, K07975	Ras homolog gene family, member G, Rho family, other
LOC115546711			-0.45										K11833	Ubiquitin carboxyl-terminal hydrolase 2
LOC115546712	-2.02					0.51								
LOC115546716				-0.52		-0.94								Cytoskeleton-associated protein 2
LOC115546720			0.44	0.40										Plastin-3
LOC115546725						0.53							K22614	NLR family CARD domain-containing protein 3
LOC115546727	-0.73													
LOC115546738	-1.91	-1.09	-1.68			-1.20							K11795	DDB1- and CUL4-associated factor 6

LOC115546740							Loss						K07377	Neurexin
LOC115546743				0.43									K00688	Glycogen phosphorylase
LOC115546751			-0.30											Transcriptional activator protein Pur-alpha
LOC115546781				2.00									K22614	NLR family CARD domain-containing protein 3
LOC115546788				-0.51		-1.15								E3 ubiquitin-protein ligase CBL
LOC115546793												Gain		Myelin protein zero
LOC115546795							Gain	Loss	Loss				K16795	Platelet-activating factor acetylhydrolase IB subunit beta/gamma
LOC115546797	-0.44													
LOC115546803				-0.34									K17901	Calcipressin-1
LOC115546807												Loss		
LOC115546817												Loss		
LOC115546821									Loss			Gain		
LOC115546826						0.58								NLR family CARD domain-containing protein 3
LOC115546827						0.78							K16459	Centrosomal protein CEP120
LOC115546834				-0.53		-1.12								C-crk adapter molecule crk
LOC115546839				0.53		0.51								
LOC115546840						-0.60							K08788	Dystrophia myotonica-protein kinase
LOC115546843				-0.33										
LOC115546852						0.82								
LOC115546853								Loss						
LOC115546857						-0.76							K22415	Zinc finger CCCH domain-containing protein 11
LOC115546865						-0.78								
LOC115546875	0.37													
LOC115546885						-0.73								Endothelial cell adhesion molecule
LOC115546891							Loss	Loss	Loss					Diacylglycerol O-acyltransferase 2
LOC115546896	0.52													Coiled-coil alpha-helical rod protein 1
LOC115546914						-0.68								Four and a half LIM domains protein 1
LOC115546915						0.58							K04429	Thousand and one amino acid protein kinase
LOC115546917	0.46			-0.42									K09489	Heat shock 70kda protein 4
LOC115546922	0.63		0.41			0.47							K01277	Dipeptidyl-peptidase III
LOC115546923						-0.57								Actin filament associated protein 1-like
LOC115546927						-0.44							K20044	Phosphatidylinositol-binding clathrin assembly protein
LOC115546930	0.32		0.44										K13189	RNA-binding protein 14

LOC115546934						0.99								
LOC115546942						-0.55							K04840	Voltage-gated sodium channel type VIII alpha
LOC115546949												Loss		Calpain-5
LOC115546955		-0.41											K09366	POU domain transcription factor, class 4
LOC115546960						0.45								
LOC115546969						-0.74								
LOC115547007	-0.37													Mitochondrial fission factor
LOC115547011						0.45							K20314	Arfaptin
LOC115547013	-0.56												K25543	Beta-galactosidase-1-like protein
LOC115547019				-0.41		-0.76							K25605	B-cell CLL/lymphoma 7 protein
LOC115547023												Loss	K06782	Cell adhesion molecule 2
LOC115547024	0.47	0.46	0.69									Gain		
LOC115547027	0.63													Ras association domain-containing protein 7/8
LOC115547029	0.28	0.28	0.40										K13044	Heterogeneous nuclear ribonucleoprotein A/B/D
LOC115547035	0.38	0.36	0.38											Serine/arginine-rich splicing factor 1
LOC115547038			-0.38											Baculoviral IAP repeat-containing protein 2/3, baculoviral IAP repeat-containing protein 7/8
LOC115547047						0.53								
LOC115547054	-0.51		-0.56			0.67								Transient receptor potential cation channel subfamily V member 1
LOC115547059						-0.36								Flotillin
LOC115547062	-0.79		-0.69										K22449	Tubulin-specific chaperone cofactor E-like protein
LOC115547069											Loss	Loss		X-linked retinitis pigmentosa gtpase regulator
LOC115547073				-0.69		-0.96							K20804	E3 ubiquitin-protein ligase RNF34
LOC115547082						-0.62							K00444	Wingless-type MMTV integration site family, member 5
LOC115547087									Gain			Loss	K16506	Protocadherin Fat 1/2/3
LOC115547096	0.39												K13211	GC-rich sequence DNA-binding factor
LOC115547112												Loss		
LOC115547113	-0.49											Loss	K14352	Solute carrier organic anion transporter family, member 2B
LOC115547114												Gain		
LOC115547120		-0.94	-0.76											Carbonic anhydrase , carbonic anhydrase 4
LOC115547123											Gain	Gain + Loss	K25739	Scavenger receptor cysteine-rich domain-containing group B protein
LOC115547129	-0.72		-0.46											

LOC115547133						-0.52								Tripartite motif-containing protein 35
LOC115547139										Loss	Loss	Loss	K04257	Olfactory receptor
LOC115547155	-0.47													
LOC115547156						0.72								Myosin XVIII
LOC115547157	0.49												K11448	Lysine-specific demethylase 6B
LOC115547159						0.81								
LOC115547161												Loss	K24511	Von Willebrand factor A domain-containing protein 7
LOC115547169						0.37	Loss						K06685	MOB kinase activator 1
LOC115547175						-0.51								
LOC115547182							Gain		Loss		Gain			
LOC115547185									Loss			Loss		Protocadherin delta 2
LOC115547187			0.41											APC membrane recruitment protein 1
LOC115547188						0.95								Neuronal PAS domain-containing protein 2
LOC115547196											Loss	Loss		
LOC115547206						0.58							K12045	Troponin T, cardiac muscle
LOC115547213								Loss						Protein phosphatase 1D
LOC115547215							Gain							
LOC115547217				-0.31										Adenylosuccinate synthase
LOC115547222				-0.44		-0.61								
LOC115547223						0.42								Histamine receptor H2
LOC115547224		0.47											K08552	Estrogen-related receptor alpha
LOC115547226									Loss					
LOC115547228				-0.39										Gap junction alpha-3 protein
LOC115547235						-0.60								
LOC115547236			-0.37											
LOC115547237	0.46					0.77							K11276	Nucleophosmin 1
LOC115547255				0.57		1.03								
LOC115547260			-0.80										K15100	Solute carrier family 25 (mitochondrial citrate transporter), member 1
LOC115547261						0.59							K26100	PWWP domain-containing protein 2
LOC115547262						0.43								Tetratricopeptide repeat protein 1
LOC115547263						-0.34							K06491	Neural cell adhesion molecule
LOC115547266						0.50						Gain		Cell adhesion molecule 1

LOC115547268				-3.36									K14648	Poly(U)-specific endoribonuclease
LOC115547292	-0.70	-0.30	-0.48										K04413	Misshapen/NIK-related kinase
LOC115547297	-1.25	-0.68	-1.33											17beta-estradiol 17-dehydrogenase / very-long-chain 3-oxoacyl-coa reductase
LOC115547298						0.64								Protocadherin-16/23
LOC115547299						0.67								
LOC115547306						-0.58								Amphiphysin
LOC115547310						0.68							K05732	Glucocorticoid receptor DNA-binding factor 1
LOC115547312	-0.87		-0.59			0.54							K16185	Ras-related GTP-binding protein A/B
LOC115547314							Gain							
LOC115547316												Loss		
LOC115547322							Gain				Loss			
LOC115547323	-0.77	-0.52	-1.05			-0.50								Tripartite motif-containing protein 2/3
LOC115547324		-0.46	-0.76											
LOC115547340						-0.52								
LOC115547347			-0.53											MFS transporter, OCT family, solute carrier family 22 (organic cation transporter), member 4/5
LOC115547348	-0.59		-1.06											
LOC115547351	0.36											Loss		Chloride channel 3/4/5
LOC115547353							Loss						K04265	Adenosine receptor A1
LOC115547358	-0.45					0.42							K10356	Myosin I
LOC115547369						0.55						Gain	K09191, K09228	General transcription factor IIIA, KRAB domain-containing zinc finger protein
LOC115547372	0.36												K09191, K09228	General transcription factor IIIA, KRAB domain-containing zinc finger protein
LOC115547375						0.98								
LOC115547378						-0.40								
LOC115547380	-0.64		-0.51										K20125	F-BAR and double SH3 domains protein
LOC115547384				-0.37		-0.46							K15190	7SK snrna methylphosphate capping enzyme
LOC115547385	0.42		0.48	0.31		0.47								Transcription elongation regulator 1
LOC115547395						-0.35							K08583	Calpain, small subunit 1
LOC115547400						0.57								Potassium voltage-gated channel Shaker-related subfamily A member 1, potassium voltage-gated channel Shaker-related subfamily A member 3, potassium voltage-gated channel Shaker-related subfamily A member 6, potassium voltage-gated channel Shaker-related subfamily A member 7

LOC115547403												Loss	K09444	Interferon regulatory factor 1
LOC115547409	-0.59		-0.55						Gain		Gain		K21122	Ubiquitin carboxyl-terminal hydrolase 28
LOC115547423						0.46							K23483	Gamma-crystallin
LOC115547428											Loss			
LOC115547429												Loss		A disintegrin and metalloproteinase with thrombospondin motifs 15
LOC115547431	-0.56		-0.37				Gain					Loss	K15706	E3 ubiquitin-protein ligase RNF167
LOC115547435							Gain							
LOC115547447			-0.26											
LOC115547450										Gain	Gain		K04995	Potassium inwardly-rectifying channel subfamily J member 1
LOC115547451	-0.76		-0.53											
LOC115547459	-0.73		-0.57											
LOC115547460											Gain			
LOC115547464	-0.44		-0.63											
LOC115547468						0.54								
LOC115547470						0.38							K05863	Solute carrier family 25 (mitochondrial adenine nucleotide translocator), member 4/5/6/31
LOC115547485	-0.70	-0.39	-0.93										K09191, K09228	General transcription factor IIIA, KRAB domain-containing zinc finger protein
LOC115547486											Loss			
LOC115547490						-0.95								Eph receptor A2
LOC115547493						0.39								Cell division cycle 2-like
LOC115547496	-0.55		-0.59										K14445	ABC-2 type transport system permease protein, solute carrier family 13 (sodium-dependent dicarboxylate transporter), member 2/3/5
LOC115547498							Gain + Loss						K04136	Adrenergic receptor alpha-1B
LOC115547499			-0.42										K07361	Lymphocyte cytosolic protein 2
LOC115547504	0.38													
LOC115547509						0.71								Pigment epithelium-derived factor
LOC115547513	-0.73			-0.76		-1.08							K07820	Beta-1,3-galactosyltransferase 2
LOC115547515	-0.49			-3.84		-3.85								Beta-1,3-galactosyltransferase 2
LOC115547518	-0.38		-0.40											E3 ubiquitin-protein ligase SIAH1 , E3 ubiquitin-protein ligase SIAH2
LOC115547525						0.63								
LOC115547526						0.56					Gain	Gain	K23961	Band 4.1-like protein 1/2/3

LOC115547528						-0.67								LHFPL tetraspan subfamily member protein
LOC115547539				-0.49		-0.78								
LOC115547546	-0.58													
LOC115547551							Gain	Gain			Loss	Loss		Transient receptor potential cation channel subfamily C member 2
LOC115547555						-0.65								
LOC115547559							Gain							NLR family CARD domain-containing protein 3
LOC115547568						0.59								
LOC115547580	-1.04												K24031	Lamina-associated polypeptide 2
LOC115547591						0.69							K01124	Glycerophosphoinositol inositolphosphodiesterase
LOC115547593							Loss		Loss				K06517	CD96 antigen
LOC115547597			-0.43											
LOC115547598				-0.58		-1.45							K09203	Early growth response protein 1
LOC115547603	0.76	0.39	0.50			-0.37								
LOC115547610						-0.75							K17280	Chordin-like protein 2
LOC115547618				-0.51		-0.81							K16197, K16198	14-3-3 protein beta/theta/zeta, 14-3-3 protein gamma/eta
LOC115547631						-0.46								
LOC115547634						0.60							K19895	GDNF family receptor alpha
LOC115547640	0.42													Ribosomal protein S6 kinase beta
LOC115547650						0.44								Protein jagunal
LOC115547651						0.58						Gain		
LOC115547653						0.42								Rab proteins geranylgeranyltransferase component A
LOC115547654	-0.85		-0.50											
LOC115547657											Gain	Gain		
LOC115547658			-0.36	-0.42		-0.78							K23794	Pleckstrin homology-like domain family B
LOC115547659	-0.77		-0.52										K24505	Glucocorticoid modulatory element-binding protein
LOC115547662			0.35											Spectrin beta
LOC115547663	-0.40												K11292	Transcription elongation factor SPT6
LOC115547667				0.46										Protein phosphatase slingshot
LOC115547668						0.75								Pleckstrin homology-like domain family B
LOC115547669											Gain			
LOC115547671	-0.37		-0.39			0.48								U2-associated protein SR140
LOC115547674						-0.48								

LOC115547675						-1.17								
LOC115547677						-0.46		Gain						
LOC115547683				-0.49		-0.73							K00778	Beta-galactoside alpha-2,6-sialyltransferase (sialyltransferase 1)
LOC115547688												Loss	K19404	Phosphatidic acid-selective phospholipase A1
LOC115547694						-0.62								Palmitoyltransferase ZDHHC2/15/20
LOC115547695										Gain				
LOC115547705	-0.52		-0.59											Ras-related protein Rab-34
LOC115547713				-0.32									K17682	Mitochondrial nucleoid factor 1
LOC115547721				-0.41		-0.68								Diamine N-acetyltransferase
LOC115547725	0.83													
LOC115547733	0.76	0.39	0.66											Heterogeneous nuclear ribonucleoprotein F/H
LOC115547736				-0.42		-0.58								Gap junction alpha-1 protein
LOC115547738												Loss		Nuclear apoptosis-inducing factor 1
LOC115547740	0.65		0.39										K10704	Ubiquitin-conjugating enzyme E2 variant
LOC115547744						-0.61							K10315	F-box protein 40
LOC115547746												Loss		
LOC115547762				-0.20										
LOC115547765				-0.48		-0.46		Gain						
LOC115547771				-0.54		-0.67								
LOC115547772	0.81	0.53	0.58	-0.48		-0.48								Protein Tob/BTG
LOC115547777						0.61					Loss		K06767	Down syndrome cell adhesion molecule
LOC115547778							Loss	Loss			Loss			Metabotropic glutamate receptor 5
LOC115547780						0.72								Protein Shroom
LOC115547782			0.22										K16939	Septin 6/8/11
LOC115547786						0.82								
LOC115547794											Gain	Gain	K19917	Double C2-like domain-containing protein beta
LOC115547799						-0.62								
LOC115547811									Gain				K05200	Glutamate receptor 4
LOC115547822	-0.54												K07994	Matrix metalloproteinase-13 (collagenase 3)
LOC115547827	0.39		0.52									Loss	K09501	Serpin peptidase inhibitor, clade H, member 1
LOC115547833	0.55													Immunoglobulin superfamily member 9B
LOC115547836						-0.82							K15287	Solute carrier family 35, member F1/2
LOC115547837	0.48		0.52											Histone deacetylase complex subunit SAP18

LOC115547845						-0.66								SR-related and CTD-associated factor 4
LOC115547855						-0.42								O-palmitoleoyl transferase
LOC115547864	0.62		0.53											Deoxycitidine/deoxyadenosine/deoxyguanosine kinase
LOC115547873							Loss	Loss	Loss				K18625	Protein Shroom
LOC115547887							Gain				Loss	Loss		
LOC115547900						3.02								Beta-crystallin
LOC115547902						-0.63						Gain		Nicotinic acetylcholine receptor epsilon
LOC115547907	0.45													Solute carrier family 7 (cationic amino acid transporter), member 3
LOC115547910												Loss		
LOC115547911						0.69								
LOC115547917	-0.53		-0.46			0.68								
LOC115547923									Loss					
LOC115547932						-0.81							K06051	Delta
LOC115547939	0.81					-0.78								
LOC115547951			0.60	0.65									K14242	NF-kappa-B inhibitor zeta
LOC115547969	-1.23	-1.32	-1.10			-0.45								MFS transporter, PCFT/HCP family, solute carrier family 46, member 3
LOC115547975	-0.48		-0.40			-0.61								
LOC115547976				0.41									K05090	Macrophage colony-stimulating factor 1 receptor
LOC115547982						0.61								
LOC115547983	-0.58													
LOC115547988	-0.63		-0.59										K07532	Rho guanine nucleotide exchange factor 12
LOC115547990						0.52							K01403	Matrix metalloproteinase-9 (gelatinase B)
LOC115547992	-0.53	-0.49	-0.77			-0.60								Dapper
LOC115547996	-2.21	-1.20	-2.45			-0.60							K16815	Calcium-independent phospholipase A2-gamma
LOC115548007											Loss	Loss		
LOC115548029	-0.79	-0.39	-0.66											
LOC115548080						-0.67								
LOC115548119						1.10								Deoxynucleoside triphosphate triphosphohydrolase SAMHD1
LOC115548167						-0.90								
LOC115548178						-0.51							K09188	
LOC115548182			0.46				Loss							Tetratricopeptide repeat protein 39
LOC115548183				0.61		0.91								5'-AMP-activated protein kinase, regulatory gamma subunit

LOC115548194						0.63	Loss		Loss					
LOC115548195						0.50								
LOC115548199							Loss						K14712	Solute carrier family 39 (zinc transporter), member 6
LOC115548200	-0.87	-0.87	-1.15											Chitinase
LOC115548203			-1.29											
LOC115548205				0.43		0.46								
LOC115548210				0.44										
LOC115548211						0.73								
LOC115548212							Loss	Loss	Loss		Gain			
LOC115548221						-0.62								
LOC115548222		0.94	0.73										K05137	Interleukin 20 receptor beta
LOC115548240	-0.40			-0.44		-0.76								Importin-13
LOC115548241						0.41							K13293	Camp-specific phosphodiesterase 4
LOC115548245				-0.37		-0.44								Sodium/potassium-transporting atpase subunit alpha
LOC115548254						0.66								
LOC115548258						-0.46								Arf-GAP with gtpase, ANK repeat and PH domain-containing protein 1/3/4/5/6/9/11
LOC115548265	-1.04	-0.62	-1.37			-0.44								Autophagy-related protein 9
LOC115548278			-0.52			0.46							K12899	Serine/arginine-rich splicing factor 11
LOC115548296	-1.43	-0.52	-2.19											
LOC115548298			-0.46											E3 ubiquitin-protein ligase RNF11
LOC115548301						0.67								
LOC115548306						0.43	Loss	Loss	Gain		Gain + Loss	Gain + Loss		
LOC115548307											Loss	Loss		
LOC115548308												Gain		
LOC115548310										Gain				
LOC115548311	-0.68					-0.66							K01068	Acyl-coenzyme A thioesterase 1/2/4
LOC115548329											Loss			
LOC115548335						0.75								Ankyrin repeat domain-containing protein 11/12
LOC115548336			0.38										K01895	Acetyl-coa synthetase
LOC115548341	1.60	1.33	1.39											Nuclear autoantigenic sperm protein
LOC115548351			0.46	0.45									K12006	Tripartite motif-containing protein 16
LOC115548352				-0.39		-0.63							K00814	Alanine transaminase

LOC115548354	-0.63		-0.36											
LOC115548357							Loss	Loss						Rhomboid-like protein
LOC115548358							Gain							KISS1 receptor
LOC115548364			0.57											
LOC115548366	-0.63													C1q-related factor
LOC115548369						0.43								
LOC115548377	0.47												K12472	Epidermal growth factor receptor substrate 15
LOC115548379	0.55	0.52	0.55			0.51							K08057	Calreticulin
LOC115548386			0.48										K10402	Kinesin family member 20
LOC115548395	0.64			-0.57		-0.60							K03260	Translation initiation factor 4G
LOC115548406						0.84							K00907	Myosin-light-chain kinase
LOC115548407	-0.48	-0.46	-0.85										K06104	Angiomotin like
LOC115548410	0.40													
LOC115548411										Loss	Loss	Loss	K13748	Alpha-1,3-mannosylglycoprotein beta-1,4-N-acetylglucosaminyltransferase C
LOC115548414			-0.47											
LOC115548418						-0.70							K09396	Forkhead box protein C
LOC115548432												Loss		
LOC115548433						-0.55							K08413	G protein-coupled receptor 55
LOC115548436			-0.79											
LOC115548440	0.53													Hairy and enhancer of split 2/6/7
LOC115548456	0.63													THAP domain-containing protein 4
LOC115548460			0.46										K10360	Myosin IX
LOC115548464											Gain	Gain		
LOC115548468						-0.78								
LOC115548469				-0.53		-1.00							K06736	Cadherin 2, type 1, N-cadherin
LOC115548471						-0.36							K20131	Rab5 GDP/GTP exchange factor
LOC115548472							Loss							
LOC115548479							Loss						K00509	Prostaglandin-endoperoxide synthase 1
LOC115548485								Gain			Loss	Gain		
LOC115548492									Gain					
LOC115548496				0.42									K12076	Disks large protein 1
LOC115548504												Loss		FYN binding protein

LOC115548515				-0.38		-0.37								Solute carrier family 25 (mitochondrial carnitine/acylcarnitine transporter), member 20/29
LOC115548518				-0.45		-0.97								
LOC115548519			0.43											Deoxyribodipyrimidine photo-lyase
LOC115548521		-0.44												LIM domain and actin-binding protein 1
LOC115548525						-0.63							K17494	Cysteine/serine-rich nuclear protein
LOC115548526						-0.57								Serine/threonine-protein kinase OSR1/STK39
LOC115548527							Loss	Gain + Loss						MFS transporter, OCT family, solute carrier family 22 (organic cation transporter), member 13
LOC115548529								Gain + Loss			Gain	Gain		MFS transporter, OCT family, solute carrier family 22 (organic cation transporter), member 13
LOC115548537						0.27							K11293	Protein HIRA/HIR1
LOC115548542								Gain				Gain	K01990, K05644	ABC-2 type transport system ATP-binding protein, ATP-binding cassette, subfamily A (ABC1), member 4
LOC115548549				0.29									K09113	MAX-like protein X
LOC115548577						-0.81							K09232	Zinc finger protein GLIS1/3
LOC115548578				0.46										Apolipoprotein L
LOC115548593						0.60								
LOC115548602											Loss		K09228	KRAB domain-containing zinc finger protein
LOC115548605	0.43		0.37	-0.41		-0.38								Solute carrier family 39 (zinc transporter), member 6
LOC115548611						0.58						Loss		
LOC115548614				0.29		0.46							K08864	Tousled-like kinase
LOC115548615	0.41		0.55											
LOC115548621			-0.38									Gain		Tensin
LOC115548623				0.42		0.86								Rab effector myrip
LOC115548628				0.32										
LOC115548632						-0.71							K21436	Ankyrin repeat domain-containing protein 11/12
LOC115548646								Gain						
LOC115548653											Gain			
LOC115548672						0.53							K06210	Nicotinamide mononucleotide adenylyltransferase
LOC115548673						0.49								
LOC115548678						-0.83							K17254	Syntenin-1
LOC115548687	-0.64	-0.44	-0.46										K10369	Supervillin
LOC115548690												Loss		Tyrosine-protein kinase Lyn
LOC115548706						-0.52								Arylsulfatase B , arylsulfatase I/J

LOC115548720						0.37							K08215	MFS transporter, OCT family, solute carrier family 22 (organic anion transporter), member 23
LOC115548722						-1.19								
LOC115548724	0.56													Carboxy-terminal domain RNA polymerase II polypeptide A small phosphatase
LOC115548725								Loss			Loss	Loss		Nuclease HARBI1
LOC115548732						-0.80								
LOC115548739			-0.42											High affinity immunoglobulin epsilon receptor subunit gamma
LOC115548744						-0.59								
LOC115548746						-0.77							K23027	Myocilin
LOC115548747						1.12								Presqualene diphosphate phosphatase
LOC115548748											Gain			
LOC115548750						0.80							K00922	Phosphatidylinositol-4,5-bisphosphate 3-kinase catalytic subunit alpha/beta/delta
LOC115548762											Gain			
LOC115548763	-0.76													
LOC115548767	0.34		0.23										K04508	Transducin (beta)-like 1
LOC115548771						0.34								Neuropilin 1
LOC115548773						-0.75							K02375	Frizzled 5/8
LOC115548774												Loss		
LOC115548775						0.52								Partitioning defective protein 3
LOC115548776												Loss		Nuclease HARBI1
LOC115548777				-0.44		-0.76								
LOC115548778		-0.46	-0.39											
LOC115548783	0.33												K12391	AP-1 complex subunit gamma-1
LOC115548784			0.59										K13781	Solute carrier family 7 (L-type amino acid transporter), member 8
LOC115548787	0.40	0.36	0.42											Kinesin family member 5
LOC115548795											Loss			SPRY domain-containing SOCS box protein 1/4
LOC115548798				0.52										
LOC115548800	1.36	1.39	1.24	-0.50		-1.06								DNA replication licensing factor MCM6
LOC115548801						0.72								Caspase 8 , ciliogenesis-associated TTC17-interacting protein
LOC115548804		-0.42												Caspase 8
LOC115548807	-0.52	-0.40	-0.46										K24205	Protein lifeguard
LOC115548808						0.65								

LOC115548810		-0.29											
LOC115548822						0.84							
LOC115548830						0.56							Glypican 1
LOC115548834	0.40		0.33										Upstream-binding transcription factor
LOC115548839						0.53							Deoxynucleoside triphosphate triphosphohydrolase SAMHD1
LOC115548840	-0.44												
LOC115548841												Gain	K15688	E3 ubiquitin-protein ligase MUL1
LOC115548848												Loss		
LOC115548849								Loss						
LOC115548851			-0.41										K04448	Transcription factor AP-1
LOC115548854	1.42							Loss	Loss			Loss		Protein-lysine 6-oxidase
LOC115548855			-0.48										K18450	Caytaxin
LOC115548856				0.57		0.62								Nicotinamide/nicotinate riboside kinase
LOC115548857	-2.48	-0.73		-0.70		-0.81								
LOC115548860	-0.44		-0.44											Tensin
LOC115548863						-0.36								MAD, mothers against decapentaplegic interacting protein
LOC115548864	-0.59													Muscleblind
LOC115548868			-0.34											Transmembrane protein 106C
LOC115548869	-0.33	-0.52	-0.70										K15116	Solute carrier family 25, member 33/36
LOC115548873			-0.38			0.45		Loss					K07597	Desmoglein 2
LOC115548879						-0.43								
LOC115548887	0.44		0.52											Kinesin family member 2/24
LOC115548920	-0.42					-0.56							K04527	Insulin receptor
LOC115548924				-0.32									K05759	Profilin
LOC115548926	-0.45		-0.74											DET1- and DDB1-associated protein 1
LOC115548930	-0.43												K05619	Transglutaminase 1
LOC115548931				-0.54		-0.80							K09208	Krueppel-like factor 9/13/14/16
LOC115548932				-0.44		-0.69								
LOC115548934				-0.52		-1.14							K07998	Matrix metalloproteinase-19 (RASI)
LOC115548935						0.57								Collagen type V/XI/XXIV/XXVII, alpha
LOC115548944						0.68								
LOC115548954	-0.99		-0.60				Loss	Loss	Loss					
LOC115548967							Gain				Loss	Loss		

LOC115548976	0.35		0.61											Long-chain acyl-coa synthetase
LOC115548985						0.32							K03869	Cullin 3
LOC115548988							Loss	Loss						
LOC115548989				0.50		0.63								
LOC115549007						0.49								Trna selenocysteine 1-associated protein 1
LOC115549008				0.44										
LOC115549011	-0.86	-0.44	-0.83										K20052	Low-density lipoprotein receptor-related protein 8
LOC115549013	-1.56	-0.65	-1.69	0.55									K22808	KN motif and ankyrin repeat domain-containing protein
LOC115549021						-0.56							K20065	SH3-containing GRB2-like protein 3-interacting protein 1
LOC115549024						-0.59								Glutamine synthetase
LOC115549026	-0.74		-0.52											Ras-related protein Rab-18
LOC115549028									Loss			Loss	K16449	Regulator of G-protein signaling
LOC115549040											Gain	Gain	K21444	Poly(rc)-binding protein 3/4
LOC115549051						0.53								AFG3 family protein
LOC115549064	0.45		0.40											
LOC115549065	0.42	0.44	0.54			0.37							K17302	Coatomer subunit beta'
LOC115549068						1.58								Histone H3
LOC115549077								Loss			Loss			Olfactomedin-like protein 1/3
LOC115549078	0.46													Heterogeneous nuclear ribonucleoprotein C1/C2
LOC115549079	-0.58						Gain		Gain			Gain		
LOC115549086	1.12		0.51	-0.50									K17804	Mitochondrial import inner membrane translocase subunit TIM44
LOC115549089	0.34		0.33								Loss		K03783	Purine-nucleoside phosphorylase
LOC115549090			-0.47										K05003	Potassium inwardly-rectifying channel subfamily J member 10
LOC115549097			0.58											Glucuronosyltransferase
LOC115549104											Loss	Loss		
LOC115549120	0.48												K08111	Glypican 5
LOC115549121			-0.53											
LOC115549125	0.77		0.66											Protein transport protein SEC61 subunit gamma and related proteins
LOC115549129										Loss		Loss	K12488	Arf-GAP with SH3 domain, ANK repeat and PH domain-containing protein
LOC115549143						-0.48								
LOC115549146						2.18							K23483	Gamma-crystallin

LOC115549148						-0.57								Corticotropin releasing hormone receptor 1, corticotropin releasing hormone receptor 2
LOC115549152	-0.58					0.55								Krueppel-like factor 2
LOC115549160											Loss	Loss		
LOC115549164						1.12								Myosin heavy chain 1/2/3/4/8/13/7B/15, myosin heavy chain 6/7
LOC115549165	0.32													Apoptotic chromatin condensation inducer in the nucleus
LOC115549178						-0.63								
LOC115549181						0.60								
LOC115549200				-0.46		-0.33								
LOC115549201		-0.28												
LOC115549209				-0.41		-0.61							K04392, K04393, K07975	Cell division control protein 42, Ras-related C3 botulinum toxin substrate 1, Rho family, other
LOC115549211	0.40					0.46							K03386, K13279	Peroxiredoxin 1 , peroxiredoxin 2/4
LOC115549214	0.40		0.41										K09291	Nucleoprotein TPR
LOC115549220	0.56													
LOC115549222	0.66													
LOC115549226	0.60	0.44	0.55											
LOC115549227				0.39		0.53								Polypyrimidine tract-binding protein 2
LOC115549228				-0.42		-0.92								A-kinase anchor protein 2
LOC115549250							Loss	Loss	Loss		Gain + Loss	Gain + Loss		
LOC115549257											Loss		K17291	MARVEL domain-containing protein 2
LOC115549259	0.51		0.48											
LOC115549262	-0.43		-0.43			-0.64								Peripheral myelin protein 22
LOC115549264	-1.18													Calsequestrin 1
LOC115549265		0.37											K13293	Camp-specific phosphodiesterase 4
LOC115549269	-0.44			-0.37		-0.58								Transcription factor jun-D
LOC115549271												Loss	K01304	Pyroglutamyl-peptidase
LOC115549275		0.49												Transferrin receptor
LOC115549295				0.40		0.47								Cyclin-dependent kinase 17
LOC115549309						-1.65								Transmembrane inner ear expressed protein
LOC115549310	-0.68		-0.39										K10473	Kelch repeat and BTB domain-containing protein 5/10
LOC115549316						-0.74	Loss	Loss						

LOC115549317	0.30					-0.32								
LOC115549323						0.58								C1q-related factor
LOC115549330	-0.64		-0.91			0.63								CREB-regulated transcription coactivator 1
LOC115549374	0.59													General transcription factor IIIA, KRAB domain-containing zinc finger protein
LOC115549379	0.66			-0.55		-0.90								
LOC115549380						0.84							K08812	Cam kinase-like vesicle-associated
LOC115549383						-0.47								Leukotriene B4 receptor 2
LOC115549384						-0.69								
LOC115549385			0.60											
LOC115549387	-0.87	-0.69	-0.59											Phosphoglucomutase
LOC115549388						0.68								
LOC115549394	-0.74													Renin
LOC115549396								Gain	Gain					
LOC115549401						-0.79								
LOC115549407	-0.33		-0.31			-0.38			Gain			Gain	K07430	25/26-hydroxycholesterol 7alpha-hydroxylase
LOC115549410						0.55								Tripartite motif-containing protein 55
LOC115549424						-0.55							K14684	Solute carrier family 25 (mitochondrial phosphate transporter), member 23/24/25/41
LOC115549427												Loss	K06794	Neurocan core protein
LOC115549432						0.57							K09063	Transcription factor E2-alpha
LOC115549434						-0.74							K15686	RNA-binding protein MEX3
LOC115549437	-0.55					-0.58						Loss		Protein fem-1 homolog A/C
LOC115549438			-0.33			-0.37								Maternally affected uncoordination
LOC115549440						-0.68								RAR-related orphan receptor beta
LOC115549443	-0.33		-0.33			-0.30								Cell division cycle 20-like protein 1, cofactor of APC complex
LOC115549451	0.68													Signal-transducing adaptor protein 2
LOC115549452						0.49							K01076	Abhydrolase domain-containing protein 17
LOC115549456						-0.71								E3 ubiquitin-protein ligase RNF115/126
LOC115549461				-0.35		-0.56								
LOC115549462	-0.69		-0.61			-0.76								Ubiquitin-conjugating enzyme E2 R
LOC115549467									Loss		Gain	Gain		Hyperpolarization activated cyclic nucleotide-gated potassium channel 2
LOC115549474						-0.62								

LOC115549486								Loss				Loss		
LOC115549488						-0.53								
LOC115549491	0.60								Loss	Loss	Loss			
LOC115549494	-0.40					0.58							K08059	Interferon, gamma-inducible protein 30
LOC115549495						0.33							K08789	Microtubule-associated serine/threonine kinase
LOC115549499							Loss	Loss			Gain	Gain	K04189	C-X-C chemokine receptor type 4
LOC115549502	-0.53		-0.39			-0.42								Calcium/calmodulin-dependent 3',5'-cyclic nucleotide phosphodiesterase , FYVE and coiled-coil domain-containing protein 1
LOC115549503				-0.37		-0.47							K24495	Myomesin
LOC115549504			-0.58										K06560	Mannose receptor, C type
LOC115549505						-0.88								Emilin
LOC115549508						-1.08								Collectin sub-family member 12
LOC115549512							Gain		Gain			Loss		
LOC115549515	-0.43												K07606	Vimentin
LOC115549518								Loss			Loss	Loss		
LOC115549540				-0.51										
LOC115549552	-0.54												K03098	Apolipoprotein D and lipocalin family protein
LOC115549557						-0.61								
LOC115549559												Loss		
LOC115549569												Gain		E3 ubiquitin-protein ligase RNF31
LOC115549570						-0.76								
LOC115549574												Loss	K15377	Solute carrier family 44 (choline transporter-like protein), member 2/4/5
LOC115549575						0.61							K19530	Junctophilin
LOC115549579												Gain		
LOC115549582		-0.69	-0.71											Glutamate decarboxylase
LOC115549591	0.49												K01764	Cytochrome c heme-lyase
LOC115549600	0.40												K18173	Cytochrome c oxidase assembly factor 1
LOC115549611						0.80							K14951	Cation-transporting P-type atpase 13A3/4/5
LOC115549615				0.42										B-cell lymphoma 6 protein, general transcription factor IIIA
LOC115549616	0.58			-0.60										
LOC115549625		-0.45												Flap endonuclease-1
LOC115549631				-0.51		-0.82							K22076	Mitochondrial fission factor

LOC115549644						3.36								Deoxynucleoside triphosphate triphosphohydrolase SAMHD1
LOC115549650						-0.70					Gain	Gain	K23860	Pleckstrin homology domain-containing family G member 4
LOC115549660						0.34								AP-2 complex subunit mu-1
LOC115549663						-0.40								Segment polarity protein dishevelled
LOC115549671									Gain					Sterol 12-alpha-hydroxylase
LOC115549672						0.64								
LOC115549680	0.78		0.59											Uridine kinase
LOC115549694				0.42									K07189	Protein phosphatase 1 regulatory subunit 3A/B/C/D/E
LOC115549713						0.75								
LOC115549746	0.80	1.26	0.64			-0.78							K04465	Nuclear receptor subfamily 4 group A member 1
LOC115549883			0.64										K07374	Tubulin alpha
LOC115549925						0.66								
LOC115549942	0.54													
LOC115549974				0.55		0.52								
LOC115550006	0.55					0.55								26S proteasome regulatory subunit N13
LOC115550094	0.76	0.62	0.58										K17399	DNA (cytosine-5)-methyltransferase 3B
LOC115550233											Loss	Loss		
LOC115550244						0.70								
LOC115550249	-0.55		-0.67											
LOC115550251	-0.46		-0.31										K18749	Protein LSM14
LOC115550272						0.38							K11279	Nucleosome assembly protein 1-like 1
LOC115550281											Loss		K13748	Alpha-1,3-mannosylglycoprotein beta-1,4-N-acetylglucosaminyltransferase C
LOC115550285	0.37			-0.36		-0.46								Dynamin 1-like protein
LOC115550293						-0.78							K06509	CD82 antigen
LOC115550296						-0.72								Protein O-mannosyl-transferase
LOC115550298	-1.74	-0.88	-1.71										K10435	Microtubule-associated protein 1 light chain
LOC115550300						0.58								
LOC115550308	-0.64													Tetraspanin-33
LOC115550309	0.67					0.75								Tetraspanin-9
LOC115550317				-0.93		-1.27								
LOC115550322	0.82													P-type Ca2+ transporter type 2B
LOC115550326							Gain + Loss	Loss	Loss		Gain	Gain + Loss	K19241	Engulfment and cell motility protein 3

LOC115550331		-0.38	-0.45			-0.74								Ceramide kinase
LOC115550332				0.37									K08150	MFS transporter, SP family, solute carrier family 2 (myo-inositol transporter), member 13
LOC115550339	0.78													
LOC115550350				-0.50		-0.58							K09525	Dnaj homolog subfamily C member 5
LOC115550365							Gain	Gain	Gain					
LOC115550366						0.68								Fibroblast growth factor
LOC115550367						-0.85								
LOC115550375			-0.70	-0.60		-0.89						Gain		
LOC115550378						-1.23								
LOC115550383						-0.45							K06460	CD9 antigen
LOC115550384			-23.08			1.69								
LOC115550385			0.40											FK506-binding protein 1
LOC115550387						1.11							K03783	Purine-nucleoside phosphorylase
LOC115550389									Gain			Gain		Purine-nucleoside phosphorylase
LOC115550391				0.38										
LOC115550395	0.51													Zinc finger protein AEBP2
LOC115550405							Loss							Transient receptor potential cation channel subfamily M member 1
LOC115550406	0.54												K09502	Dnaj homolog subfamily A member 1
LOC115550420	-0.74													
LOC115550423		-0.37				-0.39							K12495	IQ motif and SEC7 domain-containing protein
LOC115550427				0.46									K25830	Cat eye syndrome critical region protein 2
LOC115550437						-0.33								Disintegrin and metalloproteinase domain-containing protein 10
LOC115550438						-0.64							K09877	Aquaporin-9
LOC115550439	0.79													
LOC115550445						0.62								
LOC115550446	-0.84	-0.57	-1.02	-0.56		-0.45								
LOC115550447	0.67					-0.75								
LOC115550450	-0.48		-0.52											Aryl hydrocarbon receptor nuclear translocator-like protein 1
LOC115550453						-1.07								PDZ domain-containing protein 4
LOC115550466				-0.41		-0.54								ADP-ribosylation factor 1/2, ADP-ribosylation factor 3
LOC115550468				-0.39										Translation initiation factor 3 subunit J
LOC115550481	-0.44					-0.57								

LOC115550483						-0.73								K12327	Caldesmon
LOC115550484						-0.68									Myogenic factor 3
LOC115550485	-0.51													K12046	Troponin T, fast skeletal muscle
LOC115550488													Gain	K06256	CD44 antigen
LOC115550492				0.42											
LOC115550506			-0.74												NUAK family, SNF1-like kinase
LOC115550507						0.63								K12043	Troponin I, fast skeletal muscle
LOC115550508	0.75													K12043	Troponin I, fast skeletal muscle
LOC115550510	-0.55													K12043	Troponin I, fast skeletal muscle
LOC115550522													Loss		
LOC115550523						-0.69									
LOC115550531	-3.28	-1.68	-3.24											K16815	Calcium-independent phospholipase A2-gamma
LOC115550532	-0.77	-0.76	-1.48												Dnaj homolog subfamily B member 9
LOC115550536	0.33	0.38				-0.26								K13207	CUG-BP- and ETR3-like factor
LOC115550541								Loss	Loss				Gain + Loss		
LOC115550544	-0.71	-0.59	-0.96											K16613	Ornithine decarboxylase antizyme 2
LOC115550548	-0.99		-0.70												Insulin-like growth factor 1 receptor
LOC115550552			-0.49											K25447	Olfactomedin-4
LOC115550554	-0.47	-0.57	-0.76											K02833, K07827	Gtpase hras, gtpase kras
LOC115550558	0.53														
LOC115550559						-0.32									Septin 7
LOC115550561						-0.48									
LOC115550563													Gain		
LOC115550571		0.33	0.47												Calumenin
LOC115550580						0.66								K03260	Translation initiation factor 4G
LOC115550582				0.47		0.76									Ras and EF-hand domain-containing protein
LOC115550584				0.74		0.68								K09574	FK506-binding protein 8
LOC115550594							Gain								Interferon induced transmembrane protein
LOC115550596								Loss							Interferon induced transmembrane protein
LOC115550604						-0.40			Loss		Loss				Neuronal cell adhesion molecule
LOC115550608	0.59													K06632	Wee1-like protein kinase
LOC115550615	-0.50														BH3 interacting domain death agonist

LOC115550623	-0.47												K04441	P38 MAP kinase
LOC115550626						-0.71							K20164	DENN domain-containing protein 5
LOC115550638						-0.71							K17047	Ras-related protein Rab-19
LOC115550649	0.49												K11413	NAD+-dependent protein deacetylase sirtuin 3
LOC115550650		0.40	0.71											Myosin V
LOC115550659						-0.79								Pyruvate kinase
LOC115550661	0.29			-0.32										Lamina-associated polypeptide 2
LOC115550663	-0.25		-0.30										K10582	Ubiquitin-conjugating enzyme E2 Q
LOC115550664						0.77							K00016	L-lactate dehydrogenase
LOC115550667												Loss		Phosphatidylinositol-4,5-bisphosphate 3-kinase catalytic subunit gamma
LOC115550668	0.82		0.91			0.38								
LOC115550670												Loss		Hyperpolarization activated cyclic nucleotide-gated potassium channel 4
LOC115550677						0.37							K17092	Annexin A2
LOC115550678			0.31											Guanine nucleotide-binding protein G(i) subunit alpha
LOC115550683						-0.51							K04226	Arginine vasopressin receptor 1A
LOC115550686	-0.95						Loss							
LOC115550687	0.73	0.59	0.46											
LOC115550694	-1.06												K07434	Aromatase
LOC115550702						-0.65								Cgmp-inhibited 3',5'-cyclic phosphodiesterase A
LOC115550719	0.75	0.65	0.67			0.50							K04079, K09487	Heat shock protein 90kda beta, molecular chaperone htpg
LOC115550725	0.97												K08956	AFG3 family protein
LOC115550741						0.67								NLR family CARD domain-containing protein 3
LOC115550744							Loss				Loss	Gain		
LOC115550748	0.60					0.90							K04437	Filamin
LOC115550761				-0.56		-0.48								G1/S-specific cyclin-D1
LOC115550766			0.52											Cytochrome-b5 reductase
LOC115550777	-1.77	-0.50			3.84									
LOC115550779	-0.69													
LOC115550784	-0.72												K05632	E3 ubiquitin-protein ligase Itchy
LOC115550790				-0.50									K15121	Solute carrier family 25, member 44
LOC115550797			-0.26			-0.26							K16529	A-kinase anchor protein 13

LOC115550802						-0.43								
LOC115550804				0.43										Hexosaminidase
LOC115550811							Gain		Gain				K01362	Ovochymase
LOC115550813										Gain		Gain		Zinc finger BED domain-containing protein 1 (E3 SUMO-protein ligase ZBED1)
LOC115550823											Gain	Gain		Anoctamin-2
LOC115550824						-0.76								Proprotein convertase subtilisin/kexin type 5
LOC115550851						-0.34								Transcription initiation factor TFIID subunit 4
LOC115550853						-0.78					Gain		K21754	BTB/POZ domain-containing protein KCTD1/15
LOC115550855						-0.47								KIT ligand
LOC115550866				-0.51		-0.43							K00522	Ferritin heavy chain
LOC115550867						-0.45							K21599	Circadian-associated transcriptional repressor
LOC115550868				-0.57		-1.15								Semaphorin 4
LOC115550872			-0.44											Metastasis suppressor protein 1
LOC115550873				0.42				Gain					K08670	Suppressor of tumorigenicity protein 14
LOC115550882				-0.49										
LOC115550891				0.45										
LOC115550894	0.38					-0.51								Ankyrin repeat domain-containing protein 26
LOC115550901				0.42		0.47							K16779	Rab-3A-interacting protein
LOC115550909			-0.63										K10376	Desmuslin
LOC115550912	-0.72	-0.46	-1.15										K07369	Mucosa-associated lymphoid tissue lymphoma translocation protein 1
LOC115550913						-0.66								Parathyroid hormone-related hormone
LOC115550916						-0.47								
LOC115550917				-1.02										
LOC115550919											Loss	Loss		
LOC115550920						0.87								Interferon induced transmembrane protein
LOC115550921						1.12								
LOC115550925						0.59								Poly
LOC115550929				-0.50										
LOC115550934						-0.59								
LOC115550943						-0.33								
LOC115550944											Loss			Nuclease HARBI1
LOC115550948						-0.65								

LOC115550957												Gain		
LOC115550973							Loss	Loss			Loss	Loss		
LOC115550974			-0.54											
LOC115550976			-0.56											Pyrimidine nucleoside transport protein
LOC115550977				-0.34		-0.67								
LOC115550981						-0.50								Choline/ethanolamine kinase
LOC115550982			-0.59											
LOC115550985						0.68								
LOC115550991												Gain		
LOC115550997												Loss		Aminopeptidase N
LOC115551004							Gain	Gain				Loss	K25537	Fibroleukin
LOC115551006	0.31	0.27	0.25	0.35		0.27							K08867	WNK lysine deficient protein kinase
LOC115551007				-0.54		-0.97							K09210	Krueppel-like factor 15
LOC115551011			0.27											Lysine-specific demethylase 9
LOC115551014	0.33												K13024	Inositol-hexakisphosphate/diphosphoinositol-pentakisphosphate 1-kinase
LOC115551017						1.21								Anoctamin-1
LOC115551020	-1.31		-1.24										K02295	Cryptochrome
LOC115551026	0.35		0.47										K20813	Thymine-DNA glycosylase
LOC115551027	-3.42		-2.59											Patatin-like phospholipase domain-containing protein 2
LOC115551031	-0.77	-0.42	-0.82											Lactadherin
LOC115551032						0.62							K03927	Carboxylesterase 2
LOC115551035	-0.57		-0.69			-0.65								Proline-rich protein 5
LOC115551040				0.53		0.47								Abhydrolase domain-containing protein 2
LOC115551043	-3.19	-1.26	-1.87											
LOC115551044						-0.59								Creatine kinase
LOC115551048			-0.45											Hyaluronan and proteoglycan link protein 3
LOC115551049				-0.42										
LOC115551055	-2.87	-1.97	-2.97											
LOC115551063				-0.51		-0.91								
LOC115551065	-0.78													Cytochrome c oxidase subunit 8
LOC115551072	-0.46													
LOC115551073			0.31										K20366	Endoplasmic reticulum-Golgi intermediate compartment protein 2

LOC115551076			0.48											Centriolar protein POC1
LOC115551091			-0.42											
LOC115551109	-0.81		-0.57	0.67		0.75							K04577	Calcitonin receptor-like
LOC115551110						-0.48								
LOC115551128			0.65											Rho guanine nucleotide exchange factor 3/8
LOC115551133											Gain			
LOC115551135						-0.49								N-acetylglucosaminyltransferase 3, mucin type
LOC115551145											Gain	Gain		
LOC115551151	-1.04		-0.52										K20412	Peptidase inhibitor 16
LOC115551164	-0.78					-0.67								Cytosolic carboxypeptidase protein 4
LOC115551166	0.43	0.39	0.58											
LOC115551169						0.61							K08539	Vitamin D3 receptor
LOC115551172						0.59							K06270, K17457	Protein phosphatase 1 regulatory subunit 12A, protein phosphatase 1 regulatory subunit 12C
LOC115551174			0.51			0.79							K11515	Inner centromere protein
LOC115551175						0.76								
LOC115551191			-0.34										K23605	Mothers against decapentaplegic homolog 3
LOC115551199	0.53					-0.43								Mortality factor 4-like protein 1
LOC115551200						-0.59	Loss	Loss	Loss	Loss	Gain	Gain + Loss		Solute carrier family 25 (mitochondrial glutamate transporter), member 18/22
LOC115551211	1.65													
LOC115551213						-0.56							K19987	Exocyst complex component 3-like protein
LOC115551216	0.36													
LOC115551217	-0.71	-0.40	-0.47											
LOC115551221									Gain					Cholesterol 25-hydroxylase
LOC115551249		0.41												Seipin
LOC115551255							Loss		Loss					
LOC115551262				-0.45										G protein-coupled receptor 22
LOC115551268						-0.48								BTB/POZ domain-containing protein 11
LOC115551270	0.31													Cholesterol monooxygenase (side-chain-cleaving)
LOC115551275							Gain		Gain		Gain	Gain		Furin
LOC115551278				-0.39										Neuropeptides B/W receptor 2
LOC115551279						0.60								
LOC115551280						0.66								

LOC115551289			-0.47											
LOC115551290	-0.54													
LOC115551293							Loss					Gain		
LOC115551294						0.46								Transcription elongation factor S-II
LOC115551295	-0.93	-0.40		0.43										
LOC115551298	-0.37					-0.38								
LOC115551299	-0.39	-0.46	-0.67											Transcriptional enhancer factor
LOC115551301	-0.81	-0.44												Delta7-sterol 5-desaturase
LOC115551303						0.69								
LOC115551304				-0.41									K25493	Dickkopf 3
LOC115551312				0.97		0.80							K05152	Tumor necrosis factor receptor superfamily member 14
LOC115551317												Gain		
LOC115551325				0.49										
LOC115551335	-0.48		-0.53											Carnitine O-palmitoyltransferase 1, liver isoform
LOC115551336	-0.39												K19327	Anoctamin-10
LOC115551344			-1.08											
LOC115551349				-0.42		-0.72								Choline/ethanolamine kinase
LOC115551356							Gain							
LOC115551368	-0.56												K01672	Carbonic anhydrase
LOC115551373	-0.54	-0.37	-0.63	-0.61										Hypoxanthine phosphoribosyltransferase
LOC115551376				-0.52		-0.62							K05694	Receptor-type tyrosine-protein phosphatase beta
LOC115551380							Loss					Gain		
LOC115551384						-1.00								
LOC115551385	0.55													Protein phosphatase 1 regulatory subunit 3A/B/C/D/E
LOC115551399				-0.40									K00411	Ubiquinol-cytochrome c reductase iron-sulfur subunit
LOC115551400				-0.37										Protein MPAN
LOC115551401	0.45												K19041	E3 ubiquitin-protein ligase RNF38/44
LOC115551404							Loss	Loss	Loss					IQ motif and SEC7 domain-containing protein
LOC115551407		0.46	0.39										K24727	Myogenesis-regulating glycosidase
LOC115551414				-0.93		-1.14							K04349	Ras-specific guanine nucleotide-releasing factor 1
LOC115551418									Gain					
LOC115551420						-0.65								Netrin-G1 ligand
LOC115551422	-0.49													Gliomedin

LOC115551426				-0.42		-0.83		Gain						
LOC115551433	-0.82					-0.57								AMP deaminase
LOC115551441	0.50												K26164	Protein RIC-3
LOC115551447	0.67												K12333	Adrenomedullin
LOC115551452											Loss	Loss		
LOC115551464						-0.85								
LOC115551483	-0.59												K25809	Kelch domain-containing protein 10
LOC115551484						1.11								Type I protein arginine methyltransferase
LOC115551485	-0.97	-0.56	-1.10			-0.50							K10576	Ubiquitin-conjugating enzyme E2 H
LOC115551493	-0.85		-0.62										K17941	Sorting nexin-22/24
LOC115551505						-0.61							K04903	Potassium voltage-gated channel subfamily G member 4
LOC115551515			-0.42				Gain	Gain	Gain				K15009	SH3 and multiple ankyrin repeat domains protein
LOC115551516						0.54								Rab-like protein 2
LOC115551536												Loss	K25343	Adrenomedullin-2
LOC115551613												Gain		NLR family CARD domain-containing protein 3
LOC115551675						0.70								
LOC115551677						0.39								
LOC115551697				-0.41										
LOC115551763						0.54					Gain			Arginine vasopressin receptor 2
LOC115551821						0.68							K05771	Glucocorticoid receptor
LOC115551822					0.92									BTB/POZ domain-containing protein KCTD8/12/16
LOC115551823						0.75								NEDD4 family-interacting protein
LOC115551824				-0.41		-1.21							K18496	Fibroblast growth factor 1
LOC115551825							Gain						K04915	Potassium channel subfamily K member 4
LOC115551827											Loss			Annexin A5
LOC115551829											Gain	Gain	K24423	Zinc finger protein 185
LOC115551833			-0.42										K07900	Ras-related protein Rab-9B
LOC115551837	-0.66		-0.35										K01303	Acylaminoacyl-peptidase
LOC115551856						-0.79								
LOC115551868	-0.62													Cytochrome P450 family 3 subfamily A
LOC115551881						0.61								
LOC115551883						0.87								Transcriptional activator protein Pur-alpha
LOC115551885	-0.73		-0.58										K14209	Solute carrier family 36 (proton-coupled amino acid transporter)

LOC115551886						-0.71								
LOC115551893	0.43													
LOC115551902	0.43		0.36										K13217	Pre-mrna-processing factor 39
LOC115551903		0.43	0.44											EH domain-containing protein 1
LOC115551909				0.54										
LOC115551911						0.92								
LOC115551921	-0.65													
LOC115551925	0.46													Mitochondrial import receptor subunit TOM5, zinc finger and BTB domain-containing protein 5
LOC115551931						-0.61								
LOC115551937						0.59								
LOC115551939			0.44											
LOC115551941									Loss					
LOC115551942							Loss		Loss					
LOC115551944				0.54		0.61								
LOC115551947						1.46							K23482	Beta-crystallin
LOC115551949						2.41							K23482	Beta-crystallin
LOC115551953											Gain			Calcium-binding protein 1/2/4/5
LOC115551956						1.65								L-threonylcarbamoyladenylate synthase , uncharacterized protein
LOC115551963						0.69								
LOC115551970	-0.50		-0.59	0.65										
LOC115551975	0.56													
LOC115552009											Loss	Loss		
LOC115552011	0.31													
LOC115552017			0.45											
LOC115552018						0.61							K26107	Fibroblast growth factor receptor-like 1
LOC115552024	-0.71												K23843	Calcium uptake protein 3, mitochondrial
LOC115552031						-0.83						Gain		
LOC115552040						-0.52								Atlastin
LOC115552045						0.99								
LOC115552049			-0.40			-0.93								Heparan sulfate 6-O-sulfotransferase HS6ST2
LOC115552050				-0.29										
LOC115552054	-0.54													

LOC115552059						-0.53								
LOC115552062	-0.78		-1.09											Translation initiation factor 4E
LOC115552063			-0.27											RUN and FYVE domain-containing protein 1/2
LOC115552078	0.53													Mitochondrial inner membrane protein COX18
LOC115552095	-0.72		-0.38	-0.48										
LOC115552102											Gain	Gain		Glycoprotein-N-acetylgalactosamine 3-beta-galactosyltransferase
LOC115552112							Loss	Loss	Loss					G protein-coupled receptor 151
LOC115552113						-0.50								Serine/threonine-protein phosphatase 2A regulatory subunit B
LOC115552115						-0.86							K05240	Tachykinin 3
LOC115552116	0.44													Protein transport protein SEC20
LOC115552122						0.64					Loss	Gain + Loss		Solute carrier family 44 (choline transporter-like protein), member 1
LOC115552126		-0.44												
LOC115552127				0.41		0.45								V-type H+-transporting atpase subunit a
LOC115552129						-0.48								
LOC115552137						-0.81								
LOC115552143					0.87						Gain			FMS-like tyrosine kinase 4
LOC115552159						-0.75								SH3 and cysteine-rich domain-containing protein 3
LOC115552162						0.70							K16497	Protocadherin gamma subfamily C
LOC115552167	-1.07		-0.63										K15107	Solute carrier family 25 (mitochondrial glutamate transporter), member 18/22
LOC115552170						0.50								
LOC115552183						0.70								
LOC115552213				-0.51		-0.68								
LOC115552215	-0.57		-0.38											Glucosamine-6-phosphate deaminase
LOC115552221			0.25											MAP7 domain-containing protein 3
LOC115552249			-0.39											
LOC115552250						-0.47								Ase1/PRC1/MAP65 family protein, E3 ubiquitin-protein ligase RNF38/44
LOC115552251	0.57		0.46											Protein AIR1/2
LOC115552261							Gain	Loss	Gain				K23222	Nuclease HARBI1
LOC115552263	0.29													Ubiquitin-conjugating enzyme E2 D
LOC115552275			0.61										K03095	Sprt-like protein
LOC115552278	0.57		0.55										K16860	Phospholipase D3/4

LOC115552280						-0.32							K16790	Mothers against decapentaplegic homolog 5
LOC115552291				0.46		0.58							K13196	Zinc finger protein ubi-d4
LOC115552293	-1.34	-0.90	-0.91	0.64										
LOC115552298				0.46										NLR family CARD domain-containing protein 3
LOC115552300	0.48		0.58											
LOC115552301						-0.35							K15588	
LOC115552306						-0.51								Serine/threonine-protein phosphatase 2A catalytic subunit
LOC115552308				-0.36		-0.65					Loss			
LOC115552311											Loss			
LOC115552333							Loss		Loss				K04274	P2Y purinoceptor 10
LOC115552335						0.51								Midline 2
LOC115552336						0.54							K06532	Prominin 1
LOC115552337			-0.33										K15909	Phosphatidylinositol-3,4,5-trisphosphate 5-phosphatase 2
LOC115552347											Gain	Gain		Glutamate receptor-interacting protein
LOC115552353							Loss	Loss	Loss					
LOC115552354						0.56								
LOC115552356				-0.55		-0.60					Gain	Loss		Protein RD3
LOC115552357						-0.45								
LOC115552359				0.47		0.59								
LOC115552361											Loss	Loss	K01490	AMP deaminase
LOC115552369	-0.92		-0.39										K23518	O-acetyl-ADP-ribose deacetylase
LOC115552372	-1.14						Gain							
LOC115552378	-0.63													
LOC115552388						-0.45								Ras-related protein Rap-2C
LOC115552390	-0.62													Angiotensin II receptor type 2
LOC115552399		-0.38				-0.75								
LOC115552400	-1.43	-0.92	-1.04			-0.44							K11318	Ataxin-7
LOC115552401	-1.01	-0.42	-0.60											
LOC115552408	0.45													Polyamine-modulated factor 1
LOC115552409						0.55								Nucleophosmin 1
LOC115552413						-0.65							K13864	Solute carrier family 7 (cationic amino acid transporter), member 2
LOC115552416	1.00					0.64							K03283	Heat shock 70kda protein 1/2/6/8

LOC115552421						-0.87								
LOC115552424	-0.61					-0.70								Solute carrier family 6 (neurotransmitter transporter, taurine) member 6
LOC115552430			0.48										K06267	Hyaluronan-mediated motility receptor
LOC115552437			0.38	0.43									K11499	Centromere protein F
LOC115552438	0.50	0.56	0.58											Cytoplasmic polyadenylation element-binding protein
LOC115552440						-1.05								Dapper
LOC115552444				-0.49		-0.97								Myotilin
LOC115552453						-0.53								
LOC115552459							Loss		Loss					
LOC115552472						0.60								Fatty acid-binding protein 6, ileal (gastrotropin)
LOC115552482								Loss						
LOC115552489											Gain	Gain		
LOC115552490						-0.48								
LOC115552491						0.65								
LOC115552500				-0.52		-0.35							K24626	MICOS complex subunit MIC27
LOC115552501									Loss					
LOC115552507											Gain			Testican
LOC115552517						0.67							K00948	Ribose-phosphate pyrophosphokinase
LOC115552522	0.72	0.37	0.46			-0.62								
LOC115552524	0.24		0.28										K13111	WD40 repeat-containing protein SMU1
LOC115552527				0.44									K23727	Sphingoid base N-palmitoyltransferase
LOC115552528											Gain			
LOC115552530	-0.36		-0.36	0.33									K13213	Matrin 3
LOC115552537				-0.48		-0.82								
LOC115552541				-0.51		-0.99								Protocadherin delta 2
LOC115552544	-0.74		-0.67											Tripartite motif-containing protein 69
LOC115552547						0.54								
LOC115552553												Gain	K14683	Solute carrier family 34 (sodium-dependent phosphate cotransporter)
LOC115552557						-0.43							K12603	CCR4-NOT transcription complex subunit 6
LOC115552558	0.41		0.51											C-terminal binding protein
LOC115552559									Gain				K13700	Abhydrolase domain-containing protein 6
LOC115552561	-0.48													

LOC115552562	-0.75													
LOC115552566						-0.57								
LOC115552568				-0.41										
LOC115552579						0.91								
LOC115552584			-0.40											
LOC115552586						-0.68								Ubiquitin-conjugating enzyme (huntingtin interacting protein 2)
LOC115552590	-0.57		-0.59										K03899	Coagulation factor VIII
LOC115552604				-0.68		-0.90								
LOC115552613						0.63								NADH dehydrogenase (ubiquinone) 1 alpha subcomplex subunit 4
LOC115552614												Loss		HRAS-like suppressor 3
LOC115552623				0.76										Alpha-1,3-mannosylglycoprotein beta-1,4-N-acetylglucosaminyltransferase A/B
LOC115552627			-0.56										K08523	Heparin-binding EGF-like growth factor
LOC115552639						0.49							K20792	N-alpha-acetyltransferase 15/16, nata auxiliary subunit
LOC115552642	0.36			-0.51										
LOC115552643						-1.06							K18764	Nocturnin
LOC115552648						0.98								
LOC115552650				0.44										
LOC115552655											Gain	Gain		
LOC115552668						-0.40								Tyrosine-protein kinase Src
LOC115552672						0.60							K16827	Signal-regulatory protein delta
LOC115552685				0.43										E3 ubiquitin-protein ligase SH3RF
LOC115552692											Gain	Gain	K04810	Nicotinic acetylcholine receptor alpha-9
LOC115552698				-0.41		-0.84							K04236	Coagulation factor II (thrombin) receptor-like 3
LOC115552702							Loss		Loss					
LOC115552712						1.05								
LOC115552714						0.38								
LOC115552719											Gain			Seipin
LOC115552720	0.60												K01897	Long-chain acyl-coa synthetase
LOC115552748						0.61								
LOC115552749												Gain		
LOC115552757						-0.64								
LOC115552764	0.43					-0.60								

LOC115552769						0.49								
LOC115552772						1.04								
LOC115552778						-0.70						Gain		Gap junction alpha-3 protein
LOC115552779											Loss	Loss	K07620	Gap junction beta-1 protein
LOC115552792				0.39									K04808	Nicotinic acetylcholine receptor alpha-6
LOC115552794	-1.28	-1.04	-1.48											
LOC115552800				-0.46									K03020	DNA-directed RNA polymerases I and III subunit RPAC2
LOC115552801						-1.10								
LOC115552803	0.47												K05764	Thymosin, beta 4
LOC115552811												Loss	K05152	Tumor necrosis factor receptor superfamily member 14
LOC115552812											Loss	Loss		
LOC115552823				-0.42				Loss	Loss				K00911	1D-myo-inositol-triphosphate 3-kinase
LOC115552847						-0.52							K24142	Star-related lipid transfer protein 10
LOC115552858												Loss		
LOC115552861						0.62								
LOC115552866						-0.67								
LOC115552867						-0.61							K18487	Zinc finger protein ZIC 3
LOC115552868						1.11								Glycine receptor alpha-4
LOC115552873											Loss			
LOC115552881						0.58								
LOC115552888			-0.59											Protein bicaudal C
LOC115552899											Gain	Gain		
LOC115552909							Gain							
LOC115552912			0.41											MAX dimerization protein
LOC115552914						-0.74					Gain	Gain		
LOC115552916			-0.43											
LOC115552919	-1.39	-0.66	-1.63											Forkhead box protein O4
LOC115552920											Loss	Loss		
LOC115552921						0.96								
LOC115553049						0.89								
LOC115553141				0.47									K18024	Tyrosine-protein phosphatase non-receptor type 12/18/22
LOC115553203						0.43							K05152	Tumor necrosis factor receptor superfamily member 14
LOC115553252						-0.53								Collagen type VI alpha

LOC115553254											Loss	Loss		Collagen type XXVIII alpha
LOC115553270						0.70							K16944	Septin 7
LOC115553285		-0.44		0.48									K13362	FXYD domain-containing ion transport regulator 5
LOC115553299											Gain		K16665	Leucine-rich repeat transmembrane neuronal protein 1/2
LOC115553315	0.77			0.40		1.01								
LOC115553333				-1.70										Apolipoprotein A-IV
LOC115553336						1.30								
LOC115553339												Loss		
LOC115553356						-1.01							K09191, K23480	General transcription factor IIIA, zinc finger protein 362/384
LOC115553360									Loss		Loss	Gain	K04135	Adrenergic receptor alpha-1A
LOC115553361	0.37	0.27	0.42											Serine/threonine-protein phosphatase 2A regulatory subunit B
LOC115553362	-0.89	-0.46	-0.94			-0.80							K15465	BCL2/adenovirus E1B 19 kda protein-interacting protein 3-like
LOC115553363						0.59								MADS-box transcription enhancer factor 2D
LOC115553364	-0.57													Voltage-dependent calcium channel L type alpha-1S
LOC115553368										Gain				Ammonium transporter Rh, solute carrier family 12 (potassium/chloride transporter), member 5
LOC115553373						0.69								
LOC115553376						-0.80								
LOC115553378	0.69	0.70	0.67											
LOC115553383				0.47									K05081	Prolactin receptor
LOC115553390						-0.53								
LOC115553391		0.41	0.41										K08802	SNF related kinase
LOC115553399				-0.33		-0.58							K13126	Polyadenylate-binding protein
LOC115553402						0.67								
LOC115553404	-0.86	-0.40	-1.37											Protein tyrosine phosphatase type IVA
LOC115553406	-0.53													L-serine/L-threonine ammonia-lyase
LOC115553411						-0.42								Ephrin-A
LOC115553413	0.43													Ephrin-A
LOC115553436	-0.42													
LOC115553439						0.55								
LOC115553442	0.52	0.29	0.54										K07375	Tubulin beta
LOC115553446	-0.33		-0.32										K10421	CAP-Gly domain-containing linker protein 1
LOC115553452						0.62							K04572	Neurofilament light polypeptide

LOC115553458							Loss						K08143	MFS transporter, SP family, solute carrier family 2 (facilitated glucose/fructose transporter), member 5
LOC115553461	0.86						Gain		Loss		Gain + Loss	Gain + Loss		
LOC115553485	-0.72	-0.46	-0.81											A-kinase anchor protein 28
LOC115553489						0.37								Hippocalcin-like protein 1, neurocalcin delta
LOC115553491	0.43	0.29	0.42			0.30								Dolichyl-diphosphooligosaccharide---protein glycosyltransferase
LOC115553497	0.40													Glycerol-3-phosphate dehydrogenase (NAD+)
LOC115553498	0.36			-0.54		-0.48								
LOC115553505						-0.52								
LOC115553506	-1.12		-1.49										K20910	Thioredoxin-interacting protein
LOC115553507	-1.21	-0.79	-1.29											
LOC115553512						-0.81							K08121	Fibromodulin
LOC115553520	0.51	0.47	0.81										K00287	Dihydrofolate reductase
LOC115553523						0.67								
LOC115553527			0.41											Inactive phospholipase C-like protein 2
LOC115553536						0.67								Macrophage colony-stimulating factor 1
LOC115553538	0.45			-0.31		-0.25								
LOC115553553							Loss							
LOC115553556				0.59										
LOC115553558	-0.73												K08148	MFS transporter, SP family, solute carrier family 2 (facilitated glucose transporter), member 11
LOC115553576	-0.37		-0.34			-0.45								SPRY domain-containing SOCS box protein 1/4
LOC115553579				0.70									K18027	Tyrosine-protein phosphatase non-receptor type 3
LOC115553586						-0.67							K25006	RNA-binding protein 24/38
LOC115553590	-0.80	-0.42	-0.72										K11860	OTU domain-containing protein 7
LOC115553594						0.49	Loss							Lysosome membrane protein 2
LOC115553598				-0.40									K02978	Small subunit ribosomal protein s27e
LOC115553603				-0.41										Tumor necrosis factor receptor superfamily member 11B
LOC115553625	-2.24	-0.97	-2.14											Kelch-like protein 38
LOC115553626						-0.74					Gain			
LOC115553629				-0.42										Elongation of very long chain fatty acids protein 4
LOC115553634							Gain	Gain	Gain			Gain		
LOC115553641												Gain		

LOC115553652				0.46		0.73								
LOC115553665	0.63		0.42			0.68								
LOC115553684						-0.54							K06055	Hairy and enhancer of split 5
LOC115553685								Gain						
LOC115553686							Gain				Loss			
LOC115553694	-0.55		-0.49										K19876	Metalloreductase STEAP4
LOC115553695												Gain		
LOC115553696				0.47		0.75								
LOC115553719	-0.50		-0.33										K06842	Semaphorin 6
LOC115553727						0.75								G protein-coupled receptor kinase
LOC115553729	-0.83	-1.64	-0.55											Serpin A
LOC115553732						0.66							K14165	Atypical dual specificity phosphatase
LOC115553734											Loss	Loss	K21392	Adipocyte enhancer-binding protein 1
LOC115553748				-0.46										
LOC115553758						-0.40								Centrosomal protein CEP76, spire
LOC115553761						0.69							K20647	Rho gtpase-activating protein 32
LOC115553764	0.43												K04348	Serine/threonine-protein phosphatase 2B catalytic subunit
LOC115553766						0.49								Cytochrome-b5 reductase
LOC115553772						0.55							K16063	E3 SUMO-protein ligase PIAS2
LOC115553778						0.51								
LOC115553803	-1.43	-0.77	-1.11	0.44										Circadian-associated transcriptional repressor
LOC115553809	-0.50		-0.42			-0.33							K26169	Membrane progestin receptor delta
LOC115553812				0.44										Interleukin 16
LOC115553827											Loss	Loss	K01330	Complement component 1, r subcomponent
LOC115553831						-0.53								Carcinoembryonic antigen-related cell adhesion molecule
LOC115553842							Gain							Protein S100-A1, toll-like receptor 2
LOC115553864						0.89								Tripartite motif-containing protein 16
LOC115553871	-0.48												K01771, K22190	1-phosphatidylinositol phosphodiesterase , membrane-spanning 4-domains subfamily A member 3/4/8/12/15/18
LOC115553878	-0.50													
LOC115553890												Loss		
LOC115553895						-0.53							K15378	Solute carrier family 45, member 1/2/4
LOC115553904	0.69													

LOC115553906				0.82		0.86								
LOC115553918						0.56							K11643	Chromodomain-helicase-DNA-binding protein 4
LOC115553921			-0.43											
LOC115553930						-0.39							K04718	Sphingosine kinase
LOC115553933	-0.38					-0.37							K09097	Aryl hydrocarbon receptor nuclear translocator
LOC115553936											Loss	Loss		
LOC115553939			-0.46			-0.49							K05627	BAI1-associated protein 2
LOC115553942	-0.94													Neuronal pentraxin
LOC115553954											Gain	Loss	K05007	Potassium inwardly-rectifying channel subfamily J member 14
LOC115553957						0.64						Loss		NADH dehydrogenase (ubiquinone) flavoprotein 1
LOC115553963	-0.84		-0.63			-0.53								Dual specificity protein kinase CLK2/3
LOC115553970						0.51								Very-long-chain ceramide synthase
LOC115553971	-0.67		-0.57	-0.42		-1.12							K14946	RNA binding protein fox-1
LOC115553976						0.58	Gain + Loss	Gain		Gain	Gain	Gain + Loss		
LOC115553989						-0.31							K04393, K07975	Cell division control protein 42, Rho family, other
LOC115554002						-0.92								Protein-lysine 6-oxidase
LOC115554003	-1.24		-0.71			-0.96							K03145	Transcription elongation factor S-II
LOC115554005												Loss	K20465	Oxysterol-binding protein-related protein 9/10/11
LOC115554011	0.81	0.51	0.51											Acyl-coenzyme A thioesterase THEM4
LOC115554014	-0.53					-0.70							K24048	MAGUK p55 subfamily member 2/6
LOC115554019						-1.01							K10388	Plectin
LOC115554021						-0.49								
LOC115554024	0.56												K14620	Riboflavin transporter 2
LOC115554031				-0.48		-0.90								
LOC115554032	-0.46		-0.45											Dysbindin
LOC115554040				-0.48		-0.90							K15013	Long-chain-fatty-acid--coa ligase ACSBG
LOC115554053	-1.41	-0.49	-0.86			-0.53							K04894	Potassium voltage-gated channel Isk-related subfamily E member 1
LOC115554057						-0.44								Alpha-1,3-mannosyl-glycoprotein beta-1,2-N-acetylglucosaminyltransferase
LOC115554058	-0.38					-0.36								SH3-domain binding protein 5
LOC115554063												Gain		
LOC115554078						-0.43							K20217	Ubiquitin-conjugating enzyme E2 E

LOC115554092		0.47	0.60										K08626	A disintegrin and metalloproteinase with thrombospondin motifs 12
LOC115554103	-0.68													
LOC115554105		0.37											K07968	Beta-1,4-galactosyltransferase 3
LOC115554106						-0.84								Extracellular matrix protein 1
LOC115554112									Gain			Loss	K06771	Myelin associated glycoprotein
LOC115554115			0.54										K03255	Protein TIF31
LOC115554123	-1.30										Loss			
LOC115554143				0.42		0.66								
LOC115554145						-0.40							K09403	Forkhead box protein J2/3
LOC115554177		-0.39	-0.45											ADP-ribosylation factor-like protein 4
LOC115554184						-0.52								Disintegrin and metalloproteinase domain-containing protein 28
LOC115554187				-0.38									K00020	3-hydroxyisobutyrate dehydrogenase
LOC115554205	-0.36													
LOC115554211	-0.53												K01158	Deoxyribonuclease II
LOC115554221						-0.56								
LOC115554227	0.51													Histidine triad (HIT) family protein
LOC115554233						-0.94							K08042	Adenylate cyclase 2
LOC115554242	0.58					-0.53								Chondroitin sulfate proteoglycan 4
LOC115554254	-0.79		-0.90										K08531	Nuclear receptor subfamily 1 group D member 2
LOC115554256							Loss							
LOC115554263	-1.39	-0.41	-0.83											GABA(A) receptor-associated protein
LOC115554274						-1.00						Gain		Carnitine O-octanoyltransferase
LOC115554275	-1.18	-0.43	-1.04										K05940	Carnitine O-octanoyltransferase
LOC115554283												Loss		
LOC115554286		0.54				-0.71								
LOC115554287	-0.32													
LOC115554291						-0.42								General transcription factor IIIA, KRAB domain-containing zinc finger protein
LOC115554305						0.69								CD22 antigen
LOC115554307				0.41		0.58						Loss		
LOC115554314				0.43		0.57							K04188	C-X-C chemokine receptor type 3
LOC115554330						-0.55							K20688	Rho guanine nucleotide exchange factor 16
LOC115554338	0.34		0.36											GON-4-like protein

LOC115554341						-0.59								Ras gtpase-activating-like protein IQGAP2/3
LOC115554344	0.44		0.49											
LOC115554352						0.86								SH2 domain-containing adapter protein B/D/E/F
LOC115554353				-0.37		-0.58							K09228	KRAB domain-containing zinc finger protein
LOC115554354	0.31													
LOC115554356				0.43										Human immunodeficiency virus type I enhancer-binding protein
LOC115554357						-0.75								KRAB domain-containing zinc finger protein
LOC115554362	0.31		0.30											
LOC115554369			-0.45											
LOC115554371						-0.52								
LOC115554374				-0.50		-0.71								
LOC115554375						-0.68								
LOC115554383												Loss	K16367	Endothelin-2
LOC115554397						-0.31							K22376	E3 ubiquitin-protein ligase KCMF1
LOC115554408												Gain		
LOC115554440							Loss							
LOC115554451				-0.34		-0.52								
LOC115554462												Loss		
LOC115554467	-0.50													
LOC115554477						0.77								
LOC115554478							Gain		Gain					
LOC115554483	-0.52		-0.42											
LOC115554506											Gain		K10955	Mucin-2
LOC115554507						0.45								Collagen type V/XI/XXIV/XXVII, alpha
LOC115554508	0.51		0.72											Tenascin
LOC115554509				-0.42		-0.58								Scavenger receptor class F member 2
LOC115554519						-0.76					Loss			Htra serine peptidase 1
LOC115554523						0.65								Lectin, mannose-binding 2-like
LOC115554525						-0.72								
LOC115554526	0.64			0.44										
LOC115554528				0.53		0.57							K06489	CD53 antigen
LOC115554553								Gain	Gain	Gain		Gain		
LOC115554568						-0.77								

LOC115554584						-0.45							K09454	GA-binding protein transcription factor, beta
LOC115554586	-0.60													
LOC115554609				0.37		1.06								
LOC115554610			-0.66											
LOC115554626						1.04								
LOC115554641			-0.41										K02539	Myeloid leukemia cell differntiation protein MCL-1
LOC115554645												Loss		
LOC115554649	0.51		0.41			-0.96								Zinc finger protein 687
LOC115554651						0.32								26S proteasome regulatory subunit N10
LOC115554695	-0.96												K08858	Serine/threonine-protein kinase SBK
LOC115554699			-0.41										K18623	Protein cordon-bleu
LOC115554700							Gain	Gain	Gain			Loss		
LOC115554702												Loss		
LOC115554703						-0.80								POU domain transcription factor, class 3, POU domain transcription factor, class 5
LOC115554728				0.38										NLR family CARD domain-containing protein 3
LOC115554737	0.30	0.28	0.33										K13201	Nucleolysin TIA-1/TIAR
LOC115554750						-0.37							K08514	Vesicle-associated membrane protein 5
LOC115554754						0.63		Loss						
LOC115554769						-1.01								
LOC115554772	-1.18	-0.80	-1.23			-0.60							K22382	WD repeat-containing protein 26
LOC115554790												Loss		
LOC115554976							Gain							
LOC115554990	0.54		0.53											Forkhead box protein Q
LOC115554999			-0.57	0.41		0.68							K16449	Regulator of G-protein signaling
LOC115555001				0.48		0.60								
LOC115555008	0.30													Peptidyl-prolyl isomerase H (cyclophilin H)
LOC115555063												Gain		
LOC115555076						-0.85								
LOC115555079											Gain	Gain		Tetraspanin-1
LOC115555083				0.54									K10356	Myosin I
LOC115555094						-0.66								
LOC115555102	-0.67		-0.78										K19030	6-phosphofructo-2-kinase / fructose-2,6-biphosphatase 4

LOC115555116	0.69		0.41										K08057	Calreticulin
LOC115555143				0.64		1.49								NLR family CARD domain-containing protein 3
LOC115555152	-0.60					-0.58								G2/M phase-specific E3 ubiquitin-protein ligase
LOC115555155							Loss		Loss		Gain	Gain		
LOC115555167						0.66							K01411	Nardilysin
LOC115555178						-0.37					Loss			
LOC115555196				-0.38		-1.04								
LOC115555199				-0.14										
LOC115555203	-0.36		-0.32											Ras-related protein Rab-11A, Ras-related protein Rab-11B
LOC115555205				-0.48		-0.98								Cartilage intermediate layer protein
LOC115555211						0.51								
LOC115555227	-0.52	-0.68												
LOC115555228						-0.68								AT-rich interactive domain-containing protein 3
LOC115555229							Gain							
LOC115555230	0.90												K08767	Angiopoietin-like 4
LOC115555232			0.54										K17616	CTD small phosphatase-like protein 2
LOC115555241											Loss			
LOC115555250				0.50									K21124	Adaptor protein CIKS
LOC115555255			-0.38											Ubiquitin carboxyl-terminal hydrolase CYLD
LOC115555260				0.40			Loss						K06092	Inad-like protein
LOC115555266	-0.89	-0.35	-1.16			-0.48								Ubiquitin-protein ligase E3 A
LOC115555277											Gain			DNA replication licensing factor MCM3
LOC115555279	-0.95	-0.29	-0.57			-0.36							K13293	Camp-specific phosphodiesterase 4
LOC115555285						-1.19							K23413	Anosmin-1
LOC115555286						0.78								
LOC115555287								Loss	Loss				K08606	Meprin B
LOC115555296							Loss	Loss			Gain		K08385	G0-opsin
LOC115555317						0.61							K08594	Sentrin-specific protease 5
LOC115555341			0.89								Loss	Loss		Camp-specific phosphodiesterase 4
LOC115555350				-0.78		-1.19							K12394	AP-1 complex subunit sigma 1/2
LOC115555353									Gain					Retinoschisin
LOC115555362	0.84		0.47											
LOC115555402	0.63													Obscurin-rhogef

LOC115555406				0.58									K05063	Interleukin 12 receptor beta-1
LOC115555410				0.49		0.73								
LOC115555430						-0.81								Uridine kinase
LOC115555434	0.36													Importin-13
LOC115555440						0.92								
LOC115555445	-0.31													Ras-related protein Rab-11A, Ras-related protein Rab-11B
LOC115555447						-0.89								Myomegalin
LOC115555457				0.47										
LOC115555464			0.59											
LOC115555472						0.57								
LOC115555478												Gain		Selectin, platelet
LOC115555504						0.68								
LOC115555505						-0.46								
LOC115555508							Loss				Loss	Loss		Receptor expression-enhancing protein 5/6
LOC115555526						0.60								
LOC115555527						-0.37								
LOC115555536						0.57								
LOC115555549											Loss			Interleukin 12 receptor beta-2
LOC115555565						-0.59							K06736	Cadherin 2, type 1, N-cadherin
LOC115555566						0.62							K19502	Anoctamin-8
LOC115555570			-0.38			-0.91							K10462	Kelch-like protein 25/37 (ectoderm-neural cortex protein)
LOC115555582						0.47								
LOC115555583						-0.51							K08526	Retinoid X receptor gamma
LOC115555592	0.29	0.22	0.29											Scaffold attachment factor B
LOC115555600	-0.44		-0.41											
LOC115555603			-0.50											Dimethylaniline monooxygenase (N-oxide forming) / hypotaurine monooxygenase
LOC115555608							Loss	Loss			Loss	Loss		
LOC115555610											Loss	Loss		
LOC115555623						0.74							K00485	Dimethylaniline monooxygenase (N-oxide forming) / hypotaurine monooxygenase
LOC115555625												Loss		NLR family CARD domain-containing protein 3
LOC115555627						-0.50							K11971	E3 ubiquitin-protein ligase RNF14
LOC115555633						0.38								

LOC115555650						0.59							K16513	Carboxyl-terminal PDZ ligand of neuronal nitric oxide synthase protein
LOC115555652			-0.49	0.43		0.87							K04527	Insulin receptor
LOC115555654	0.54												K13373	17beta-estradiol 17-dehydrogenase / 3beta-hydroxysteroid 3-dehydrogenase
LOC115555655			0.41											COPII coat assembly protein SEC16
LOC115555657						-0.69							K09191	General transcription factor IIIA
LOC115555658						0.38								Growth arrest and DNA-damage-inducible protein
LOC115555662						0.62								Fragile X mental retardation protein
LOC115555665						-0.53								Protein phosphatase 1H
LOC115555674												Loss		
LOC115555675	-0.60	-0.64	-1.02										K17594	Phosphatase and actin regulator
LOC115555691						-0.43					Loss	Loss		
LOC115555715						-0.42								Kinesin-associated protein 3
LOC115555718						0.69								Adhesion G-protein coupled receptor D2
LOC115555723						-0.52							K00485	Dimethylaniline monooxygenase (N-oxide forming) / hypotaurine monooxygenase
LOC115555727						0.58								Kinase insert domain protein receptor
LOC115555734				0.38		1.36								Kinase insert domain protein receptor
LOC115555738						-0.52							K18080	Tensin
LOC115555744						0.63							K16833	Protein phosphatase inhibitor 2
LOC115555748	0.46		0.52											
LOC115555750						0.50							K24190	Lens fiber membrane intrinsic protein
LOC115555754						0.73								F-BAR domain only protein
LOC115555777						-0.86								
LOC115555795						-0.30								
LOC115555797											Gain	Gain		
LOC115555800	0.43												K13203	Zinc finger RNA-binding protein
LOC115555806	-0.52					-0.79							K15010	Homer
LOC115555823	-1.44													
LOC115555824						-0.97								
LOC115555825	-0.44												K00907	Myosin-light-chain kinase
LOC115555834	0.56	0.37												SH2 domain-containing adapter protein B/D/E/F
LOC115555837	0.44												K05759	Profilin

LOC115555845								Loss						Phosphatidate phosphatase
LOC115555853	0.44		0.62											
LOC115555854						-0.41								3-methylcrotonyl-coa carboxylase beta subunit
LOC115555882						-1.47							K23949	Sterile alpha motif domain-contaning protein 9
LOC115555890			-0.45										K04389	Tumor necrosis factor ligand superfamily member 6
LOC115555892	-0.67		-0.49											
LOC115555897	-0.35		-0.30										K07820	Beta-1,3-galactosyltransferase 2
LOC115555903							Gain		Loss			Loss		
LOC115555919				-0.60		-1.16								Lipoprotein lipase
LOC115555942						0.66							K06778	Receptor-type tyrosine-protein phosphatase S
LOC115555951		0.88											K05064	Interleukin 12 receptor beta-2
LOC115555953			-0.58											
LOC115555962						0.64								
LOC115555973						0.74								
LOC115555977										Gain	Gain	Gain	K07848	Rad and Gem related GTP binding protein 2
LOC115555978			0.42	0.36		0.50								
LOC115556003			-0.43			-0.60							K10839	UV excision repair protein RAD23
LOC115556012						-0.50								
LOC115556016						-0.62							K20685	Guanine nucleotide exchange factor MCF2
LOC115556033	0.62													Ribonuclease P protein subunit RPR2
LOC115556039	-0.97													
LOC115556045						-1.05								
LOC115556046			0.53											Glutamine synthetase
LOC115556057						0.73								
LOC115556059										Loss		Loss	K05869	Calcium/calmodulin-dependent protein kinase IV
LOC115556089						-0.73							K04587	Pituitary adenylate cyclase-activating polypeptide type I receptor
LOC115556104				0.48										Janus kinase 3
LOC115556105	0.49													
LOC115556106						0.62								Potassium channel subfamily V member 2
LOC115556109						-0.95								
LOC115556117				-0.50		-1.29							K01691	WNT inhibitory factor 1
LOC115556140						-0.47	Loss						K23096	Repulsive guidance molecule A
LOC115556154				0.48			Gain	Gain					K06097	Tight junction protein 3

LOC115556174	0.29	0.36	0.29											Microtubule-associated protein 1
LOC115556181						1.08								
LOC115556190	0.70					-0.63							K23578	Insulin-like growth factor-binding protein 1
LOC115556201	-0.65		-0.47			-0.36								26S proteasome regulatory subunit N13, oxysterol-binding protein-related protein 1/2
LOC115556210	-0.66	-0.53	-0.90											Occludin
LOC115556222	0.69													
LOC115556229	0.35		0.34											Groucho
LOC115556234						0.92								
LOC115556240	0.31		0.38										K08592	Sentrin-specific protease 1
LOC115556254				0.44										ABC-2 type transport system ATP-binding protein, ATP-binding cassette, subfamily A (ABC1), member 1
LOC115556269			-0.46			-0.49						Loss	K22029	Palladin
LOC115556276				-0.41		-0.85							K25386	BMP/retinoic acid-inducible neural-specific protein 2/3
LOC115556277						-0.50							K16449	Regulator of G-protein signaling
LOC115556281						-0.51								Caytaxin
LOC115556289						-0.30							K01076	Abhydrolase domain-containing protein 17
LOC115556290	-0.22													AN1-type zinc finger protein 5/6
LOC115556303	-0.62		-0.73			-1.01							K09445	Interferon regulatory factor 4
LOC115556308							Gain		Gain		Loss	Loss	K24637, K24640	Zinc finger BED domain-containing protein 1 (E3 SUMO-protein ligase ZBED1) , zinc finger BED domain-containing protein 4
LOC115556312			0.26											Methyl-cpg-binding domain protein 2, methyl-cpg-binding domain protein 3
LOC115556328	0.41					0.35							K25449	Olfactomedin-like protein 2
LOC115556337						0.94								Interleukin 4 receptor
LOC115556347								Loss	Loss		Gain	Gain		Alkaline phosphatase
LOC115556349				-0.44		-0.86								Notch 2
LOC115556351						-0.54							K16340	PH domain and leucine-rich repeat-containing protein phosphatase
LOC115556358						-0.90								Protein N-terminal monomethyltransferase
LOC115556361			-0.70											
LOC115556429						0.58								
LOC115556441						0.79								
LOC115556521			0.32											Coronin-1B/1C/6
LOC115556525	0.52													

LOC115556538	-0.41		-0.36										K07897	Ras-related protein Rab-7A
LOC115556551						-0.71							K07189	Protein phosphatase 1 regulatory subunit 3A/B/C/D/E
LOC115556554							Loss							
LOC115556558	0.52		0.41											ATP-dependent RNA helicase DDX19/DBP5
LOC115556561						-0.82	Loss							Arf-GAP with coiled-coil, ANK repeat and PH domain-containing protein
LOC115556568	-0.50		-0.63										K09105	Transcription factor E3
LOC115556571						-0.48								Novel protein kinase C delta type
LOC115556574	-0.55													
LOC115556576										Gain	Gain	Loss		
LOC115556579	-0.36			0.70		0.57							K11408	Histone deacetylase 7
LOC115556580						-0.90								
LOC115556592						0.36								
LOC115556595				0.42										NCK-associated protein 1
LOC115556597						-0.66								Protein phosphatase 1 regulatory subunit 1A
LOC115556598				-0.39		-0.49								DDB1- and CUL4-associated factor 1
LOC115556609				0.49										
LOC115556613	-0.85	-0.41	-0.60			-0.66								
LOC115556617						-0.56							K18266	Protein NDRG1
LOC115556626						-0.88								
LOC115556627						0.62							K11885	DNA damage-inducible protein 1
LOC115556628	-1.55													
LOC115556630				-0.44										
LOC115556633				-0.68										
LOC115556634						0.57								
LOC115556637											Loss			
LOC115556638						1.37								
LOC115556650				1.61										Putative transposase
LOC115556664							Gain					Gain		
LOC115556665							Loss							
LOC115556683						0.82								Aquaporin-7
LOC115556685	-0.69	-0.53	-0.60			-0.65							K04958	Inositol 1,4,5-triphosphate receptor type 1
LOC115556687	-0.44		-0.47											Syndecan 4

LOC115556690				-0.40		-0.96							K22591	Activity-dependent neuroprotector homeobox protein
LOC115556693				-0.47		-0.62								
LOC115556694						0.39							K04570	Bcl-2-like 1 (apoptosis regulator Bcl-X)
LOC115556696				0.45									K01490	AMP deaminase
LOC115556699	-1.82	-0.81	-1.86										K08539	Vitamin D3 receptor
LOC115556704	-0.64													Zinc fingers and homeoboxes protein 3
LOC115556722			0.42										K14004	Protein transport protein SEC13
LOC115556723	0.52												K14004	Protein transport protein SEC13
LOC115556736	0.41	0.39	0.43											
LOC115556743						-0.65							K22583	Metalloproteinase inhibitor 2
LOC115556746	0.51													SWI/SNF-related matrix-associated actin-dependent regulator of chromatin subfamily C
LOC115556747							Loss							Poly(U)-specific endoribonuclease
LOC115556758	0.52	0.55					Gain		Gain		Loss			
LOC115556771	-0.66	-0.32	-0.60											E3 ubiquitin-protein ligase Itchy
LOC115556778				0.48									K07526	SLIT-ROBO Rho gtpase activating protein
LOC115556780	-0.55												K01831	Prostacyclin synthase
LOC115556788	0.41		0.29										K14966	Host cell factor
LOC115556802						0.76							K06840	Semaphorin 3
LOC115556804	-0.54													
LOC115556807						-0.41								Copine 1/2/3, RNA-binding protein 12
LOC115556808	-0.51					-0.69							K04441	P38 MAP kinase
LOC115556813						-0.63							K25812	Protein APCDD1
LOC115556818	-0.83	-0.55												Deoxyribonuclease-1-like protein
LOC115556822				0.50		1.30								
LOC115556826				-0.42		-0.40								
LOC115556834	0.37													
LOC115556839	-1.61	-1.20	-1.15											Noggin
LOC115556852	0.46													
LOC115556856							Loss	Loss	Loss				K24513	Inter-alpha-trypsin inhibitor heavy chain H3
LOC115556857							Loss						K24513	Inter-alpha-trypsin inhibitor heavy chain H3
LOC115556866						0.62							K07605	Type II keratin, basic
LOC115556867									Loss					

LOC115556877	0.59													IQ motif and SEC7 domain-containing protein
LOC115556887								Gain				Loss		
LOC115556889	0.66												K04265	Adenosine receptor A1
LOC115556894							Loss	Loss						
LOC115556895						0.67								
LOC115556898			0.37										K11757	Protein polybromo-1
LOC115556905							Gain	Gain	Gain					
LOC115556919									Gain				K21444	Poly(rc)-binding protein 3/4
LOC115556924	0.40		0.57											Rac gtpase-activating protein 1
LOC115556936							Loss	Gain				Gain + Loss		
LOC115556940												Loss		
LOC115556942						0.43							K04550	Low-density lipoprotein receptor-related protein 1 (alpha-2-macroglobulin receptor)
LOC115556947						-0.51								
LOC115556953			0.45									Loss	K09536	Dnaj homolog subfamily C member 16
LOC115556964	0.71													Voltage-dependent calcium channel T type alpha-1I
LOC115556973	-0.54	-0.49	-0.56											Paxillin
LOC115556974		0.30											K17558	Protein phosphatase 1 regulatory subunit 15B
LOC115556978			0.34											Citron Rho-interacting kinase
LOC115556979							Gain						K11701	Helicase required for rnai-mediated heterochromatin assembly 1
LOC115556984						1.25								NLR family CARD domain-containing protein 3
LOC115557001						-0.51								
LOC115557002				-0.42		-1.16								Solute carrier family 38 (sodium-coupled neutral amino acid transporter), member 3
LOC115557017	-0.76		-1.14											
LOC115557021				-0.43		-1.10								Transmembrane reductase
LOC115557025	-2.19	-2.02	-2.70											6-phosphofructo-2-kinase / fructose-2,6-biphosphatase 4
LOC115557039							Gain							Caveolin 2
LOC115557051			-0.45											
LOC115557055							Loss						K23539	Protein unc-119
LOC115557057	-0.37		-0.42								Gain	Gain + Loss	K08826	Homeodomain interacting protein kinase
LOC115557060							Gain							
LOC115557086	-0.70		-1.27										K25556	Guanylate cyclase activator 2

LOC115557088						-0.30								RNA-binding protein 24/38
LOC115557093						1.04								
LOC115557094						0.51						Gain		Retinoic acid receptor gamma
LOC115557099	0.37												K11432	
LOC115557112	0.64	0.34		0.43									K03462	Nicotinamide phosphoribosyltransferase
LOC115557129						-0.72							K15008	Discs, large-associated protein 1
LOC115557131				0.37									K15728	Phosphatidate phosphatase LPIN
LOC115557132						-0.53								Homeobox protein TGIF2
LOC115557136			-0.45										K12755, K12757	Myosin regulatory light chain 12, myosin regulatory light chain 9
LOC115557155	0.41		0.26											Poly(rc)-binding protein 2, poly(rc)-binding protein 3/4
LOC115557162											Loss		K06840	Semaphorin 3
LOC115557165						-0.60							K03129	Transcription initiation factor TFIID subunit 4
LOC115557168											Loss	Loss	K20189	Dysbindin
LOC115557174							Loss		Loss				K23222	Nuclease HARBI1
LOC115557188				0.42		0.35								
LOC115557190							Loss							Wingless-type MMTV integration site family, member 2
LOC115557199												Loss		
LOC115557202		-0.30	-0.35										K03871	Von Hippel-Lindau disease tumor supressor
LOC115557206											Loss			
LOC115557210				-0.39		-0.43							K01251	Adenosylhomocysteinase
LOC115557212	1.85													Cyclin-dependent kinase inhibitor 1A
LOC115557213	0.50		0.47											Serine/arginine-rich splicing factor 3, serine/arginine-rich splicing factor 7
LOC115557214				0.40										
LOC115557222	-0.98		-0.62					Gain			Loss	Loss		
LOC115557226						-0.67								Inositol-hexakisphosphate 5-kinase
LOC115557227	-0.53													Receptor-type tyrosine-protein phosphatase gamma
LOC115557229			0.40											
LOC115557234								Loss				Gain		
LOC115557239											Gain			Vomeronasal 1 receptor
LOC115557240	-0.32		-0.29											ADP-ribosylation factor-like protein 8
LOC115557252							Gain	Gain	Gain + Loss		Gain + Loss	Loss	K18763	La-related protein 4

LOC115557253						0.55								Activating transcription factor 7
LOC115557254											Gain	Gain		Activating transcription factor 1
LOC115557257						0.57								
LOC115557262			0.55											
LOC115557263	-0.60		-0.49											Next to BRCA1 gene 1 protein
LOC115557274	-0.42												K05100	Macrophage-stimulating 1 receptor
LOC115557283	-0.52													Transglutaminase 2
LOC115557284						-0.57								Mitogen-activated protein kinase kinase kinase 12
LOC115557294						-0.44								
LOC115557304				2.52		0.93								
LOC115557306							Gain	Gain			Loss	Loss		Advillin
LOC115557317	-0.35					-0.41							K23860	Pleckstrin homology domain-containing family G member 4
LOC115557326								Loss	Loss				K20838	N-acetyltransferase 8
LOC115557332						-0.74							K16667	Receptor-type tyrosine-protein phosphatase gamma
LOC115557346	-0.45													
LOC115557347	-0.68													
LOC115557348						0.68							K23770	Protein S100-B
LOC115557350						-1.10								Nuclear receptor subfamily 1 group D member 1
LOC115557357						-1.18								
LOC115557370	-0.69	-0.52												
LOC115557378						-0.84								
LOC115557379						-0.69								Sortilin
LOC115557382						0.72								Matrix remodeling-associated protein 8
LOC115557383							Gain	Gain	Gain					
LOC115557391				-0.44		-0.73								
LOC115557395						0.63		Gain		Loss	Loss	Loss	K01197	Hyaluronoglucosaminidase
LOC115557398	0.56					-0.63								Myogenic factor 4 (myogenin)
LOC115557401	0.37	0.40	0.44											Methyl-cpg-binding domain protein 1
LOC115557412	0.46													Peptidyl-trna hydrolase, PTH2 family
LOC115557417				0.53									K05084	Receptor tyrosine-protein kinase erbb-3
LOC115557423										Loss	Loss	Loss	K01197	Hyaluronoglucosaminidase
LOC115557424						0.55							K25100	Msx2-interacting protein
LOC115557428	1.06	0.47	0.72										K17681	Atpase family AAA domain-containing protein 3A/B

LOC115557445						-0.69							K04739	Camp-dependent protein kinase regulator
LOC115557466	-0.44		-0.47											
LOC115557468	0.36		0.34											Kinetochore-associated protein DSN1
LOC115557474						-1.02								Solute carrier family 6 (neurotransmitter transporter, GABA) member 1
LOC115557478							Loss	Loss						
LOC115557483						0.57								Cell death activator CIDE-C
LOC115557486	0.51	0.43	0.53	-0.36										
LOC115557490	0.47		0.73											Aurora kinase A
LOC115557496						-0.73							K19749	Retinoic acid-induced protein 1
LOC115557498						-0.68						Gain		
LOC115557504						0.39								Cytochrome c oxidase assembly factor 14
LOC115557518	-0.93													Purine-nucleoside phosphorylase
LOC115557545						0.64							K04927	Potassium voltage-gated channel KQT-like subfamily member 2
LOC115557548						0.61							K17920	Sorting nexin-5/6/32
LOC115557550						0.49								
LOC115557558	-0.46		-0.55											
LOC115557569						0.47							K20690	Rho guanine nucleotide exchange factor 25
LOC115557571		-0.39	-0.45						Loss		Gain		K13567	Activin receptor type-1B
LOC115557585	0.50													Pre-mrna-splicing factor ISY1
LOC115557588	-0.63													
LOC115557590											Loss		K09442	SAM pointed domain-containing ETS transcription factor
LOC115557594				0.39		0.72							K07766	Diphosphoinositol-polyphosphate diphosphatase
LOC115557596				0.47									K04607	Metabotropic glutamate receptor 4
LOC115557598	-0.74		-2.13			0.68								
LOC115557604	0.83			0.42										
LOC115557624			-0.46			-0.97								
LOC115557629	-0.64		-0.68											
LOC115557631				0.49		0.57							K05166	Interleukin 17 receptor C
LOC115557633	-1.15					-0.82								
LOC115557636											Loss			1-phosphatidylinositol-5-phosphate 4-kinase
LOC115557637	-0.25													Ran-binding protein 9/10
LOC115557638	0.63		0.47											Cyclin-dependent kinase 4

LOC115557640	-0.52												K23732	Coiled-coil domain-containing protein 63/114
LOC115557642								Loss			Loss	Gain	K23335	Glucose-induced degradation protein 4
LOC115557662	-1.38	-1.25	-1.65											
LOC115557678	0.45												K22936	Protein canopy 1/2
LOC115557682						-1.05								
LOC115557687		0.38											K00588	Caffeoyl-coa O-methyltransferase
LOC115557688						-0.49								2-epi-5-epi-valiolone synthase
LOC115557693						1.22								
LOC115557695				-0.32		-0.39							K09191, K09228	General transcription factor IIIA, KRAB domain-containing zinc finger protein
LOC115557700	-4.41	-2.20	-4.23									Gain + Loss		
LOC115557711						-0.78								Tensin
LOC115557714						0.55								Melanocyte protein PMEL
LOC115557716							Loss						K06761	Contactin 3
LOC115557717						-0.52								Serine/threonine-protein phosphatase 6 regulatory ankyrin repeat subunit C
LOC115557739						-1.07							K17620	Eyes absent homolog 2
LOC115557745				-0.51		-0.93								Extracellular sulfatase Sulf , N-acetylglucosamine-6-sulfatase
LOC115557753		0.42				0.66					Loss			CD34 antigen
LOC115557762									Gain			Loss	K12958	Caveolin 2
LOC115557765							Loss		Loss			Loss		
LOC115557767				-0.49		-0.68								Scotin
LOC115557774						-0.72							K22608	Parathyroid hormone-related hormone
LOC115557775						-0.52								P-type Ca2+ transporter type 2B
LOC115557778				0.77		0.61								Protein TASOR
LOC115557781		-0.65												
LOC115557786	-0.73		-1.06											Class B basic helix-loop-helix protein 2
LOC115557796						0.86								NLR family CARD domain-containing protein 3
LOC115557797				0.42										NLR family CARD domain-containing protein 3
LOC115557802						0.48								Prosaposin receptor
LOC115557817						-0.30								Ubiquitin carboxyl-terminal hydrolase 4/11
LOC115557818						0.65								
LOC115557823						-0.64							K11350, K11380	Bromodomain and PHD finger-containing protein 3, nua3 HAT complex component NTO1

LOC115557836				-0.43		-0.74								Solute carrier family 32 (vesicular inhibitory amino acid transporter)
LOC115557845						0.64								
LOC115557849						-0.74								Protein kinase C-binding protein 1
LOC115557853				-3.13										
LOC115557860				-0.43		-0.87							K08121	Fibromodulin
LOC115557864	-1.67	-1.15	-1.65											
LOC115557895												Loss		
LOC115557898						0.72								
LOC115558028						-0.56							K11307	Histone acetyltransferase MYST2
LOC115558054						-0.65								
LOC115558091											Loss	Gain		
LOC115558176												Gain	K25856	AT-rich interactive domain-containing protein 3
LOC115558179						-0.59								
LOC115558195						-0.60							K04147	Dopamine receptor D4
LOC115558199							Loss				Loss	Gain + Loss		
LOC115558204						0.62								Aromatase
LOC115558210						-0.55							K09395	Forkhead box protein B
LOC115558212				0.58									K20045	Intersectin
LOC115558213				-0.35		-0.46								GC-rich sequence DNA-binding factor
LOC115558214	-0.54	-0.68	-0.49											
LOC115558216									Gain				K24636	Stereocilin
LOC115558222	0.98													
LOC115558241	0.46		0.40										K11824	AP-2 complex subunit alpha
LOC115558247						0.71								Isocitrate dehydrogenase
LOC115558248		0.34				0.36								Isocitrate dehydrogenase
LOC115558260	-0.36		-0.56	-0.45		-0.60							K09269	Transcription factor SOX5/6/13 (SOX group D)
LOC115558261						1.19							K01025	Sulfotransferase
LOC115558264						0.77								
LOC115558269		-0.40	-0.61											
LOC115558272						-1.02							K07127	5-hydroxyisourate hydrolase
LOC115558275							Loss		Loss			Gain		
LOC115558277	-0.40												K01363	Cathepsin B

LOC115558292									Loss					
LOC115558301	-0.33	-0.32		0.39		0.32								Cathepsin A (carboxypeptidase C)
LOC115558304			0.30											Transcription factor E2F4/5
LOC115558311						-0.64								
LOC115558318				-0.44		-0.96						Loss		
LOC115558321				-0.41		-0.69							K08622	A disintegrin and metalloproteinase with thrombospondin motifs 7
LOC115558322				-1.34		-0.63					Gain	Gain	K01025	Sulfotransferase
LOC115558325						-1.07							K19931	Protein lin-7
LOC115558352				-0.37		-0.62							K09191, K09228	General transcription factor IIIA, KRAB domain-containing zinc finger protein
LOC115558358			-0.41											Metastasis suppressor protein 1
LOC115558360				0.39		0.48							K05701	Tight junction protein 1
LOC115558364						-0.68								
LOC115558366			-0.40			-0.50								Ras-related protein Rab-8A, Ras-related protein Rab-8B
LOC115558367						-0.40								
LOC115558382				0.41		0.57							K10360	Myosin IX
LOC115558386						1.09								
LOC115558389						0.43								26S proteasome regulatory subunit T5
LOC115558395	-0.36												K07865	Ras homolog gene family, member U
LOC115558410						-0.57								
LOC115558415						0.67								Neuroplastin
LOC115558417	-0.31		-0.34	0.29									K10582	Ubiquitin-conjugating enzyme E2 Q
LOC115558429												Gain		
LOC115558437	-0.71		-0.49			-0.65							K17822	DCN1-like protein 1/2
LOC115558446												Gain	K04913	Potassium channel subfamily K member 2
LOC115558451				-0.42		-0.54							K19480	Anoctamin-5
LOC115558463						-0.33							K02146	V-type H+-transporting atpase subunit d
LOC115558467						0.57								Rho family-interacting cell polarization regulator
LOC115558475				0.37									K06559	Lymphocyte antigen 75
LOC115558476	-0.53		-0.45										K16529	A-kinase anchor protein 13
LOC115558477											Loss		K24144	Nance-Horan syndrome protein and NHS-like proteins
LOC115558480			-0.36										K05087	Insulin-like growth factor 1 receptor
LOC115558482						-0.54								Aminopeptidase N

LOC115558483	-0.59	-0.46	-0.51											Aminopeptidase N
LOC115558484	-1.94	-0.44	-2.03			0.61							K15728	Phosphatidate phosphatase LPIN
LOC115558499	0.83	0.47	0.53			0.73								
LOC115558505			-0.50					Loss			Gain + Loss			
LOC115558510						0.60								Solute carrier family 7 (D/L-type amino acid transporter), member 10
LOC115558516						-0.61								Homeobox protein HB9
LOC115558519						0.81							K14361	Organic solute transporter subunit beta
LOC115558538	-0.46	-0.42	-0.64											
LOC115558549				0.42		0.54							K25782	Immunoglobulin-like domain-containing receptor 2
LOC115558554											Gain	Gain		
LOC115558562			-0.44			-0.52								Tripartite motif-containing protein 66
LOC115558564						0.67							K21414	Ankyrin repeat and SAM domain-containing protein 4B
LOC115558577	-2.49													Protein N-lysine methyltransferase METTL21C
LOC115558578	0.46		0.44										K13354	Solute carrier family 25 (peroxisomal adenine nucleotide transporter), member 17
LOC115558587	1.04	0.54	0.57											Ubiquitin carboxyl-terminal hydrolase 10
LOC115558592											Loss	Loss		Growth arrest-specific 1
LOC115558596			-0.41										K13024	Inositol-hexakisphosphate/diphosphoinositol-pentakisphosphate 1-kinase
LOC115558607						-0.42								Carnitine O-palmitoyltransferase 1, liver isoform
LOC115558616	-0.63					0.70					Gain			
LOC115558634	-0.47												K17988	Tectonin beta-propeller repeat-containing protein 1
LOC115558644			0.60											Membrane dipeptidase
LOC115558646			-0.56					Loss	Loss					Solute carrier family 27 (fatty acid transporter), member 2
LOC115558654						0.61								
LOC115558656	0.49		0.59										K21770	G2/mitotic-specific cyclin-B2
LOC115558665												Gain	K04903	Potassium voltage-gated channel subfamily G member 4
LOC115558668						-0.56								
LOC115558691						-0.44								
LOC115558700	-0.47		-0.45			-0.57							K17335	Nuclear factor of activated T-cells 5
LOC115558703			-0.34											RNA polymerase II elongation factor ELL
LOC115558705	-1.65		-0.99			-0.58	Loss	Loss					K08532	RAR-related orphan receptor alpha
LOC115558735	-0.95										Gain			Protein N-lysine methyltransferase METTL21C

LOC115558757	0.68			-0.55									K17653	Dimethyladenosine transferase 2, mitochondrial
LOC115558759	0.47		0.69											Cytoskeleton-associated protein 5
LOC115558782						-0.53								Protein phosphatase 1 regulatory subunit 3A/B/C/D/E
LOC115558806											Gain			Cocaine- and amphetamine-regulated transcript protein
LOC115558807						0.52								Kinesin family member 13
LOC115558815						-0.44							K15293	Protein unc-13 A/B/C
LOC115558818						-0.61								Cytochrome P450 family 1 subfamily A
LOC115558826						-0.50								Bromodomain-containing protein 4
LOC115558833						-1.10								
LOC115558839						-0.53								Relaxin family peptide receptor 3
LOC115558843						-0.74							K17653	Dimethyladenosine transferase 2, mitochondrial
LOC115558849						0.49							K07374	Tubulin alpha
LOC115558851				-0.46		-0.84								Lysyl oxidase-like protein 1
LOC115558864	0.69			-0.42		-0.56								Isocitrate dehydrogenase (NAD+)
LOC115558867						0.61								Protein PET100, animal type
LOC115558873						-0.34							K19347	SUN domain-containing protein 1/2
LOC115558877				-0.41		-0.64								SIN3-HDAC complex-associated factor
LOC115558879							Loss	Gain	Gain			Gain	K10404	Kinesin family member 26
LOC115558883											Gain	Gain		Tripartite motif-containing protein 35
LOC115558885	0.54	0.35	0.37			-0.37								Protein ELYS
LOC115558894	-1.33		-0.61			0.60								Uncharacterized protein
LOC115558897	-3.23	-0.55	-3.29											
LOC115558898	-0.91		-0.92										K23168	Protein MPAN
LOC115558944						0.71								Tripartite motif-containing protein 16
LOC115558945			0.51									Gain		Collagen and calcium-binding EGF domain-containing protein 1
LOC115558949				0.45										Thyroid hormone receptor alpha
LOC115558951			0.50											
LOC115558968	-1.39		-1.08											GABA(A) receptor-associated protein
LOC115558977						0.97								
LOC115558981	-0.39													Mothers against decapentaplegic homolog 6
LOC115558982	-0.35													Pleckstrin homology domain-containing family G member 4
LOC115558988						0.44							K22594	Pogo transposable element with ZNF domain
LOC115558993						0.64					Loss	Loss		Transcription initiation factor TFIID subunit 4

LOC115558995				-0.27		-0.28							K18749	Protein LSM14
LOC115558997							Loss	Gain + Loss	Loss					
LOC115559000						-0.43							K15121	Solute carrier family 25, member 44
LOC115559001						-0.44								CD82 antigen
LOC115559002	-0.61													
LOC115559006				-0.99		-1.01				Loss				All-trans-retinol 3,4-desaturase
LOC115559011				-0.44										
LOC115559037	0.47					0.52								
LOC115559050						-0.62								Integrin beta 3
LOC115559057		-0.48												
LOC115559064	0.51													Lamin B
LOC115559079						-0.76						Gain	K04373	Ribosomal protein S6 kinase alpha-1/2/3/6
LOC115559081	-0.51		-0.40			-0.56					Gain		K16938	Septin 3/9/12
LOC115559084									Loss					
LOC115559085						0.44						Gain	K20163	DENN domain-containing protein 4
LOC115559088							Loss						K05152	Tumor necrosis factor receptor superfamily member 14
LOC115559089						-0.61							K10703	Very-long-chain (3R)-3-hydroxyacyl-coa dehydratase
LOC115559095											Gain		K06792	Aggrecan 1
LOC115559098						0.67								
LOC115559115						-0.53								Mitogen-activated protein kinase 8 interacting protein 1
LOC115559123				-0.42										Cytochrome c oxidase subunit 5a
LOC115559129						0.35							K25780	Kelch domain-containing protein 4
LOC115559134						0.47								Furin
LOC115559136						-0.39							K23969	FERM domain-containing protein 3/5
LOC115559147						-0.55								
LOC115559152							Loss		Loss				K04230	Galanin receptor 1
LOC115559153											Loss			
LOC115559173						-0.56								Rho gtpase-activating protein 17
LOC115559178									Gain					Tripartite motif-containing protein 35
LOC115559188												Gain		
LOC115559197	-0.56		-0.49			-0.62					Gain	Gain		
LOC115559199				0.47		0.51								

LOC115559211				0.48										
LOC115559217						0.50								Histone-binding protein RBBP4
LOC115559226	0.55													
LOC115559236	-2.13	-0.68	-2.03											
LOC115559240				-0.28		-0.37								Cytosolic phospholipase A2
LOC115559252				0.43										
LOC115559256											Loss	Loss		Complement C1q and tumor necrosis factor-related protein 4
LOC115559271				0.50		3.13					Gain	Gain		
LOC115559272						0.64			Gain		Gain	Gain		
LOC115559273						-0.67								
LOC115559284						-0.55								
LOC115559285	-1.91	-0.43	-1.02			-0.95								
LOC115559286	-1.30		-1.01											
LOC115559289	-0.40	-0.35	-0.53	-0.38		-0.41							K06816	Golgi apparatus protein 1
LOC115559303								Loss	Loss		Loss	Loss		Calretinin
LOC115559307	0.53													Regulator of G-protein signaling 9-binding protein
LOC115559313						-0.66						Gain		
LOC115559314						0.76							K18492	Insulin gene enhancer protein ISL-2
LOC115559319						1.00			Loss		Gain	Gain		
LOC115559320												Gain	K04697	Suppressor of cytokine signaling 4
LOC115559327	0.53	0.64	0.54										K18743	Caprin-1
LOC115559378						0.69								
LOC115559435	0.40		0.34			-0.57								
LOC115559442						0.41								Charged multivesicular body protein 6
LOC115559454	-3.56	-6.49												
LOC115559476	-0.73												K03663	4-galactosyl-N-acetylglucosaminide 3-alpha-L-fucosyltransferase
LOC115559486						-0.41								
LOC115559487	-0.39		-0.57											C-mer proto-oncogene tyrosine kinase
LOC115559488	0.80			-0.42										Nucleolar and coiled-body phosphoprotein 1
LOC115559489											Loss		K21989	Calcium permeable stress-gated cation channel
LOC115559490						-0.46								Calpain-1
LOC115559491								Loss						
LOC115559494	0.46												K06698	Proteasome activator subunit 3 (PA28 gamma)

LOC115559496						-0.41								Solute carrier family 29 (equilibrative nucleoside transporter), member 1/2/3
LOC115559517	-0.29					-0.29							K22403	Zinc finger MIZ domain-containing protein
LOC115559527						-0.50							K11251	Histone H2A
LOC115559531						0.68							K04440	Mitogen-activated protein kinase 8/9/10 (c-Jun N-terminal kinase)
LOC115559532						-0.59								Ankyrin, uncharacterized protein
LOC115559556			-0.39										K09095	Hypoxia-inducible factor 2 alpha
LOC115559562											Gain			
LOC115559570	-1.78	-0.49	-0.96										K15699	RING finger protein 122
LOC115559573			-0.32	-0.27		-0.47								
LOC115559579				-0.41		-0.85							K02259	Heme a synthase
LOC115559586						-0.56							K07424	Cytochrome P450 family 3 subfamily A
LOC115559604					0.81								K16629	Collagen type XXI alpha
LOC115559605					0.98								K08131	Collagen type IX alpha
LOC115559606											Loss	Loss		
LOC115559612	-0.49	-0.48	-0.63			-0.56								Collagen type XIX alpha
LOC115559615						0.60								
LOC115559616				0.46										Lysophosphatidic acid acyltransferase / lysophosphatidylinositol acyltransferase
LOC115559621		-0.40												L-asparaginase / beta-aspartyl-peptidase
LOC115559639			-0.28										K13097	Methylcytosine dioxygenase
LOC115559645			0.48											Ninein-like protein
LOC115559648				0.36									K26127	SMC5-SMC6 complex localization factor protein 2
LOC115559660						-0.52								
LOC115559662							Loss	Loss				Loss		
LOC115559663	0.65	0.43	0.45											
LOC115559672				-0.52		-0.85								General transcription factor IIIA, KRAB domain-containing zinc finger protein
LOC115559679	0.52		0.39											Sprouty-related, EVH1 domain-containing protein
LOC115559682				0.74										Tumor necrosis factor receptor superfamily member 14
LOC115559686						1.14								Elongation factor 1-alpha
LOC115559687	0.37													Phosphatidylinositol 4-kinase type 2
LOC115559692						-0.46							K06889, K13704	Abhydrolase domain-containing protein 12 , uncharacterized protein
LOC115559693						0.74							K00789	S-adenosylmethionine synthetase

LOC115559705										Loss	Loss	Loss	K10203	Elongation of very long chain fatty acids protein 6
LOC115559708				-0.47		-0.58								
LOC115559711			-0.41										K22074	NFU1 iron-sulfur cluster scaffold homolog, mitochondrial
LOC115559712							Loss	Loss						Transmembrane protein 150
LOC115559716	-0.55					-0.48								Prostamide/prostaglandin f2alpha synthase
LOC115559718	0.45			-0.52		-0.46								Translation initiation factor 4E
LOC115559722	0.36											Loss		Serine/threonine-protein phosphatase 2B regulatory subunit
LOC115559723	0.55												K19055	Ala-trna(Pro) deacylase
LOC115559726						-0.59								
LOC115559733	0.51												K02270	Cytochrome c oxidase subunit 7a
LOC115559746								Gain						
LOC115559750	0.67		0.42										K07152	Protein SCO1
LOC115559751								Loss	Loss		Gain			Nuclease HARBI1
LOC115559756						0.71							K17534	Mitogen-activated protein kinase kinase kinase MLK4
LOC115559762							Loss							Cadherin 5, type 2, VE-cadherin
LOC115559765						0.79								
LOC115559772						-0.42							K04496	C-terminal binding protein
LOC115559773	-0.96		-0.87											
LOC115559780	-0.62													Steroid 17alpha-monooxygenase / 17alpha-hydroxyprogesterone deacetylase
LOC115559781	-1.03	-0.56	-1.64											WW domain-binding protein 1
LOC115559783				-0.46		-1.03							K13811	3'-phosphoadenosine 5'-phosphosulfate synthase
LOC115559784											Loss			Multiple inositol-polyphosphate phosphatase / 2,3-bisphosphoglycerate 3-phosphatase
LOC115559786	0.34												K10839	UV excision repair protein RAD23
LOC115559789	0.90	0.58	1.12											Ribosome-binding protein 1
LOC115559796	-0.48												K22750	Glycine N-acyltransferase-like protein 3
LOC115559797						0.30								
LOC115559804		0.34	0.72										K10949	ER lumen protein retaining receptor
LOC115559810								Gain			Gain	Gain		
LOC115559811	-0.34													Dnaj homolog subfamily B member 12
LOC115559815	0.61												K01507	Inorganic pyrophosphatase
LOC115559818						-0.90							K07953	GTP-binding protein SAR1
LOC115559823						0.56								

LOC115559832							Gain				Loss	Loss		
LOC115559836				-0.46		-0.51			Loss		Loss	Gain		Multiple inositol-polyphosphate phosphatase / 2,3-bisphosphoglycerate 3-phosphatase
LOC115559839				-0.28		-0.32								Anthrax toxin receptor
LOC115559844						0.62								
LOC115559860				-0.58		-1.16								Regulator of G-protein signaling
LOC115559871				0.42									K07371	B-cell linker protein
LOC115559873				0.39										UTP--glucose-1-phosphate uridylyltransferase
LOC115559875	-0.31													Atlastin
LOC115559894			0.33						Gain				K01110	Phosphatidylinositol-3,4,5-trisphosphate 3-phosphatase and dual-specificity protein phosphatase PTEN
LOC115559902			0.43											NLR family CARD domain-containing protein 3
LOC115559906						-0.56								
LOC115559908	-0.83		-1.08											Nuclear factor, interleukin 3 regulated
LOC115559909				-0.43		-1.03							K16463	Centrosomal protein CEP170
LOC115559922	-0.79		-0.71			0.65							K16519	A-kinase anchor protein 2
LOC115559924						-0.68								
LOC115559925						0.61							K09565	Peptidyl-prolyl isomerase F (cyclophilin D)
LOC115559938	-2.68	-1.59	-2.68											
LOC115559941						-0.54								RNA-binding protein 5/10
LOC115559953	-0.57	-0.42	-0.62											Inhibitor of nuclear factor kappa-B kinase subunit alpha
LOC115559956						-0.82					Gain		K05700	Vinculin
LOC115559957						0.52							K12398	AP-3 complex subunit mu
LOC115559959	0.85	0.58	0.48			0.68							K00856	Adenosine kinase
LOC115559960						0.79							K14165	Atypical dual specificity phosphatase
LOC115559982	0.58													
LOC115559990	-0.78		-0.48			0.64								Tumor necrosis factor receptor superfamily member 6
LOC115560002				0.44									K07376	Cgmp-dependent protein kinase 1
LOC115560010	-0.37												K18622	Adducin
LOC115560011						-0.67								Collagen type XXI alpha
LOC115560031								Loss						
LOC115560032						0.46								Voltage-dependent anion channel protein 2, voltage-dependent anion channel protein 3
LOC115560049	-0.71	-0.48	-0.79			-1.12							K15014	Solute carrier family 29 (equilibrative nucleoside transporter), member 1/2/3

LOC115560055				0.53									K18932	Palmitoyltransferase
LOC115560058												Loss		Leucine-rich repeat, immunoglobulin-like domain and transmembrane domain-containing protein
LOC115560059							Loss	Loss						Cadherin-related family member 1
LOC115560061				-0.57		-0.95								Chondroitin 6-sulfotransferase 3
LOC115560069											Gain		K11251	Histone H2A
LOC115560091	-0.50												K22125	Myoferlin
LOC115560095				-0.45		-0.94						Gain		Leucine-rich repeat LGI family member 1
LOC115560097	-0.56	-0.54	-0.58			-0.99			Loss		Loss		K22028	Myopalladin
LOC115560098						0.52							K11252	Histone H2B
LOC115560099						-0.72								Protein FAM83H
LOC115560108								Loss	Loss				K06540	Disintegrin and metalloproteinase domain-containing protein 8
LOC115560110						-0.73							K04990	Polycystin 2L1
LOC115560122	-0.65													
LOC115560127	-0.73													
LOC115560128	-0.87													
LOC115560137							Gain					Loss		Regulator of microtubule dynamics protein 3
LOC115560139						0.59							K21444	Poly(rc)-binding protein 3/4
LOC115560162						0.80								
LOC115560167	0.37													4a-hydroxytetrahydrobiopterin dehydratase
LOC115560173										Gain	Gain	Gain		
LOC115560174							Loss							
LOC115560179												Loss		
LOC115560192								Loss						
LOC115560196				-0.45										Cytochrome c
LOC115560198						0.74								Solute carrier family 5 (high affinity choline transporter), member 7
LOC115560210												Loss		
LOC115560223						-0.56								
LOC115560224				0.71		0.73							K17277	Epidermal growth factor receptor kinase substrate 8
LOC115560230				-0.44		-1.14							K16500	Protocadherin-15
LOC115560239	-0.79	-0.41											K10137	Zinc finger, matrin type 3
LOC115560242	-0.79		-0.59											
LOC115560248				-0.88										Eukaryotic translation initiation factor 2-alpha kinase 2

LOC115560251						0.56								Eukaryotic translation initiation factor 2-alpha kinase 2
LOC115560255						0.54								
LOC115560266						-0.46								
LOC115560275						0.59								
LOC115560277	-0.41												K19477	Cgmp-dependent protein kinase 2
LOC115560283	-0.62			-0.43		-0.91								
LOC115560291								Gain	Gain		Loss	Loss		
LOC115560302						-0.36							K09624	Neurotrypsin
LOC115560304	-0.43		-0.60										K23294	Phosphofurin acidic cluster sorting protein 2
LOC115560307												Gain	K10407	Kinesin light chain
LOC115560308	-0.47													General transcription factor IIIA
LOC115560310						-0.77	Loss				Loss	Loss		
LOC115560319									Loss		Loss			
LOC115560320								Loss						Brorin
LOC115560326	-0.58													
LOC115560329						0.50								NLR family CARD domain-containing protein 3
LOC115560345												Gain		
LOC115560347	-0.49													NLR family CARD domain-containing protein 3
LOC115560349				0.45										NLR family CARD domain-containing protein 3
LOC115560357			-0.40											Nuclear factor of kappa light polypeptide gene enhancer in B-cells 2, shieldin complex subunit 2
LOC115560359	-0.88		-1.49										K04930	Potassium voltage-gated channel KQT-like subfamily member 5
LOC115560362									Gain			Gain + Loss		
LOC115560372	-1.72	-2.20	-3.20											
LOC115560376				0.74		0.68								
LOC115560382											Loss	Loss		
LOC115560384				-0.30		-0.65								
LOC115560394	0.49					0.56							K07375	Tubulin beta
LOC115560410							Loss					Gain		C-X-C motif chemokine 9
LOC115560419												Gain		
LOC115560420				-0.46		-1.28								Growth differentiation factor 10
LOC115560421						-0.38								
LOC115560435	-0.95	-0.67	-0.98										K11862	Ubiquitin thioesterase ZRANB1

LOC115560449				-0.28										
LOC115560465											Gain + Loss	Loss		
LOC115560472	-0.40		-0.61										K23687	Ras and Rab interactor 2/3
LOC115560473	0.46	0.38	0.44										K14290	Exportin-1
LOC115560474	-0.51		-0.67											Solute carrier family 24 (sodium/potassium/calcium exchanger), member 3
LOC115560477						0.68								Paired-like homeodomain transcription factor 3
LOC115560491						0.69								
LOC115560493	0.57		0.63										K15043	Importin subunit alpha-1/8
LOC115560494							Loss					Gain		
LOC115560497							Loss				Gain	Gain		MFS transporter, SP family, solute carrier family 2 (facilitated glucose transporter), member 9
LOC115560507						-0.68								Homeobox protein hoxa/B/C/D4
LOC115560533	0.50	0.42											K09516	All-trans-retinol 13,14-reductase
LOC115560549			0.45			-0.43							K11651	SWI/SNF-related matrix-associated actin-dependent regulator of chromatin subfamily E member 1
LOC115560617			0.49											Matrix metalloproteinase-23 (CA-MMP)
LOC115560618						-0.62								
LOC115560624			0.37	-0.37		-0.72								
LOC115560633	0.50													Hairy and enhancer of split 2/6/7
LOC115560634						-0.67								
LOC115560651	-0.68		-0.42			-0.47								Rab-interacting lysosomal protein
LOC115560652	0.40		0.57											
LOC115560655		0.50												
LOC115560658						-0.46								Multiple epidermal growth factor-like domains protein 6
LOC115560662			-0.40											
LOC115560663				0.67		0.83								Caspase 8
LOC115560668				0.84		1.06								
LOC115560669	0.54													
LOC115560678						1.75					Gain			
LOC115560682				0.42		0.84								
LOC115560685				0.75		0.65								
LOC115560686					-1.11									Caspase 1
LOC115560689						-0.64							K09665	Beta-1,3-N-acetylglucosaminyltransferase 8

LOC115560692				-0.42		-1.23								
LOC115560696							Loss					Loss		
LOC115560697	0.56	0.53	0.56			-0.73								Atpase family AAA domain-containing protein 5
LOC115560701		-0.36												Regulatory factor X 1/2/3
LOC115560707	-0.47		-0.68	0.44		0.81								
LOC115560712		-0.67				-0.65								Intraflagellar transport protein 80
LOC115560714						0.63								
LOC115560721										Gain		Gain		
LOC115560729						-0.75								
LOC115560737												Gain		
LOC115560744							Loss							
LOC115560760			-0.52			-0.71								
LOC115560762	-0.89													
LOC115560770						-0.79								KN motif and ankyrin repeat domain-containing protein
LOC115560775						-0.78								T-lymphoma invasion and metastasis-inducing protein 1
LOC115560776	-0.44													
LOC115560794				0.53									K05222	Transient receptor potential cation channel subfamily V member 1
LOC115560796						-0.94							K05222	Transient receptor potential cation channel subfamily V member 1
LOC115560797	-0.25		-0.22											Amyloid-like protein 2
LOC115560804							Loss						K04703	Sprouty-related, EVH1 domain-containing protein
LOC115560808						-0.71								Flotillin
LOC115560820						-0.79								Histone H3
LOC115560823							Gain							
LOC115560830				-0.40		-0.66							K04456	RAC serine/threonine-protein kinase
LOC115560831						0.42								
LOC115560833						-0.99							K04917	Potassium channel subfamily K member 6
LOC115560841	0.38		0.48											
LOC115560842				-0.51		-0.89							K08825	Dual specificity tyrosine-phosphorylation-regulated kinase 1
LOC115560843						-0.42								Microtubule-associated protein 6
LOC115560850											Gain			
LOC115560856											Gain	Gain		
LOC115560874						-0.60								Myeloid leukemia factor 1

LOC115560875											Gain	Gain	K13823, K13825	Hemoglobin subunit beta, hemoglobin subunit epsilon
LOC115560885				-0.49		-0.77							K12567	Titin
LOC115560893	0.32		0.47											Heterogeneous nuclear ribonucleoprotein L
LOC115560902	0.55					0.36					Gain		K10352	Myosin heavy chain 9/10/11/14
LOC115560903						-1.00								Alpha-2-macroglobulin-like protein
LOC115560908												Loss		
LOC115560912	-0.54													
LOC115560915	-1.15					0.74							K01332	Complement component 2
LOC115560935						0.77							K20526	Transgelin
LOC115560938							Gain							
LOC115560963						0.82								Dipeptidase E
LOC115560969						0.39							K16601	Tubulin polyglutamylase TTLL4
LOC115560979			-0.33				Loss							
LOC115560981												Gain + Loss		
LOC115561009						0.63								
LOC115561019						-1.15								
LOC115561036				0.45										Nuclear apoptosis-inducing factor 1
LOC115561045	0.80													
LOC115561049	0.93	0.13	0.74	0.35		0.38								
LOC115561056	0.63		0.90											Ribonucleoside-diphosphate reductase alpha chain , ribonucleoside-diphosphate reductase subunit M1
LOC115561057		-0.51	-0.42										K09253	Transcription factor relb
LOC115561066				-0.49		-1.13								
LOC115561070						0.41								
LOC115561080	-0.80					-0.58							K06616	Alpha-N-acetylgalactosaminide alpha-2,6-sialyltransferase (sialyltransferase 7B)
LOC115561101												Loss		
LOC115561107				0.55									K23687	Ras and Rab interactor 2/3
LOC115561110				0.49		0.56							K20912	Caspase recruitment domain-containing protein 10
LOC115561117						-0.84								
LOC115561144						-0.86								ATP-binding cassette, subfamily G (WHITE), member 1, ATP-binding cassette, subfamily G (WHITE), member 4
LOC115561159							Loss							Vomeronasal 2 receptor

LOC115561185						-0.71								Rho gtpase-activating protein 32
LOC115561192	-0.63	-0.51	-0.71										K09042	Transcription regulator BACH
LOC115561197											Loss	Loss		
LOC115561199						-0.86								
LOC115561208						-0.36							K08574	Calpain-5
LOC115561214						-0.66								Forkhead box protein O1
LOC115561228				-0.46		-1.07								
LOC115561238			-0.34											Deleted in liver cancer protein
LOC115561245	-1.78	-1.55	-1.82											
LOC115561247						0.81								Poliovirus receptor-related protein 3
LOC115561251	0.41		0.41											Arginyl aminopeptidase-like 1
LOC115561258						-0.98								FRAS1-related extracelluar matrix protein 1/2
LOC115561271						-0.87							K01124	Glycerophosphoinositol inositolphosphodiesterase
LOC115561280	-0.58		-0.73										K05743	LIM domain kinase 1
LOC115561285	-0.42					-0.65							K01992, K14429	ABC-2 type transport system permease protein, solute carrier family 12 (potassium/chloride transporters), member 9
LOC115561288			-0.52											ATP-dependent RNA helicase DDX17 , ATP-dependent RNA helicase DDX5/DBP2
LOC115561290	-0.61		-0.41										K24222	Inositol polyphosphate 5-phosphatase INPP5J/K
LOC115561292	-0.66					-0.52								Solute carrier family 38 (sodium-coupled neutral amino acid transporter), member 5
LOC115561301						0.61								Vitronectin
LOC115561306			-0.49											Alpha-2-macroglobulin-like protein
LOC115561307						-0.48							K14360	Organic solute transporter subunit alpha
LOC115561310						0.55								Alpha-2-macroglobulin-like protein
LOC115561317									Loss					
LOC115561319									Loss	Gain	Gain	Gain		
LOC115561330	-0.98		-0.71			-0.74								Neuroblast differentiation-associated protein AHNAK
LOC115561334								Loss			Loss	Loss		
LOC115561336				0.71										Guanylate cyclase activator 1
LOC115561338				-0.46										NADH dehydrogenase (ubiquinone) 1 subunit C2
LOC115561353	-0.23													Bromodomain and WD repeat domain containing protein 1/3
LOC115561366							Loss							
LOC115561367						0.38								

LOC115561369						0.69							K08745	Solute carrier family 27 (fatty acid transporter), member 1/4
LOC115561373						-1.07								
LOC115561379	-0.50			-0.44		-0.70								Tripartite motif-containing protein 35
LOC115561384	-0.46												K13616	Arylacetamide deacetylase
LOC115561397			-0.95			-1.78								
LOC115561415	0.65													DNA-directed RNA polymerase I subunit RPA34
LOC115561425			0.57											Multidrug resistance protein, MATE family
LOC115561428	-2.08	-0.55	-0.65	-0.46										Adenine phosphoribosyltransferase
LOC115561430	0.49	0.45	0.54	-0.32										Collagen beta-1,O-galactosyltransferase
LOC115561437			-0.47										K22449	Tubulin-specific chaperone cofactor E-like protein
LOC115561452											Loss	Loss		Numb
LOC115561463	0.87	0.46	0.78										K09501	Serpin peptidase inhibitor, clade H, member 1
LOC115561464								Loss	Loss		Loss		K09191	General transcription factor IIIA
LOC115561468						0.59								Cgmp-inhibited 3',5'-cyclic phosphodiesterase A
LOC115561469						-0.78							K20805	E3 ubiquitin-protein ligase RNF169
LOC115561470						-0.82								
LOC115561475												Loss	K00913	Inositol-1,3,4-trisphosphate 5/6-kinase / inositol-tetrakisphosphate 1-kinase
LOC115561478		-0.33				-0.36								Lysophosphatidic acid receptor 6
LOC115561482						-0.53								Sperm surface protein Sp17
LOC115561492	0.58		0.31										K12890	Serine/arginine-rich splicing factor 1
LOC115561493						0.62							K17603	Zinc finger FYVE domain-containing protein 1
LOC115561503						-0.99								
LOC115561515			-0.44											Solute carrier family 6 (neurotransmitter transporter, serotonin) member 4
LOC115561518						-0.61								
LOC115561520							Loss	Loss						
LOC115561521	0.24		0.43	-0.41		-0.31								
LOC115561527								Gain						BICD family-like cargo adapter
LOC115561529	0.50												K07755	Arsenite methyltransferase
LOC115561530	-0.42													Protein FAM98B
LOC115561536			0.40											
LOC115561548											Gain			
LOC115561553				0.57										

LOC115561557						0.36							K17824	DCN1-like protein 4/5
LOC115561561	-0.51		-0.44											
LOC115561567											Loss	Loss		Von Willebrand factor A domain-containing protein 7
LOC115561569									Loss	Loss	Gain + Loss			Nuclease HARBI1
LOC115561570											Gain	Gain		Trace amine associated receptor
LOC115561592						-0.92								
LOC115561597				-0.53		-0.74							K12567	Titin
LOC115561610												Gain	K07497	Putative transposase
LOC115561612						-0.75								
LOC115561614						0.58						Loss		Fer-1-like protein 6
LOC115561615						-1.06								Autophagy-related protein 18
LOC115561625							Loss		Loss		Loss	Loss		
LOC115561627							Loss				Loss	Loss		Protein unc-119
LOC115561630	0.52			-0.46		-0.66							K00545	Catechol O-methyltransferase
LOC115561635				0.39		0.78								
LOC115561654											Gain	Gain		
LOC115561658						0.38							K11657	Remodeling and spacing factor 1
LOC115561668	0.42													Arfaptin
LOC115561672				0.48									K06767	Down syndrome cell adhesion molecule
LOC115561674						0.53							K10418	Dynein light chain LC8-type
LOC115561677						-0.97								
LOC115561683						-0.74							K18753	Butyrate response factor
LOC115561684						-0.54							K04429	Thousand and one amino acid protein kinase
LOC115561703			0.33			-0.41								Protein-tyrosine sulfotransferase
LOC115561705	0.40												K00948	Ribose-phosphate pyrophosphokinase
LOC115561707						-0.53								EF-hand domain-containing family member D1
LOC115561719	-0.50													PDZ domain-containing protein 3
LOC115561720			-0.49										K15103	Solute carrier family 25 (mitochondrial uncoupling protein), member 8/9
LOC115561721		-0.90	-0.91			-0.71							K12185	ESCRT-I complex subunit VPS37
LOC115561726							Loss							
LOC115561731						-0.91							K18753	Butyrate response factor
LOC115561745						-0.33								RNA-binding protein 7

LOC115561751	-0.86		-1.05											
LOC115561752				0.43		0.74							K00907	Myosin-light-chain kinase
LOC115561759				1.76									K23284	C1q-related factor
LOC115561761	-1.18												K07200	5'-AMP-activated protein kinase, regulatory gamma subunit
LOC115561770						-0.87							K25605	B-cell CLL/lymphoma 7 protein
LOC115561779						0.55							K23482	Beta-crystallin
LOC115561784									Loss					
LOC115561791						0.68								
LOC115561800	1.45												K09044	Activating transcription factor 5
LOC115561809	-0.63										Loss	Loss		
LOC115561814	1.00											Gain		
lonp1	0.70	0.39	0.37										K08675	ATP-dependent Lon protease
lonp2			-0.33			-0.57							K01338	ATP-dependent Lon protease
lonrf2	-0.35		-0.35											
loxl3						0.50							K00280	Lysyl oxidase-like protein 2/3/4
lpcat2						0.34							K13510	Lysophosphatidylcholine acyltransferase / lyso-PAF acetyltransferase
lpcat3				0.43									K13515	Lysophospholipid acyltransferase 5
lpgat1	0.34													Lysophosphatidylglycerol acyltransferase 1
lpin1	-0.42													Phosphatidate phosphatase LPIN
lpl	-0.34		-0.50											Lipoprotein lipase
lrch3		0.24				0.33								
lrig2							Gain							Leucine-rich repeats and immunoglobulin-like domains protein 2
lrp10	-0.39	-0.33	-0.36									Loss	K20050	Low-density lipoprotein receptor-related protein 3/10/12
lrp1b											Gain	Gain		Low-density lipoprotein receptor-related protein 1B
lrp4				0.51		1.17								Low-density lipoprotein receptor-related protein 4
lrp5						-0.35	Gain							Low density lipoprotein receptor-related protein 5/6
lrpap1				-0.31		-0.33								Alpha-2-macroglobulin receptor-associated protein
lrpprc	0.51			-0.38									K17964	Leucine-rich PPR motif-containing protein, mitochondrial
lrrc1						-0.53								Protein scribble
lrrc2	-1.88	-0.85	-1.21			-0.90								
lrrc28						0.57								
lrrc30	-0.74					0.70								

lrrc42	-0.27		-0.41											
lrrc45	-1.38	-0.82	-0.92											Leucine-rich repeat-containing protein 45
lrrc47		0.31		-0.57										
lrrc56						-0.59							K25425	Leucine-rich repeat-containing protein 56
lrrc58						-0.54								
lrrc59				-0.63										
lrrc66	-1.36		-1.17											
lrrc73	0.86													
lrrc75a						-0.60								
lrrc8a			-0.51											Volume-regulated anion channel
lrrcc1			0.48										K16475	Leucine-rich repeat and coiled-coil domain-containing protein 1
lrrk1				0.43									K08843	Leucine-rich repeat kinase 1
lrrk2						0.54	Gain						K08844	Leucine-rich repeat kinase 2
lrrn1				-0.46		-0.80								Leucine-rich repeat neuronal protein 1/2/3, OTU domain-containing protein 5
lrsam1	-0.73		-0.75										K10641	E3 ubiquitin-protein ligase LRSAM1
lrtm1						-0.59								
lrwd1			0.42				Loss							Leucine-rich repeat and WD repeat-containing protein 1
lsm2	0.44													U6 snrna-associated Sm-like protein lsm2
lsm4	0.34					0.32								U6 snrna-associated Sm-like protein lsm4
ltbp1						-0.44								Latent transforming growth factor beta binding protein 1
ltbp3						-0.32								
ltn1	0.44	0.41												E3 ubiquitin-protein ligase listerin
ltv1						0.45							K14798	Protein LTV1
luc7l						0.81							K13212	RNA-binding protein Luc7-like 2
lurap1l						-0.64					Loss	Loss		
lyar	0.38			-0.44									K15263	Cell growth-regulating nucleolar protein
lypla1			-0.49											Lysophospholipase I , lysophospholipase II , phospholipase/carboxylesterase
lyplal1			-0.68									Gain	K06999	Phospholipase/carboxylesterase
lyrm2				-0.48										
lyrm9				-0.49		-0.80								
lysmd2	0.38													
lyst	0.84		0.34										K22937	Lysosomal-trafficking regulator

lzic						-0.55			Gain					
lzts2	-0.45													
maco1				0.48		0.44								
mad2l1			0.42										K02537	Mitotic spindle assembly checkpoint protein MAD2
maea	-0.32	-0.24	-0.33											Macrophage erythroblast attacher
maf						0.93								Transcription factor Maf
maf1	-0.94		-0.49											Repressor of RNA polymerase III transcription MAF1
mafa						0.66								Transcription factor MAFA
mafk	-1.07		-2.15										K09037	Transcription factor MAFF/G/K
magix												Loss		
magoh	0.52					0.63							K12877	Protein mago nashi
magt1	0.38													Magnesium transporter 1, oligosaccharyltransferase complex subunit gamma
mak16						0.55							K14831	Protein MAK16
mal						0.94								
maml2						-0.88								Mastermind
man1a2	0.29												K01230	Mannosyl-oligosaccharide alpha-1,2-mannosidase
man1b1	-0.46		-0.43											Endoplasmic reticulum Man9GlcNAc2 1,2-alpha-mannosidase
man2a2	0.44	0.40	0.65											Alpha-mannosidase II
man2b1	-0.43												K12311	Lysosomal alpha-mannosidase
man2b2				0.59										Epididymis-specific alpha-mannosidase
manba				0.64		0.46							K01192	Beta-mannosidase
manf	0.84	0.61	0.62			0.61							K22556	Mesencephalic astrocyte-derived neurotrophic factor
map1lc3a	-0.65		-0.84			-1.21							K10435	Microtubule-associated protein 1 light chain
map2k5						-0.57								Mitogen-activated protein kinase kinase 5
map3k1						-0.61								Mitogen-activated protein kinase kinase kinase 1
map3k12	-0.69			-0.76		-0.90							K04423	Mitogen-activated protein kinase kinase kinase 12
map3k2											Loss		K04420, K04421	Mitogen-activated protein kinase kinase kinase 2 , mitogen-activated protein kinase kinase kinase 3
map3k3	-0.86												K04421	Mitogen-activated protein kinase kinase kinase 3
map3k4		0.33	0.60											Mitogen-activated protein kinase kinase kinase 4
map4k2						-0.49							K04414	Mitogen-activated protein kinase kinase kinase kinase 2
map7				0.51										Microtubule-associated protein 7
map7d1						-0.71		Loss			Gain			MAP7 domain-containing protein 1

mapk1			-0.46											Mitogen-activated protein kinase 1/3
mapk10				-0.42		-0.77							K04440	Mitogen-activated protein kinase 8/9/10 (c-Jun N-terminal kinase)
mapk15				0.42									K19603	Mitogen-activated protein kinase 15
mapk4												Gain		Mitogen-activated protein kinase 4/6
mapk6			0.25											Mitogen-activated protein kinase 4/6
mapkapk5	0.35		0.35										K04442	Mitogen-activated protein kinase-activated protein kinase 5
mapkbp1						0.75							K21763	Mitogen-activated protein kinase binding protein 1
mapre1	0.84	0.46	0.68											Microtubule-associated protein, RP/EB family
mapre2				-0.35		-0.75							K10436	Microtubule-associated protein, RP/EB family
mars2	-0.51	-0.31	-0.61										K01874	Methionyl-tRNA synthetase
mars3			-0.40										K01874	Methionyl-tRNA synthetase
marchf2						-0.49							K10657	E3 ubiquitin-protein ligase MARCH2
marchf7						-0.39								E3 ubiquitin-protein ligase MARCH7
marcks				0.41										Myristoylated alanine-rich C-kinase substrate
mark1				-0.45		-1.17							K08798	MAP/microtubule affinity-regulating kinase
mark2	0.41												K08798	MAP/microtubule affinity-regulating kinase
mark3	0.85	0.60	0.57										K08798	MAP/microtubule affinity-regulating kinase
masp2							Loss		Loss		Loss	Gain		
mast4	-1.17	-0.74	-1.13										K08789	Microtubule-associated serine/threonine kinase
mastl			0.55										K16309	Serine/threonine-protein kinase greatwall
mat2a	1.22	0.85	0.75										K00789	S-adenosylmethionine synthetase
matn2						0.54		Loss				Loss	K19467	Matrilin
mau2	-0.87		-0.77											Maternally affected uncoordination
mb	0.45		0.52											Myoglobin
mbd4	-1.31	-0.87	-1.25											Methyl-cpg-binding domain protein 4
mbd5						-0.37							K23219	Methyl-cpg-binding domain protein 5
mblac1				-0.52										
mbnl1	-1.32	-0.71	-0.70			-0.51								Muscleblind
mbtps2	0.60		0.44			0.40								S2P endopeptidase
mc2r						-0.56							K04200	Melanocortin 2 receptor
mcam						-0.46								Melanoma cell adhesion molecule
mccc1				-0.71		-0.85							K01968	3-methylcrotonyl-coa carboxylase alpha subunit
mcee				-0.42										Methylmalonyl-coa/ethylmalonyl-coa epimerase

mcf2l												Gain	K20685	Guanine nucleotide exchange factor MCF2
mcfd2						0.86							K20364	Multiple coagulation factor deficiency protein 2
mcidas									Gain		Gain		K26119	Multicilin
mcm10	1.22	0.88	0.80											Minichromosome maintenance protein 10
mcm2	1.74	1.57	1.67											DNA replication licensing factor MCM2
mcm3	1.37	0.94	1.13			-0.52							K02541	DNA replication licensing factor MCM3
mcm3ap				-0.35		-1.00							K24317	Germinal-center associated nuclear protein
mcm4	1.56	0.83	1.35			-0.80							K02212	DNA replication licensing factor MCM4
mcm5	1.66	1.34	1.61										K02209	DNA replication licensing factor MCM5
mcm7	0.85	0.52	0.70											DNA replication licensing factor MCM7
mcmbp	0.82		0.59											
mcmdc2						0.58								
mcrip2			-0.43			0.46								
mctp1				0.54										
mdc1						-0.46								Mediator of DNA damage checkpoint protein 1
mdm2	0.37												K06643	E3 ubiquitin-protein ligase Mdm2
mdn1						0.54								Midasin
me1												Loss	K00029	Malate dehydrogenase (oxaloacetate-decarboxylating)(NADP+)
med1	0.38		0.36			-0.50							K15144	Mediator of RNA polymerase II transcription subunit 1
med10	0.27		0.28	-0.38										Mediator of RNA polymerase II transcription subunit 10
med15			0.35											Mediator of RNA polymerase II transcription subunit 15
med20	0.44													Mediator of RNA polymerase II transcription subunit 20
med21	0.58	0.48	0.60											Mediator of RNA polymerase II transcription subunit 21
med22	0.44		0.50										K15139	Mediator of RNA polymerase II transcription subunit 22
med28			0.35											Mediator of RNA polymerase II transcription subunit 28
med31				-0.39		-0.44								Mediator of RNA polymerase II transcription subunit 31
med6	0.26			-0.27									K15128	Mediator of RNA polymerase II transcription subunit 6
med7				-0.29		-0.30								Mediator of RNA polymerase II transcription subunit 7
mef2b											Loss	Loss		MADS-box transcription enhancer factor 2B
megf11									Loss		Gain			Multiple epidermal growth factor-like domains protein 10/11
meioc							Loss			Gain	Gain	Gain		
melk	0.59		0.57										K08799	Maternal embryonic leucine zipper kinase
meox1						0.39								Homeobox protein MOX

meox2						-0.71								Homeobox protein MOX
mesd	0.34													LRP chaperone MESD
mest						0.39								
metap1				-0.40										Methionyl aminopeptidase
mettl1	0.48		0.42										K03439	Trna (guanine-N7-)-methyltransferase
mettl16	0.86		0.75										K11393	U6 snrna m6a methyltransferase
mettl17						0.62								
mettl21a						0.73								Protein N-lysine methyltransferase METTL21A
mettl26						-0.42								
mettl3	0.58		0.48										K05925	Mrna m6a methyltransferase catalytic subunit
mettl8		-0.50												Mrna N(3)-methylcytidine methyltransferase METTL8
mettl9			0.40											
mex3b				-0.46		-1.27							K15686	RNA-binding protein MEX3
mfap1												Gain	K13110	Microfibrillar-associated protein 1
mfap2						0.43			Loss				K25406	Microfibrillar-associated protein 2
mfn1			-0.32			-0.57								Mitofusin 1
mfn2						-0.48							K06030, K21356	Mitofusin 1 , mitofusin 2
mfsd1	-0.98	-0.47	-0.74											
mfsd13a	0.40	0.61	0.51											
mfsd14a				-0.32		-0.46								
mfsd3	-0.66													MFS transporter, PAT family, beta-lactamase induction signal transducer ampg
mfsd4a							Gain					Loss		MFS transporter, FHS family, Na+ dependent glucose transporter 1
mfsd5			0.44										K24175	MFS transporter, MFS domain-containing protein family, molybdate-anion transporter
mfsd8						-0.49							K12307	MFS transporter, ceroid-lipofuscinosis neuronal protein 7
mfsd9						-0.54								
mgat2						0.36							K00736	Alpha-1,6-mannosyl-glycoprotein beta-1,2-N-acetylglucosaminyltransferase
mgat4a						0.48							K00738	Alpha-1,3-mannosylglycoprotein beta-1,4-N-acetylglucosaminyltransferase A/B
mgll				-0.41		-0.81								Acylglycerol lipase
mgp					1.23									Matrix Gla protein
mgrn1	-0.33	-0.38	-0.40											E3 ubiquitin-protein ligase MGRN1

mia2	-0.35												K23703	Melanoma inhibitory activity protein 2
mia3		0.45	0.38			0.40							K23704	Transport and golgi organization protein 1
mib1						0.83							K10645	E3 ubiquitin-protein ligase mind-bomb
mical2	-0.59		-0.57											F-actin monooxygenase
micu2	-0.47													Calcium uptake protein 2, mitochondrial
mid1ip1	-1.34	-1.02	-1.08											
mier3						-0.32								
miga1						-0.47								
miga2	0.52													
miip	0.40													
minpp1						-0.54							K03103	Multiple inositol-polyphosphate phosphatase / 2,3-bisphosphoglycerate 3-phosphatase
miox	-0.69		-0.99										K00469	Inositol oxygenase
mipep	0.70													Mitochondrial intermediate peptidase
mis12	0.59		0.72											Kinetochore-associated protein MIS12
mis18bp1			0.64											
mki67	0.70		0.82										K17582	Antigen KI-67
mknk2	-1.22	-0.54	-1.70										K04372	MAP kinase interacting serine/threonine kinase
mkrn1	-0.58		-0.43											E3 ubiquitin-protein ligase makorin
mkrn2						0.34							K15687	E3 ubiquitin-protein ligase makorin
mkx	-0.36												K24890	Homeobox protein Mohawk
mlc1								Gain						Membrane protein MLC1
mlec	0.96	0.64	0.77											
mlh1			0.40											DNA mismatch repair protein MLH1
mlip	-0.70		-0.58											
mllt11	-0.54	-0.40	-0.55											
mllt3		0.71	1.31											YEATS domain-containing protein 1/3
mllt6						0.59							K23588	Protein AF-17/10
mlx	-0.88	-0.39	-1.11								Loss	Loss	K09113	MAX-like protein X
mmadhc	0.23					0.25							K26006	Cobalamin trafficking protein cbld
mmd	0.42												K11064	Monocyte to macrophage differentiation protein
mmel1						-0.45								Neprilysin
mmp13						0.88							K07994	Matrix metalloproteinase-13 (collagenase 3)

mmp2				-0.40		-0.89								Matrix metalloproteinase-2 (gelatinase A)
mmp28						-0.47							K08006	Matrix metalloproteinase-28 (epilysin)
mms19	0.59	0.42	0.29			0.42							K15075	DNA repair/transcription protein MET18/MMS19
mms22l	0.92		0.50										K26125	Protein MMS22-like
mmaa	0.42													Gtpase
mnd1	0.58					-0.52								
mns1	0.74		0.44								Gain	Gain		Meiosis-specific nuclear structural protein 1
mnt						-0.88							K09115	MAX-binding protein
mocos								Loss			Gain			Elongator complex protein 2, molybdenum cofactor sulfurtransferase
mocs1						0.71	Gain	Gain	Gain					Cyclic pyranopterin monophosphate synthase , GTP 3',8-cyclase , GTP 3',8-cyclase / cyclic pyranopterin monophosphate synthase
mocs3	0.50		0.62											Adenylyltransferase and sulfurtransferase
mogs	0.84		0.64										K01228	Mannosyl-oligosaccharide glucosidase
mok						-0.70							K08830	Renal tumor antigen
morc2				0.51		0.67								MORC family CW-type zinc finger protein
mosmo												Loss		Modulator of smoothened protein
mospd1	-0.92	-0.72	-1.12											
mospd2	-0.53	-0.30	-0.42	0.40		0.43								
mpdu1	0.38		0.47										K09660	Mannose-P-dolichol utilization defect 1
mpeg1	-0.55		-0.55											
mpg	-0.77	-0.39	-0.48				Gain						K03652	DNA-3-methyladenine glycosylase
mphosph10	0.41					0.48							K14559	U3 small nucleolar RNA-associated protein MPP10
mphosph6	0.39			-0.39									K12593	M-phase phosphoprotein 6, animal type
mphosph8						-0.59						Gain		M-phase phosphoprotein 8
mphosph9	0.42		0.48										K16775	M-phase phosphoprotein 9
mpp1	-0.48		-0.39	0.47									K21880	MAGUK 55 kda erythrocyte membrane protein
mpp3							Gain		Gain		Gain	Gain		MAGUK p55 subfamily member 3/7
mpp7						-0.70							K24049	MAGUK p55 subfamily member 3/7
mprip	-0.94	-0.87	-1.00										K23792	Myosin phosphatase Rho-interacting protein
mpv17	0.37													Protein Mpv17
mpv17l2	0.70	0.43				0.63								Protein Mpv17
mras						0.58								Ras-related protein M-Ras
mrc2	0.36												K06560	Mannose receptor, C type

mre11			0.46										K10865	Double-strand break repair protein MRE11
mri1	0.37												K00899, K08963	5-methylthioribose kinase , methylthioribose-1-phosphate isomerase
mrm1	0.62		0.39			0.52								21S rrna (GM2251-2'-O)-methyltransferase
mrm2				-0.42									K02427	23S rrna (uridine2552-2'-O)-methyltransferase
mrm3	0.62												K20095	16S rrna (guanosine(1370)-2'-O)-methyltransferase
mroh1				0.46		0.75								Maestro heat-like repeat-containing protein family member 1
mrpl11	0.37			-0.46									K02867	Large subunit ribosomal protein L11
mrpl12	0.67	0.43	0.59										K02935	Large subunit ribosomal protein L7/L12
mrpl13	0.40												K02871	Large subunit ribosomal protein L13
mrpl18	0.38			-0.45										Large subunit ribosomal protein L18
mrpl19	0.33												K02884	Large subunit ribosomal protein L19
mrpl20	1.13	0.64	0.56			0.44							K02887	Large subunit ribosomal protein L20
mrpl22	0.57													Large subunit ribosomal protein L22
mrpl24	0.47			-0.37									K02895	Large subunit ribosomal protein L24
mrpl27						0.37								Large subunit ribosomal protein L27
mrpl3				-0.47									K02906	Large subunit ribosomal protein L3
mrpl30				-0.57		-0.49							K02907	Large subunit ribosomal protein L30
mrpl37	0.58		0.47											Large subunit ribosomal protein L37
mrpl38						0.39								Large subunit ribosomal protein L38
mrpl4	0.59		0.37										K02926	Large subunit ribosomal protein L4
mrpl40	0.86		0.32	-0.38									K17421	Large subunit ribosomal protein L40
mrpl42				-0.39										Large subunit ribosomal protein L42
mrpl43	0.42			-0.42									K17424	Large subunit ribosomal protein L43
mrpl45	0.44	0.39	0.37											Large subunit ribosomal protein L45
mrpl47	0.70												K17428	Large subunit ribosomal protein L47
mrpl51				-0.51									K17432	Large subunit ribosomal protein L51
mrpl54	0.78		0.43										K17435	Large subunit ribosomal protein L54
mrpl58	0.50		0.37											Peptidyl-trna hydrolase ICT1
mrpl9	0.30												K02939	Large subunit ribosomal protein L9
mrps10				-0.29									K02946	Small subunit ribosomal protein S10
mrps12				-0.44		-0.56								Small subunit ribosomal protein S12
mrps16				-0.39									K02959	Small subunit ribosomal protein S16

mrps17	0.65												K02961	Small subunit ribosomal protein S17
mrps18a	0.55		0.41										K02963	Small subunit ribosomal protein S18
mrps18b						0.53							K16174	Small subunit ribosomal protein s18b, mitochondrial
mrps23	0.31			-0.32										Small subunit ribosomal protein S23
mrps26	0.48													Small subunit ribosomal protein S26
mrps30	0.51	0.48											K17409	Small subunit ribosomal protein S30
mrps31	0.57												K17410	Small subunit ribosomal protein S31
mrps34	0.56												K17412	Small subunit ribosomal protein S34
mrps36				-0.41										Small subunit ribosomal protein S36
mrps6				-0.81							Gain	Gain	K02990	Small subunit ribosomal protein S6
mrps7	0.55			-0.45										Small subunit ribosomal protein S7
mrs2	-0.37		-0.36	-0.34		-0.68					Loss			Magnesium transporter
mrtfa	-0.51	-0.43	-0.48										K22525	MKL/myocardin-like protein
mrtfb				0.31										MKL/myocardin-like protein
mrto4						0.72							K14815	Mrna turnover protein 4
mrvi1								Loss	Loss		Loss	Loss	K12337	Inositol 1,4,5-triphosphate receptor-associated cgmp kinase substrate
msantd1											Loss			
msantd4						-0.48								
msh2	1.06	0.67	0.77											DNA mismatch repair protein MSH2
msh3	-0.36												K08736	DNA mismatch repair protein MSH3
msh6	0.49		0.41											DNA mismatch repair protein MSH6
msi1						-0.67								RNA-binding protein Musashi
msl1	-0.30		-0.56										K13163	Male-specific lethal 1
msra				-0.43		-0.56							K07304	Peptide-methionine (S)-S-oxide reductase
msrb2	0.54										Loss	Loss	K07305	Peptide-methionine (R)-S-oxide reductase
msrb3				-0.74									K07305	Peptide-methionine (R)-S-oxide reductase
mss51						0.80								Mitochondrial splicing suppressor protein 51
msto1	0.58													
msx1						-0.71								Homeobox protein MSX
mta2			0.41										K11660	Metastasis-associated protein MTA
mtap	0.42												K00772	5'-methylthioadenosine phosphorylase
mtbp	0.46													Mdm2-binding protein

mtcl1											Loss			
mtdh						0.46								
mterf1	0.65		0.57										K15031, K15032	Mterf domain-containing protein, mitochondrial, transcription termination factor, mitochondrial
mterf2											Loss	Loss	K15031	Transcription termination factor, mitochondrial
mterf3			-0.41											Mterf domain-containing protein, mitochondrial
mterf4	0.55		0.41											
mtf1						-0.73							K09191, K23317	General transcription factor IIIA, metal regulatory transcription factor 1
mtf2						-0.45								Metal regulatory transcription factor 2
mtfr1l						-0.26								
mtg2	0.63													Gtpase
mthfd1	0.57												K00288	Methylenetetrahydrofolate dehydrogenase (NADP+) / methenyltetrahydrofolate cyclohydrolase / formyltetrahydrofolate synthetase
mthfd2				0.41										Methylenetetrahydrofolate dehydrogenase(NAD+) / 5,10-methenyltetrahydrofolate cyclohydrolase
mtif2	0.82												K02519	Translation initiation factor IF-2
mtmr12						0.56								
mtmr14	-0.78	-0.61	-0.85										K18086	Myotubularin-related protein 14
mtmr3	-0.84	-0.77	-0.88											Myotubularin-related protein 3/4
mtmr9	-1.22	-0.85	-0.96											Myotubularin-related protein 9
mtnr1a	-0.82		-0.50										K04285	Melatonin receptor type 1A
mto1			0.38										K03495	Trna uridine 5-carboxymethylaminomethyl modification enzyme
mtpap	0.88	0.61	0.57											Poly(A) RNA polymerase, mitochondrial
mtrex	0.65	0.58	0.59										K12598	ATP-dependent RNA helicase DOB1
mtrf1l	0.35		0.35											Peptide chain release factor 1
mtrr						0.65						Loss	K00597	Methionine synthase reductase
musk			-0.93											Muscle, skeletal, receptor tyrosine kinase
mvb12b						-0.50							K12186	ESCRT-I complex subunit MVB12
mvk						0.60								Mevalonate kinase
mxd1	-0.61			0.47		0.62								MAX dimerization protein
mxra5							Loss				Loss	Loss		Matrix remodeling-associated protein 5
myadm						-0.98								
mybbp1a	0.61												K02331	DNA polymerase phi

mybl2	0.94		0.55										K21769	Myb-related protein B
mybpc2						0.67		Loss	Loss			Gain	K12558	Myosin-binding protein C, fast-type
mycbp2	0.32			0.34									K10693	RCR-type E3 ubiquitin transferase
myd88			0.52											Myeloid differentiation primary response protein myd88
mydgf	0.66	0.40	0.83			0.37								
myef2						0.44								Myelin expression factor 2
myh11						0.75							K10352	Myosin heavy chain 9/10/11/14
myh9			0.44										K10352	Myosin heavy chain 9/10/11/14
mylk	-0.48													Myosin-light-chain kinase
mymk	0.45													Protein myomaker
myo18b	-0.54													Myosin XVIII
myo1b						0.79								Myosin I
myo1e				0.33		0.33								Myosin I
myo3b	0.53													Myosin III
myo5c				0.59									K10357	Myosin V
myo9b	0.37		0.30	0.42		0.31							K10360	Myosin IX
myoc	-0.59			-0.57		-0.82								Myocilin
myocd			-0.43										K22526	Myocardin
myorg	-0.80	-0.53	-1.10										K24727	Myogenesis-regulating glycosidase
myoz1	-0.74												K26050	Myozenin
mysm1	-0.58	-0.32	-0.60										K11865	Histone H2A deubiquitinase
mzt1	0.36		0.38											Mitotic-spindle organizing protein 1
nacc1		0.41												BTB/POZ domain-containing protein 14
nadk2	-0.43		-0.46											NAD+ kinase
nadsyn1			0.41											NAD+ synthase (glutamine-hydrolysing)
nae1	-0.38												K04532	NEDD8-activating enzyme E1 regulatory subunit
naf1	0.59	0.34	0.55											H/ACA ribonucleoprotein complex non-core subunit NAF1
nagpa	-0.64					0.37								N-acetylglucosamine-1-phosphodiester alpha-N-acetylglucosaminidase
nanos1						-1.29								Protein nanos 1
nans											Loss		K05304	Sialic acid synthase
nap1l1			0.39			0.34							K11279	Nucleosome assembly protein 1-like 1
naprt						-0.41							K00763	Nicotinate phosphoribosyltransferase

narf	0.56	0.45	0.69											
nasp	0.53	0.52	0.68										K11291	Nuclear autoantigenic sperm protein
nat14	0.48													
nat9						0.60								Tubulin N-terminal N-acetyltransferase
naxd			0.43										K17757	ATP-dependent NAD(P)H-hydrate dehydratase
nbeal2				0.47		0.79								Neurobeachin-like protein 1/2
ncan						0.72							K06794	Neurocan core protein
ncapd2	0.39		0.58										K06677	Condensin complex subunit 1
ncapd3			0.54											Condensin-2 complex subunit D3
ncapg			0.43										K06678	Condensin complex subunit 3
ncaph	0.52					-0.63							K06676	Condensin complex subunit 2
ncaph2			0.49											Condensin-2 complex subunit H2
ncbp1	0.67		0.60										K12882	Nuclear cap-binding protein subunit 1
ncbp2	0.49												K12883	Nuclear cap-binding protein subunit 2
ncbp2as2	0.74		0.69											
ncbp3	0.33	0.37												
ncdn						0.75								
nceh1						-0.64								Neutral cholesterol ester hydrolase 1
ncf1						0.75								Neutrophil cytosolic factor 1
ncf2				0.48		1.04							K08010	Neutrophil cytosolic factor 2
nckap1	0.28	0.29	0.36										K05750	NCK-associated protein 1
nckap5l						-1.00								
nckipsd						-0.50								
ncl	0.61			-0.43									K11294	Nucleolin
ncln	0.91		0.73											
ncoa3						-0.46	Gain + Loss	Loss	Gain + Loss		Gain + Loss	Gain + Loss	K11256	Nuclear receptor coactivator 3
ncoa5	0.44	0.29	0.52			-0.33								
ncor1	-0.35	-0.37	-0.34			0.35							K04650	Nuclear receptor co-repressor 1
ncor2						-0.58							K06065	Nuclear receptor co-repressor 2
ncstn						-0.63								Nicastrin
ndc1	0.49		0.42											Nucleoporin NDC1
ndc80			0.58											Kinetochore protein NDC80

nde1				0.35										Nuclear distribution protein nude
ndfip2	-0.36												K25839	NEDD4 family-interacting protein
ndor1						-0.65								
ndrg4		-0.42							Gain		Gain	Gain	K18266	Protein NDRG1
ndst1	0.36	0.30	0.48											Heparan sulfate N-deacetylase/N-sulfotransferase NDST1
ndst2			0.29										K02577	Heparan sulfate N-deacetylase/N-sulfotransferase NDST2
ndufa11				-0.38										NADH dehydrogenase (ubiquinone) 1 alpha subcomplex subunit 11
ndufa2				-0.38									K03946	NADH dehydrogenase (ubiquinone) 1 alpha subcomplex subunit 2
ndufa8				-0.50								Gain	K03952	NADH dehydrogenase (ubiquinone) 1 alpha subcomplex subunit 8
ndufaf3	0.44													NADH dehydrogenase
ndufaf6	0.58													NADH dehydrogenase
ndufb11				-0.47									K11351	NADH dehydrogenase (ubiquinone) 1 beta subcomplex subunit 11
ndufb3				-0.48										NADH dehydrogenase (ubiquinone) 1 beta subcomplex subunit 3
ndufb7				-0.38										NADH dehydrogenase (ubiquinone) 1 beta subcomplex subunit 7
ndufs4				-0.40										NADH dehydrogenase (ubiquinone) Fe-S protein 4
ndufs6				-0.43									K03939	NADH dehydrogenase (ubiquinone) Fe-S protein 6
nebl			-0.38										K24420	LIM and SH3 domain protein 1, nebulette
necab2				-0.47		-0.85								N-terminal EF-hand calcium-binding protein
necap2						-0.47								Adaptin ear-binding coat-associated protein 1/2
nedd4	-0.32													E3 ubiquitin-protein ligase NEDD4
nedd4l						0.60								E3 ubiquitin-protein ligase NEDD4 , E3 ubiquitin-protein ligase NEDD4-like
neil1											Gain	Gain	K10567	Endonuclease VIII-like 1
nek4							Gain				Gain	Gain	K08857	Serine/threonine-protein kinase Nek1/4/5
nek8						-0.50								Serine/threonine-protein kinase Nek8
nelfb						0.51								Negative elongation factor B
nelfcd	-0.31													Negative elongation factor C/D
nemf						0.45								Nuclear export mediator factor NEMF
nenf							Gain	Gain				Gain		Neudesin
neo1						-0.44								Neogenin
nepro				-0.46		-0.67								
neu1				0.44										Sialidase-1
neurl1b	-0.47		-0.42	-0.41		-0.77								Protein neuralized

neurl4						0.53							K16777	Neuralized-like protein 4
neurod4						-0.83							K09079	Neurogenic differentiation factor 4
nexmif				-0.48		-0.75								Neurite extension and migration factor
nexn						-0.94							K23918	Nexilin
nf1				-0.39		-0.78							K08052	Neurofibromin 1
nfatc1			-0.57			-0.97							K04446	Nuclear factor of activated T-cells, cytoplasmic 1
nfatc2						-0.81							K17332	Nuclear factor of activated T-cells, cytoplasmic 2
nfib						0.63								
nfkbia	-0.44	-0.47	-0.42											NF-kappa-B inhibitor alpha
nfkbil1						0.66							K09256	NF-kappa-B inhibitor-like protein 1
nfxl1	0.83	0.66	0.86											NF-X1-type zinc finger protein NFXL1
nfya				-0.64		-0.80								Nuclear transcription factor Y, alpha
ngdn	0.72												K14765	U3 small nucleolar ribonucleoprotein protein LCP5
ngly1						-0.39								Peptide-N4-(N-acetyl-beta-glucosaminyl)asparagine amidase
nhlrc2						0.70								
nhlrc3	-0.36		-0.31											
nhp2	0.69	0.35	0.50											H/ACA ribonucleoprotein complex subunit 2
niban1			0.50											
nicn1						0.66							K16607	Nicolin-1
nid1				-0.63		-1.32								Nidogen (entactin)
ninj1						-0.50								
nip7						0.48							K07565	60S ribosome subunit biogenesis protein NIP7
nipal2						0.90								Magnesium transporter
nipal3								Loss	Loss			Loss		Magnesium transporter
nipbl						-0.34							K06672	Cohesin loading factor subunit SCC2
nkain1	-0.53		-0.57											Sodium/potassium-transporting atpase subunit beta-1-interacting protein
nkain2						-0.82								Sodium/potassium-transporting atpase subunit beta-1-interacting protein
nkap	-0.27												K25931	NF-kappa-B-activating protein
nkd1						-0.65								Naked cuticle
nkiras2						0.59								NF-kappa-B inhibitor-interacting Ras-like protein
nktr				0.59		0.77								NK-tumor recognition protein, peptidyl-prolyl isomerase G (cyclophilin G)

nkx1-1						-1.27								Homeobox protein Nkx-1
nkx3-2						0.65								Homeobox protein Nkx-3.2
nle1	0.54												K14855	Ribosome assembly protein 4
nlk						-0.76								Nemo like kinase
nmd3											Loss			60S ribosomal export protein NMD3
nme3	0.64		0.44											Nucleoside-diphosphate kinase
nme6						0.75								Nucleoside-diphosphate kinase
noa1	0.42												K19832	Nitric oxide-associated protein 1
nob1						0.37							K11883	RNA-binding protein NOB1
noc2l						0.55							K14833	Nucleolar complex protein 2
noc3l	0.44													Nucleolar complex protein 3
noc4l				-0.43										U3 small nucleolar RNA-associated protein 19
noct	-0.74		-0.46											Nocturnin
nod1						-0.56							K08727	Nucleotide-binding oligomerization domain-containing protein 1
nol10	0.43	0.33											K14788	Ribosome biogenesis protein ENP2
nol11						-0.40								
nol7	0.52													
nom1	0.52													Nucleolar MIF4G domain-containing protein 1
nop10	0.38			-0.57										H/ACA ribonucleoprotein complex subunit 3
nop14	0.60					0.45							K14766	Nucleolar protein 14
nop2	0.62													25S rrna (cytosine2870-C5)-methyltransferase
nop9	0.41					0.52								Nucleolar protein 9
nostrin	-0.62												K20126	Nostrin
noxo1				0.91										NADPH oxidase organizer 1
npl			-0.40	0.56		0.50								N-acetylneuraminate lyase
nploc4						0.62								Nuclear protein localization protein 4
npm3	0.53												K11278	Nucleophosmin 3
npnt						-0.50								Nephronectin
npr1						0.70								
nprl2	-0.89	-0.59	-0.95											Nitrogen permease regulator 2
nr1d1	-1.23		-1.41	-0.70		-0.60							K03728	Nuclear receptor subfamily 1 group D member 1
nr1d2	-0.43													Nuclear receptor subfamily 1 group D member 2
nr1h3						0.62							K08536	Liver X receptor alpha

nr2c2ap	0.48													
nr2f6			-0.50			0.62								Nuclear receptor subfamily 2 group F member 6
nr3c2	-0.50													Mineralocorticoid receptor
nr4a3				0.43									K08559	Nuclear receptor subfamily 4 group A member 3
nrbf2	-0.61	-0.30	-0.38											Nuclear receptor-binding factor 2
nrbp2	-0.31												K08875	Nuclear receptor-binding protein
nrde2	-0.40	-0.33	-0.59										K26109	Nuclear exosome regulator NRDE2
nrf1	0.33	0.33	0.33											Nuclear respiratory factor 1
nrip1			-0.51										K17965	Nuclear receptor-interacting protein 1
nrm	0.46												K21310	Methanethiol S-methyltransferase
nsa2						0.62							K14842	Ribosome biogenesis protein NSA2
nsd2	0.70	0.37	0.71			-0.67								
nsdhl			0.69										K07748	Sterol-4alpha-carboxylate 3-dehydrogenase (decarboxylating)
nsf												Loss	K06027	Vesicle-fusing atpase
nsfl1c						0.57								UBX domain-containing protein 1
nsmaf	-0.47		-0.41				Gain			Gain	Gain	Gain + Loss		Factor associated with neutral sphingomyelinase activation
nsrp1						0.38							K13206	Coiled-coil domain-containing protein 55
nsun2	0.73	0.64	0.70											Trna (cytosine34-C5)-methyltransferase
nsun6												Gain	K21971	Methyltransferase NSUN6
nt5c2	-1.23	-0.44	-0.75										K01081	5'-nucleotidase
nt5c3a	-1.05		-0.96										K24242	7-methylguanosine nucleotidase
nt5dc2	-0.60													
nt5m	0.43													
ntrk2						0.58							K04360	Neurotrophic tyrosine kinase receptor type 2
ntrk3			0.49											Neurotrophic tyrosine kinase, receptor type 3
nuak1											Loss	Gain		NUAK family, SNF1-like kinase
nub1	-1.42	-0.77	-1.52											
nubp2						-0.48								ATP-binding protein involved in chromosome partitioning
nucb1	0.48												K20371	Nucleobindin
nucks1	0.55	0.39	0.62											
nudc	0.39												K25866	Nuclear migration protein nudc
nudcd1	0.44													

nudcd2						0.82								
nudt12						0.44							K03426	NAD+ diphosphatase
nudt18						0.72							K17817	8-oxo-dgdp phosphatase
nudt19		0.37											K13355	Nucleoside diphosphate-linked moiety X motif 19, mitochondrial
nudt21	0.39		0.52										K14397	Cleavage and polyadenylation specificity factor subunit 5
nudt7							Loss							Peroxisomal coenzyme A diphosphatase NUDT7
nudt8	-0.33		-0.44											Nudix motif 8
numa1			0.59											Nuclear mitotic apparatus protein 1
numb		-0.33	-0.38										K06057	Numb
nup107			0.34											Nuclear pore complex protein Nup107
nup153	0.53		0.37	-0.40		-0.70							K14296	Nuclear pore complex protein Nup153
nup160	0.65		0.45										K14303	Nuclear pore complex protein Nup160
nup188	0.71	0.48	0.55											Nuclear pore complex protein Nup188
nup205	0.51	0.35	0.40										K14310	Nuclear pore complex protein Nup205
nup210	0.79		0.46	-0.58										Nuclear pore complex protein Nup210
nup214		0.37											K14317	Nuclear pore complex protein Nup214
nup35	0.47	0.34	0.69											Nuclear pore complex protein Nup53
nup37	0.47		0.49										K14302	Nuclear pore complex protein Nup37
nup43	0.49	0.44	0.60											Nuclear pore complex protein Nup43
nup50	0.48													Nuclear pore complex protein Nup50
nup58	0.54	0.40	0.58										K14307	Nucleoporin p58/p45
nup62	0.43					-0.76							K14306	Nuclear pore complex protein Nup62
nup85	0.30		0.32											Nuclear pore complex protein Nup85
nup93	0.65	0.35	0.74											Nuclear pore complex protein Nup93
nup98						-0.28								Nuclear pore complex protein Nup98-Nup96
nus1	0.46		0.42											Dehydrodolichyl diphosphate syntase complex subunit NUS1
nusap1			0.62											Nucleolar and spindle-associated protein 1
nxnl1								Gain					K17609	Nucleoredoxin
nxnl2						-0.84							K17609	Nucleoredoxin
nxph2						-0.54							K16657	Neurexophilin-2
naa25				-0.41		-0.82							K17973	N-terminal acetyltransferase B complex non-catalytic subunit
naa40	0.50	0.27	0.27									Gain		N-alpha-acetyltransferase 40
oaf						-0.41								

oat	-0.45													Ornithine--oxo-acid transaminase
oaz1	-0.38												K16548	Ornithine decarboxylase antizyme 1
obi1	0.76	0.48	0.83											ORC ubiquitin ligase 1
obscn						-0.89								Obscurin-rhogef
obsl1							Gain					Gain		Obscurin-like protein 1
ociad1	0.41		0.30			0.35								
odf2	0.44													Outer dense fiber protein 2
ofd1			0.52										K16480	Oral-facial-digital syndrome 1 protein
oga	-0.32	-0.40	-0.53										K15719	Protein O-glcnacase / histone acetyltransferase
ogdh						-0.46							K00164	2-oxoglutarate dehydrogenase E1 component
ogfod1	0.72		0.46										K24029	Prolyl 3-hydroxylase /prolyl 3,4-dihydroxylase
ogfod2						-1.06								
ogfod3			0.42						Loss					
ogg1						-0.33							K03660	N-glycosylase/DNA lyase
olfm3												Gain	K25446	Noelin-3
oma1				0.39									K23010	Metalloendopeptidase OMA1, mitochondrial
opn3					3.40								K04256	C-opsin
optn	-0.41													Optineurin
orc1	1.51	0.65	0.99											Origin recognition complex subunit 1
orc2	0.88		0.62										K02604, K21844	Origin recognition complex subunit 2, protein FAM126
orc3	0.76	0.46	0.78										K02605	Origin recognition complex subunit 3
orc4	0.79	0.56	0.84										K02606	Origin recognition complex subunit 4
orc5	1.01	0.89	1.09										K02607	Origin recognition complex subunit 5
orc6	0.49													Origin recognition complex subunit 6
osbpl9						-0.55							K20465	Oxysterol-binding protein-related protein 9/10/11
oscp1	0.53		0.48											
oser1	-0.40		-0.45											
osgepl1						0.46							K01409	N6-L-threonylcarbamoyladenine synthase
ostc			0.40											
ostf1						-0.55								
ostm1						0.29							K23863	Osteopetrosis-associated transmembrane protein 1 (chloride channel 7 beta subunit)
ostn						-0.72								Osteocrin

otor	-0.55												K25714	Otoraplin
otub1			0.39											Ubiquitin thioesterase protein OTUB1
otub2		-0.29											K09603	Ubiquitin thioesterase protein OTUB2
otud1	-0.51	-1.58	-0.53										K13716	OTU domain-containing protein 1
otud4				0.36										OTU domain-containing protein 4
otud5	-0.53	-0.33	-0.59										K12655	OTU domain-containing protein 5
otx1											Gain		K09326	Homeobox protein OTX1
oxct1				-0.43		-0.81							K01027, K01028	3-oxoacid coa-transferase , 3-oxoacid coa-transferase subunit A
oxnad1	0.67													
oxr1	-1.29	-0.49	-1.04										K25437	Oxidation resistance protein 1
oxsm			0.44			0.52							K09458	3-oxoacyl-
oxsr1								Gain					K08835	Serine/threonine-protein kinase OSR1/STK39
oxtr						-1.00								Oxytocin receptor
p2rx2						0.95							K05216	P2X purinoceptor 2
p2rx5									Loss					P2X purinoceptor 5
p2ry6	-0.67		-0.37										K04272	P2Y purinoceptor 6
p3h1	0.82	0.41	0.73											Procollagen-proline 3-dioxygenase 1
p3h3	0.36		0.47											Procollagen-proline 3-dioxygenase 3
p3h4	0.55	0.42	0.62										K22461	Endoplasmic reticulum protein SC65
p4ha1	0.70	0.92	0.95	-0.46									K00472	Prolyl 4-hydroxylase
p4hb	0.42		0.57											Protein disulfide-isomerase A1
pabpn1	0.90		0.60											Polyadenylate-binding protein 2
pacsin2						-0.54							K20123	Protein kinase C and casein kinase substrate in neurons protein
padi2			0.51										K01481	Protein-arginine deiminase
paf1			0.40			0.53								RNA polymerase II-associated factor 1
pah						-0.78								Phenylalanine-4-hydroxylase
paip1	-0.52					-1.10							K14322	Polyadenylate-binding protein-interacting protein 1
paip2	-2.08	-1.06	-2.30											
pak1	0.54		0.46										K04409	P21-activated kinase 1
pak1ip1	0.39													Protein MAK11
pak2	-0.64	-0.30	-0.43											P21-activated kinase 2
palb2	0.65		0.53											Partner and localizer of BRCA2

pald1						-0.35								
pam	-0.44	-0.38	-0.43			-0.29							K24006	Peptidylglycine monooxygenase / peptidylamidoglycolate lyase
pam16	0.76													Mitochondrial import inner membrane translocase subunit TIM16
pank4	-0.42		-0.41											Bifunctional damage-control phosphatase, subfamily II, fusion protein, type II pantothenate kinase
panx3						-0.59						Loss		Homeobox protein PKNOX, pannexin 2/3
paox						-0.69								N1-acetylpolyamine oxidase
pappa												Gain		Pappalysin-1
papss1	0.45	0.31	0.32											3'-phosphoadenosine 5'-phosphosulfate synthase
pard6g						-0.87							K06093	Partitioning defective protein 6
parn	0.62		0.31											Poly(A)-specific ribonuclease
parp1	0.40	0.37	0.41											Poly
parp16	-0.32					-0.22						Loss	K00774	Poly
parp2	0.38		0.45										K10798	Poly
parp4	-0.44												K10798	Poly
parp8			-0.33											Poly
parpbp			0.44											
pask						-0.51							K08801	PAS domain containing serine/threonine kinase
patz1						0.75								POZ-AT hook zinc finger protein 1
pawr			0.51											
pax2						-0.52								Paired box protein 2
pax6								Gain	Gain				K08031	Paired box protein 6
pax7						0.75								Paired box protein 3/7
pax9						0.59							K09382	Paired box protein 1/9
paxip1						-0.47							K14972	PAX-interacting protein 1
pbk			0.46											PDZ-binding kinase
pbx1		0.38												
pc	0.41												K01958	Pyruvate carboxylase
pcbd2	-0.57													4a-hydroxytetrahydrobiopterin dehydratase
pcca	0.41												K01965	Propionyl-coa carboxylase alpha chain
pccb						-0.45							K01966	Propionyl-coa carboxylase beta chain
pcdh20						-0.59								Protocadherin delta 1
pcdh8						-1.06						Gain		Protocadherin delta 2

pcf11	-0.36													Pre-mrna cleavage complex 2 protein Pcf11
pcgf2	0.34													Polycomb group RING finger protein 4
pcgf3						0.61							K11488	Polycomb group RING finger protein 3
pcgf6	0.62		0.50				Gain						K11470	Polycomb group RING finger protein 6
pcid2	-0.68													Nuclear mrna export protein PCID2/THP1
pcif1	-0.52		-0.58										K17584	Phosphorylated CTD-interacting factor 1
pclaf	0.57													
pcm1				0.54										Pericentriolar material 1 protein
pcmtd1	-1.97	-0.57	-2.06											Protein-L-isoaspartate(D-aspartate) O-methyltransferase
pcna	1.03	0.74	0.92											Proliferating cell nuclear antigen
pcnx4						-0.61								
pcsk2						0.57							K01360	Proprotein convertase subtilisin/kexin type 2
pcsk6				-0.43		-1.05							K08672	Proprotein convertase subtilisin/kexin type 6
pcyox1l						0.71							K05906	Prenylcysteine oxidase / farnesylcysteine lyase
pdcd11	0.56													Rrna biogenesis protein RRP5
pdcd4	-1.88	-0.62	-1.29											Programmed cell death protein 4
pdcd6ip						-0.51								Programmed cell death 6-interacting protein
pdcd7	0.57	0.34	0.67			-0.38								
pdcl				-0.43		-0.64								
pdcl3						0.40								
pde12	-0.37													2',5'-phosphodiesterase
pde1b						-0.55	Gain	Gain	Gain				K13755	Calcium/calmodulin-dependent 3',5'-cyclic nucleotide phosphodiesterase
pde3b	-0.55					0.65								Cgmp-inhibited 3',5'-cyclic phosphodiesterase B
pde4dip	-1.15	-0.42	-0.59			-0.82			Gain					Myomegalin
pde5a	-0.53		-0.48								Loss	Loss	K13762	Cgmp-specific 3',5'-cyclic phosphodiesterase
pde7a						-0.34								
pde7b						0.74							K18436	High affinity camp-specific 3',5'-cyclic phosphodiesterase 7
pde9a						-0.49								High affinity cgmp-specific 3',5'-cyclic phosphodiesterase 9
pdf	0.48												K01462	Peptide deformylase
pdgfc	0.84	0.70	1.03										K05450	Platelet derived growth factor C/D
pdia3			0.56										K08056	Protein disulfide-isomerase A3
pdia4	0.83		0.80										K09582	Protein disulfide-isomerase A4

pdia5			0.53											Protein disulfide-isomerase A5
pdia6	0.48	0.56	0.53			0.55								Protein disulfide-isomerase A6
pdk3						0.62							K00898	Pyruvate dehydrogenase kinase 2/3/4
pdk4			-0.61											Pyruvate dehydrogenase kinase 2/3/4
pdlim2						-0.45								PDZ and LIM domain protein 1/2/3/4
pdlim4						-0.53							K23353	PDZ and LIM domain protein 1/2/3/4
pdlim7						0.76							K19867	PDZ and LIM domain protein 5/6/7
pdp1						0.57							K01102	Pyruvate dehydrogenase phosphatase
pdss1						0.58								Decaprenyl-diphosphate synthase subunit 1
pdss2				-0.38										Decaprenyl-diphosphate synthase subunit 2
pdxp	-0.40		-0.45											Pyridoxal phosphatase
pdzd11						-0.85								PDZ domain-containing protein 11
pdzd3				1.14										PDZ domain-containing protein 3
pdzd8				0.35										PDZ domain-containing protein 8
pdzrn3				-0.33									K15682	Ligand of Numb protein X 3/4
pebp1				-0.44									K06910	Phosphatidylethanolamine-binding protein
pecr						0.58								Peroxisomal trans-2-enoyl-coa reductase
peli1						-0.59								Pellino
pelo	0.45													Protein pelota
pelp1		0.41	0.51											Proline-, glutamic acid- and leucine-rich protein 1
pepd											Loss	Loss	K14213	Xaa-Pro dipeptidase
pes1	0.35													Pescadillo
pex1				0.52		0.47								Peroxin-1
pex10	0.69	0.37	0.64										K13346	Peroxin-10
pex11g						-0.88							K13353	Peroxin-11C
pex13	-0.41					-0.80								Peroxin-13
pex3				-0.35		-0.59								Peroxin-3
pfas	0.67	0.46	0.50											Phosphoribosylformylglycinamidine synthase
pfkfb1			0.34	0.50		0.43							K19028	6-phosphofructo-2-kinase / fructose-2,6-biphosphatase 1
pfkfb3		0.37											K01103	6-phosphofructo-2-kinase / fructose-2,6-biphosphatase 3
pgam5	0.41		0.56										K15637	Serine/threonine-protein phosphatase PGAM5
pgd			0.45											6-phosphogluconate dehydrogenase
pggt1b	0.27													Geranylgeranyl transferase type-1 subunit beta

pgm2l1	0.57					0.46								Glucose-1,6-bisphosphate synthase
pgpep1	-1.39		-1.63											Pyroglutamyl-peptidase
phax	0.49		0.46											Phosphorylated adapter RNA export protein
phb2	0.35													Prohibitin 2
phc1		0.33												Polyhomeotic-like protein 1
phex						0.84							K08636	Phosphate-regulating neutral endopeptidase
phf10			0.39			-0.76								BRG1-associated factor 45A
phf14		0.29												
phf19	0.39		0.47			-0.37								PHD finger protein 19
phf21a				0.39									K24651	PHD finger protein 21A
phf3	-0.40					-0.76								
phf5a	0.40		0.42	-0.50										PHD finger-like domain-containing protein 5A
phf8				-0.32		-0.56								
phgdh	0.69		0.57											D-3-phosphoglycerate dehydrogenase / 2-oxoglutarate reductase
phip						0.51								PH-interacting protein
phka1						-0.65							K07190	Phosphorylase kinase alpha/beta subunit
phka2	-1.22		-1.14										K07190	Phosphorylase kinase alpha/beta subunit
phlda3			-0.58										K23793	Pleckstrin homology-like domain family A
phldb1						0.52	Gain						K23794	Pleckstrin homology-like domain family B
phospho2	0.63		0.39											Pyridoxal phosphate phosphatase PHOSPHO2
phrf1						0.63	Loss		Loss					PHD and RING finger domain-containing protein 1
phyh						-0.57							K00477	Phytanoyl-coa hydroxylase
pi15						0.67								
pi4k2b						0.60							K13711	Phosphatidylinositol 4-kinase type 2
pibf1						0.42							K16538	Progesterone-induced-blocking factor 1
pick1						-0.36								PRKCA-binding protein
piezo1	-0.34													Piezo-type mechanosensitive ion channel component 1/2
piezo2						0.59								Piezo-type mechanosensitive ion channel component 1/2
pif1			0.50			-0.87								ATP-dependent DNA helicase PIF1
pigb							Loss		Loss					GPI mannosyltransferase 3
pigf	0.47		0.38										K05287	GPI ethanolamine phosphate transferase 2/3 subunit F
pigg								Loss						Ethanolamine phosphate transferase 2 subunit G
pigh				-0.38		-0.46								Phosphatidylinositol N-acetylglucosaminyltransferase subunit H

pigo	1.03		0.61											GPI ethanolamine phosphate transferase 3 subunit O
pigq	0.39												K03860	Phosphatidylinositol N-acetylglucosaminyltransferase subunit Q
pigu				-0.37		-0.61							K05293	GPI-anchor transamidase subunit U
pigv			0.35											GPI mannosyltransferase 2
pigw											Loss		K05283	Glucosaminylphosphatidylinositol acyltransferase
pigx	-0.39												K07541	GPI mannosyltransferase 1 subunit X
pih1d2						0.83							K25427	PIH1 domain-containing protein 2
pik3ap1				0.47									K12230	Phosphoinositide 3-kinase adapter protein 1
pik3c2a	0.32	0.30	0.42	0.43		0.44								Phosphatidylinositol-4-phosphate 3-kinase
pik3c3	-0.83	-0.49	-0.62											Phosphatidylinositol 3-kinase
pik3cd				0.43									K00922	Phosphatidylinositol-4,5-bisphosphate 3-kinase catalytic subunit alpha/beta/delta
pik3cg	-0.43		-0.37			-0.71								Phosphatidylinositol-4,5-bisphosphate 3-kinase catalytic subunit gamma
pik3ip1	-1.35	-0.97	-1.21											
pink1	-0.93		-0.81			-0.38							K05688	PTEN induced putative kinase 1
pip4k2b	0.45					-0.46							K00920	1-phosphatidylinositol-5-phosphate 4-kinase
pip4k2c						-0.41								1-phosphatidylinositol-5-phosphate 4-kinase
pip4p2	-0.62	-0.39	-0.77											Phosphatidylinositol-4,5-bisphosphate 4-phosphatase
pisd				-0.44		-0.65							K01613	Phosphatidylserine decarboxylase
pitpnc1				-0.43		-1.21								
pitrm1	1.15												K06972	Presequence protease
pkd2				-0.43		-0.55								Polycystin 2
pkdcc	-0.87	-0.57												Extracellular tyrosine-protein kinase PKDCC
pkib				-0.45										
pkmyt1	0.39	0.39	0.45										K06633	Membrane-associated tyrosine- and threonine-specific cdc2-inhibitory kinase
pkn2	0.46		0.34										K23691	Serine/threonine-protein kinase N2
pknox2				-0.43		-0.96							K24891	Homeobox protein PKNOX
pkp2							Loss		Gain	Loss				Plakophilin 2
pkp4						-0.44							K23491	Catenin delta-2
pla2g15	-0.75		-0.69											Lysophospholipase III
pla2g6	-0.93	-0.45	-0.94	0.31									K16343	Calcium-independent phospholipase A2
pla2r1											Gain	Gain	K06560	Mannose receptor, C type

plag1	-0.58		-0.55											General transcription factor IIIA, zinc finger protein PLAG1
plagl2				-0.41		-0.90							K19486	Zinc finger protein PLAGL2
plbd2				0.42		0.42								
plcb1				0.42		0.81								Phosphatidylinositol phospholipase C, beta
plcb3				0.48										Phosphatidylinositol phospholipase C, beta
plcb4				0.44		0.72							K05858	Phosphatidylinositol phospholipase C, beta
plcd1				0.47									K05857	Phosphatidylinositol phospholipase C, delta
plcd4						0.69								Phosphatidylinositol phospholipase C, delta
plcg2				0.59									K05859	Phosphatidylinositol phospholipase C, gamma-2
plch1											Loss	Loss	K19006	Phosphatidylinositol phospholipase C, eta
plcxd3							Loss							
pld1				0.56							Loss		K01115	Phospholipase D1/2
pld3	-0.60		-0.32											Phospholipase D3/4
plek		-0.41	-0.53											Pleckstrin
plekha3	-0.71	-0.67	-0.91											Pleckstrin homology domain-containing family A member 3
plekha5						-0.73							K23797	Pleckstrin homology domain-containing family A member 4/5/6/7
plekhb2	-0.86	-0.44	-0.91										K23857	Pleckstrin homology domain-containing family B
plekhf1			-0.45									Loss	K23858	Pleckstrin homology domain-containing family F
plekhf2						-0.43							K23858	Pleckstrin homology domain-containing family F
plekhg4b						-0.51								Pleckstrin homology domain-containing family G member 4
plekhm1				0.34										Pleckstrin homology domain-containing family M member 1
plekhm3	-0.45					-0.52							K23282	Pleckstrin homology domain-containing family M member 1
plgrkt	-1.21													
plin2												Loss		
plk1			0.55			-0.63							K06631	Polo-like kinase 1
plk3	0.42													Polo-like kinase 3
plk4	0.53												K08863	Polo-like kinase 4
plod1		0.54	0.63											Procollagen-lysine,2-oxoglutarate 5-dioxygenase 1
plod2						0.49								Procollagen-lysine,2-oxoglutarate 5-dioxygenase 2
plp1						-0.69								Myelin proteolipid protein
plpp1				0.49										Phosphatidate phosphatase
plpp7				-0.78		-1.04							K22904	Presqualene diphosphate phosphatase
plrg1	0.37		0.42					Loss					K12862	Pleiotropic regulator 1

plvap				-0.51		-1.00							K17309	Plasmalemma vesicle-associated protein
plxna1									Loss					Plexin A
plxna3	0.38					0.41							K06820	Plexin A
plxnd1						-0.35							K06822	Plexin D
pmp22	-0.98	-0.37	-0.79											Peripheral myelin protein 22
pmpca								Loss			Loss			Mitochondrial-processing peptidase subunit alpha
pmpcb	0.37												K17732	Mitochondrial-processing peptidase subunit beta
pms2			0.39										K10858, K25458	DNA mismatch repair protein PMS2, radial spoke head protein 10B
pnkp						-0.48								Bifunctional polynucleotide phosphatase/kinase
pnn	0.41		0.43											Pinin
pnpla4							Loss	Loss					K11157	Patatin-like phospholipase domain-containing protein 4
poc1a				-0.46		-0.67							K16482	Centriolar protein POC1
podn						0.63								Podocan
pogk							Loss					Gain		
poglut2	0.80	0.41	0.72											EGF-domain serine glucosyl/xylosyltransferase
poglut3	0.37		0.47										K13667	EGF-domain serine glucosyl/xylosyltransferase
pogz	1.15	0.56	1.23										K22594	Pogo transposable element with ZNF domain
pola2	0.66	0.71	0.65										K02321	DNA polymerase alpha subunit B
pold1	0.49													DNA polymerase delta subunit 1
pold2	0.83	0.58	0.72											DNA polymerase delta subunit 2
pold3				0.44										DNA polymerase delta subunit 3
pold4	-0.53					-0.45							K03505	DNA polymerase delta subunit 4
poldip3	0.34													Polymerase delta-interacting protein 3
pole	0.82	0.87	0.84										K02324	DNA polymerase epsilon subunit 1
pole2	0.49	0.45	0.56											DNA polymerase epsilon subunit 2
pole3	0.74	0.40												DNA polymerase epsilon subunit 3
pole4	0.50		0.51	-0.44		-0.41							K03506	DNA polymerase epsilon subunit 4
polg						0.63							K02332	DNA polymerase gamma 1
polg2				-0.40										DNA polymerase gamma 2
poli	-0.63		-0.47											DNA polymerase iota
polm		-0.32	-0.32										K03513	DNA polymerase mu
polq												Gain	K02349	DNA polymerase theta

polr1b						0.69								DNA-directed RNA polymerase I subunit RPA2
polr1c	0.40					0.38								DNA-directed RNA polymerases I and III subunit RPAC1
polr1d	-0.59	-0.54	-0.82											
polr1e			-0.43											DNA-directed RNA polymerase I subunit RPA49
polr2c	0.24		0.29										K03011	DNA-directed RNA polymerase II subunit RPB3
polr2d			0.48										K03012	DNA-directed RNA polymerase II subunit RPB4
polr2f				-0.31									K03014	DNA-directed RNA polymerases I, II, and III subunit RPABC2
polr2g	0.39													DNA-directed RNA polymerase II subunit RPB7
polr2h	0.89		0.63											DNA-directed RNA polymerases I, II, and III subunit RPABC3
polr2m				-0.37									K21987	DNA-directed RNA polymerase II subunit GRINL1A
polr3a	0.37					0.37							K03018	DNA-directed RNA polymerase III subunit RPC1
polr3d	0.37													DNA-directed RNA polymerase III subunit RPC4
polr3e	0.56													DNA-directed RNA polymerase III subunit RPC5
polr3g	0.72													DNA-directed RNA polymerase III subunit RPC7
pom121	0.36												K14316	Nuclear pore complex protein Nup121
pomgnt1						0.78								Beta-1,2-N-acetylglucosaminyltransferase
pomgnt2	0.43												K18207	Protein O-mannose beta-1,4-N-acetylglucosaminyltransferase
pomt1	0.49												K00728	Dolichyl-phosphate-mannose-protein mannosyltransferase
pop1	0.38					0.35							K01164	Ribonuclease P/MRP protein subunit POP1
pop4			-0.56											Ribonuclease P protein subunit POP4
pop5	0.38												K03537	Ribonuclease P/MRP protein subunit POP5
pop7						-0.55							K14527	Ribonuclease P/MRP protein subunit RPP20
postn						-0.43								Periostin
pou2f1							Loss				Gain	Gain		POU domain transcription factor, class 2
pou3f3						-0.62							K09365	POU domain transcription factor, class 3
pou3f4						-0.78								POU domain transcription factor, class 3
pou6f1						-0.82							K09368	POU domain transcription factor, class 6
ppa2				-0.39			Gain	Gain	Gain		Loss	Gain + Loss	K01507	Inorganic pyrophosphatase
ppan	0.36												K14859	Ribosome biogenesis protein SSF1/2
ppard		0.32												Peroxisome proliferator-activated receptor delta
ppargc1b				-0.51		-0.70								Peroxisome proliferator-activated receptor gamma coactivator 1-beta
ppfia1						0.84								

ppfibp1	-0.31	-0.40	-0.44											
ppfibp2	-0.41		-0.57											
pphln1				-0.44		-1.01							K21872	Periphilin-1
ppib	0.38		0.61			0.49							K03768	Peptidyl-prolyl cis-trans isomerase B (cyclophilin B)
ppid						0.58							K05864	Peptidyl-prolyl isomerase D
ppie	0.59		0.52											Peptidyl-prolyl isomerase E (cyclophilin E)
ppig	1.16	0.86	1.03											Peptidyl-prolyl isomerase G (cyclophilin G)
ppil1	0.37		0.51										K12733	Peptidyl-prolyl cis-trans isomerase-like 1
ppm1b	-0.37		-0.30											
ppm1d						-0.48								Protein phosphatase 1D
ppm1g	0.33	0.32												Protein phosphatase 1G
ppm1h			-0.42										K17503	Protein phosphatase 1H
ppm1k			-0.63											Protein phosphatase 1K
ppp1r10	-0.56													Protein phosphatase 1 regulatory subunit 10
ppp1r13b	-0.41												K17554	Apoptosis-stimulating of p53 protein 1
ppp1r14b				-0.45		-0.89							K17555	Protein phosphatase 1 regulatory subunit 14B
ppp1r14c			-0.38										K17556	Protein phosphatase 1 regulatory subunit 14C
ppp1r16b				-0.43		-0.63								Protein phosphatase 1 regulatory inhibitor subunit 16B
ppp1r1b				0.40										Protein phosphatase 1 regulatory subunit 1B
ppp1r1c						-0.64								Protein phosphatase 1 regulatory subunit 1C
ppp1r37												Gain	K17576	Protein phosphatase 1 regulatory subunit 37
ppp1r3c						-0.50								Protein phosphatase 1 regulatory subunit 3A/B/C/D/E
ppp1r3d	-0.63					-0.70								Protein phosphatase 1 regulatory subunit 3A/B/C/D/E
ppp4c	0.32	0.33	0.54											Serine/threonine-protein phosphatase 4 catalytic subunit
ppp4r2			0.41										K15425	Serine/threonine-protein phosphatase 4 regulatory subunit 2
ppp4r3b						0.51							K17491	Protein phosphatase 4 regulatory subunit 3
ppp5c	0.96	0.65	0.80			0.55							K04460	Serine/threonine-protein phosphatase 5
pprc1	0.48			-0.43		-0.77							K17963	Peroxisome proliferator-activated receptor gamma coactivator-related protein 1
pptc7						0.58							K17508	Protein phosphatase PTC7
ppwd1	0.45	0.35	0.63										K12736	Peptidylprolyl isomerase domain and WD repeat-containing protein 1
pqbp1	0.26					0.30							K12865	Polyglutamine-binding protein 1
pradc1	0.43			-0.40									K25720	Protease-associated domain-containing protein 1

prc1			0.47										K16732	Ase1/PRC1/MAP65 family protein
prcp			-0.29											Lysosomal Pro-X carboxypeptidase
prdm10						-0.84								PR domain zinc finger protein 10
prdm11	0.47					0.54							K24644	PR domain zinc finger protein 11
prdm15	0.55												K09191, K24647	General transcription factor IIIA, PR domain zinc finger protein 15
prdm8						-0.65								PR domain zinc finger protein 8
prdx3	0.39												K03386, K20011	Peroxiredoxin 2/4 , peroxiredoxin 3
prdx4			0.30											Peroxiredoxin 2/4
prelid1	0.45													
prep	0.47													Prolyl oligopeptidase
prex2						-0.48							K17588	Phosphatidylinositol 3,4,5-trisphosphate-dependent Rac exchanger 2 protein
prg4						0.86						Loss		
prickle2	-0.91		-0.58										K04511	Prickle
prickle3						-0.83								Prickle
prim1	0.67		0.53										K02684	DNA primase small subunit
prim2	0.77	0.67	0.61										K02685	DNA primase large subunit
prima1		-0.48												
prkar1a			-0.38											Camp-dependent protein kinase regulator
prkca	-0.31													Classical protein kinase C alpha type
prkcb							Loss				Loss	Gain + Loss		Classical protein kinase C beta type
prkch				0.42										Novel protein kinase C eta type
prkcq			-0.44										K18052	Novel protein kinase C theta type
prkcsh	0.53	0.34	0.63										K08288	Protein kinase C substrate 80K-H
prkd1						0.74							K06070	Protein kinase D
prkn						0.56							K04556	Parkin
prkra				-0.40		-0.85							K24540	Interferon-inducible double-stranded RNA-dependent protein kinase activator A
prkrip1	0.62		0.45										K26159	PRKR-interacting protein 1
prkaa2						0.41								5'-AMP-activated protein kinase, catalytic alpha subunit
prmt3	0.57													Type I protein arginine methyltransferase
prmt5	0.66	0.40	0.48											Type II protein arginine methyltransferase

prmt7				-0.53		-0.76								Type III protein arginine methyltransferase
prmt9	0.78	0.43	0.65										K19737	Type II protein arginine methyltransferase
prorp	0.62		0.37											Mitochondrial ribonuclease P protein 3
pros1						0.77							K03908	Protein S
prpf18	-0.24													Pre-mrna-splicing factor 18
prpf19	0.92	0.54	0.83										K10599	Pre-mrna-processing factor 19
prpf31	0.60		0.40										K12844	U4/U6 small nuclear ribonucleoprotein PRP31
prpf38b			0.28										K12850	Pre-mrna-splicing factor 38B
prpf40a	0.73	0.57	0.79										K12821	Pre-mrna-processing factor 40
prpf4b	0.38		0.38										K08827	Serine/threonine-protein kinase PRP4
prpf8	0.44	0.33	0.48										K12856	Pre-mrna-processing factor 8
prr13						-0.45								
prrc1	0.51	0.59	0.80											
prrc2c	0.62	0.55	0.44											
prrg4												Loss		
prrt4							Gain				Loss	Loss		
prrx1						-0.69							K09329	Paired mesoderm homeobox protein
prss12						-0.78							K09624	Neurotrypsin
prtg	1.64													Immunoglobulin superfamily DCC subclass member 3, immunoglobulin superfamily DCC subclass member 4
prune1	-0.45		-0.56											Exopolyphosphatase
psat1	0.54													Phosphoserine aminotransferase
psen2	-0.45		-0.30										K04505	Presenilin 1
pskh1				-0.41		-0.80								Protein serine kinase H
psma5						0.62								20S proteasome subunit alpha 5
psma6			0.44			0.44							K02730	20S proteasome subunit alpha 1
psmb5											Loss	Loss		20S proteasome subunit beta 5
psmc1						0.56								26S proteasome regulatory subunit T2
psmc2						0.43							K03061	26S proteasome regulatory subunit T1
psmc6						0.58								26S proteasome regulatory subunit T4
psmd1		0.32	0.41											26S proteasome regulatory subunit N2
psmd10						0.49								26S proteasome non-atpase regulatory subunit 10
psmd12						0.65							K03035	26S proteasome regulatory subunit N5

psmd2						0.57								26S proteasome regulatory subunit N1
psmd5	-0.36												K06692	26S proteasome non-atpase regulatory subunit 5
psmd6			0.36			0.40							K03037	26S proteasome regulatory subunit N7
psmd7						0.38							K03038	26S proteasome regulatory subunit N8
psmd8	0.37	0.31	0.42			0.40								26S proteasome regulatory subunit N12
psmd9						0.34								26S proteasome regulatory subunit N4
psme3	0.64													Proteasome activator subunit 3 (PA28 gamma)
psmg1			0.32											Proteasome assembly chaperone 1
psmg3						0.76							K11877	Proteasome assembly chaperone 3
pspc1	0.32													Paraspeckle component 1
psph	0.75												K01079	Phosphoserine phosphatase
pstpip1	-0.44		-0.88					Loss				Loss		Proline-serine-threonine phosphatase interacting protein 1
pstpip2	0.56													Proline-serine-threonine phosphatase interacting protein 2
ptcd3	0.40												K17659	Pentatricopeptide repeat domain-containing protein 3
ptges3	0.42													Cytosolic prostaglandin-E synthase
ptgfrn						0.36								Prostaglandin F2 receptor negative regulator
ptgr2						0.97								Prostaglandin reductase 2
pth1r						0.60								Parathyroid hormone receptor 1
pth2						-0.56							K23143	Tuberoinfundibular peptide of 39 residues
ptk2b						-0.52							K05871	Focal adhesion kinase 2
ptk7	0.45													PTK7 protein tyrosine kinase 7
ptpa	0.29													Serine/threonine-protein phosphatase 2A activator
ptpmt1	0.38													
ptpn11						-0.54						Gain	K07293	Tyrosine-protein phosphatase non-receptor type 11
ptpn13				0.43										FERM and PDZ domain-containing protein 2, tyrosine-protein phosphatase non-receptor type 13
ptpn2	-0.48		-0.63			-0.41								Tyrosine-protein phosphatase non-receptor type 2
ptpn9						0.69							K18038	Tyrosine-protein phosphatase non-receptor type 9
ptprc		-0.87												Receptor-type tyrosine-protein phosphatase C
ptprh			-0.45											Receptor-type tyrosine-protein phosphatase H
ptrh1						0.67								Peptidyl-trna hydrolase, PTH1 family
ptrh2	0.51												K04794	Peptidyl-trna hydrolase, PTH2 family
ptx3						-0.66							K25724	Pentraxin-related protein PTX3

pudp											Gain	Gain	K17623	Pseudouridine 5'-phosphatase
puf60			0.33			0.31								Poly(U)-binding-splicing factor PUF60
pum2				-0.42		-0.86							K17943	Pumilio RNA-binding family
pum3	0.72													Pumilio homology domain family member 6
pus1	0.52					0.38								Trna pseudouridine38-40 synthase
pus10	0.63													Trna pseudouridine synthase 10
pus3						0.58							K01855	Trna pseudouridine38/39 synthase
pus7l						0.43								Trna pseudouridine13 synthase
pwp1	0.95		0.46			0.48							K14791	Periodic tryptophan protein 1
pxdc1	-0.33		-0.32			-0.35								
pxdn	0.42													Peroxidase
pxk	-1.16	-0.41	-0.65											PX domain-containing protein kinase-like protein
pxmp2						0.83							K13347	Peroxisomal membrane protein 2
pxmp4	0.51		0.38										K13350	Peroxisomal membrane protein 4
pxylp1	0.47		0.41										K21403	2-phosphoxylose phosphatase
pygl					0.86	0.59								Glycogen phosphorylase
pym1						-0.47							K14294	Partner of Y14 and mago
pyroxd1				0.39										Pyridine nucleotide-disulfide oxidoreductase domain-containing protein 1
qsox1						0.88							K10758	Thiol oxidase
qsox2						-0.54								Thiol oxidase
qtrt2						0.71								Queuine trna-ribosyltransferase accessory subunit
r3hdm1						-0.38								
r3hdm4	-0.49													
rab10	0.34													Ras-related protein Rab-10
rab11fip5						-0.78							K12484	Rab11 family-interacting protein 1/2/5
rab12			-0.33											Ras-related protein Rab-12
rab18						0.59								Ras-related protein Rab-18
rab22a	-0.59		-0.69											Ras-related protein Rab-22
rab24	-0.33		-0.37										K07912	Ras-related protein Rab-24
rab32						0.40								Ras-related protein Rab-32
rab33b				-0.42		-0.57								Ras-related protein Rab-33B
rab34				-0.47		-0.70							K07921	Ras-related protein Rab-34

rab3gap1	-0.48					-0.71							K18270	Rab3 gtpase-activating protein catalytic subunit
rab3gap2						0.38							K19937	Rab3 gtpase-activating protein non-catalytic subunit
rab40c	-0.29													Ras-related protein Rab-40
rab4a						-0.57							K07879, K07880	Ras-related protein Rab-4A, Ras-related protein Rab-4B
rab5c				-0.34		-0.75								Ras-related protein Rab-5C
rab9a	-0.34	-0.35	-0.48			-0.34							K07899	Ras-related protein Rab-9A
rabac1						-0.63							K20359	PRA1 family protein 1
rabep1						0.63								Rab gtpase-binding effector protein 1
rabgap1	-0.29		-0.36											Rab gtpase-activating protein 1
rabgef1	-0.54		-0.67										K20131, K21917	BTB/POZ domain-containing protein KCTD7/14, Rab5 GDP/GTP exchange factor
rabggtb	0.38													Geranylgeranyl transferase type-2 subunit beta
rabl6												Loss		
rad1						0.51							K02830	Cell cycle checkpoint protein
rad21			0.23											Cohesin complex subunit SCC1
rad50						0.65							K10866	DNA repair protein RAD50
rad51	0.60		0.51											DNA repair protein RAD51
rad51b	-0.34	-0.46	-0.33											DNA-repair protein XRCC3, RAD51-like protein 1
rad54b	0.55												K10875, K10877	DNA repair and recombination protein RAD54 and RAD54-like protein , DNA repair and recombination protein RAD54B
rad9a	0.46	0.35	0.77	0.45										Cell cycle checkpoint control protein RAD9A
radil						-0.78								
rae1	0.46		0.35										K14298	Mrna export factor
raf1	-0.36					-0.25								RAF proto-oncogene serine/threonine-protein kinase
ralbp1				-0.41		-1.12							K08773	Rala-binding protein 1
ralgapa1						0.54							K25768	Ral gtpase-activating protein subunit alpha
ralgapa2				0.38										Ral gtpase-activating protein subunit alpha
ralgps1						0.92							K24023	Ras-specific guanine nucleotide-releasing factor ralgps
ralyl											Loss	Loss	K24994	RNA-binding Raly-like protein
ramac						0.57							K18708	RNMT-activating mini protein
ramp2	-0.42		-0.39			0.35							K08448	Receptor activity modifying protein 2
ran			0.41										K07936	GTP-binding nuclear protein Ran
ranbp1	0.78	0.50	0.69											Ran-binding protein 1

ranbp3	0.43	0.41	0.52										K15304	Ran-binding protein 3
rangap1	0.43		0.46											Ran gtpase-activating protein 1
rap1gap2			-0.35			0.41							K17708	RAP1 gtpase activating protein 2
rap1gds1							Gain							
rap2b						-0.60							K07838, K07839	Ras-related protein Rap-2B, Ras-related protein Rap-2C
rapgef1				0.41									K06277	Rap guanine nucleotide exchange factor 1
rapgef6			-0.26											Rap guanine nucleotide exchange factor 6
rapsn				-0.56		-0.99							K24924	Receptor-associated protein of the synapse
rara	-0.39												K08527	Retinoic acid receptor alpha
rasa1				0.32									K04352	Ras gtpase-activating protein 1
rasd1	-0.49													RAS, dexamethasone-induced Ras-related protein 1
rasef												Loss		Ras and EF-hand domain-containing protein
rasgrp2	-0.44					-0.37								RAS guanyl-releasing protein 2
rasgrp3	-0.99	-0.66	-1.10											RAS guanyl-releasing protein 3
rasl11b	0.71												K07853	Ras-like protein family member 11B
rassf1				-0.52										Ras association domain-containing protein 1
rassf2						-0.41								Ras association domain-containing protein 2/4
rb1		0.42											K06618	Retinoblastoma-associated protein
rb1cc1	-0.70	-0.39	-0.61											RB1-inducible coiled-coil protein 1
rbbp4	0.60	0.61	0.77	-0.34										Histone-binding protein RBBP4
rbbp8						0.45							K20773	DNA endonuclease RBBP8
rbfa	0.70										Loss	Loss	K02834	Ribosome-binding factor A
rbl1	0.49					-0.79			Loss					Retinoblastoma-like protein 1
rbl2	-0.58	-0.32	-0.53										K04681, K16332	Retinoblastoma-like protein 1, retinoblastoma-like protein 2
rbm20						0.71								RNA-binding protein 20
rbm26				-0.38		-0.53								RNA-binding protein 26
rbm27						-0.62								RNA-binding protein 26, RNA-binding protein 27
rbm34	0.54					0.38								Nucleolar protein 12
rbm42	0.74	0.33	0.64											RNA-binding protein 42
rbm8a	0.33													RNA-binding protein 8A
rbms1						-0.76								RNA-binding motif, single-stranded-interacting protein
rbp2						0.76							K14622	Retinol binding protein 2

rbpj						0.66								Recombining binding protein suppressor of hairless
rbpms			-0.41										K25091	RNA-binding protein with multiple splicing
rbpms2	-0.56		-0.48										K25091	RNA-binding protein with multiple splicing
rc3h1				-0.51		-1.08							K15690	RING finger and CCCH-type zinc finger domain-containing protein
rcbtb1	-0.40												K11494	RCC1 and BTB domain-containing protein
rcc1	0.90	0.41	0.72										K11493	Regulator of chromosome condensation
rcc1l							Loss	Loss	Loss			Gain		RCC1-like G exchanging factor-like protein
rccd1						0.63								
rce1	0.42	0.32											K07052, K08658	CAAX protease family protein, prenyl protein peptidase
rchy1								Gain						RING finger and CHY zinc finger domain-containing protein 1
rcn2						0.94							K23899	Reticulocalbin-2
rcor2	0.57												K11829	REST corepressor 1
rcor3							Gain						K11829	REST corepressor 1
rd3							Loss				Loss	Gain + Loss		Protein RD3
rdh10	-0.48		-0.45										K11151	Retinol dehydrogenase 10
rdh13												Gain	K11161	Retinol dehydrogenase 13
recql4	0.85	0.70	1.04											ATP-dependent DNA helicase Q4
reep3	-0.30											Loss	K17338	Receptor expression-enhancing protein 1/2/3/4
relch						-0.36							K26154	RAB11-binding protein RELCH
relt						0.48							K05156	Tumor necrosis factor receptor superfamily member 19-like
renbp	-0.58		-0.52			0.78							K01787	N-acylglucosamine 2-epimerase
reps1	0.44		0.45										K20068	Ralbp1-associated Eps domain-containing protein
rer1	0.30							Gain	Gain		Gain + Loss	Gain		
rerg	-0.39													Ras-related and estrogen-regulated growth inhibitor
rest	-0.74	-0.30	-0.59			-0.53								RE1-silencing transcription factor
retreg1	-2.23	-1.51	-2.83											Reticulophagy regulator
retreg2	-0.84	-0.33	-0.96											Reticulophagy regulator
rexo2	0.77													Oligoribonuclease
rexo4	0.52		0.41											RNA exonuclease 4
rfc1	0.62		0.71											Replication factor C subunit 1
rfc2			0.43											Replication factor C subunit 2/4

rfc3	0.52		0.54										K10756	Replication factor C subunit 3/5
rfc4	0.83	0.52	0.67			-0.42							K10755	Replication factor C subunit 2/4
rffl				0.44									K20804	E3 ubiquitin-protein ligase RNF34
rflnb						-0.86								
rft1						-0.47							K06316	Oligosaccharide translocation protein RFT1
rftn1			-0.38			0.44								
rfx1			-0.39			-0.90							K09173	Regulatory factor X 1/2/3
rfx2						-0.99							K09173	Regulatory factor X 1/2/3
rfx5						0.58							K08061	Regulatory factor X 5
rfx7						-0.76								Regulatory factor X 5
rfxank		-0.41	-0.34										K08062	Regulatory factor X-associated ankyrin-containing protein
rgcc		-0.51	-0.45											Regulator of cell cycle RGCC
rgl1	-0.33		-0.40										K17635	Ral guanine nucleotide dissociation stimulator-like 1
rgma						-0.80							K23096	Repulsive guidance molecule A
rgp1	-0.35													RAB6A-GEF complex partner protein 2
rhbdd3						-0.66								
rhbdf1				0.43		0.32	Gain							
rhbdf2						-0.56								
rhbdl3				-0.40		-0.82							K02857	Rhomboid-related protein 1/2/3
rhebl1						0.55							K07208	Ras homolog enriched in brain
rhof			-0.42											Ras homolog gene family, member F, Rho family, other
rhoh								Loss			Loss		K07873	Ras homolog gene family, member H
rhoq	-1.20	-0.63	-1.19											Ras homolog gene family, member Q
rhot2						0.56								Mitochondrial Rho gtpase 1 , mitochondrial Rho gtpase 2
rhpn1			-0.44			-0.53								Rhophilin-1
ric8b						-0.29								
rif1	0.38												K11138	Telomere-associated protein RIF1
rilpl1												Loss		RILP-like protein 1
rin2	-0.36					-0.48								Ras and Rab interactor 2/3
riox1	0.41					0.43								Protein-L-histidine (3S)-3-hydroxylase /
ripor1						0.42							K24818	Rho family-interacting cell polarization regulator
ripor2						-0.42						Gain	K24818	Rho family-interacting cell polarization regulator
rlbp1						0.60							K19625	Retinaldehyde-binding protein 1

rmc1	-0.35												K24763	Regulator of MON1-CCZ1 complex
rmi1	0.38		0.37										K10990	Recq-mediated genome instability protein 1
rmi2	0.45													Recq-mediated genome instability protein 2
rmnd1	0.68													Required for meiotic nuclear division protein 1
rmnd5b						0.69								E3 ubiquitin-protein transferase RMND5
rnaset2	-0.39												K01166	Ribonuclease T2
rnf10						-0.39								
rnf103	-0.71	-0.81	-0.82										K15695	E3 ubiquitin-protein ligase RNF103
rnf11						0.55								E3 ubiquitin-protein ligase RNF11
rnf111	-0.43	-0.45	-0.57											E3 ubiquitin-protein ligase Arkadia
rnf114	-0.43		-0.43			0.48								E3 ubiquitin-protein ligase RNF114
rnf115						0.40								E3 ubiquitin-protein ligase RNF115/126
rnf122			-0.58										K15699	RING finger protein 122
rnf13	-0.31		-0.39											E3 ubiquitin-protein ligase RNF13
rnf14				-0.33		-0.50							K11971	E3 ubiquitin-protein ligase RNF14
rnf141			0.49											
rnf144a	-0.75		-0.41										K11975	E3 ubiquitin-protein ligase RNF144
rnf145				-0.68		-1.05								E3 ubiquitin-protein ligase RNF145
rnf146	-0.87	-0.50	-0.71			-0.52								E3 ubiquitin-protein ligase RNF146
rnf151	-0.51													RING finger protein 151
rnf185						0.84								E3 ubiquitin-protein ligase RNF5
rnf2	-0.85		-0.51										K10695	E3 ubiquitin-protein ligase RNF1/2
rnf20			0.36										K10696	E3 ubiquitin-protein ligase BRE1
rnf216				0.49		0.56								E3 ubiquitin-protein ligase RNF216
rnf217	-0.40		-0.47											E3 ubiquitin-protein ligase RNF217
rnf24	-1.81	-0.89	-1.82										K15699	RING finger protein 122
rnf25	-0.86		-0.45			0.47							K10640	E3 ubiquitin-protein ligase RNF25
rnf31	-0.51												K11974	E3 ubiquitin-protein ligase RNF31
rnf41	0.48													E3 ubiquitin-protein ligase NRDP1
rnf5						0.31							K10666	E3 ubiquitin-protein ligase RNF5
rnf6						-0.44								E3 ubiquitin-protein ligase RLIM , E3 ubiquitin-protein ligase RNF6
rnf7				-0.36										RING-box protein 2
rnf8	0.31						Loss	Loss	Loss		Loss			E3 ubiquitin-protein ligase RNF8

rnft1						0.73								E3 ubiquitin-protein ligase RNFT1
rnps1			0.42					Loss			Gain			Pre-mrna-splicing regulator WTAP, RNA-binding protein with serine-rich domain 1
robo4				-0.41		-0.80							K06784	Roundabout, axon guidance receptor 4
ror2						-0.83								Receptor tyrosine kinase-like orphan receptor 2
rp1											Gain		K19538	Retinitis pigmentosa 1
rpa1			0.44						Gain		Gain	Gain		Replication factor A1
rpa2	0.57	0.44	0.76										K10739	Replication factor A2
rpa3	0.50												K10740	Replication factor A3
rpf1	0.45					0.40								Ribosome production factor 1
rpgrip1l				-0.61		-0.84					Loss	Loss	K16550	Protein fantom
rpia	0.51												K01807	Ribose 5-phosphate isomerase A
rpl12	-0.49												K02870	Large subunit ribosomal protein l12e
rpl35a				-0.41									K02917	Large subunit ribosomal protein L35Ae
rpl7a	-0.38												K02936	Large subunit ribosomal protein L7Ae
rpl8	-0.41					0.41								Large subunit ribosomal protein l8e
rpn1	0.45		0.46										K12666	Oligosaccharyltransferase complex subunit alpha (ribophorin I)
rpn2	0.46		0.45											Oligosaccharyltransferase complex subunit delta (ribophorin II)
rpp30		-0.32	-0.36										K03539	Ribonuclease P/MRP protein subunit RPP1
rpp38			-0.41										K14523	Ribonucleases P/MRP protein subunit RPP38
rprd1b	0.37			-0.35									K15559	Regulator of Ty1 transposition protein 103
rprm		0.56	0.55										K10128	Reprimo, TP53-dependent G2 arrest mediator candidate
rps10	-0.38													Small subunit ribosomal protein s10e
rps2						0.69								Small subunit ribosomal protein s2e
rps23				-0.40										Small subunit ribosomal protein s23e
rps3a	-0.40													Small subunit ribosomal protein S3Ae
rps6kc1	0.53													Ribosomal protein S6 kinase-like
rps6kl1												Loss		Ribosomal protein S6 kinase-like
rps8	-0.39													Small subunit ribosomal protein s8e
rptor						0.71								Regulatory associated protein of mtor
rpusd2	0.56		0.48	-0.47										Trna pseudouridine32 synthase
rrad						0.74							K07845	Ras-related associated with diabetes
rrn3			-0.34			0.36								RNA polymerase I-specific transcription initiation factor RRN3

rrp12	0.55												K14794	Ribosomal RNA-processing protein 12
rrp15	0.66			-0.36										
rrp1b	0.71	0.48	0.49			0.41							K14849	Ribosomal RNA-processing protein 1
rrp36	0.36					0.40							K14795	Ribosomal RNA-processing protein 36
rrp7a	0.58			-0.37										Ribosomal RNA-processing protein 7
rrp8	0.36			-0.42										Ribosomal RNA-processing protein 8
rrp9	0.53		0.38											Ribosomal RNA-processing protein 9
rrs1						0.48								Regulator of ribosome biosynthesis
rskr	-0.47	-0.58	-0.76											Uncharacterized serine/threonine-protein kinase sgk494
rsl1d1	0.50		0.42										K14775	Ribosome biogenesis protein UTP30
rsl24d1			-0.43										K02896	Large subunit ribosomal protein l24e
rsrc1	0.55												K24594	Arginine/serine-rich coiled-coil protein 1
rsrc2				0.26										Arginine/serine-rich coiled-coil protein 2
rsu1				-0.47		-0.37								
rtca			0.36											RNA 3'-terminal phosphate cyclase (ATP)
rtel1	0.52		0.35										K11136	Regulator of telomere elongation helicase 1
rtf2	-0.58													Replication termination factor 2
rtkn			-0.34			0.42							K24024	Rhotekin
rtn4ip1	0.72					0.56							K23164	Reticulon-4-interacting protein 1, mitochondrial
rttn											Gain + Loss	Gain + Loss		Rotatin
rufy2	-0.40		-0.44										K12482	RUN and FYVE domain-containing protein 1/2
rundc3a		-0.54	-0.45			-0.69								RUN domain-containing protein 3
runx1t1						0.58							K10053	CBFA2/RNUX1 translocation partner 1
runx2						0.56							K09278	Runt-related transcription factor 2
ruvbl1	0.48		0.48											Ruvb-like protein 1
ruvbl2	1.05	0.43	0.83										K11338	Ruvb-like protein 2
rwdd2b	0.39			-0.45										
rwdd3												Gain		
rwdd4						0.46								
rxfp2						0.59							K04307	Relaxin family peptide receptor 2
rxra						0.58								Retinoid X receptor alpha
rxylt1	-0.44		-0.60											Alpha-dystroglycan beta1,4-xylosyltransferase

ryk			0.35											RYK receptor-like tyrosine kinase
ryr3	-0.53													Ryanodine receptor 3
sacs			-0.50											Sacsin
sae1	0.45		0.58											Ubiquitin-like 1-activating enzyme E1 A
sall1						-0.49							K19871	Sal-like protein
sall3						-0.46							K19871	Sal-like protein
samd15											Loss	Loss		
samd7								Gain			Gain			
samd8						-0.71							K22697	Sphingomyelin synthase-related protein 1
samhd1			0.47											Deoxynucleoside triphosphate triphosphohydrolase SAMHD1
sap130							Gain	Gain					K19192	Histone deacetylase complex subunit SAP130
sap18			0.32											Histone deacetylase complex subunit SAP18
sar1b	0.42	0.37	0.45										K07953	GTP-binding protein SAR1
saraf	-0.41													
sardh				-0.52		-0.55								Sarcosine dehydrogenase
sarm1						-0.48								
sars2	0.50		0.48											Seryl-trna synthetase
sart3	0.45		0.37	-0.39									K22611	Squamous cell carcinoma antigen recognized by T-cells 3
sash1				-0.38		-0.87							K23705	SAM and SH3 domain-containing protein 1
sass6	0.57												K16487	Spindle assembly abnormal protein 6
sat2	0.43		0.46											Diamine N-acetyltransferase
satb1		-0.44				-0.70							K23225	DNA-binding protein SATB1
sav1						-0.28							K16686	Scaffold protein salvador
saysd1	-0.47	-0.34	-0.55											
sbno1						-0.29								
scaf11	0.42	0.36	0.39											
scamp1						-0.27							K19995	Secretory carrier-associated membrane protein
scamp3				-0.48		-0.98							K19995	Secretory carrier-associated membrane protein
scaper						0.36							K24869	S phase cyclin A-associated protein in the endoplasmic reticulum
scara3						-0.51								
scara5				-0.47		-0.39								
scarb2	-1.04	-0.73	-1.18										K12384	Lysosome membrane protein 2
sccpdh						0.48								

scfd1			0.24										K19998	Sec1 family domain-containing protein 1
schip1				-0.49		-0.66							K24832, K24833	IQ domain-containing protein J, schwannomin-interacting protein 1
scn3b						0.75								Voltage-gated sodium channel type III beta
scn8a											Gain		K04840	Voltage-gated sodium channel type VIII alpha
scnm1	0.46												K24827	Sodium channel modifier 1
scrn3						0.35								Secernin
sctr							Gain + Loss	Gain + Loss	Gain + Loss			Loss	K04588	Secretin receptor
scyl1						-0.60							K08876	SCY1-like protein 1
scyl3	-0.29													SCY1-like protein 3
sdccag8		-0.53												Serologically defined colon cancer antigen 8
sdf2l1	0.42													
sdha						-0.72								Succinate dehydrogenase (ubiquinone) flavoprotein subunit , succinate dehydrogenase flavoprotein subunit
sdhb				-0.32		-0.42							K00235, K00240	Succinate dehydrogenase (ubiquinone) iron-sulfur subunit , succinate dehydrogenase iron-sulfur subunit
sdk1						0.52								Protein sidekick
sdk2											Gain		K16353	Protein sidekick
sdr16c5												Loss	K15734	All-trans-retinol dehydrogenase (NAD+)
sec11a						0.43							K13280	Signal peptidase I
sec16a	0.22	0.23											K20353	COPII coat assembly protein SEC16
sec23ip	0.42	0.31	0.48											Phospholipase DDHD2
sec24a	0.31		0.31			-0.35							K14007	Protein transport protein SEC24
sec31a						-0.68								Protein transport protein SEC31
sec61a1	0.61	0.46	0.62											Protein transport protein SEC61 subunit alpha
sec61b	0.60		0.60										K09481	Protein transport protein SEC61 subunit beta
sec63		0.48											K09540	Translocation protein SEC63
secisbp2l												Loss		Selenocysteine insertion sequence-binding protein 2
sel1l	0.35	0.30	0.33											SEL1 protein, uncharacterized protein
selenoi				-0.35										Ethanolaminephosphotransferase
selenok						0.52								
selenoo	0.60													Protein adenylyltransferase
selenop	-1.88		-1.59										K25753	Selenoprotein P
selenos						0.40								Selenoprotein S

selp	-0.59		-0.89											Selectin, platelet
sema3d				-0.53		-0.89								Semaphorin 3
sema3g						0.71							K06840	Semaphorin 3
sema4c				-0.58		-0.85							K06521	Semaphorin 4
sema4d						0.67							K06521	Semaphorin 4
sema4g											Gain	Gain		Semaphorin 4
senp7			-0.48											
senp8						0.45								Sentrin-specific protease 8
sephs1			0.38										K01008	Selenide, water dikinase
septin10		0.31	0.35										K16940	Septin 10
serac1											Loss	Loss	K23504	Protein SERAC1
sergef	0.46													Secretion-regulating guanine nucleotide exchange factor
serinc1	-1.50		-1.32											Serine incorporator 1
serinc4						-1.26							K24177	Serine incorporator 4
sertad3												Loss		
sesn1	-1.70	-1.17	-2.06										K10141	Sestrin 1/3
sestd1						0.26								
setd1a						-0.49								
setd3						0.60								
setd4						-0.74								
setd6	0.75		0.44											N-lysine methyltransferase SETD6
sf1			0.28				Gain						K13095	Splicing factor 1
sf3a1	0.30	0.34	0.43										K12825	Splicing factor 3A subunit 1
sf3a2		0.39	0.55			0.38								Splicing factor 3A subunit 2
sf3a3	0.67	0.30	0.50											Splicing factor 3A subunit 3
sf3b2	0.54		0.52											Splicing factor 3B subunit 2
sf3b3	0.63	0.37	0.51											Splicing factor 3B subunit 3
sf3b4	0.87	0.61	0.94										K12831	Splicing factor 3B subunit 4
sfmbt1			-0.36			-0.42								
sfmbt2				0.45										
sfswap				0.54		0.52								
sft2d3				0.46		0.52								
sfxn1	0.40													Sideroflexin-1/3

sfxn2	0.45												K23501	Sideroflexin-2
sfxn4	0.44													Sideroflexin-4
sgcb						-0.68							K12566	Beta-sarcoglycan
sgcd						0.64					Loss	Loss	K12563	Delta-sarcoglycan
sgcg				-0.40		-0.42							K12564	Gamma-sarcoglycan
sgip1				0.53									K20065	SH3-containing GRB2-like protein 3-interacting protein 1
sgk1	-0.41		-0.44										K13302	Serum/glucocorticoid-regulated kinase 1
sgk2												Gain		
sgms1	-0.58													Shingomyelin synthase
sgtb						-0.47								Small glutamine-rich tetratricopeptide repeat-containing protein alpha, small glutamine-rich tetratricopeptide repeat-containing protein beta
sh2b1						0.63							K12459	SH2B adaptor protein 1/3
sh2b2											Loss	Loss	K07193	SH2B adapter protein 2
sh3bp2						0.69							K07984	SH3-domain binding protein 2
sh3bp4						-0.51							K20066	SH3 domain-binding protein 4
sh3gl1	0.34	0.26	0.33										K11247	Endophilin-A
sh3kbp1				0.40		0.92							K12470	SH3 domain-containing kinase-binding protein 1
sh3pxd2a						-0.61								SH3 and PX domain-containing protein 2
sh3rf1						-0.54								E3 ubiquitin-protein ligase SH3RF
sh3tc1			-0.47								Gain		K24313	SH3 domain and tetratricopeptide repeat-containing protein
sh3tc2								Loss	Loss	Gain	Loss	Gain + Loss	K24313	SH3 domain and tetratricopeptide repeat-containing protein
shank2											Gain			SH3 and multiple ankyrin repeat domains protein
sharpin			-0.43											Shank-associated RH domain-interacting protein
she						0.45								SH2 domain-containing adapter protein B/D/E/F
shf											Gain	Gain		SH2 domain-containing adapter protein B/D/E/F
shkbp1		0.26	0.33											SH3KBP1-binding protein 1
shmt2						0.47							K00600	Glycine hydroxymethyltransferase
shpk			-0.55											Sedoheptulokinase
shprh				0.51		0.86							K15710	E3 ubiquitin-protein ligase SHPRH
shroom4						-0.67							K18625	Protein Shroom
siah1						-1.07								E3 ubiquitin-protein ligase SIAH1
sidt2	-1.56	-1.17	-1.47										K26112	SID1 transmembrane family member 2

sike1	0.39													Suppressor of IKK-epsilon
sil1						0.41							K14001	Nucleotide exchange factor SIL1
sin3a	-0.82	-0.41	-0.74										K11644	Paired amphipathic helix protein Sin3a
sin3b				-0.36		-0.94					Gain			Paired amphipathic helix protein Sin3a, paired amphipathic helix protein Sin3b
sirt1						-0.68								NAD+-dependent protein deacetylase SIR2 , NAD+-dependent protein deacetylase sirtuin 1
sirt2	-0.50		-0.42										K11412	NAD+-dependent protein deacetylase sirtuin 2
sirt4			-0.40										K11414	NAD+-dependent protein deacetylase sirtuin 4
sirt5			-0.40										K11415	NAD+-dependent protein deacetylase sirtuin 5 , NAD-dependent deacetylase
sirt6						-0.48							K11416	NAD+-dependent protein deacetylase sirtuin 6
six1	-0.39												K15614	Homeobox protein SIX1
six2						-0.40							K15614, K19472	Homeobox protein SIX1, homeobox protein SIX2
six4			-0.39			-0.62							K15615	Homeobox protein SIX4
ska1	0.50		0.55										K26094	Spindle and kinetochore-associated protein 1
ska2						0.52								Spindle and kinetochore-associated protein 2
ska3			0.40											Spindle and kinetochore-associated protein 3
skap2				0.39		0.57							K23471	Src kinase-associated phosphoprotein 2
skil						0.60								Ski-like protein
skor1				-0.42		-0.92								
skp2	0.45		0.51										K03875	F-box and leucine-rich repeat protein 1 (S-phase kinase-associated protein 2)
sla						0.52								Src-like-adapter
slain1						-0.77								SLAIN motif-containing protein 1/2
slain2			-0.48			-0.46								Anaphase-promoting complex subunit 4, SLAIN motif-containing protein 1/2
slbp	0.52	0.36	0.48			-0.71							K18710	Histone RNA hairpin-binding protein
slc12a3	-1.51		-1.28	0.25										Solute carrier family 12 (sodium/chloride transporter), member 3
slc12a4						0.46					Gain	Gain	K14427	Solute carrier family 12 (potassium/chloride transporter), member 4/6
slc12a5							Gain	Gain					K23967	Solute carrier family 12 (potassium/chloride transporter), member 5
slc12a9	-0.42												K14429	Solute carrier family 12 (potassium/chloride transporters), member 9
slc13a3											Loss	Loss		Solute carrier family 13 (sodium-dependent dicarboxylate transporter), member 2/3/5

slc15a4	-0.45		-0.52			-0.46								Solute carrier family 15 (peptide/histidine transporter), member 3/4
slc15a5							Gain	Gain					K14639	Solute carrier family 15, member 5
slc16a1	-1.14	-0.42	-2.05											MFS transporter, MCT family, solute carrier family 16 (monocarboxylic acid transporters), member 1
slc16a12	-0.69					-0.53							K11502, K11810	Centromere protein J, MFS transporter, MCT family, solute carrier family 16 (monocarboxylic acid transporters), member 12
slc16a13			-0.54											MFS transporter, MCT family, solute carrier family 16 (monocarboxylic acid transporters), member 13
slc16a5	-0.49					-0.57							K08182	MFS transporter, MCT family, solute carrier family 16 (monocarboxylic acid transporters), member 5
slc16a9		-0.48											K08186	MFS transporter, MCT family, solute carrier family 16 (monocarboxylic acid transporters), member 9
slc17a5						-0.44							K08193, K12301	MFS transporter, ACS family, solute carrier family 17 (sodium-dependent inorganic phosphate cotransporter), member 5, MFS transporter, ACS family, solute carrier family 17 (sodium-dependent inorganic phosphate cotransporter), other
slc19a1				-0.39		-0.49								Solute carrier family 19 (folate transporter), member 1
slc19a2												Loss	K14610	Solute carrier family 19 (thiamine transporter), member 2/3
slc1a1			-0.51											Solute carrier family 1 (neuronal/epithelial high affinity glutamate transporter), member 1
slc1a4						1.16								Solute carrier family 1 (neutral amino acid transporter), member 4, solute carrier family 1 (neutral amino acid transporter), member 5
slc1a7						-0.82								Solute carrier family 1 (glutamate transporter), member 7
slc22a15	0.83	0.36	0.75											MFS transporter, OCT family, solute carrier family 22 (organic cation transporter), member 15
slc22a16						-0.75							K08212	MFS transporter, OCT family, solute carrier family 22 (organic cation transporter), member 16
slc22a18												Gain		MFS transporter, OCT family, solute carrier family 22 (organic cation transporter), member 18
slc22a2							Loss						K08199, K08200, K08202	MFS transporter, OCT family, solute carrier family 22 (organic cation transporter), member 2, MFS transporter, OCT family, solute carrier family 22 (organic cation transporter), member 3, MFS transporter, OCT family, solute carrier family 22 (organic cation transporter), member 4/5
slc23a2				0.56										Solute carrier family 23 (nucleobase transporter), member 1, solute carrier family 23 (nucleobase transporter), member 2
slc24a1									Loss				K13749	Solute carrier family 24 (sodium/potassium/calcium exchanger), member 1
slc25a14	0.61													Solute carrier family 25 (mitochondrial carrier), member 14/30
slc25a15	0.77			-0.44									K15101	Solute carrier family 25 (mitochondrial ornithine transporter) member 2/15

slc25a16											Gain			Solute carrier family 25 (mitochondrial carrier protein), member 16
slc25a19	1.18	0.50	0.87										K15108	Solute carrier family 25 (mitochondrial thiamine pyrophosphate transporter), member 19
slc25a20	0.49					-0.43								Solute carrier family 25 (mitochondrial carnitine/acylcarnitine transporter), member 20/29
slc25a26	0.49													Solute carrier family 25 (mitochondrial S-adenosylmethionine transporter), member 26
slc25a28						0.41							K15113	Solute carrier family 25 (mitochondrial iron transporter), member 28/37
slc25a29				-0.56			Gain		Gain			Gain + Loss		Solute carrier family 25 (mitochondrial carnitine/acylcarnitine transporter), member 20/29
slc25a33			-0.74	-0.37		-0.52	Gain							Solute carrier family 25, member 33/36
slc25a34	-0.66		-0.76			-0.66							K15117	Solute carrier family 25, member 34/35
slc25a37						-1.05								Solute carrier family 25 (mitochondrial iron transporter), member 28/37
slc25a42	-1.30	-0.85	-1.55											Solute carrier family 25, member 42
slc25a43	-1.12	-0.68	-1.47											Solute carrier family 25, member 43
slc25a45										Loss	Loss		K15123	Solute carrier family 25, member 45/47
slc25a48	-2.17		-0.76			-0.60							K15124	Solute carrier family 25, member 48
slc26a11						-0.52								Solute carrier family 26 (sodium-independent sulfate anion transporter), member 11
slc26a2	-0.66		-0.42										K14701	Solute carrier family 26 (sulfate anion transporter), member 2
slc26a4							Gain	Gain	Gain					Solute carrier family 26 (sodium-independent chloride/iodide transporter), member 4
slc26a5				-0.44										Solute carrier family 26, member 5
slc27a4	0.63		0.62										K08745	Solute carrier family 27 (fatty acid transporter), member 1/4
slc27a6						0.56							K08749	Solute carrier family 27 (fatty acid transporter), member 6
slc28a3	-0.60													Pyrimidine nucleoside transport protein
slc2a10						-0.71								MFS transporter, SP family, solute carrier family 2 (facilitated glucose transporter), member 10
slc2a8	-0.36		-0.41			-0.52								Facilitated trehalose transporter, MFS transporter, SP family, solute carrier family 2 (facilitated glucose transporter), member 8
slc2a9	-0.54		-0.78			0.57		Gain					K08146	MFS transporter, SP family, solute carrier family 2 (facilitated glucose transporter), member 9
slc30a1						-0.56								Solute carrier family 30 (zinc transporter), member 1
slc30a6						0.54							K14693	Solute carrier family 30 (zinc transporter), member 6
slc30a7			0.62											Solute carrier family 30 (zinc transporter), member 5/7
slc30a9						0.42								Solute carrier family 30 (zinc transporter), member 9

slc31a1	0.40	0.33												Solute carrier family 31 (copper transporter), member 1
slc33a1	0.63	0.52	0.53				Loss							MFS transporter, PAT family, solute carrier family 33 (acetyl-coa transportor), member 1
slc34a2								Loss	Loss					Solute carrier family 34 (sodium-dependent phosphate cotransporter)
slc35a4	0.62					-0.55								Solute carrier family 35 (probable UDP-sugar transporter), member A4
slc35b2	0.35		0.36											Solute carrier family 35 (adenosine 3'-phospho 5'-phosphosulfate transporter), member B2
slc35c1											Gain		K15279	Solute carrier family 35 (GDP-fucose transporter), member C1
slc35d1						0.77								Solute carrier family 35, member D1/2/3
slc35e4			-0.56			-0.46								Solute carrier family 35, member E4
slc35f5	-0.95	-0.60	-0.69					Gain				Gain	K15289	Solute carrier family 35, member F5
slc35f6	-0.73	-0.51	-0.67	0.41										
slc35g1						-0.84								
slc37a1						-0.38								MFS transporter, OPA family, solute carrier family 37 (glycerol-3-phosphate transporter), member 1/2
slc38a10						-0.41							K14996	Solute carrier family 38 (sodium-coupled neutral amino acid transporter), member 10
slc38a11							Gain							Solute carrier family 38 (sodium-coupled neutral amino acid transporter), member 11
slc38a9	-0.52		-0.52											Solute carrier family 38 (sodium-coupled neutral amino acid transporter), member 9
slc39a10				0.39		0.56							K14716	Solute carrier family 39 (zinc transporter), member 10
slc39a14	0.67		0.60										K14720	Solute carrier family 39 (zinc transporter), member 14
slc39a7	0.50		0.41											Solute carrier family 39 (zinc transporter), member 7
slc39a9			0.43											Solute carrier family 39 (zinc transporter), member 9
slc3a2			-0.62										K06519	Solute carrier family 3, member 2
slc40a1	-0.51		-0.64			-0.39								Solute carrier family 40 (iron-regulated transporter), member 1
slc41a1						0.67							K15122	Solute carrier family 41
slc44a5	-0.95	-0.48	-0.82			-0.44	Gain + Loss	Gain			Loss			Solute carrier family 44 (choline transporter-like protein), member 2/4/5
slc45a3						-0.44							K15379	Solute carrier family 45, member 3
slc49a3				0.46										MFS transporter, FLVCR family, MFS-domain-containing protein 7
slc49a4	-1.78	-1.10	-1.50											MFS transporter, FLVCR family, disrupted in renal carcinoma protein 2
slc4a10							Gain	Gain				Gain	K13861	Solute carrier family 4 (sodium bicarbonate transporter), member 10
slc4a1ap			0.31											

slc50a1	-1.89	-0.43	-1.12	-0.80		-0.82								Solute carrier family 50 (sugar transporter)
slc66a1	-1.95	-1.18	-2.55										K23678	Solute carrier family 66 (lysosomal lysine-arginine transporter), member 1
slc66a3	0.50	0.60	0.63											Solute carrier family 66, member 3
slc6a14	-2.11		-2.25											Solute carrier family 6 (neurotransmitter transporter) member 14
slc6a9						-0.52							K05038	Solute carrier family 6 (neurotransmitter transporter, glycine) member 5/9
slc7a4						-0.71								Solute carrier family 7 (cationic amino acid transporter), member 4
slc7a5				-0.32		-0.54							K13780	Solute carrier family 7 (L-type amino acid transporter), member 5
slc7a6							Gain				Loss	Gain		Solute carrier family 7 (L-type amino acid transporter), member 6, solute carrier family 7 (L-type amino acid transporter), member 7
slc7a9	-0.65		-0.53										K13868	Solute carrier family 7 (L-type amino acid transporter), member 9/15
slc8a3							Gain							Solute carrier family 8 (sodium/calcium exchanger)
slc8b1	-0.78	-0.60	-0.59											Solute carrier family 24 (sodium/potassium/calcium exchanger), member 6
slc9a6				0.41										Solute carrier family 9 (sodium/hydrogen exchanger), member 6/7
slco2a1	-1.59	-0.90	-0.89											Solute carrier organic anion transporter family, member 2A
slfnl1											Loss			
slit3				-0.64		-1.14								Slit 3
slitrk2						-0.61					Gain			SLIT and NTRK-like protein 2
slitrk4						-0.81								SLIT and NTRK-like protein 4
slitrk5											Loss			SLIT and NTRK-like protein 5
slk						-1.05							K08836	STE20-like kinase
sltm						-0.59								SAFB-like transcription modulator
slu7			0.39										K12819	Pre-mrna-processing factor SLU7
slx4				0.41								Loss		Structure-specific endonuclease subunit SLX4 (BTB/POZ domain-containing protein 12)
smad1			-0.35			-0.65								
smad2	-0.19												K04500	Mothers against decapentaplegic homolog 2
smarca5	0.66	0.59	0.69										K11654	SWI/SNF-related matrix-associated actin-dependent regulator of chromatin subfamily A member 5
smarcad1	0.49		0.35										K14439	SWI/SNF-related matrix-associated actin-dependent regulator of chromatin subfamily A containing DEAD/H box 1
smarcb1			0.39											SWI/SNF-related matrix-associated actin-dependent regulator of chromatin subfamily B member 1
smarcc1	0.36		0.36											SWI/SNF-related matrix-associated actin-dependent regulator of chromatin subfamily C

smarce1						0.58							K11651	SWI/SNF-related matrix-associated actin-dependent regulator of chromatin subfamily E member 1
smc2	0.51		0.61											Structural maintenance of chromosome 2
smc4			0.43										K06675	Structural maintenance of chromosome 4
smc5	0.48		0.65			0.41								Structural maintenance of chromosomes protein 5
smg1	0.41												K08873	Serine/threonine-protein kinase SMG1
smg6	-0.58	-0.32	-0.51											Protein SMG6
smim12	0.70		0.43											
smim13	-0.54	-0.46	-0.69											
smim14	-0.41													
smim15			0.33											
smim22	-0.54													
smim30	0.47													
smim8				-0.38										
smndc1						0.34							K12839	Survival of motor neuron-related-splicing factor 30
smo				-0.51		-1.22							K06226	Smoothened
smox						-0.90							K12259	Spermine oxidase
smpd3						0.71							K12352	Sphingomyelin phosphodiesterase 3
smpdl3a	-0.53	-0.53												Sphingomyelin phosphodiesterase acid-like 3
smpx						0.61								Small muscular protein
sms	0.39													Spermine synthase
smug1	-1.07	-1.32	-1.33										K10800	Single-strand selective monofunctional uracil DNA glycosylase
smurf2	0.35		0.43										K04678	E3 ubiquitin ligase SMURF1/2
smyd5	0.48					0.45							K17770, K24635	Mitochondrial import receptor subunit TOM20, SET and MYND domain-containing protein 5
snap47	-1.57	-1.23	-1.77										K18212	Synaptosomal-associated protein 47
snapc2							Loss	Loss						Snrna-activating protein complex subunit 2
snapc3						0.50								Snrna-activating protein complex subunit 3
snapc4	0.46		0.56			0.58							K09453	Snrna-activating protein complex subunit 4
snapc5			-0.40											Snrna-activating protein complex subunit 5
snip1			0.44											Smad nuclear-interacting protein 1
snrnp200	0.51	0.45	0.58											Pre-mrna-splicing helicase BRR2
snrnp25	0.42												K13153	U11/U12 small nuclear ribonucleoprotein 25 kda protein
snrnp40	0.75		0.39											Prp8 binding protein

snrnp48	0.33												K13156	U11/U12 small nuclear ribonucleoprotein 48 kda protein
snrpa	0.48		0.55										K11091	U1 small nuclear ribonucleoprotein A
snrpa1	0.64	0.38	0.69										K11092	U2 small nuclear ribonucleoprotein A'
snrpb	0.36		0.55			0.34							K11086	Small nuclear ribonucleoprotein B and B'
snrpb2	0.62		0.57											U2 small nuclear ribonucleoprotein B''
snrpc						-0.54							K11095	U1 small nuclear ribonucleoprotein C
snrpd1	0.56													Small nuclear ribonucleoprotein D1
snrpe	0.47													Small nuclear ribonucleoprotein E
snrpf	0.47												K11098	Small nuclear ribonucleoprotein F
snta1	0.41												K24063	Alpha-syntrophin
sntb2						-0.64								Beta-syntrophin
snu13	0.63												K12845	U4/U6 small nuclear ribonucleoprotein SNU13
snw1				-0.37		-0.69							K06063	SNW domain-containing protein 1
snx1						-0.76							K17917	Sorting nexin-1/2
snx11						0.49								Sorting nexin-10/11
snx13				0.34									K17925	Sorting nexin-13
snx15						0.59			Loss				K17927	Sorting nexin-15
snx16				-0.42		-0.65					Gain	Gain		Sorting nexin-16
snx17						0.71								Sorting nexin-17
snx2						0.59								Sorting nexin-1/2
snx30						0.56								Sorting nexin-7/30
snx9				0.34		0.30								Sorting nexin-9/18/33
sobp		0.70	0.47											
socs7	0.50	0.68	0.72										K04699	Suppressor of cytokine signaling 6/7
sord	-0.77												K00008	L-iditol 2-dehydrogenase
sorl1				0.40		0.43							K24498	Sortilin-related receptor
sort1												Gain		Sortilin
sox17		-0.42											K04495	Transcription factor SOX17 (SOX group F)
sox4						-0.37							K23581	Transcription factor SOX4 (SOX group C)
sox7				0.50									K09270	Transcription factor SOX7/8/10/18 (SOX group E/F)
sox9				-0.44		-1.26							K18435	Transcription factor SOX9 (SOX group E)
sp1		-0.32				-0.30							K04684	Transcription factor Sp1
sp3						0.58							K09193	Transcription factor Sp3

sp4						-0.63							K09194	Transcription factor Sp4
sp7						-0.56							K09197	Transcription factor Sp7
spag1						-0.41							K19870	Sperm-associated antigen 1
spart								Gain	Gain		Loss			Spartin
spata1		-0.53	-0.99											
spata2l	-0.54					-0.39		Gain			Gain	Gain	K17595	Spermatogenesis-associated protein 2
spata6				-0.42		-1.49								Spermatogenesis-associated protein 6
spc25	0.53		0.44										K11550	Kinetochore protein Spc25, animal type
spcs2						0.49							K12947	Signal peptidase complex subunit 2
spdl1	0.37		0.39								Gain		K26098	Protein Spindly
specc1						0.57								Cytospin
specc1l						0.74								Cytospin
speg											Gain		K08809	Striated muscle-specific serine/threonine protein kinase
spg11				0.46									K19026	Spatacsin
spg7				-0.44		-1.11							K09552	Spastic paraplegia 7
spice1			0.44											Spindle and centriole-associated protein 1
spns1	-0.50	-0.55	-0.57										K23677	MFS transporter, Spinster family, sphingosine-1-phosphate transporter
spns2											Gain	Gain		MFS transporter, Spinster family, sphingosine-1-phosphate transporter
spock2						0.54								Testican
spon2				-0.43		-0.56								Spondin-2
spop			-0.32			0.37							K10523	Speckle-type POZ protein
spout1	0.73		0.38										K09142	Methyltransferase
sppl2b	-0.33			-0.30		-0.32								Signal peptide peptidase-like 2B
sppl3				-0.34		-0.70								Signal peptide peptidase-like 3
spr				-0.56		-1.24							K00072	Sepiapterin reductase
spry1						-0.65								Protein sprouty homolog 1
spryd4				-0.35										
spryd7	-0.43		-0.49											
spsb4			0.47											SPRY domain-containing SOCS box protein 1/4
sptssa											Gain	Gain		
spty2d1						0.37								Protein SPT2
sqle			0.44										K00511	Squalene monooxygenase

sqstm1	-0.54												K14381	Sequestosome 1
srbd1			0.42										K06959	Protein Tex
srd5a1	-0.40													3-oxo-5-alpha-steroid 4-dehydrogenase 1
srd5a3	-0.57		-0.50											3-oxo-5-alpha-steroid 4-dehydrogenase 3 / polyprenol reductase
srek1				0.40		0.53							K13165	Splicing regulatory glutamine/lysine-rich protein 1
srfbp1						0.38								
srgap2						-0.49							K07526	SLIT-ROBO Rho gtpase activating protein
srgap3						0.85							K07526	SLIT-ROBO Rho gtpase activating protein
srm	0.41													Spermidine synthase
srp68	0.49	0.43	0.36											Signal recognition particle subunit SRP68
srpk1						-0.59								Serine/threonine-protein kinase SRPK1 , serine/threonine-protein kinase SRPK3
srpra	0.27	0.27												Signal recognition particle receptor subunit alpha
srprb				-0.35										Signal recognition particle receptor subunit beta
srrd						0.66								
srrm1						-0.50								Serine/arginine repetitive matrix protein 1
srrt						0.36								
srsf10						-0.31								Serine/arginine-rich splicing factor 10
srsf11			0.30			-0.42							K12899	Serine/arginine-rich splicing factor 11
srsf2	0.48	0.38	0.38			0.61							K12891	Serine/arginine-rich splicing factor 2/8
srsf3						-0.33								Serine/arginine-rich splicing factor 3
srsf4	0.47	0.55	0.53										K12893	Serine/arginine-rich splicing factor 4/5/6
srsf6	0.44			-0.41		-0.70							K12893	Serine/arginine-rich splicing factor 4/5/6
srsf7	0.34												K12896	Serine/arginine-rich splicing factor 7
srxn1	-1.01		-0.89			0.57							K12260	Sulfiredoxin
ss18						-0.50					Loss	Loss	K15623	Protein SSXT
ssb	0.61	0.38	0.34											Lupus La protein
ssbp1	0.67													Single-strand DNA-binding protein
ssbp3						-0.29								
ssna1	0.47		0.50										K16780	Sjoegren syndrome nuclear autoantigen 1
sspn	-0.52													Sarcospan
ssr1						0.49							K13249	Translocon-associated protein subunit alpha
ssr2		0.39				0.63								Translocon-associated protein subunit beta

ssr3						0.40								Translocon-associated protein subunit gamma
ssr4						0.60								Translocon-associated protein subunit delta
ssrp1	0.63	0.39	0.46											FACT complex subunit SSRP1/POB3
ssu72				-0.43		-0.60							K15544	RNA polymerase II subunit A C-terminal domain phosphatase SSU72
st13	0.45	0.34	0.42			0.39								Suppressor of tumorigenicity protein 13
st5				-0.44		-0.50							K20161	DENN domain-containing protein 2
st8sia2	0.92	0.43	0.58										K06612	Alpha-N-acetyl-neuraminate alpha-2,8-sialyltransferase (sialyltransferase 8B)
stab1	-0.56													Stabilin-1
stab2						0.70							K19013	Stabilin-2
stac	-0.47													SH3 and cysteine-rich domain-containing protein
stac2						0.61							K23712	SH3 and cysteine-rich domain-containing protein 2
stard10				0.39									K24142	Star-related lipid transfer protein 10
stard3nl			-0.31											Star-related lipid transfer protein 3
stard9						0.94								Star-related lipid transfer protein 9
stat3	-0.41					-0.45								Signal transducer and activator of transcription 3
stc2	-1.56		-0.91			-0.55								
stil	0.64		0.51										K16724	SCL-interrupting locus protein
stip1	0.59													Stress-induced-phosphoprotein 1
stk11	-0.27		-0.25										K07298	Serine/threonine-protein kinase 11
stk11ip	-0.59		-0.52											
stk16						-0.57								Serine/threonine kinase 16
stk17b						-0.40							K08804	Serine/threonine kinase 17
stk19	-0.41		-0.45										K08880	Serine/threonine kinase 19
stk25						0.41							K08838	Serine/threonine-protein kinase 24/25/MST4
stk38	-0.40													Serine/threonine kinase 38
stom	-0.64		-0.54			-0.59								Erythrocyte band 7 integral membrane protein
stoml1						0.39								
stoml2	0.46													
ston2	-0.88		-0.48			-0.46								Stonin-1/2
stox2	0.38								Loss		Gain	Gain		
strap	0.60		0.34											Serine-threonine kinase receptor-associated protein
strip2				-0.40										

strn3						0.62								Striatin 1/3/4
stt3a	0.51		0.51										K07151	Dolichyl-diphosphooligosaccharide---protein glycosyltransferase
stx10	-0.44					0.41							K23937	Syntaxin 10
stx11											Loss	Loss	K08487	Syntaxin 11
stx17				0.38		0.44								Syntaxin 17
stx18			-0.36											Syntaxin 18
stx19						-0.56							K08487	Syntaxin 11
stx8				0.45		0.60							K08501	Syntaxin 8
stxbp3	-0.26												K15301	Syntaxin-binding protein 3
stxbp5l						-0.66								Syntaxin-binding protein 5
stxbp6	-0.49												K08519	Syntaxin-binding protein 6
sub1	0.40												K25815	Activated RNA polymerase II transcriptional coactivator p15
suds3	0.44		0.32										K19201	Sin3 histone deacetylase corepressor complex component SDS3
sufu			-0.26											Suppressor of fused
sugt1	-0.24	-0.30	-0.27										K12795	Suppressor of G2 allele of SKP1
sulf1						0.58	Gain							Extracellular sulfatase Sulf
sumf1						0.55							K13444	Formylglycine-generating enzyme
sumf2			0.34			0.33								Formylglycine-generating enzyme
sumo2						-0.72								Small ubiquitin-related modifier
suox						-0.56								Sulfite oxidase
supt16h	0.51	0.53	0.54									Loss		FACT complex subunit SPT16
supv3l1	0.55	0.42											K17675	ATP-dependent RNA helicase SUPV3L1/SUV3
surf2	0.42			-0.62										
surf6	0.65					0.67								
susd5						0.96								Sushi domain-containing protein 5
susd6	-0.30													Sushi domain-containing protein 6
suz12	0.47		0.49										K11463	Polycomb protein SUZ12
sv2c							Gain							MFS transporter, VNT family, synaptic vesicle glycoprotein 2
svbp	0.50													Small vasohibin-binding protein
svep1											Loss	Loss	K24469	Sushi, von Willebrand factor type A, EGF and pentraxin domain-containing protein 1
swt1	-0.65				-0.74									
sycp2l									Loss					Synaptonemal complex protein 2

syde2		0.34							Gain + Loss		Gain + Loss	Gain + Loss		Rho gtpase-activating protein SYDE
sympk	0.55		0.55										K06100	Symplekin
syn2						-0.68	Loss				Loss		K19941	Synapsin
sync	-1.29													Syncoilin 1
syncrip	0.75	0.61	0.65											
synj1												Gain		Synaptojanin
synj2bp	0.90												K20057	Synaptojanin-2-binding protein
synpr	-0.53		-0.60											
syt16									Gain			Gain		Synaptotagmin-14/16
syt6						-0.62					Gain	Gain		Synaptotagmin-6
sytl2				0.57		0.53							K17598	Synaptotagmin-like protein
sytl3						-0.61								Synaptotagmin-like protein
saal1						0.44								
tab2						-0.37								TAK1-binding protein 2
tacc1	-0.48		-0.48										K14281	Transforming acidic coiled-coil-containing protein 1
tacc3	0.45		0.75											Transforming acidic coiled-coil-containing protein 3
tacr1							Gain		Gain		Gain			Tachykinin receptor 1, tachykinin receptor 3
tacr2						-1.12								Tachykinin receptor 2
tada1						-0.80							K11317	Transcriptional adapter 1
taf1						0.48							K03125	Transcription initiation factor TFIID subunit 1
taf11			-0.33											Transcription initiation factor TFIID subunit 11
taf2						-0.42							K03128	Transcription initiation factor TFIID subunit 2
taf3						-0.83								Transcription initiation factor TFIID subunit 3
taf4						0.74								Transcription initiation factor TFIID subunit 4
taf5	0.81		0.73										K03130	Transcription initiation factor TFIID subunit 5
taf6l						0.74								Transcription initiation factor TFIID subunit 6
tamm41						0.53							K17807	Mitochondrial translocator assembly and maintenance protein 41
tanc1	0.35													Ankyrin repeat domain-containing protein 50
tango2	-0.50					-0.40								
tarbp1	1.51	0.71	0.90										K15333	Trna guanosine-2'-O-methyltransferase
tardbp	0.44												K23600	TAR DNA-binding protein 43
tasp1						-0.59	Loss			Gain		Gain		Taspase, threonine aspartase, 1

tat			0.41										K00815	Tyrosine aminotransferase
tatdn1			0.39											Tatd dnase family protein
tatdn2												Loss	K03424	Tatd dnase family protein
tatdn3	-0.61	-0.46	-0.82											Tatd dnase family protein
tax1bp1	-0.40		-0.24											Tax1-binding protein 1
tax1bp3						-0.36							K24067	Tax1-binding protein 3
tbc1d13						-0.39							K24796	TBC1 domain family member 13
tbc1d14				0.48										TBC1 domain family member 14
tbc1d15	-0.50	-0.31	-0.62										K20168	TBC1 domain family member 15
tbc1d17	-0.61													TBC1 domain family member 15, TBC1 domain family member 17
tbc1d2						-0.57								TBC1 domain family member 2A
tbc1d25			-0.41											TBC1 domain family member 25
tbc1d32				0.37		0.54								
tbc1d4	-1.59	-1.15	-1.40											TBC1 domain family member 4
tbc1d5				0.39									K18469	TBC1 domain family member 5
tbc1d9				0.37									K19951	TBC1 domain family member 8/9
tbc1d9b	-0.87	-0.77	-0.88			-0.41							K19951	TBC1 domain family member 8/9
tbcb	0.31													Tubulin-specific chaperone B
tbccd1	0.45		0.61			-0.57								TBCC domain-containing protein 1
tbck						-0.44							K17544	TBC domain-containing protein kinase-like protein
tbk1	-0.53			0.31									K05410	TANK-binding kinase 1
tbkbp1	-0.82										Gain			TANK-binding kinase 1-binding protein
tbl1x						-0.45							K04508	Transducin (beta)-like 1
tbl1xr1	-0.77	-0.39	-0.67										K04508	Transducin (beta)-like 1
tbl2	0.59		0.51											Transducin beta-like protein 2
tbl3	0.31													U3 small nucleolar RNA-associated protein 13
tbp	0.44					-0.57								Transcription initiation factor TFIID TATA-box-binding protein
tbrg4	0.41													
tbx1				0.52		0.62								T-box protein 1
tbx15						-0.52					Loss		K10182	T-box protein 15
tbxas1						0.73							K01832	Thromboxane-A synthase
tc2n						0.73								Tandem C2 domains nuclear protein
tcap			-0.83											Telethonin

tceanc2	-0.51	-0.42	-0.71											
tcf19	1.09	0.54	1.04											
tcf3				-0.33		-0.49							K09063	Transcription factor E2-alpha
tcf4				-0.40		-0.35		Loss					K15603	Transcription factor 4/12
tcf7l2						-0.38							K04491	Transcription factor 7-like 2
tchp						0.59								Trichoplein keratin filament-binding protein
tcim	1.24													
tcp11l2	-2.34	-0.69	-1.67			-0.57							K25628	T-complex protein 11
tcta						0.42								
tctex1d1		-0.51	-0.65										K25421	Dynein light chain Tctex-type 5
tctn2						0.40								Tectonic-2
tdrd15		0.40		0.38										Tudor domain-containing protein 1/4/6/7
tdrd3						0.45							K18404	Tudor domain-containing protein 3
tdrkh	0.67		0.40											Tudor domain-containing protein 2
tead1			-0.56											Transcriptional enhancer factor
tead3						-0.48								Transcriptional enhancer factor
tecpr2	-0.69	-0.52	-0.86											Tectonin beta-propeller repeat-containing protein 2
tecr				0.40										Very-long-chain enoyl-coa reductase
tecta											Gain	Gain	K18273	Alpha-tectorin
tefm	0.73	0.48	0.67										K17658	Transcription elongation factor, mitochondrial
tenm2												Gain		Teneurin
tenm4											Gain			Teneurin
tent4a	0.41		0.67											Non-canonical poly(A) RNA polymerase PAPD5/7
tent5b												Gain	K23033	Terminal nucleotidyltransferase 5A/B
tent5c				-0.46										
tep1	-0.34			0.34										Telomerase protein component 1
tepsin			-0.31			-0.44								
terf2ip				-0.40		-0.74							K11113	Telomeric repeat-binding factor 2-interacting protein 1
tert			0.70										K11126	Telomerase reverse transcriptase
tes						-0.50								Testin
tesk2	-1.65	-1.17	-1.75											Testis-specific kinase 2
tespa1				0.43										
tet2	-0.47													Methylcytosine dioxygenase TET2/3

tex14						-0.99							K17540	Inactive serine/threonine-protein kinase TEX14
tex261						0.58								
tex30						0.68					Gain	Gain		Uncharacterized protein
tex9	-0.83		-0.49											
tfam				-0.41										Transcription factor A, mitochondrial
tfap2c						-0.73							K09177	Transcription factor AP-2 gamma
tfb1m	0.39												K15266	Dimethyladenosine transferase 1, mitochondrial
tfeb	-0.60		-0.61										K15590	Transcription factor EB
tfg	0.37		0.32			-0.39							K09292	Protein TFG
tfpi2						0.73								Tissue factor pathway inhibitor 2
tgfb1i1						-0.53								Transforming growth factor beta-1-induced transcript 1 protein
tgfb2						0.67							K13376	Transforming growth factor beta-2
tgfb3				-0.37										Transforming growth factor beta-3
tgfbi						-0.52								Transforming growth factor-beta-induced protein
tgfbrap1				0.43									K20177	Vacuolar protein sorting-associated protein 3
tgs1		0.41				0.51							K14292	Trimethylguanosine synthase
thada						0.66							K24169	Thyroid adenoma-associated protein
thegl				-0.53		-1.20								
thnsl1	-1.28	-0.57	-1.42				Gain				Gain	Gain	K01733	Threonine synthase
thoc1	-0.28		-0.33										K12878	THO complex subunit 1
thoc2						0.53								THO complex subunit 2
thoc3	0.64		0.48											THO complex subunit 3
thoc6	0.51		0.55											THO complex subunit 6
thrb	-0.43													
thsd7a						0.70								
thumpd1	0.60	0.32	0.34	-0.38									K06963	Trna acetyltransferase TAN1
thumpd2	0.60		0.68											
tial1						0.74							K13201	Nucleolysin TIA-1/TIAR
tiam2						-0.43								T-lymphoma invasion and metastasis-inducing protein 2
ticrr	0.69		0.67											Treslin
tifa												Loss		TRAF-interacting protein with FHA domain-containing protein A
timeless	0.88	0.52	0.58										K10997	Replication fork protection complex subunit TIMELESS/Tof1/Swi1
timm10	0.62													Mitochondrial import inner membrane translocase subunit TIM10

timm13	0.54			-0.47										Mitochondrial import inner membrane translocase subunit TIM13
timm17b						-0.48							K17795	Mitochondrial import inner membrane translocase subunit TIM17
timm21	0.35												K17796	Mitochondrial import inner membrane translocase subunit TIM21
timm22	0.68			-0.43									K17790	Mitochondrial import inner membrane translocase subunit TIM22
timm23						0.55								Mitochondrial import inner membrane translocase subunit TIM23
timm29	0.48													
timm44			-0.48										K17804	Mitochondrial import inner membrane translocase subunit TIM44
timm50	0.51												K17496	Mitochondrial import inner membrane translocase subunit TIM50
timm8b				-0.40										Mitochondrial import inner membrane translocase subunit TIM8
timmdc1	0.61										Gain		K23505	Complex I assembly factor TIMMDC1
timp4			-0.45			-0.73								Metalloproteinase inhibitor 4
tipin	0.46	0.61	0.58										K10998	Replication fork protection complex subunit TIPIN/Csm3/Swi3
tiprl			0.25											Type 2A phosphatase activator TIP41
tlcd2	-0.51		-0.56											
tlcd3a	-0.62													
tle3						-0.33							K04497	Groucho
tle4		-0.49		-0.40		-0.81								Groucho
tln1						-0.90							K06271	Talin
tln2						-0.66							K06271	Talin
tlr3	-0.51											Gain	K05401	Toll-like receptor 3
tm2d1	-0.45	-0.36												
tm2d3						-0.50								
tm7sf2	0.51		0.47											Delta14-sterol reductase
tma16						0.67							K14860	Translation machinery-associated protein 16
tmbim4				0.39									K24205	Protein lifeguard
tmbim6						0.68							K21889	Bax inhibitor 1
tmc5									Loss				K21988	Transmembrane channel-like protein
tmc6				0.48										Transmembrane channel-like protein
tmco1						0.46							K21891	Calcium load-activated calcium channel
tmco4						0.63								
tmco6						0.61								
tmed2	0.37	0.33	0.42											P24 family protein beta-1
tmed9						0.89							K20346	P24 family protein alpha

tmem104	-0.28	-0.29												
tmem106b	-0.46		-0.47				Gain							Transmembrane protein 106B
tmem116						-0.72								
tmem125								Loss	Loss		Loss			
tmem127			-0.28			-0.51							K25206	Transmembrane protein 127
tmem128						-0.50								
tmem132c												Loss		Transmembrane protein 132
tmem135						-0.90								
tmem141		-0.30					Gain			Loss		Loss		
tmem14a						0.43								
tmem150a		0.43				0.61							K21846	Transmembrane protein 150
tmem150b						-0.69								Transmembrane protein 150
tmem158						-0.80								
tmem161a	0.68													
tmem161b	0.51	0.44	0.35											
tmem164			0.51											
tmem165						-0.41							K23541	Ca2+/H+ antiporter, TMEM165/GDT1 family
tmem167a						0.41								
tmem167b						0.34								
tmem169				-0.42		-0.55								
tmem170b	0.53													
tmem175	-0.38	-0.24	-0.35					Loss	Loss		Gain	Gain		TMEM175 potassium channel family protein
tmem177	0.58					0.52								
tmem178a						-0.63								
tmem184c	-0.90	-0.52	-0.79										K14360	Organic solute transporter subunit alpha
tmem19		0.40												
tmem192	-0.57	-0.54	-0.59	0.42										
tmem200a			-0.61								Loss			
tmem203			0.54											
tmem208	0.43		0.34											
tmem209	0.47	0.42	0.49											
tmem214			0.40											
tmem218						0.55								

tmem220	-0.70													
tmem221						0.71								
tmem229b									Loss		Loss	Loss		
tmem233	-0.49					0.47								
tmem234	-0.59		-0.56											
tmem237				-0.43		-0.87							K22765	Transmembrane protein 237
tmem242	0.61					0.65								
tmem245	0.39										Loss	Loss		
tmem250						0.69						Gain		
tmem254						0.32								
tmem255a										Gain	Gain	Gain		
tmem258	0.45													
tmem26	-0.45		-0.40											
tmem267						-0.65								
tmem268	-0.42													
tmem269						0.54								
tmem271							Loss				Loss	Loss		
tmem33	0.62												K20724	Transmembrane protein 33
tmem35a				-0.54										
tmem39a	0.58	0.54	0.49											
tmem41b						-0.58								
tmem50a						0.72								
tmem51						-0.59								
tmem60						-0.73								
tmem62	0.71													
tmem64						-0.43								
tmem65			0.44											
tmem69	0.51										Gain	Gain		
tmem70	0.35												K17966	Transmembrane protein 70, mitochondrial
tmem71						0.73								
tmem86a	-1.33	-0.95	-1.51										K18575	Alkenylglycerophosphocholine hydrolase
tmem87a				0.35										
tmem87b	0.29		0.31											

tmem8a				0.40										Transmembrane protein 8A/B
tmem94			-0.38										K25291	Transmembrane protein 94
tmem9b						-0.44								
tmf1	-0.37												K20286	TATA element modulatory factor
tmigd1						0.43								
tmprss15							Loss	Loss					K01316	Enterokinase
tmprss5											Loss	Loss	K09636	Transmembrane protease serine 5
tmprss9	0.62													Transmembrane protease serine 9
tmub2						-0.58	Loss	Loss						Transmembrane and ubiquitin-like domain-containing protein
tnfaip1						0.39							K15074	BTB/POZ domain-containing adapter for CUL3-mediated rhoa degradation protein
tnfrsf12a						-0.60								Tumor necrosis factor receptor superfamily member 12A
tnip1	-0.56		-0.29										K23829	TNFAIP3-interacting protein 1
tnip2						-0.59								
tnk2		-0.52											K08886	Activated CDC42 kinase 1
tnn						-0.79								Tenascin
tnpo2	0.71	0.53	0.36			-0.40							K18727, K18752	Transportin-1, transportin-2
tnr											Loss	Loss		Tenascin
tnrc6a	-0.37					-0.65								Trinucleotide repeat-containing gene 6 protein
tns1		-0.43	-0.50			-0.44							K18080	Tensin
tob1			-0.52			-0.74							K14443	Protein Tob/BTG
toe1	0.33	0.33												Target of EGR1 protein 1
togaram1			-0.42			-0.69								TOG array regulator of axonemal microtubules protein
tollip	-0.43												K05402	Toll-interacting protein
tomm22	0.61												K17769	Mitochondrial import receptor subunit TOM22
tomm7	0.37													Mitochondrial import receptor subunit TOM7
tomm70	0.42												K17768	Mitochondrial import receptor subunit TOM70
tonsl	0.75		0.52										K09257	NF-kappa-B inhibitor-like protein 2
top1	0.42	0.37	0.34										K03163	DNA topoisomerase I
top2b	0.53	0.48	0.56										K03164	DNA topoisomerase II
top3a	0.90	0.68	0.85											DNA topoisomerase III
top3b						0.64								DNA topoisomerase III
tor2a	-0.41		-0.36											Torsin-2A

tor4a	-0.56													
tox2						-0.79								
tp53bp1	0.40		0.45											Tumor suppressor p53-binding protein 1
tp53bp2				0.44									K16823	Apoptosis-stimulating of p53 protein 2
tp53i11						0.45					Gain	Gain		
tp53inp1	-0.99	-0.36	-0.91											Tumor protein p53-inducible nuclear protein 1
tp53inp2	-0.79		-0.54											Tumor protein p53-inducible nuclear protein 2
tpbg										Loss		Loss		
tpd52l1	-0.60	-0.39	-0.52											
tpgs2						0.48								Tubulin polyglutamylase complex subunit 2
tpmt							Loss							Thiopurine S-methyltransferase
tpp1	-1.21	-0.80	-1.34										K01279	Tripeptidyl-peptidase I
tppp	-0.49													Tubulin polymerization-promoting protein
tpr	0.45												K09291	Nucleoprotein TPR
tpra1	-0.32		-0.33											Transmembrane protein adipocyte-associated 1
tprkb						0.82								EKC/KEOPS complex subunit TPRKB/CGI121
tpx2	0.47		0.58											Targeting protein for Xklp2
tra2a	0.60		0.48										K12897	Transformer-2 protein
traf3	-0.40		-0.47										K03174	TNF receptor-associated factor 3
trafd1						-0.51						Loss		
traip	0.45		0.41			-0.59							K11985	TRAF-interacting protein
tram2	0.66	0.65	0.83											Translocating chain-associated membrane protein 1
trap1	0.48					0.55							K04079, K09488	Molecular chaperone htpg, TNF receptor-associated protein 1
trappc10				0.39		0.43								Trafficking protein particle complex subunit 10
trappc11						-0.36							K20308	Trafficking protein particle complex subunit 11
trappc13	-0.30	-0.37	-0.31											Trafficking protein particle complex subunit 13
trappc3	0.34		0.27										K20302	Trafficking protein particle complex subunit 3
trappc4												Gain		Trafficking protein particle complex subunit 4
trdn	-0.47												K23449	Triadin
trib2						-0.54							K08814	Tribbles homolog 1/2
trim32	-0.47													Tripartite motif-containing protein 32
trim36	0.61												K12013	Tripartite motif-containing protein 36

trim37	0.42											Loss		Tripartite motif-containing protein 37
trim45			0.38										K12021	Tripartite motif-containing protein 45
trim8				0.40									K12001	Tripartite motif-containing protein 8
triobp	-0.35	-0.29	-0.33			-0.39							K23751	TRIO and F-actin-binding protein
trip10						-0.30								Thyroid hormone receptor interactor 10
trip12	-0.31												K10590	E3 ubiquitin-protein ligase TRIP12
trip13	0.66		0.46											Pachytene checkpoint protein 2
trit1	0.50												K00791	Trna dimethylallyltransferase
trmt10c	0.61	0.42					Loss						K17654	Mitochondrial ribonuclease P protein 1
trmt11	0.63		0.56			0.33							K15430	Trna (guanine10-N2)-methyltransferase
trmt13						0.45							K15446	Trna:m4x modification enzyme
trmt2b	0.49	0.42												Trna (uracil-5-)-methyltransferase
trmt44						0.63							K15447	Trnaser (uridine44-2'-O)-methyltransferase
trmt6	0.73		0.40											Trna (adenine58-N1)-methyltransferase non-catalytic subunit
trmt61a				-0.51		-0.42							K07442	Trna (adenine57-N1/adenine58-N1)-methyltransferase catalytic subunit
trmt61b	0.57												K07442	Trna (adenine57-N1/adenine58-N1)-methyltransferase catalytic subunit
trmt9b												Loss		
trrap	0.46		0.41										K08874	Transformation/transcription domain-associated protein
tsc2	-0.44												K07207	Tuberous sclerosis 2
tsc22d2										Loss		Loss		
tsc22d3						-0.74								
tsen15						0.64							K15324	Trna-splicing endonuclease subunit Sen15
tsen34	1.36	0.72	0.98											Trna-splicing endonuclease subunit Sen34
tsen54				0.46										Trna-splicing endonuclease subunit Sen54
tsfm	0.55		0.42	-0.42									K02357	Elongation factor Ts
tshz1						-0.78							K09236	Teashirt
tshz2				-0.48		-1.11							K09236	Teashirt
tshz3				-0.43		-0.76							K09236	Teashirt
tsnaxip1						-0.73								
tspan12	-0.37												K17355	Tetraspanin-12
tspan14				0.39										Tetraspanin-14
tspan17	-0.40									Gain			K10303	F-box protein 23

tspan3	-0.43		-0.72	-0.36									Tetraspanin-3
tspan7		-0.38	-0.49										Tetraspanin-7
tspo	-0.40											K05770	Translocator protein
tsr2	0.67												Pre-rrna-processing protein TSR2
tstd2	0.69	0.40	0.56										UPF0176 protein
tstd3			-0.55										Thiosulfate:glutathione sulfurtransferase
ttc12				0.40		0.50						K24652	Tetratricopeptide repeat protein 12
ttc13						0.85							
ttc19			-0.31	-0.35									Tetratricopeptide repeat protein 19, mitochondrial
ttc23	-0.79		-0.53				Loss	Loss				K24934	Tetratricopeptide repeat protein 23
ttc27	0.65											K24935	Tetratricopeptide repeat protein 27
ttc28						-0.65						K24936	Tetratricopeptide repeat protein 28
ttc36			0.47										Tetratricopeptide repeat protein 36
ttc37				0.37			Loss		Loss			K12600	Superkiller protein 3
ttc39b		0.71											Tetratricopeptide repeat protein 39
ttc5	0.42												Tetratricopeptide repeat protein 5
ttf2	0.41		0.49										Transcription termination factor 2
tti1	0.47									Loss	Loss	K20403	TELO2-interacting protein 1
ttk			0.47										Serine/threonine-protein kinase TTK/MPS1
ttpa	0.53		0.54								Gain	K24455	Alpha-tocopherol transfer protein
ttpal						-0.40							Retinaldehyde-binding protein 1
tub						-0.68						K19600	Tubby and related proteins
tubd1						-0.80						K10390	Tubulin delta
tubg1	0.42		0.43									K10389	Tubulin gamma
tubgcp3						0.54						K16570	Gamma-tubulin complex component 3
tubgcp6	0.56		0.55									K16573	Gamma-tubulin complex component 6
tuft1	-0.51		-0.67										
tut1	1.29	0.45	0.92										Speckle targeted PIP5K1A-regulated poly(A) polymerase
tut4	-0.78	-0.34	-0.74			-0.36	Gain	Gain					Terminal uridylyltransferase
twist1						-0.73						K09069	Twist
twistnb						0.55							DNA-directed RNA polymerase I subunit RPA43
twnk	0.77			-0.66								K17680	Twinkle protein
txlnb	-0.48		-0.53										

txndc11						0.72								
txndc15						0.49							K25389	Thioredoxin domain-containing protein 15
txndc5	0.43													Thioredoxin domain-containing protein 5
txndc9						0.45								
txnip	-0.89		-0.53										K20910	Thioredoxin-interacting protein
txnl4a	0.40			-0.45									K12859	U5 snrnp protein, DIM1 family
txnl4b			-0.35										K12859	U5 snrnp protein, DIM1 family
txnrd2	0.50													Thioredoxin reductase (NADPH)
tyk2	-0.56	-0.44	-0.54	0.34										Non-receptor tyrosine-protein kinase TYK2
tyms	0.65		0.91										K00560	Thymidylate synthase
tyro3	-0.48												K05116	TYRO3 protein tyrosine kinase 3
tyrobp	0.52					-0.48								
tysnd1	-0.58		-0.35											Peroxisomal leader peptide-processing protease
u2af2		0.26	0.28										K12837	Splicing factor U2AF 65 kda subunit
u2surp	0.50													U2-associated protein SR140
uba2	0.39	0.38	0.57										K10685	Ubiquitin-like 1-activating enzyme E1 B
uba6	0.54	0.38	0.45										K10699	Ubiquitin-activating enzyme E1-like protein 2
ubac2	0.44													Ubiquitin-associated domain-containing protein 2
ubap1l												Loss		
ubap2	0.32		0.31											
ubap2l						-0.66								
ube2a			-0.40			0.36							K10573, K10574	Ubiquitin-conjugating enzyme E2 A , ubiquitin-conjugating enzyme E2 B
ube2b						-0.42	Gain	Gain					K10573, K10574	Ubiquitin-conjugating enzyme E2 A , ubiquitin-conjugating enzyme E2 B
ube2c			0.44										K06688	Ubiquitin-conjugating enzyme E2 C
ube2i			0.53										K10577	Ubiquitin-conjugating enzyme E2 I
ube2j2	-0.34		-0.33										K04554	Ubiquitin-conjugating enzyme E2 J2
ube2k						-0.40								Ubiquitin-conjugating enzyme (huntingtin interacting protein 2)
ube2l3			0.34										K04552	Ubiquitin-conjugating enzyme E2 L3
ube2m						0.35								Ubiquitin-conjugating enzyme E2 M
ube2r2	-0.72		-0.55											Ubiquitin-conjugating enzyme E2 R
ube2s				-0.33		-0.37							K10583	Ubiquitin-conjugating enzyme E2 S
ube3d	1.48	0.78	1.19										K20803	Ubiquitin-protein ligase E3 D

ube4a						-0.40							K10596	Ubiquitin conjugation factor E4 A
ubfd1						0.50								
ubl3				-0.36		-0.90								
ublcp1	0.83		0.71								Loss		K17618	Ubiquitin-like domain-containing CTD phosphatase 1
ubox5			0.52										K10600	U-box domain-containing protein 5
ubp1			-0.38										K09275	Transcription factor CP2 and related proteins
ubqln4						-0.36								Ubiquilin
ubr2				0.55		0.50								E3 ubiquitin-protein ligase UBR2
ubr7				0.42		0.73							K11979	E3 ubiquitin-protein ligase UBR7
ubxn1						0.47								UBX domain-containing protein 1/4
ubxn2a				-0.39									K24349	UBX domain-containing protein 2A
ubxn6	-0.54	-0.42	-0.56											UBX domain-containing protein 6
ubxn7			-0.51										K24350	UBX domain-containing protein 7
uchl5						0.27								Ubiquitin carboxyl-terminal hydrolase L5
uck1	-0.70		-0.64			-0.42								Uridine kinase
uckl1	-0.58		-0.52	-0.50		-0.94							K00876	Uridine kinase
ugcg				-0.56		-0.46								Ceramide glucosyltransferase
ugdh	0.70	0.69	0.89											Udpglucose 6-dehydrogenase
uggt1			0.43										K11718	UDP-glucose:glycoprotein glucosyltransferase
uhrf1	0.80	0.75	0.65			-0.41							K10638	E3 ubiquitin-protein ligase UHRF1
uimc1	-0.53					0.51							K20775	BRCA1-A complex subunit RAP80
umad1			-0.34											
unc119						-1.03							K23539	Protein unc-119
unc13a	0.83					0.59							K15293	Protein unc-13 A/B/C
unc13d				0.47										Protein unc-13 D
unc45a						-0.82							K21991	Protein unc-45
unc45b				-0.62		-0.58								Protein unc-45
unc5b						-0.62		Loss	Loss			Loss		Netrin receptor unc-5
unc5c							Gain						K07521	Netrin receptor unc-5
uncx							Gain		Gain				K09328	Homeobox protein Unc-4
ung	0.40		0.42											Uracil-DNA glycosylase
unk	-0.43			0.55									K23048	RING finger protein unkempt
unkl	-1.52	-0.55	-1.40			-0.52							K23047	E3 ubiquitin-protein ligase UNKL

upf1	0.31			0.30		0.35							K14326	Regulator of nonsense transcripts 1
upf2	-0.62		-0.61	0.38							Gain		K14327	Regulator of nonsense transcripts 2
uprt						0.47								
urb1	0.59													Nucleolar pre-ribosomal-associated protein 1
urb2	0.93	0.47	0.57										K14862	Nucleolar pre-ribosomal-associated protein 2
urod												Gain	K01599	Uroporphyrinogen decarboxylase
usb1	-0.61	-0.54	-0.76										K23093	U6 snrna phosphodiesterase
uso1	0.40		0.28										K20361	Intracellular protein transport protein USO1
usp1		0.30	0.38										K11832	Ubiquitin carboxyl-terminal hydrolase 1
usp13			-0.49										K11836	Ubiquitin carboxyl-terminal hydrolase 5/13
usp14						0.43							K11843	Ubiquitin carboxyl-terminal hydrolase 14
usp15												Gain		Ubiquitin carboxyl-terminal hydrolase 15
usp16			0.39											Ubiquitin carboxyl-terminal hydrolase 16/45
usp20	-0.52													Ubiquitin carboxyl-terminal hydrolase 20/33
usp24						0.36								Ubiquitin carboxyl-terminal hydrolase 9/24
usp25	-0.56		-0.60			0.64							K11849	Ubiquitin carboxyl-terminal hydrolase 25
usp3						-0.47								Ubiquitin carboxyl-terminal hydrolase 3
usp30	-0.28												K11851	Ubiquitin carboxyl-terminal hydrolase 30
usp31				0.38									K11852	Ubiquitin carboxyl-terminal hydrolase 31
usp32	-0.82	-0.65	-0.80											Ubiquitin carboxyl-terminal hydrolase 6/32
usp33	0.48		0.31										K11848	Ubiquitin carboxyl-terminal hydrolase 20/33
usp37	0.49		0.52			0.50							K11850	Ubiquitin carboxyl-terminal hydrolase 26/29/37
usp39			0.51										K12847	U4/U6.U5 tri-snrnp-associated protein 2
usp42	0.40					-0.64							K11855	Ubiquitin carboxyl-terminal hydrolase 36/42
usp45	-0.54		-0.54										K11844	Ubiquitin carboxyl-terminal hydrolase 16/45
usp47						0.48								Ubiquitin carboxyl-terminal hydrolase 47
usp49						-0.39								Ubiquitin carboxyl-terminal hydrolase 44/49
usp5	0.39		0.36											Ubiquitin carboxyl-terminal hydrolase 5/13
usp6nl						-0.41							K20133	USP6 N-terminal-like protein
usp7	0.27	0.28	0.24			-0.21							K11838	Ubiquitin carboxyl-terminal hydrolase 7
usp8	-0.35		-0.39										K11839	Ubiquitin carboxyl-terminal hydrolase 8
uspl1	0.75		0.52										K23008	SUMO-specific isopeptidase USPL1
ust				-0.35		-0.33							K03193	Dermatan/chondrotin sulfate uronyl 2-O-sulfotransferase UST

utp15	0.52			-0.38										U3 small nucleolar RNA-associated protein 15
utp18						0.62								U3 small nucleolar RNA-associated protein 18
utp20	0.43													U3 small nucleolar RNA-associated protein 20
utp25						0.54								U3 small nucleolar RNA-associated protein 25
utp3	1.10	0.44	0.57			0.44							K14767	U3 small nucleolar RNA-associated protein 3
utp4	0.45					0.48								U3 small nucleolar RNA-associated protein 4
utp6	0.37			-0.34										U3 small nucleolar RNA-associated protein 6
uvssa						-0.72								UV-stimulated scaffold protein A
vac14						-0.52							K15305	Vacuole morphology and inheritance protein 14
vamp7	-0.63	-0.36	-0.63											Vesicle-associated membrane protein 7
vapa						-0.30							K06096	Vesicle-associated membrane protein-associated protein A
vars	0.73													Valyl-trna synthetase
vars2	0.50					0.53							K01873	Valyl-trna synthetase
vasp						-0.33							K06274	Vasodilator-stimulated phosphoprotein
vav1				0.39		0.65								Guanine nucleotide exchange factor VAV
vav3				0.43		0.61							K05730	Guanine nucleotide exchange factor VAV
vax2												Loss		Homeobox protein ventral anterior
vcl	0.35					-0.37							K05700	Vinculin
vcp				0.40		0.60								Transitional endoplasmic reticulum atpase
vcpkmt	0.49												K21806	Protein N-lysine methyltransferase METTL21D
vegfa				-0.40									K05448	Vascular endothelial growth factor A
vill						-0.79								
vipas39	-0.67	-0.67	-0.43										K23287	VPS33B-interacting protein in polarity and apical restriction
vma21						-0.39								Vacuolar atpase assembly integral membrane protein VMA21
vmac						-0.45								
vps13a				0.42									K19525	Vacuolar protein sorting-associated protein 13A/C
vps13c						-0.64							K19525	Vacuolar protein sorting-associated protein 13A/C
vps13d				0.43										Vacuolar protein sorting-associated protein 13D
vps16	-0.60													Vacuolar protein sorting-associated protein 16
vps26a				-0.29		-0.36							K18466	Vacuolar protein sorting-associated protein 26A/B
vps28	-0.47					-0.76								ESCRT-I complex subunit VPS28
vps33a	-0.57		-0.39			0.39							K20182	Vacuolar protein sorting-associated protein 33A
vps35	-0.34												K18468	Vacuolar protein sorting-associated protein 35

vps35l						-0.52								K25731	VPS35 endosomal protein-sorting factor-like
vps36						0.32									ESCRT-II complex subunit VPS36
vps37b						-0.43								K12185	ESCRT-I complex subunit VPS37
vps39				0.49											Vam6/Vps39-like protein vacuolar protein sorting-associated protein 39
vps41	-0.47														Vacuolar protein sorting-associated protein 41
vps4a	-0.56		-0.41											K12196	Vacuolar protein-sorting-associated protein 4
vps50													Loss	K23288	Syndetin
vps51			0.51												Vacuolar protein sorting-associated protein 51
vps52		-0.43													Vacuolar protein sorting-associated protein 52
vps54	0.62	0.41	0.47												Vacuolar protein sorting-associated protein 54
vps9d1						-0.64									
vrk1			0.41											K08816	Vaccinia related kinase
vrk2				0.40										K08816	Vaccinia related kinase
vsir	-0.54		-0.82											K23268	V-type immunoglobulin domain-containing suppressor of T-cell activation
vstm4						0.57									
vta1	-0.35														Vacuolar protein sorting-associated protein VTA1
vti1b	-0.64	-0.38	-0.73	0.49										K08493	Vesicle transport through interaction with t-snares 1
vwc2l							Loss		Loss		Gain	Gain			Brorin
wac	-0.46		-0.33											K23884	WW domain-containing adapter protein with coiled-coil
wapl		0.23												K25163	Wings apart-like protein
wars1	0.60		0.35												Tryptophanyl-trna synthetase
wars2	0.63														Tryptophanyl-trna synthetase
washc3	-0.65	-0.51	-0.69			0.49									WASH complex subunit CCDC53
washc4	-0.43		-0.51												WASH complex subunit 7
washc5	-0.53	-0.39	-0.38											K18464	WASH complex subunit strumpellin
wbp11	0.70	0.57	0.65												WW domain-binding protein 11
wbp2nl	-0.77		-0.49	0.36										K22532	Postacrosomal sheath WW domain-binding protein
wbp4						-0.87									WW domain-binding protein 4
wdcp			-0.52												
wdfy1						-0.27								K23299	WD repeat and FYVE domain-containing protein 1
wdfy3			-0.23			-0.24								K22262	WD repeat and FYVE domain-containing protein 3
wdfy4				0.54											WD repeat and FYVE domain-containing protein 4

wdr1			0.42										K24736	WD repeat-containing protein 1 (actin-interacting protein 1)
wdr12	0.76					0.43							K14863	Ribosome biogenesis protein
wdr27						0.60								
wdr35	0.57													WD repeat-containing protein 35
wdr36	0.42													U3 small nucleolar RNA-associated protein 21
wdr37	-0.45	-0.30	-0.41	0.38									K24744	WD repeat-containing protein 37
wdr41	-0.71	-0.66	-0.75			-0.66								WD repeat-containing protein 41
wdr43	0.29												K14546	U3 small nucleolar RNA-associated protein 5
wdr44	-0.64	-0.30	-0.33											WD repeat-containing protein 44
wdr45	-1.51	-0.83	-1.52											WD repeat-containing protein 45
wdr46						0.33								U3 small nucleolar RNA-associated protein 7
wdr53						0.85								WD repeat-containing protein 53
wdr55	0.51			-0.39									K24750	WD repeat-containing protein 55
wdr59	-0.40		-0.46										K20409	SEA/GATOR complex protein SEA3/WDR59
wdr61			0.35										K12602	WD repeat-containing protein 61
wdr77	0.57		0.50										K13221	Methylosome protein 50
wdr81	-0.45		-0.52			-0.76								WD repeat-containing protein 81
wdr83	0.76		0.47										K13124	Mitogen-activated protein kinase organizer 1
wdr83os			0.39										K24963	PAT complex subunit Asterix
wdr91	-0.79	-0.51	-0.52	0.40									K24760	WD repeat-containing protein 91
wdtc1	-0.47		-0.68										K11807	WD and tetratricopeptide repeats protein 1
wee1			0.34										K06632	Wee1-like protein kinase
whamm	-0.78	-0.68	-1.01											WASP homolog-associated protein with actin, membranes and microtubules
wif1						-1.29								WNT inhibitory factor 1
wipf1	0.49						Loss						K19475	WAS/WASL-interacting protein
wipf3						-0.65							K19475	WAS/WASL-interacting protein
wipi1	-0.61													Autophagy-related protein 18
wls						-0.44								
wnk3	-0.51					-0.54								WNK lysine deficient protein kinase
wnt3a						-0.77							K00312	Wingless-type MMTV integration site family, member 3
wrap53	0.42												K23314	Telomerase Cajal body protein 1
wsb1				0.40		0.34								WD repeat and SOCS box-containing protein 1

wsb2	-0.49			0.50										WD repeat and SOCS box-containing protein 2
wscd2						-0.68								
wt1	-1.11		-1.33											Wilms tumor protein 1
wtap				-0.35		-0.45							K22824	Pre-mrna-splicing regulator WTAP
wwc3						0.62							K16685	Protein KIBRA
wwtr1	-0.80	-0.41	-0.79										K16820	WW domain-containing transcription regulator protein 1
xpc	0.79	0.80	0.96			0.52								Xeroderma pigmentosum group C-complementing protein
xpo4						-0.75								Exportin-4
xpo5						-0.82							K14289	Exportin-5
xpo7	0.52	0.30	0.50											Exportin-7
xpot	0.76													Exportin-T
xrcc1			0.34										K10803	DNA-repair protein XRCC1
xrcc2	-0.65	-0.32	-0.39											DNA-repair protein XRCC2
xrcc4						0.48							K10886	DNA-repair protein XRCC4
xrn1						-0.39								5'-3' exoribonuclease 1
xxylt1				-0.41		-0.64								Xylosyl alpha-1,3-xylosyltransferase
xylb	-0.44	-0.37											K00854	Xylulokinase
yae1							Loss		Loss			Gain		
yaf2	0.75					-0.51								YY1-associated factor 2
yap1						-0.75							K16687	Transcriptional coactivator YAP1
yars2	0.84		0.44	-0.48									K01866	Tyrosyl-trna synthetase
ybey	0.71													Probable rrna maturation factor
ydjc	0.50													
yeats4	0.41		0.40									Gain		YEATS domain-containing protein 4
yif1a				-0.35		-0.33								Protein transport protein YIF1
yipf3	-0.31												K22941	Protein YIPF3
yipf5	0.34		0.32											Protein YIPF5/7
yipf6	-0.31													Protein YIPF6
yju2			0.37											
ylpm1	0.66	0.34	0.64											YLP motif-containing protein 1
yme1l1			0.34											ATP-dependent metalloprotease
yod1			0.37										K13719	Ubiquitin thioesterase OTU1
yrdc	1.02												K07566	L-threonylcarbamoyladenylate synthase

ythdc1						-0.33							K20100	YTH domain-containing protein 1
ywhab				0.41										14-3-3 protein beta/theta/zeta
yy1	-0.35													Transcription factor YY
zadh2			0.41										K07119	Prostaglandin reductase 3
zbed4						-0.99								Zinc finger BED domain-containing protein 4
zbtb1	-0.31		-0.42			-0.50								Zinc finger and BTB domain-containing protein 1
zbtb12			-0.50			-0.54								Zinc finger and BTB domain-containing protein 12
zbtb17						-0.78								General transcription factor IIIA, zinc finger and BTB domain-containing protein 17
zbtb18	0.38													Zinc finger and BTB domain-containing protein 18/42
zbtb2	-0.80		-0.82											Zinc finger and BTB domain-containing protein 2
zbtb20	-0.61		-0.40										K10501	Zinc finger and BTB domain-containing protein 20
zbtb21			-0.35			-0.68	Loss							Zinc finger and BTB domain-containing protein 21
zbtb24						-0.82					Gain		K09191, K10503	General transcription factor IIIA, zinc finger and BTB domain-containing protein 24
zbtb32								Gain			Gain	Gain		
zbtb38	-0.55	-0.41	-0.48	-0.56		-1.23							K10510	Zinc finger and BTB domain-containing protein 38
zbtb41	-0.43		-0.37											General transcription factor IIIA, KRAB domain-containing zinc finger protein, zinc finger and BTB domain-containing protein 41
zbtb46	-0.48		-0.42	-0.39										
zc3h12b									Gain	Loss	Loss	Loss	K18668	Ribonuclease ZC3H12
zc3h13							Gain	Gain					K22909	Zinc finger CCCH domain-containing protein 13
zc3h14			0.50											Zinc finger CCCH domain-containing protein 14
zc3h15						0.43								
zc3h18			0.29											Nuclear protein NHN1
zc3h7a						-0.50							K24945	Zinc finger CCCH domain-containing protein 7
zc3hc1						-0.44								
zc4h2						-0.66								Zinc finger C4H2 domain-contaning protein
zcchc10	0.49		0.43											
zcchc8	0.31		0.41											Zinc finger CCHC domain-containing protein 8
zcchc9				-0.45		-0.47							K17578	Zinc finger CCHC domain-containing protein 9
zdhhc15						0.42								Palmitoyltransferase ZDHHC2/15/20
zdhhc17						0.52								Palmitoyltransferase ZDHHC13/17
zdhhc22						-0.62								

zdhhc3	0.36		0.35											Palmitoyltransferase ZDHHC3/7/25
zdhhc6						0.29								Palmitoyltransferase ZDHHC6
zdhhc8						0.63								Palmitoyltransferase ZDHHC5/8
zer1	-0.53		-0.34			-0.61							K10350	Zyg-11 protein
zfand4						-0.65								AN1-type zinc finger protein 4
zfand5	-0.49	-0.34	-0.88											AN1-type zinc finger protein 5/6
zfand6						-0.46								AN1-type zinc finger protein 5/6
zfc3h1		0.35				0.40								
zfhx4						-0.49								Zinc finger homeobox protein 4
zfyve1	-2.13	-1.72	-2.47										K17603	Zinc finger FYVE domain-containing protein 1
zfyve19				0.44		0.47								Abscission/nocut checkpoint regulator
zfyve26		-0.33	-0.28										K19027	Zinc finger FYVE domain-containing protein 26
zfyve27						-0.58							K19368	Protrudin
zfyve28						-0.46							K24780	Lateral signaling target protein 2
zgpat			0.34									Loss		
zic1						-0.68								Zinc finger protein ZIC 1
zic2				-0.60		-1.45								Zinc finger protein ZIC 2
zmat5				-0.52		-0.71								U11/U12 small nuclear ribonucleoprotein 20 kda protein
zmpste24			0.31	-0.31										STE24 endopeptidase
zmym4	0.41	0.33	0.33											Zinc finger MYM-type protein 2/3/4
zmynd10						-0.68								Zinc finger MYND domain-containing protein 10
zmynd12												Gain		
zmynd19						0.60							K24632	Zinc finger MYND domain-containing protein 19
znf131						-0.55							K24806	Zinc finger and BTB domain-containing protein 35
znf148						-0.43							K24370	Zinc finger protein 148
znf207	0.45		0.46											
znf236	0.44		0.42			-0.49							K09191, K09228	General transcription factor IIIA, KRAB domain-containing zinc finger protein
znf276				0.35										
znf318	0.46		0.48											
znf330				-0.38										
znf346				-0.34		-0.43							K10137	Zinc finger, matrin type 3
znf367	0.47	0.43	0.61	-0.51		-0.78								

znf395	-0.98		-2.46											
znf407						-1.03								
znf451			-0.43										K25176	E3 SUMO-protein ligase ZNF451
znf503	-0.58					-0.53								
znf507						-0.59								
znf593						0.43								Bud site selection protein 20
znf598	0.47			0.34		0.49								E3 ubiquitin-protein ligase ZNF598
znf618						-0.35								
znf622	0.53												K14816	Pre-60S factor REI1
znf628						0.63								General transcription factor IIIA, KRAB domain-containing zinc finger protein
znf644							Loss	Loss					K24374	Zinc finger protein 644
znf652				-0.30		-0.35							K09191, K10518	General transcription factor IIIA, zinc finger and BTB domain-containing protein 47
znf704	-1.14	-0.69	-1.07											
znf706				-0.56		-1.28								
znf827						0.62								
znhit1						0.56							K11663	Zinc finger HIT domain-containing protein 1
znrf3						-0.85								E3 ubiquitin-protein ligase ZNRF3
zpld1								Gain						
zpr1	0.46										Loss	Loss	K06874	Zinc finger protein
zranb2			0.28										K26076	Zinc finger Ran-binding domain-containing protein 2
zrsr2	0.36		0.34										K24273	U2 small nuclear ribonucleoprotein auxiliary factor 35 kda subunit-related protein
zswim7	0.67										Gain		K25770	Zinc finger SWIM domain-containing protein 7
zwilch	0.65		0.66										K11579	Protein zwilch
zyg11b	-1.15	-0.41	-1.20											Zyg-11 protein
zzef1						0.52								
aar2	0.48	0.43	0.47											A1 cistron-splicing factor AAR2
aarsd1	-0.43													Misacylated trna(Ala) deacylase
aasdh						0.87							K00142	Acyl-coa synthetase
aass	0.58	0.31				0.33							K14157	Alpha-aminoadipic semialdehyde synthase
