# Supplementary material for: Intergenerational effects of early life-stage temperature modulation on gene expression and DNA methylation in Atlantic cod (Gadus morhua)
Source: Epigenetics. 2023 Jul 27;18(1):2237759. doi: 10.1080/15592294.2023.2237759 (PMC10376914; doi:10.1080/15592294.2023.2237759)
Supplement: Supplemental Material [file KEPI_A_2237759_SM3549.zip › Supplementary files/Supplementary_table_3.docx]

Supplementary table 3. Genes with differential expression and differential methylation.

| **Gene (gadMor3.0)** | **Generation/treatment** | | | **Annotation (KEGG)** | **Pathway (KEGG)** |  |
| --- | --- | --- | --- | --- | --- | --- |
| abcb6 | F1 T7 |  |  | ATP-binding cassette, subfamily B (MDR/TAP), member 6 |  |  |
| akap10 | F1 T7 |  |  | A-kinase anchor protein 10 |  |  |
| atad2b | F1 T7 |  |  | ATPase family AAA domain-containing protein 2 |  |  |
| atxn2l | F1 T7 |  |  | Ataxin 2/2L |  |  |
|  |  |  |  |  | MicroRNAs in cancer/Axon |  |
| bmpr2 | F1 T7 |  |  | Bone morphogenetic protein receptor type-2 | guidance/Signaling pathways regulating |  |
|  |  |  |  |  | pluripotency of stem cells |  |
| c1h1orf174 | F1 T7 |  |  |  |  |  |
| cactin | F1 T7 |  |  | Cactin |  |  |
| camsap3 | F1 T1 |  |  | Calmodulin-regulated spectrin-associated protein |  |  |
| cdc6 | F0 T7 |  |  | Cell division control protein 6 |  |  |
| cemip2 | F1 T7 |  |  | Cell surface hyaluronidase |  |  |
| cers6 | F1 T7 |  |  | Sphingoid base N-palmitoyltransferase |  |  |
| cftr | F1 T7 |  |  | Cystic fibrosis transmembrane conductance regulator |  |  |
| ciao2b | F1 T7 |  |  |  |  |  |
| crybb1 | F1 T7 |  |  | Beta-crystallin |  |  |
| dennd2c | F1 T4 |  |  | DENN domain-containing protein 2 |  |  |
| des | F1 T7 |  |  | Desmin |  |  |
| diablo | F1 T7 |  |  | Diablo |  |  |
| dnmt3a | F1 T7 |  |  | DNA (cytosine-5)-methyltransferase 3A | MicroRNAs in cancer |  |
| dyrk2 | F0 T1 |  |  | Dual specificity tyrosine-phosphorylation-regulated kinase |  |  |
| e2f3 | F0 T1 | F0 T4 | F0 T7 | Transcription factor E2F3 | Pathways in cancer/MicroRNAs in cancer |  |
| emid1 | F1 T7 |  |  | EMI domain-containing protein 1 |  |  |
| ephb4 | F1 T7 |  |  | Eph receptor B4 | Axon guidance |  |
| epyc | F1 T4 | F1 T7 |  | Epiphycan |  |  |
| exosc10 | F1 T7 |  |  | Exosome complex exonuclease RRP6 |  |  |
| fbxl22 | F1 T7 |  |  | F-box and leucine-rich repeat protein 22 |  |  |
| fdxr | F0 T1 |  |  | Adrenodoxin-NADP+ reductase/Ferredoxin/flavodoxin---NADP+ |  |  |
|  |  |  |  | reductase |  |  |
|  |  |  |  |  |  |  |
| frmd4a | F1 T7 |  |  | FERM domain-containing protein 4 |  |  |
| git1 | F1 T7 |  |  | Gprotein-coupled receptor kinase interactor 1 |  |  |

| gla | F1 T7 |  |  | Alpha-galactosidase |  |  |
| --- | --- | --- | --- | --- | --- | --- |
| gpatch2 | F0 T1 | F0 T7 |  | Gpatch domain-containing protein 2 |  |  |
| gpd2 | F1 T7 |  |  | Glycerol-3-phosphate dehydrogenase |  |  |
| grem2 | F1 T7 |  |  | Gremlin |  |  |
| gtpbp2 | F0 T1 | F0 T4 |  | GTP-binding protein 2 |  |  |
| hnf1a | F1 T4 |  |  | Transcription factor 1, hepatocyte nuclear factor 1-alpha |  |  |
| LOC115528926 | F1 T7 |  |  | Claudin |  |  |
| LOC115529367 | F1 T7 |  |  |  |  |  |
| LOC115529662 | F0 T1 |  |  |  |  |  |
| LOC115529749 | F1 T7 |  |  | Solute carrier family 29 (equilibrative nucleoside transporter), |  |  |
|  |  |  |  | member 37653 |  |  |
|  |  |  |  |  |  |  |
| LOC115530027 | F1 T7 |  |  | Very-long-chain enoyl-CoA reductase |  |  |
| LOC115530782 | F1 T7 |  |  | ATP-binding cassette, subfamily A (ABC1), member 5 |  |  |
| LOC115531027 | F0 T1 |  |  |  |  |  |
| LOC115531287 | F0 T7 |  |  | MFS transporter, MCT family, solute carrier family 16 |  |  |
|  |  |  |  | (monocarboxylic acid transporters), member 6 |  |  |
|  |  |  |  |  |  |  |
| LOC115531411 | F1 T7 |  |  | Thousand and one amino acid protein kinase |  |  |
| LOC115531431 | F0 T1 |  |  | ATP-dependent RNA helicase DHX8/PRP22 |  |  |
| LOC115531513 | F0 T1 |  |  | Alpha-N-acetylgalactosaminide alpha-2,6-sialyltransferase |  |  |
|  |  |  |  | (sialyltransferase 7B) |  |  |
|  |  |  |  |  |  |  |
| LOC115531543 | F0 T4 |  |  | Alpha-N-acetylgalactosaminide alpha-2,6-sialyltransferase |  |  |
|  |  |  |  | (sialyltransferase 7A) /Matrix remodeling-associated protein 7 |  |  |
|  |  |  |  |  |  |  |
|  |  |  |  |  |  |  |
| LOC115532063 | F1 T7 |  |  |  |  |  |
| LOC115532277 | F1 T7 |  |  |  |  |  |
| LOC115532547 | F1 T7 |  |  | Type I protein arginine methyltransferase |  |  |
| LOC115532710 | F1 T7 |  |  | Homeobox protein HB9 |  |  |
| LOC115532841 | F1 T7 |  |  |  |  |  |
| LOC115533343 | F0 T1 |  |  | Chromobox protein 1 |  |  |
| LOC115533457 | F1 T7 |  |  | Potassium voltage-gated channel Isk-related subfamily E |  |  |
|  |  |  |  | member 2 |  |  |
|  |  |  |  |  |  |  |
| LOC115533512 | F1 T7 |  |  |  |  |  |
| LOC115533975 | F1 T7 |  |  |  |  |  |

| LOC115534169 | F1 T7 |  |  |  |  |  |
| --- | --- | --- | --- | --- | --- | --- |
| LOC115535637 | F0 T4 |  |  | Splicing factor, proline- and glutamine-rich |  |  |
| LOC115535639 | F1 T7 |  |  |  |  |  |
| LOC115537194 | F1 T7 |  |  | Claudin |  |  |
| LOC115537425 | F0 T1 |  |  |  |  |  |
| LOC115538212 | F1 T7 |  |  | NLR family CARD domain-containing protein 3 |  |  |
| LOC115538223 | F1 T7 |  |  |  |  |  |
| LOC115539795 | F1 T7 |  |  | HtrA serine peptidase 3 |  |  |
| LOC115539797 | F1 T7 |  |  | Prostaglandin E receptor 1 | Pathways in cancer |  |
| LOC115539803 | F0 T4 |  |  | Gprotein-coupled receptor 26 |  |  |
| LOC115539857 | F0 T1 |  |  | Pentatricopeptide repeat domain-containing protein 1 |  |  |
| LOC115540156 | F1 T7 |  |  |  |  |  |
| LOC115540218 | F1 T7 |  |  | General transcription factor IIIA/KRAB domain-containing zinc |  |  |
|  |  |  |  | finger protein |  |  |
|  |  |  |  |  |  |  |
| LOC115540474 | F1 T7 |  |  | Receptor-type tyrosine-protein phosphatase delta |  |  |
| LOC115540511 | F1 T7 |  |  | Protocadherin delta 2 |  |  |
| LOC115541107 | F1 T7 |  |  |  |  |  |
| LOC115541533 | F1 T7 |  |  |  |  |  |
| LOC115541713 | F1 T7 |  |  | Striated muscle-specific serine/threonine protein kinase |  |  |
|  |  |  |  |  |  |  |
| LOC115542055 | F0 T1 |  |  | Interleukin 6 signal transducer | Pathways in cancer/Signaling pathways |  |
|  |  |  |  |  | regulating pluripotency of stem cells |  |
|  |  |  |  |  |  |  |
| LOC115542486 | F1 T7 |  |  | Tenascin | MicroRNAs in cancer |  |
| LOC115542790 | F1 T7 |  |  | Semaphorin 3 | Axon guidance |  |
| LOC115542875 | F1 T7 |  |  |  |  |  |
| LOC115542962 | F1 T7 |  |  | Activating transcription factor 7 |  |  |
| LOC115543123 | F1 T4 |  |  |  |  |  |
| LOC115543138 | F1 T7 |  |  |  |  |  |
| LOC115543396 | F1 T7 |  |  |  |  |  |
| LOC115543445 | F1 T7 |  |  | Rab11 family-interacting protein 38384 |  |  |
| LOC115543632 | F0 T4 |  |  |  |  |  |
| LOC115543736 | F1 T7 |  |  |  |  |  |
| LOC115543738 | F1 T7 |  |  |  |  |  |

|  |  |  |  | Solute carrier family 5 (sodium-coupled monocarboxylate |  |  |
| --- | --- | --- | --- | --- | --- | --- |
| LOC115543802 | F0 T7 |  |  | transporter), member 45268/Solute carrier family 5 (sodium- |  |  |
|  |  |  |  | dependent multivitamin transporter), member 6/Solute:Na+ |  |  |
|  |  |  |  |  |  |  |
|  |  |  |  | symporter, SSS family |  |  |
|  |  |  |  |  |  |  |
| LOC115543967 | F1 T7 |  |  | Transcription factor CP2 and related proteins |  |  |
| LOC115544350 | F1 T7 |  |  | Potassium channel subfamily K member 10 |  |  |
| LOC115544501 | F1 T7 |  |  |  |  |  |
| LOC115545287 | F1 T7 |  |  |  |  |  |
| LOC115545317 | F0 T7 |  |  |  |  |  |
| LOC115545509 | F1 T7 |  |  |  |  |  |
| LOC115545564 | F1 T7 |  |  |  |  |  |
| LOC115545721 | F1 T7 |  |  |  |  |  |
| LOC115545727 | F1 T7 |  |  | Leukemia inhibitory factor receptor | Signaling pathways regulating pluripotency |  |
|  |  |  |  |  | of stem cells |  |
|  |  |  |  |  |  |  |
| LOC115545832 | F1 T7 |  |  |  |  |  |
| LOC115546014 | F1 T7 |  |  |  |  |  |
| LOC115546523 | F0 T7 |  |  |  |  |  |
| LOC115547266 | F1 T7 |  |  | Cell adhesion molecule 1 |  |  |
| LOC115547369 | F1 T7 |  |  | General transcription factor IIIA/KRAB domain-containing zinc |  |  |
|  |  |  |  | finger protein/KRAB domain-containing zinc finger protein |  |  |
|  |  |  |  |  |  |  |
|  |  |  |  |  |  |  |
| LOC115547409 | F0 T7 |  |  | Ubiquitin carboxyl-terminal hydrolase 28 |  |  |
| LOC115547431 | F0 T1 |  |  | E3 ubiquitin-protein ligase RNF167 |  |  |
| LOC115547526 | F1 T7 |  |  | Band 4.1-like protein 37653 |  |  |
| LOC115547651 | F1 T7 |  |  |  |  |  |
| LOC115547902 | F1 T7 |  |  | Nicotinic acetylcholine receptor epsilon |  |  |
| LOC115548306 | F1 T7 |  |  |  |  |  |
| LOC115548611 | F1 T7 |  |  |  |  |  |
| LOC115548954 | F0 T1 | F0 T7 |  |  |  |  |
| LOC115549079 | F0 T1 |  |  |  |  |  |
| LOC115549407 | F0 T7 | F1 T7 |  | 25/26-hydroxycholesterol 7alpha-hydroxylase |  |  |
| LOC115549437 | F1 T7 |  |  | Protein fem-1 homolog A/C |  |  |

| LOC115549650 | F1 T7 |  |  | Pleckstrin homology domain-containing family G member 4 |  |  |
| --- | --- | --- | --- | --- | --- | --- |
|  |  |  |  |  |  |  |
| LOC115550375 | F1 T7 |  |  |  |  |  |
| LOC115550686 | F0 T1 |  |  |  |  |  |
| LOC115551200 | F1 T7 |  |  | Solute carrier family 25 (mitochondrial glutamate transporter), |  |  |
|  |  |  |  | member 18/22 |  |  |
|  |  |  |  |  |  |  |
| LOC115551515 | F0 T7 |  |  | SH3 and multiple ankyrin repeat domains protein |  |  |
| LOC115552031 | F1 T7 |  |  |  |  |  |
| LOC115552122 | F1 T7 |  |  | Solute carrier family 44 (choline transporter-like protein), |  |  |
|  |  |  |  | member 1 |  |  |
|  |  |  |  |  |  |  |
| LOC115552143 | F1 T4 |  |  | FMS-like tyrosine kinase 4 | Pathways in cancer |  |
| LOC115552356 | F1 T7 |  |  | Protein RD3 |  |  |
| LOC115552372 | F0 T1 |  |  |  |  |  |
| LOC115552778 | F1 T7 |  |  | Gap junction alpha-3 protein |  |  |
| LOC115552914 | F1 T7 |  |  |  |  |  |
| LOC115553461 | F0 T1 |  |  |  |  |  |
| LOC115553957 | F1 T7 |  |  | NADH dehydrogenase (ubiquinone) flavoprotein 1 |  |  |
| LOC115553976 | F1 T7 |  |  |  |  |  |
| LOC115554274 | F1 T7 |  |  | Carnitine O-octanoyltransferase |  |  |
| LOC115554307 | F1 T7 |  |  |  |  |  |
| LOC115555691 | F1 T7 |  |  |  |  |  |
| LOC115556269 | F1 T7 |  |  | Palladin |  |  |
| LOC115556758 | F0 T1 |  |  |  |  |  |
| LOC115557094 | F1 T7 |  |  | Retinoic acid receptor gamma |  |  |
| LOC115557395 | F1 T7 |  |  | Hyaluronoglucosaminidase |  |  |
| LOC115557498 | F1 T7 |  |  |  |  |  |
| LOC115557571 | F0 T7 |  |  | Activin receptor type-1B | Signaling pathways regulating pluripotency |  |
|  |  |  |  |  | of stem cells |  |
|  |  |  |  |  |  |  |
| LOC115558318 | F1 T7 |  |  |  |  |  |
| LOC115558322 | F1 T7 |  |  | Sulfotransferase |  |  |
| LOC115558646 | F0 T7 |  |  | Solute carrier family 27 (fatty acid transporter), member 2 |  |  |
|  |  |  |  |  |  |  |
| LOC115558705 | F0 T1 |  |  | RAR-related orphan receptor alpha |  |  |

| LOC115558993 | F1 T7 |  |  | Transcription initiation factor TFIID subunit 4 |  |  |
| --- | --- | --- | --- | --- | --- | --- |
| LOC115559006 | F1 T1 |  |  | All-trans-retinol 3,4-desaturase |  |  |
| LOC115559079 | F1 T7 |  |  | Ribosomal protein S6 kinase alpha-1/2/3/6 |  |  |
| LOC115559085 | F1 T7 |  |  | DENN domain-containing protein 4 |  |  |
| LOC115559197 | F1 T7 |  |  |  |  |  |
| LOC115559271 | F1 T7 |  |  |  |  |  |
| LOC115559272 | F1 T7 |  |  |  |  |  |
| LOC115559313 | F1 T7 |  |  |  |  |  |
| LOC115559319 | F1 T7 |  |  |  |  |  |
| LOC115559836 | F1 T7 |  |  | Multiple inositol-polyphosphate phosphatase / 2,3- |  |  |
|  |  |  |  | bisphosphoglycerate 3-phosphatase |  |  |
|  |  |  |  |  |  |  |
| LOC115559894 | F0 T7 |  |  | Phosphatidylinositol-3,4,5-trisphosphate 3-phosphatase and | Pathways in cancer/MicroRNAs in cancer |  |
|  |  |  |  | dual-specificity protein phosphatase PTEN |  |  |
|  |  |  |  |  |  |  |
|  |  |  |  |  |  |  |
| LOC115560095 | F1 T7 |  |  | Leucine-rich repeat LGI family member 1 |  |  |
| LOC115560097 | F0 T7 |  |  | Myopalladin |  |  |
| LOC115560310 | F1 T7 |  |  |  |  |  |
| LOC115561614 | F1 T7 |  |  | Fer-1-like protein 6 |  |  |
| lurap1l | F1 T7 |  |  |  |  |  |
| matn2 | F1 T7 |  |  | Matrilin |  |  |
| mpg | F0 T1 |  |  | DNA-3-methyladenine glycosylase |  |  |
| mphosph8 | F1 T7 |  |  | M-phase phosphoprotein 8 |  |  |
| mtrr | F1 T7 |  |  | Methionine synthase reductase |  |  |
| mybpc2 | F1 T7 |  |  | Myosin-binding protein C, fast-type |  |  |
| ncoa3 | F1 T7 |  |  | Nuclear receptor coactivator 3 | Pathways in cancer |  |
| nsmaf | F0 T1 |  |  | Factor associated with neutral sphingomyelinase activation |  |  |
|  |  |  |  |  |  |  |
| ogfod3 | F0 T7 |  |  |  |  |  |
| panx3 | F1 T7 |  |  | Homeobox protein PKNOX/Pannexin 44987 |  |  |
| parp16 | F1 T7 |  |  | Poly |  |  |
| pcdh8 | F1 T7 |  |  | Protocadherin delta 2 |  |  |
| pcgf6 | F0 T1 |  |  | Polycomb group RING finger protein 6 | Signaling pathways regulating pluripotency |  |
|  |  |  |  |  | of stem cells |  |
|  |  |  |  |  |  |  |

| pde4dip | F0 T7 |  |  | Myomegalin |  |  |
| --- | --- | --- | --- | --- | --- | --- |
| prg4 | F1 T7 |  |  |  |  |  |
| ptpn11 | F1 T7 |  |  | Tyrosine-protein phosphatase non-receptor type 11 | Axon guidance |  |
| ripor2 | F1 T7 |  |  | Rho family-interacting cell polarization regulator |  |  |
| rnf8 | F0 T1 |  |  | E3 ubiquitin-protein ligase RNF8 |  |  |
| rpa1 | F0 T7 |  |  | Replication factor A1 |  |  |
| rpgrip1l | F1 T7 |  |  | Protein fantom |  |  |
| sgcd | F1 T7 |  |  | Delta-sarcoglycan |  |  |
| slc12a4 | F1 T7 |  |  | Solute carrier family 12 (potassium/chloride transporter), |  |  |
|  |  |  |  | member 45081 |  |  |
|  |  |  |  |  |  |  |
| slc33a1 | F0 T1 |  |  | MFS transporter, PAT family, solute carrier family 33 (acetyl- |  |  |
|  |  |  |  | CoA transportor), member 1 |  |  |
|  |  |  |  |  |  |  |
| slc35f5 | F0 T4 |  |  | Solute carrier family 35, member F5 |  |  |
| slc44a5 | F0 T1 | F0 T4 |  | Solute carrier family 44 (choline transporter-like protein), |  |  |
|  |  |  |  | member 38444 |  |  |
|  |  |  |  |  |  |  |
| snx16 | F1 T7 |  |  | Sorting nexin-16 |  |  |
| spata2l | F1 T7 |  |  | Spermatogenesis-associated protein 2 |  |  |
| ss18 | F1 T7 |  |  | Protein SSXT |  |  |
| syt6 | F1 T7 |  |  | Synaptotagmin-6 |  |  |
| tasp1 | F1 T7 |  |  | Taspase, threonine aspartase, 1 |  |  |
| tex30 | F1 T7 |  |  | Uncharacterized protein |  |  |
| thnsl1 | F0 T1 |  |  | Threonine synthase |  |  |
| tmem106b | F0 T1 |  |  | Transmembrane protein 106B |  |  |
| tmem175 | F0 T4 | F0 T7 |  | TMEM175 potassium channel family protein |  |  |
| tmem250 | F1 T7 |  |  |  |  |  |
| tp53i11 | F1 T7 |  |  |  |  |  |
| trafd1 | F1 T7 |  |  |  |  |  |
| trmt10c | F0 T1 |  |  | Mitochondrial ribonuclease P protein 1 |  |  |
| ttc23 | F0 T1 |  |  | Tetratricopeptide repeat protein 23 |  |  |
| tut4 | F0 T1 | F0 T4 |  | Terminal uridylyltransferase |  |  |
| unc5b | F1 T7 |  |  | Netrin receptor unc-5 | Axon guidance |  |
| wipf1 | F0 T1 |  |  | WAS/WASL-interacting protein |  |  |
